# A Membrane-Bound Cytochrome Enables *Methanosarcina acetivorans* To Conserve Energy from Extracellular Electron Transfer

**DOI:** 10.1128/mBio.00789-19

**Published:** 2019-08-20

**Authors:** Dawn E. Holmes, Toshiyuki Ueki, Hai-Yan Tang, Jinjie Zhou, Jessica A. Smith, Gina Chaput, Derek R. Lovley

**Affiliations:** a Department of Microbiology, University of Massachusetts—Amherst, Morrill IV N Science Center, Amherst, Massachusetts, USA; b Department of Physical and Biological Sciences, Western New England University, Springfield, Massachusetts, USA; c Jiangsu Provincial Key Lab for Organic Solid Waste Utilization, National Engineering Research Center for Organic-based Fertilizers, Jiangsu Collaborative Innovation Center for Solid Organic Waster Resource Utilization, Nanjing Agricultural University, Nanjing, China; d School of Life Science and Biotechnology, Dalian University of Technology, Dalian, Liaoning Province, China; e Department of Biomolecular Sciences, Central Connecticut State University, New Britain, Connecticut, USA; Max Planck Institute for Marine Microbiology

**Keywords:** AQDS reduction, *Methanosarcina*, *c*-type cytochrome, extracellular electron transfer, genetics, transcriptome

## Abstract

Extracellular electron exchange in *Methanosarcina* species and closely related *Archaea* plays an important role in the global carbon cycle and enhances the speed and stability of anaerobic digestion by facilitating efficient syntrophic interactions. Here, we grew Methanosarcina acetivorans with methanol provided as the electron donor and the humic analogue, anthraquione-2,6-disulfonate (AQDS), provided as the electron acceptor when methane production was inhibited with bromoethanesulfonate. AQDS was reduced with simultaneous methane production in the absence of bromoethanesulfonate. Transcriptomics revealed that expression of the gene for the transmembrane, multiheme, *c*-type cytochrome MmcA was higher in AQDS-respiring cells than in cells performing methylotrophic methanogenesis. A strain in which the gene for MmcA was deleted failed to grow via AQDS reduction but grew with the conversion of methanol or acetate to methane, suggesting that MmcA has a specialized role as a conduit for extracellular electron transfer. Enhanced expression of genes for methanol conversion to methyl-coenzyme M and the Rnf complex suggested that methanol is oxidized to carbon dioxide in AQDS-respiring cells through a pathway that is similar to methyl-coenzyme M oxidation in methanogenic cells. However, during AQDS respiration the Rnf complex and reduced methanophenazine probably transfer electrons to MmcA, which functions as the terminal reductase for AQDS reduction. Extracellular electron transfer may enable the survival of methanogens in dynamic environments in which oxidized humic substances and Fe(III) oxides are intermittently available. The availability of tools for genetic manipulation of *M. acetivorans* makes it an excellent model microbe for evaluating *c*-type cytochrome-dependent extracellular electron transfer in *Archaea*.

## Abstract (precis)

The discovery of a methanogen that can conserve energy to support growth solely from the oxidation of organic carbon coupled to the reduction of an extracellular electron acceptor expands the possible environments in which methanogens might thrive. The potential importance of *c*-type cytochromes for extracellular electron transfer to syntrophic bacterial partners and/or Fe(III) minerals in some *Archaea* was previously proposed, but these studies with Methanosarcina acetivorans provide the first genetic evidence for cytochrome-based extracellular electron transfer in *Archaea*. The results suggest parallels with Gram-negative bacteria, such as *Shewanella* and *Geobacter* species, in which multiheme outer-surface *c*-type cytochromes are an essential component for electrical communication with the extracellular environment. *M. acetivorans* offers an unprecedented opportunity to study mechanisms for energy conservation from the anaerobic oxidation of one-carbon organic compounds coupled to extracellular electron transfer in *Archaea* with implications not only for methanogens but possibly also for *Archaea* that anaerobically oxidize methane.

## INTRODUCTION

Extracellular electron exchange is central to the environmental function of diverse *Archaea* that oxidize and/or produce methane. Some methanogens can divert electrons from methane production to the reduction of extracellular electron carriers such as Fe(III), U(VI), V(IV), and anthraquinone-2,6-disulfonate (AQDS), a humic acid analog (1–9). Diversion of electron flux from methane production to extracellular electron transfer may influence the extent of methane production and metal geochemistry in anaerobic soils and sediments. Methanogens such as *Methanothrix* (formerly *Methanosaeta*) and *Methanosarcina* species can accept electrons via direct interspecies electron transfer from electron-donating partners, such as *Geobacter* species, in important methanogenic environments such as anaerobic digesters and rice paddy soils (10–12). Anaerobic methane oxidation also plays an important role in the global carbon cycle and diverse anaerobic methane-oxidizing archaea (ANME) transfer electrons derived from methane oxidation to extracellular electron acceptors, such as other microbial species, Fe(III), or extracellular quinones (13–20). The electrical contacts for extracellular electron exchange have yet to be definitively identified in any of these *Archaea*.

It has been hypothesized that outer-surface cytochromes enable electron transfer to electron-accepting microbial partners or Fe(III) in some ANME (13–19). Genes for multiheme *c*-type cytochromes that are present in ANME genomes can be highly expressed and in some instances the encoded proteins have been detected (14, 19). The putative function of outer-surface cytochromes is terminal electron transfer to extracellular electron acceptors, similar to the role that outer surface *c*-type cytochromes play in extracellular electron transfer in Gram-negative bacteria such as *Shewanella* and *Geobacter* species (21–23). Similar *c*-type cytochrome electrical contacts have been proposed for Fe(III)-reducing *Archaea*, such as *Ferroglobus* and *Geoglobus* species (24–26). However, the study of the mechanisms for extracellular electron transfer in these archaea has been stymied by the lack of microorganisms available in pure culture that can grow via extracellular electron transfer and are genetically tractable.

Tools are available for genetic manipulation of the methanogen Methanosarcina acetivorans (27–29). A methyl‐coenzyme M reductase from an uncultured ANME was introduced into *M. acetivorans* to generate a strain that could convert methane to acetate with simultaneous reduction of Fe(III) (30). Most of the electrons from the methane consumed were recovered in acetate (30), and it was not shown that energy was conserved from Fe(III) reduction. *In vitro* reactions catalyzed by membrane vesicles of wild-type *M. acetivorans* suggested that the membrane-bound heterodisulfide reductase HdrDE reduced Fe(III)-citrate and AQDS and that an outer-surface multiheme *c*-type cytochrome might also function as a potential electron donor for Fe(III)-citrate reduction (31). However, *in vitro* assays with cell components are not a definitive approach for determining the physiologically relevant mechanisms involved in the reduction of Fe(III) and AQDS. This is because constituents that do have access to extracellular electron acceptors *in vivo* are exposed to extracellular electron acceptors *in vitro* and many reduced cofactors and redox-proteins, including *c*-type cytochromes, can nonspecifically reduce these electron acceptors (32). Analysis of the phenotypes of intact cells that result from specific gene deletions can provide more conclusive evidence.

We report here that *M. acetivorans* can be grown without methane production with AQDS as the sole electron acceptor. Analysis of gene expression patterns and phenotypes of gene deletion strains suggest a mechanism for energy conservation during extracellular electron transfer.

## RESULTS AND DISCUSSION

### Growth of *M*. *acetivorans* with AQDS as the sole terminal electron acceptor.

**FIG 1:**
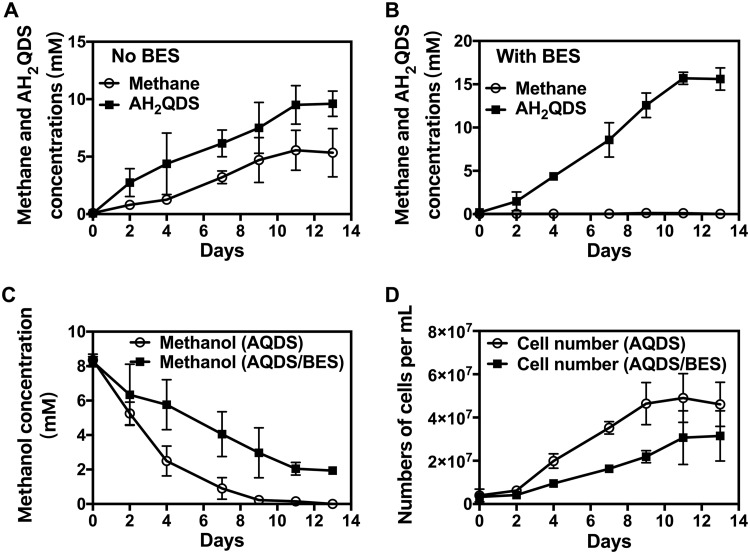
Growth of M. acetivorans with methanol provided as an electron donor and AQDS as an electron acceptor in the presence or absence of BES. (A) Methane and AH_2_QDS concentrations generated by cultures grown without BES. (B) Methane and AH_2_QDS concentrations generated by cultures grown with BES. (C and D) Methanol concentrations (C) and cell numbers (D) from cultures grown in the presence or absence of BES. The complete inhibition of methane production in the presence of BES is also shown on an expanded scale in Fig. S1.

In medium with methanol provided as the electron donor and AQDS as a potential electron acceptor, *M. acetivorans* simultaneously produced methane and reduced AQDS (Fig. 1a). The addition of bromoethanesulfonate (BES) inhibited methane production and increased the extent of AQDS reduction (Fig. 1B; Fig. S1). The metabolism of methanol (Fig. 1c) was accompanied by an increase in cell numbers (Fig. 1D). In the BES-amended cultures, 6.3 ± 0.43 mM (mean of triplicate cultures ± the standard deviation) methanol was consumed with the reduction of 15.7 ± 0.61 mM AQDS. When the need to divert some of the methanol metabolized to cell biomass is considered, this stoichiometry is consistent with the oxidation of methanol to carbon dioxide, with AQDS serving as the sole electron acceptor: CH_3_OH + 3AQDS + H_2_O → 3AH_2_QDS + CO_2_. Methanol consumption stopped once all the AQDS was reduced in the BES-amended cultures (Fig. 1C). However, in the absence of BES, all of the methanol could be consumed because methanol was also converted to methane.

10.1128/mBio.00789-19.1 FIG S1 Methane concentrations detected in Methanosarcina acetivorans cultures grown with methanol as the electron donor and AQDS as the electron acceptor in the presence of the methanogenic inhibitor BES. Each point and error bars represent the average and standard deviation of triplicate measurements. Download FIG S1, TIF file, 1.5 MB. Copyright © 2019 Holmes et al. 2019 Holmes et al. This content is distributed under the terms of the Creative Commons Attribution 4.0 International license.

The growth of *M. acetivorans* with AQDS as the sole electron acceptor (Fig. 1) is the first example of a methanogen conserving energy to support growth with electron transfer to an external electron acceptor. The ability of *M. acetivorans* to grow in this manner and the availability of tools for genetic manipulation (27–29) provide an opportunity for functional analysis of extracellular electron transfer by an archaeon.

### Transcriptomics and gene deletion studies demonstrate that the multiheme *c*-type cytochrome MmcA is involved in AQDS reduction.

In order to obtain insight into potential electron carriers involved in AQDS reduction, the transcriptome of cells grown with AQDS as the sole electron acceptor in the presence of BES was compared to the transcriptome of cells grown with methanol in the absence of AQDS or BES, so that methane production was the sole route of electron flux. The generation time (0.69 ± 0.13 days) of the cells grown via methanogenesis (Fig. S2) was longer than previously reported (generation time, 6.3 h; [33]). The lower growth rate in our study might be due to the omission of cysteine and lower sulfide content in our medium (0.3 mM compared to 0.5 mM) in order to reduce medium constituents that might abiotically reduce AQDS. However, methanogenic growth was ∼4-fold faster than growth via AQDS respiration in the presence of BES (generation time, 2.9 ± 0.18 days). Consistent with the lower growth rate, most of the genes related to cell growth (amino acid biosynthesis; protein synthesis; biosynthesis of purines, pyrimidines, nucleosides, and nucleotides; and transcription) had greater expression in methanogenic cells than cells grown via AQDS respiration (see Table S1B in the supplemental material).

10.1128/mBio.00789-19.2 FIG S2 Methanol (A), methane (B), AQDS concentrations (C), and cell numbers (D) in control cultures. All points and error bars represent the average and standard deviation for triplicate samples. For methanogenic conditions, Methanosarcina acetivorans cells were grown with methanol as the substrate. For the cells/BES/methanol condition, methanogenesis was inhibited with BES, and no electron acceptor (AQDS) was provided. For the cells/BES/AQDS condition, cells were provided with electron acceptor (AQDS), methanogenesis was inhibited with BES, and no electron donor (methanol) was provided. For the no cells/BES/AQDS condition, BES and AQDS were added to the medium but no inoculum was added. Download FIG S2, TIF file, 1.5 MB. Copyright © 2019 Holmes et al. 2019 Holmes et al. This content is distributed under the terms of the Creative Commons Attribution 4.0 International license.

10.1128/mBio.00789-19.6 TABLE S1 (A) Differential expression of genes from M. acetivorans cells grown with methanol provided as electron donor and AQDS (with BES) as electron acceptor (experimental condition) versus cells grown via methylotrophic methanogenesis (control condition). Values were obtained with EdgeR software. Only genes with *P* values of ≥0.05 are included in this table. Positive log fold change values indicate that the gene was more highly transcribed in AQDS-respiring cells; negative log fold change values indicate that the gene was more highly transcribed in cells grown via methylotrophic methanogenesis. (B) Number of growth-related genes that were more highly expressed in M. acetivorans cells grown with methanol as the electron donor and AQDS as the electron acceptor in the presence of BES (experimental condition) or in *M. acetivorans* cells grown via methylotrophic methanogenesis (control condition). These results are a summary of data presented in more detail in Table S1A. Download Table S1, XLSX file, 0.3 MB. Copyright © 2019 Holmes et al. 2019 Holmes et al. This content is distributed under the terms of the Creative Commons Attribution 4.0 International license.

Remarkably, despite the lower growth rate on AQDS, the gene *MA0658*, which encodes a seven-heme, outer-surface *c*-type cytochrome, was 4.5-fold more highly expressed in AQDS-reducing versus methanogenic cells (Table 1). For future reference, this cytochrome was designated MmcA (membrane multiheme cytochrome A). Multiheme *c*-type cytochromes are of particular interest as potential electron carriers in extracellular electron transport because of the well-documented role of multiheme *c*-type cytochromes in bacteria such as *Shewanella* and *Geobacter* species that are highly effective in extracellular electron transfer (21–23). *MA3739*, a gene coding for a five-heme *c*-type cytochrome, was transcribed at similar levels as *mmcA*, and 4.1-fold-higher expression was detected in AQDS-reducing than methanogenic cells (Table 1).

**TABLE 1:** Differential expression of genes encoding *c*-type cytochrome proteins in *M. acetivorans* cells^*a*^

Locus	No. of:		Predicted localization	Fold upregulation^*b*^	*P*	FDR
	Heme groups	Transmembrane helices				
MA0658	7	1	Membrane	4.53	0.002	0.006
MA3739	5	0	Unknown	4.14	0.047	0.031
MA0167	1	1	Membrane	5.97	0.018	0.037
MA2925	2	1	Membrane	NS		
MA2908	2	1	Membrane	NS		

a Cells were grown with methanol provided as the electron donor and AQDS as the electron acceptor in the presence of BES or were grown via methanogenesis with methanol as the substrate. Genes were only considered differentially expressed if the *P* value and FDR (false discovery rate) were ≤0.05. NS, no significant difference in read abundance between conditions.

b That is, in AQDS/BES versus methanogenesis.

There are three other putative *c*-type cytochrome genes in the *M. acetivorans* genome (26). *MA0167*, which encodes a monoheme cytochrome with predicted localization in the cell membrane, was six times more highly expressed in cells grown via AQDS respiration (Table 1). Functional analysis of the outer membrane of G. sulfurreducens has suggested that a monoheme *c*-type cytochrome may play a role in regulating the expression of multiheme *c*-type cytochromes, possibly by providing a sensor function (34, 35). It is possible that the protein encoded by *MA0167* plays a similar role in *M. acetivorans*. The expressions of *MA2925* and *MA2908*, both of which encode two-heme *c*-type cytochromes, were comparable in AQDS-reducing versus methanogenic cells (Table 1). These cytochromes are homologous to methylamine utilization protein G (MauG) and the diheme cytochrome *c* peroxidase (CcpA). MauG is required for aerobic methylamine metabolism (36–38), and CcpA proteins reduce hydrogen peroxide to water and protect the cell from reactive oxygen species (39, 40). Thus, it seems unlikely that either of these cytochromes is involved in extracellular electron transfer.

**FIG 2:**
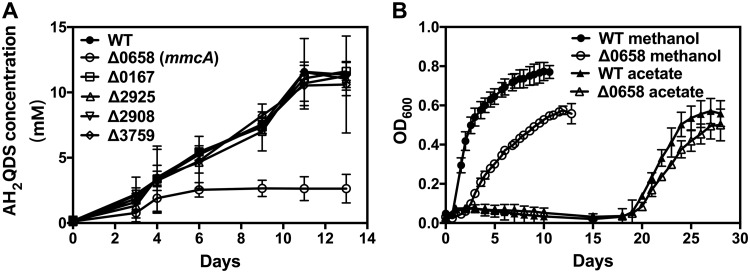
Impact of deletion of *c*-type cytochrome genes on growth of *M. acetivorans* under different conditions. (A) AH_2_QDS production during growth with methanol as the electron donor and AQDS as the acceptor in the presence of BES. The locus for the deleted cytochrome gene is designated next to the corresponding symbol. (B) Growth of wild-type and ΔMA0658 strains under methanogenic conditions as measured as the *A*_600_ with methanol or acetate provided as the substrates.

In order to evaluate the potential role of *c*-type cytochromes in AQDS reduction, deletion mutant strains were constructed in *M. acetivorans* for each *c*-type cytochrome gene in the genome (Table 1). Only the deletion of *mmcA* inhibited AQDS reduction (Fig. 2A). Deletion of *mmcA* had a slight impact on methanogenic growth with methanol (Fig. 2B). These results suggest that MmcA is a major component for extracellular electron transfer to AQDS but not for the conversion of methanol to methane.

Previous studies have suggested that MmcA is part of the Rnf complex, which is required for acetoclastic methanogenesis (41), and that *mmcA* is cotranscribed with Rnf genes located in the same region of the chromosome (42). However, deletion of the MmcA gene did not substantially impact growth on acetate (Fig. 2B) or transcription of other genes from the Rnf complex (Fig. S2). Furthermore, the expression profiles of *mmcA* and genes for the Rnf complex were also different (Tables 1 and 2).

**TABLE 2:** Comparison of transcripts from genes coding for components of the Rnf and Mrp complexes in *M. acetivorans* cells^*a*^

Locus	Description	Gene	Fold upregulation^*b*^	*P*	FDR
MA0659	Electron transport complex protein RnfC	*rnfC*	1.52	0.02	0.04
MA0660	Electron transport complex protein RnfD	*rnfD*	NS		
MA0661	Electron transport complex protein RnfG	*rnfG*	1.66	0.006	0.01
MA0662	Electron transport complex protein RnfE	*rnfE*	1.45	0.02	0.05
MA0663	Electron transport complex protein RnfA	*rnfA*	1.66	0.006	0.01
MA0664	Electron transport complex protein RnfB	*rnfB*	1.57	0.008	0.01
MA4572	Multisubunit sodium/proton antiporter, MrpA subunit	*mrpA*	5.44	5.77 × 10^–8^	5.07 × 10^–6^
MA4665	Multisubunit sodium/proton antiporter, MrpB subunit	*mrpB*	5.41	8.99 × 10^–8^	6.06 × 10^–6^
MA4570	Multisubunit sodium/proton antiporter, MrpC subunit	*mrpC*	6.50	7.25 × 10^–8^	5.71 × 10^–6^
MA4569	Multisubunit sodium/proton antiporter, MrpD subunit	*mrpD*	4.84	1.38 × 10^–7^	7.21 × 10^–6^
MA4568	Multisubunit sodium/proton antiporter, MrpE subunit	*mrpE*	3.70	3.79 × 10^–6^	5.56 × 10^–5^
MA4567	Multisubunit sodium/proton antiporter, MrpF subunit	*mrpF*	4.79	3.78 × 10^–7^	1.28 × 10^–5^
MA4566	Multisubunit sodium/proton antiporter, MrpG subunit	*mrpG*	4.57	3.39 × 10^–7^	1.20 × 10^–5^

a Cells were grown with methanol and AQDS in the presence of BES or were grown via methanogenesis with methanol as the substrate. Genes were only considered differentially expressed if the *P* value and FDR were ≤0.05. NS, no significant difference in read abundance.

b That is, in AQDS/BES versus methanogenesis.

### Model for electron transport to AQDS via MmcA.

**FIG 3:**
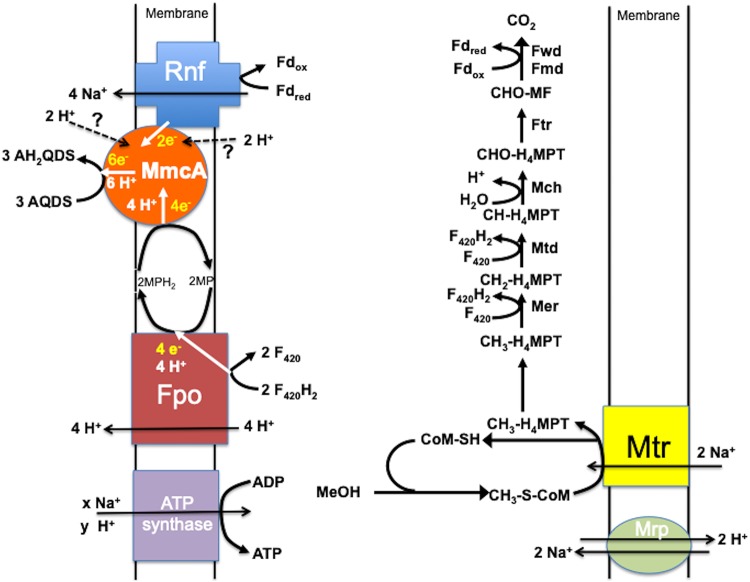
Proposed model for extracellular electron transport to AQDS by M. acetivorans when methanol is provided as the electron donor and methanogenesis is prevented by the addition of BES.

MmcA is a strong candidate for the terminal AQDS reductase because its localization in the cell membrane (42) is likely to provide access to AQDS and because of the well-known role of outer-membrane multiheme *c*-type cytochromes in reduction of AQDS and various forms of Fe(III) in Gram-negative bacteria such as *Shewanella* and *Geobacter* species (21–23, 43). It was previously suggested that MmcA could be a terminal reductase for the reduction of soluble Fe(III)-citrate, based on the *in vitro* oxidation of MmcA in membrane vesicles upon addition of Fe(III)-citrate (31). Such *in vitro* assays can be poor predictors of *in vivo* activity because Fe(III)-citrate typically oxidizes *c*-type cytochromes *in vitro*, regardless of physiological function, due to its very positive redox potential. However, as detailed below, multiple lines of evidence support a model in which energy can be conserved when MmcA serves as the terminal reductase during *in vivo* methanol oxidation coupled to AQDS reduction (Fig. 3).

During methane production from methanol, methanol is converted to CH_3_-CoM by the activity of three enzymes, methyltransferase 1 (MtaB), methyltransferase 2 (MtaA), and methanol corrinoid protein (MtaC) (44–46). The oxidation of one molecule of CH_3_-CoM to CO_2_ generates the reducing equivalents necessary to reduce three molecules of CH_3_-CoM to methane. During methanol oxidation coupled to AQDS reduction in the presence of BES, the step that reduces CH_3_-CoM to methane is blocked, but the option for CH_3_-CoM oxidation remains (Fig. 3). Genes coding for enzymes involved in the oxidation of CH_3_-CoM to carbon dioxide were more highly expressed in methanogenic cells, consistent with increased transcription of growth-related genes in methanogenic cells and the need for this pathway to generate reductants to support methanogenesis (Table S2).

10.1128/mBio.00789-19.7 TABLE S2 Differential expression of genes coding for proteins from the oxidative branch of the methylotrophic methanogenesis pathway and methyl-coenzyme M reductase (Mcr) in *M. acetivorans* cells grown with methanol and AQDS in the presence of BES or cells grown via methanogenesis with methanol as the substrate. Negative values indicate that genes were more significantly expressed in methanogenic cells. Genes were only considered differentially expressed if the *P* value and FDR (False Discovery Rate) were ≤0.05. NS: no significant difference in read abundance between conditions Download Table S2, DOCX file, 0.1 MB. Copyright © 2019 Holmes et al. 2019 Holmes et al. This content is distributed under the terms of the Creative Commons Attribution 4.0 International license.

Differential expression of genes encoding isomers of MtaB, MtaA, and MtaC suggested that there might be some differences in the route for methanol conversion to CH_3_-CoM (Table 3). The genes for the isomers MtaB1, MtaA1, and MtaC1 were more highly transcribed in methanogenic cells, whereas AQDS-respiring cells had greater expression of genes coding for the alternative MtaB, MtaA, and MtaC isomers (Table 3). Differences in the activity of these isomers are unknown, but in previous studies *mtaA1*, *mtaB1*, and *mtaC1* genes were highly transcribed during methanogenesis from methanol and MtaA1 was required for growth on methanol, whereas MtaA2 was dispensable (46).

**TABLE 3:** Differential expression of genes encoding methanol methyltransferase enzymes in *M. acetivorans* cells^*a*^

Locus	Annotation	Gene	Fold upregulation^*b*^	*P*	FDR
MA4379	Co-methyl-5-hydroxybenzimi- dazolylcobamide:2-mercapto-ethanesulphonic acid methyltransferase, isozyme 1	*mtaA1*	–1.68	0.01	0.02
MA0455	Methanol:5-hydroxybenzimidazolyl-cobamide methyltransferase, isozyme 1	*mtaB1*	–6.84	0.02	0.04
MA0456	Corrinoid-containing methyl-accepting protein, isozyme 1	*mtaC1*	–7.95	0.01	0.03
MA4392	Methanol:5-hydroxybenzimidazolylcobamide methyltransferase, isozyme 2	*mtaB2*	68.55	5.70 × 10^–11^	2.56 × 10^–7^
MA4391	Corrinoid-containing methyl-accepting protein, isozyme 2	*mtaC2*	48.28	3.27 × 10^–10^	5.54 × 10^–7^
MA1615	Co-methyl-5-hydroxybenzimidazolylcobamide:2- mercapto-ethanesulphonic acid methyltransferase, isozyme 2	*mtaA2*	5.39	1.77 × 10^–7^	8.04 × 10^–6^
MA1616	Methanol:5-hydroxybenzimidazolylcobamide methyltransferase, isozyme 3	*mtaB3*	9.66	5.24 × 10^–8^	4.89 × 10^–6^
MA1617	Corrinoid-containing methyl-accepting protein, isozyme 3	*mtaC3*	8.49	2.52 × 10^–7^	1.00 × 10^–5^

a Cells were grown with methanol provided as an electron donor and AQDS provided as an electron acceptor in the presence of BES or cells grown via methanogenesis with methanol as the substrate. Negative values indicate that genes were more significantly expressed in methanogenic cells. Genes were only considered differentially expressed if the *P* value and FDR were ≤0.05.

b That is, in AQDS/BES versus methanogenesis.

Oxidation of methanol to carbon dioxide is expected to yield reduced ferredoxin and reduced F_420_ (F_420_H_2_). It is likely that the Rnf complex oxidizes reduced ferredoxin with electron transfer to MmcA (47). Transcripts for genes coding for components of the Rnf complex were slightly higher (∼1.5-fold) than those in methanogenic cells (Table 2), suggesting an important role for the Rnf complex in energy conservation from methanol oxidation coupled to AQDS reduction.

In methanogenic cells, the membrane-bound Fpo complex (F_420_:methanophenazine oxidoreductase) oxidizes F_420_H_2_ derived from methanol oxidation with the reduction of methanophenazine and proton translocation (48–52). Transcription of all Fpo subunit genes was higher in methanogenic cells than AQDS-reducing cells, as expected because of the importance of Fpo in oxidizing F_420_H_2_ in cells producing methane (Table S3). However, all of the Fpo complex genes were also being actively expressed in AQDS-respiring cells, suggesting that Fpo is important for the oxidation of F_420_H_2_ generated in methanol-oxidizing, AQDS-reducing cells. The reduced methanophenazine that Fpo generates from F_420_H_2_ oxidation could transfer electrons to MmcA (41, 42, 47, 53). Although it has also been proposed that reduced methanophenazine may be able to directly transfer electrons to extracellular electron carriers in *M. acetivorans* (31), the requirement for MmcA for growth via AQDS reduction indicates that this is an unlikely route for AQDS reduction.

10.1128/mBio.00789-19.8 TABLE S3 Differential expression of genes coding for proteins from the proton-translocating F_420_:methanophenazine oxidoreductase (Fpo) complex in *M. acetivorans* cells grown with methanol as the electron donor and AQDS (in the presence of BES) as the acceptor versus cells grown via methylotrophic methanogenesis with methanol as the substrate. Negative values indicate that genes were more significantly expressed in methanogenic cells. Genes were only considered differentially expressed if the *P* value and FDR (false discovery rate) were <0.05. Download Table S3, DOCX file, 0.1 MB. Copyright © 2019 Holmes et al. 2019 Holmes et al. This content is distributed under the terms of the Creative Commons Attribution 4.0 International license.

In methanogenic cells, reduced methanophenazine could also donate electrons to the membrane-bound heterodisulfide reductase HdrDE (52, 54–59). *In vitro* evidence with membrane vesicles suggested that HdrDE can reduce AQDS with CoM-SH and CoB-SH oxidation to form CoM-S-S-CoB (31). However, the redox-active components of HdrE and HdrD responsible for electron transfer to an electron acceptor are embedded in the membrane and the cytoplasm, respectively (52), and thus unlikely to access extracellular AQDS *in vivo*. The relative expressions of *hdrD* and *hdrE* were slightly lower in AQDS-reducing cells than in methanogenic cells (Table S2). Furthermore, the inability of the MmcA-deficient strain to grow via AQDS reduction indicates that HdrDE is not capable of functioning as the sole AQDS reductase to support growth. Thus, based on the lack of strong evidence for a role for HdrDE, the likely simpler and more direct route for AQDS-dependent oxidation of reduced methanophenazine is electron transfer to MmcA.

Based on these considerations and current understanding of the function of the redox proteins involved (52, 60, 61), it is apparent that a net positive export of Na^+^ and H^+^ outside the cell membrane during AQDS respiration that can support the generation of ATP is feasible (Fig. 3). In this model, two Na^+^ must be translocated into the cell for the initial oxidation of CH_3_-S-CoM (62–64). Two moles of F_420_H_2_ and one mole of reduced ferrodoxin are generated per mole of CH_3_-S-CoM oxidized to carbon dioxide. Fpo oxidizes the F_420_H_2_ with H^+^ translocation and the reduction of methanophenazine (49–51). The reduced methanophenazine transfers electrons to MmcA, which reduces AQDS. The Rnf complex oxidizes the reduced ferredoxin coupled with Na^+^ translocation (41, 47, 65) and the reduction of MmcA. MmcA may transfer protons, as well as electrons during AQDS reduction, as observed in other *c*-type cytochromes (66–71). The ATP synthase couples both Na^+^ and H^+^ transport to ATP synthesis (72), but the H^+^/Na^+^ antiporter complex Mrp can be important for balancing external Na^+^/H^+^ ratios (73). Genes for Mrp were highly expressed in AQDS-reducing cells (Table 2).

Uncertainties in the stoichiometry of Na^+^/H^+^ transport per ATP synthesized and the total amount of H^+^ translocated prevent an accurate estimate of the theoretical ATP yield per mole of methanol oxidized with the reduction of AQDS. However, the proposed metabolic route suggests a likely mechanism for net ATP synthesis to support the observed growth of *M. acetivorans* with methanol oxidation coupled to AQDS reduction.

### Implications.

The discovery that *M. acetivorans* can conserve energy to support growth from the oxidation of a one-carbon compound coupled to the reduction of an extracellular electron acceptor has important implications for the biogeochemistry of anaerobic soils and sediments and provides a genetically tractable model microbe for further analysis of the mechanisms of extracellular electron transfer in *Archaea*. Humic substances and Fe(III) are often abundant extracellular electron acceptors in a wide variety of anaerobic soils and sediments, and their availability for microbial respiration can reduce the extent of methane production (74–77). Competition for electron donors between methanogens and Fe(III)- and humic-reducing microorganisms is one factor (78–80). However, the finding that some methanogens may conserve energy by reducing extracellular electron acceptors suggests a mechanism for methanogens to survive in environments in which Fe(III) and oxidized forms of humic substances are abundant and then rapidly switch to methane production as these extracellular electron acceptors are depleted.

A comprehensive survey of the ability of diverse methanogens to conserve energy to support growth from electron transport to extracellular electron acceptors is warranted. Most methanogens, including other *Methanosarcina* species, lack membrane-bound multiheme cytochromes like MmcA and would need other mechanisms for extracellular electron transfer. The findings that MmcA is not essential for methane production and that expression of *mmcA* was increased when AQDS served as an electron acceptor suggest that the primary role of MmcA is extracellular electron transfer. If so, the presence of MmcA further suggests that there are environments in which the capacity for extracellular electron transfer substantially benefits *M. acetivorans*.

A wide diversity of archaea are capable of extracellular electron transfer (81). For archaea such as Ferroglobus placidus (24), Geoglobus ahangari (25), and diverse ANME (13–19), it has been proposed that outer-membrane cytochromes are the terminal reductase. It also appears that methanogens have evolved efficient means of extracellular electron transport; however, the mechanisms are poorly understood. The rapid nonphysiological reduction of extracellular electron acceptors by a range of redox-active proteins and cofactors *in vitro* necessitates genetically tractable model organisms for physiologically relevant functional studies. Thus, *M. acetivorans* may serve as an important model organism for better understanding cytochrome-based extracellular electron transfer in *Archaea*.

## MATERIALS AND METHODS

### Strains and growth conditions.

Methanosarcina acetivorans strains were routinely cultured under strict anaerobic conditions at 37°C in a previously described (27) medium with either 8.5 mM methanol or 40 mM acetate provided as the substrates.

*M. acetivorans* mutant strains were constructed with *M. acetivorans* WWM1 (Δ*hpt*) (82) as the parent strain, as described previously (28). For the construction of *MA0658*, *MA3739*, *MA2908*, *MA0167*, and *MA2925* deletion strains, genes were replaced with the *pac* gene (puromycin resistance gene). First, regions 500 to 1,000 bp upstream and downstream from the target genes were amplified by PCR (see Table S4 and Fig. S4 in the supplemental material). The DNA fragments of the upstream and downstream regions of *MA0658* were digested with SacI/XbaI and EcoRI/XhoI. Upstream and downstream regions of *MA3739* were digested with SalI/XbaI and SacI/NotI. Upstream and downstream regions of *MA2908*, *MA0167*, and *MA2925* were digested with XhoI/HindIII and BamHI/NotI. The upstream fragment was ligated into the pJK3 plasmid (27). The downstream fragment was ligated into the pJK3 plasmid already containing the upstream fragment. This recombinant plasmid was then linearized and used for transformation. The deletion and replacement of all genes with *pac* was verified with primers (Table S4). All transformants were selected on medium supplemented with puromycin (2 μM final concentration), as previously described (27).

10.1128/mBio.00789-19.3 FIG S3 Reverse-transcription PCR with primers targeting two other genes from the Rnf complex in the Methanosarcina acetivorans genome, MA0659 and MA0664. RNA was extracted from cells of the MA0658 deletion mutant strain of *M. acetivorans* grown with acetate provided as the substrate for growth. Download FIG S3, TIF file, 1.5 MB. Copyright © 2019 Holmes et al. 2019 Holmes et al. This content is distributed under the terms of the Creative Commons Attribution 4.0 International license.

10.1128/mBio.00789-19.4 FIG S4 Steps involved in construction of the mutant strain ΔMA0658. The coding region from amino acid residues 54Cys to 457Arg was deleted. P1, P2, and P3 were primers utilized for verification of the mutant construct. Primer sequences used for construction and verification of this mutant strain and other strains included in this study are provided in Table S4. Download FIG S4, TIF file, 1.5 MB. Copyright © 2019 Holmes et al. 2019 Holmes et al. This content is distributed under the terms of the Creative Commons Attribution 4.0 International license.

10.1128/mBio.00789-19.9 TABLE S4 Primers used to construct various deletion mutant strains (restriction sites are underlined). Genetically recombinant plasmids were checked for accuracy by PCR with verification primers. Download Table S4, DOCX file, 0.1 MB. Copyright © 2019 Holmes et al. 2019 Holmes et al. This content is distributed under the terms of the Creative Commons Attribution 4.0 International license.

Additions of anthraquinone-2,6,-disulphonate (AQDS) were made from a concentrated stock to provide a final concentration of 16 mM. Cysteine was omitted from all cultures. When noted, 2-bromoethanesulfonate (BES) was added from a concentrated stock to provide a final concentration of 15 mM. Growth with AQDS was measured by determining numbers of cells stained with acridine orange with epifluorescence microscopy (83). For comparing methanogenic growth in wild-type and mutant cells, growth was monitored by spectrometry at an absorbance of 600 nm (84).

### Analytical techniques.

Methanol concentrations were monitored with a gas chromatograph equipped with a headspace sampler and a flame ionization detector (Clarus 600; Perkin-Elmer, Inc., San Jose, CA). Methane in the headspace was measured by gas chromatography with a flame ionization detector (Shimadzu, GC-8A) as previously described (85). The production of reduced AQDS reduction was monitored by spectrophotometry at an absorbance of 450 nm as previously described (86).

### RNA extraction.

Cells were harvested from triplicate 50 ml cultures of *M. acetivorans* grown with methanol (10 mM) provided as the electron donor and AQDS (16 mM) in the presence of the methanogenesis inhibitor BES (15 mM) or via methanogenesis with 40 mM methanol provided as the substrate. Cells were harvested when AQDS-respiring cultures had reduced ∼8 mM AQDS (midexponential phase) and when methanogenic cells reached an optical density at 600 nm of 0.5.

Cells were split into 50-ml conical tubes (BD Sciences), mixed with RNAProtect (Qiagen) in a 1:1 ratio, and pelleted by centrifugation at 3,000 × *g* for 15 min at 4°C. Pellets were then immediately frozen in liquid nitrogen and stored at –80°C. Total RNA was extracted from all six cell pellets according to the previously described protocol (87) and cleaned using an RNeasy minikit (Qiagen). All six RNA samples (three AQDS-respiring and three methanogenic) were then treated with Turbo DNA-free DNase (Ambion, Austin, TX). In order to ensure that samples were not contaminated with genomic DNA, PCR with primers targeting the 16S rRNA gene was done with RNA that had not been reverse transcribed. Further enrichment of mRNA was done with the MICROB*Express* kit (Ambion), according to the manufacturer’s instructions.

### RT-PCR analysis.

Total RNA was prepared from *M. acetivorans hpt* and Δ*MA0658* strains grown methanogenically with acetate (40 mM). Complementary DNA (cDNA) was prepared by reverse transcription with AMV reverse transcriptase (New England Biolabs, Ipswich, MA) with the primers TCAGCATGCCTCATTCCAAC (*MA0659*) or TCGCAGACAGCCTTAACGTC (*MA0664*) according to the manufacturer’s specifications. This cDNA was then used as a template for PCR with the following primers: CAGTGACCTCGCTTATGTCC/TCAGCATGCCTCATTCCAAC (*MA0695*) or TGTGGAGGTTGCGGATTTGC/TCGCAGACAGCCTTAACGTC (*MA0664*). The amplified fragments were analyzed by agarose gel electrophoresis.

### Illumina sequencing and data analysis.

Directional multiplex libraries were prepared with the ScriptSeq v2 RNA-Seq Library preparation kit (Epicentre), and paired-end sequencing was performed on a Hi-Seq 2000 platform at the Deep Sequencing Core Facility at the University of Massachusetts Medical School in Worchester, MA.

All raw data generated by Illumina sequencing were quality checked by visualization of base quality scores and nucleotide distributions with FASTQC (http://www.bioinformatics.babraham.ac.uk/projects/fastqc/). Initial raw nonfiltered forward and reverse sequencing libraries contained an average of 134,187,478 ± 20,358,059 reads that were ∼100 bp in length (Table S5). Sequences from all of the libraries were trimmed and filtered with Trimmomatic (88), with the sliding window approach set to trim bases with quality scores lower than 3, strings of 3+N’s, and reads with a mean quality score lower than 20. Bases were also cut from the start and end of reads that fell below a threshold quality of 3, and any reads smaller than 50 bp were eliminated from the library. These parameters yielded an average of 90,596,717 ± 23,433,670 quality reads per RNA-Seq library.

10.1128/mBio.00789-19.10 TABLE S5 (A) Summary of statistics from RNAseq libraries assembled from RNA extracted from Methanosarcina acetivorans cells grown via methylotrophic methanogenesis with methanol provided as the substrate (3 biological replicates). (B) Summary of statistics from RNAseq libraries assembled from RNA extracted from Methanosarcina acetivorans cells grown with methanol provided as the electron donor and AQDS as the electron acceptor in the presence of BES (3 biological replicates). (C) The glmQLFit() function was used to fit the negative binomial generalized log-linear model for each tag. Fitted values for each gene and biological replicate (three AQDS/BES [AQB] and three methanol [Met] samples) are provided in the table below. For differential expression analysis of these genes, the glmQLFTest() function was used, which applies empirical Bayes quasi-likelihood (QL) F tests to the fitted values. Download Table S5, DOCX file, 0.9 MB. Copyright © 2019 Holmes et al. 2019 Holmes et al. This content is distributed under the terms of the Creative Commons Attribution 4.0 International license.

All paired-end reads were then merged with FLASH (89), resulting in 40,312,494 ± 8,686,910 reads with an average read length of 145 bp. After merging the QC-filtered reads, SortMeRNA (90) was used to separate all rRNA reads from nonribosomal reads, and this resulted in 30,679,551 ± 6,275,120 mRNA reads.

### Mapping of mRNA reads.

Trimmed and filtered mRNA reads from the triplicate samples for the two different culture conditions were mapped against the *M. acetivorans* strain C2A genome (NC_003552) downloaded from IMG/MER (img.jgi.doe.gov) using ArrayStar software (DNAStar). Analyses of reads from all three biological replicates for each condition demonstrated that the results were highly reproducible (Table S5 and Fig. S5).

10.1128/mBio.00789-19.5 FIG S5 Figure showing QuarterRoot mean deviance versus average log_2_ cpm (counts per million). The raw QL dispersion estimates are represented by black dots. The trend line is the mean dependent trend that was fitted to the raw QL dispersion estimates. The raw estimates were then squeezed toward this trend, called EB estimates (red dots). EBs were used in place of raw values for downstream hypothesis testing because the EB strategy reduces the uncertainty of the estimates and improves testing power. Download FIG S5, TIF file, 1.5 MB. Copyright © 2019 Holmes et al. 2019 Holmes et al. This content is distributed under the terms of the Creative Commons Attribution 4.0 International license.

Reads were normalized and processed for differential expression studies using the edgeR package in Bioconductor (91), with AQDS/BES considered the experimental condition and methanol the control. Genes with *P* values of ≤0.05 were considered differentially expressed. Using these criteria, 1,188 genes were downregulated, 2,121 genes were not differentially expressed, and 1,182 genes were upregulated (Table S1).

### Genome data analysis.

Gene sequence data for *M. acetivorans* C2A was acquired from the U.S. Department of Energy Joint Genome Institute (http://www.jgi.doe.gov) or from GenBank at the National Center for Biotechnology Information (NCBI; https://www.ncbi.nlm.nih.gov). Initial analyses were done with tools available on the Integrated Microbial Genomes (IMG) website (img.jgi.doe.gov). Some protein domains were identified with NCBI conserved domain search (92) and Pfam search (93) functions. Transmembrane helices were predicted with TMpred (94), TMHMM (95), and HMMTOP (96), and signal peptides were identified with PSORTb v3.0.2 (97) and Signal P v4.1 (98).

### Data availability.

Illumina sequence reads have been submitted to the SRA NCBI database under BioProject PRJNA509433 and Biosample SAMN10580613 (SRX5113605 to SRX5113610).
