# Supplementary figures and images for: A Membrane-Bound Cytochrome Enables Methanosarcina acetivorans To Conserve Energy from Extracellular Electron Transfer
Source: mBio. 2019 Aug 20;10(4):e00789-19. doi: 10.1128/mBio.00789-19 (PMC6703419; doi:10.1128/mBio.00789-19)

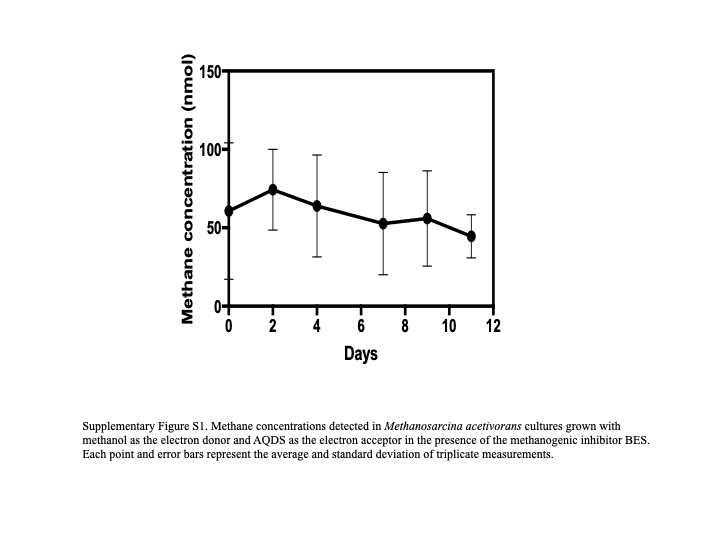

Supplement: FIG S1 [file mBio.00789-19-sf001.tif]

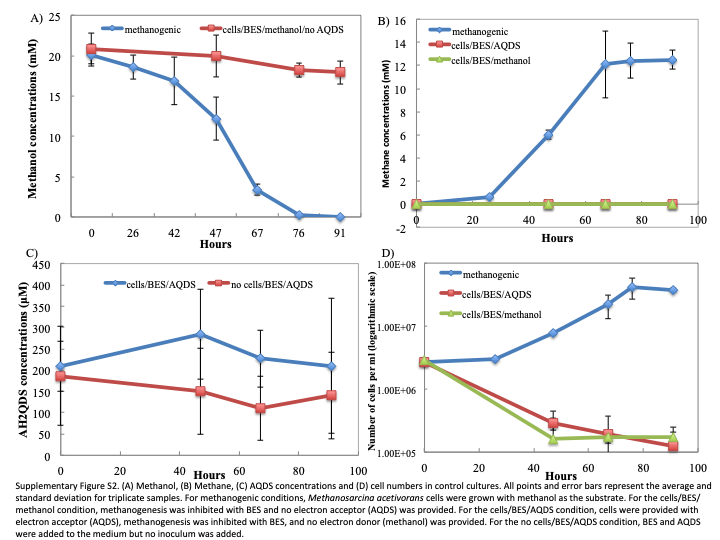

Supplement: FIG S2 [file mBio.00789-19-sf002.tif]

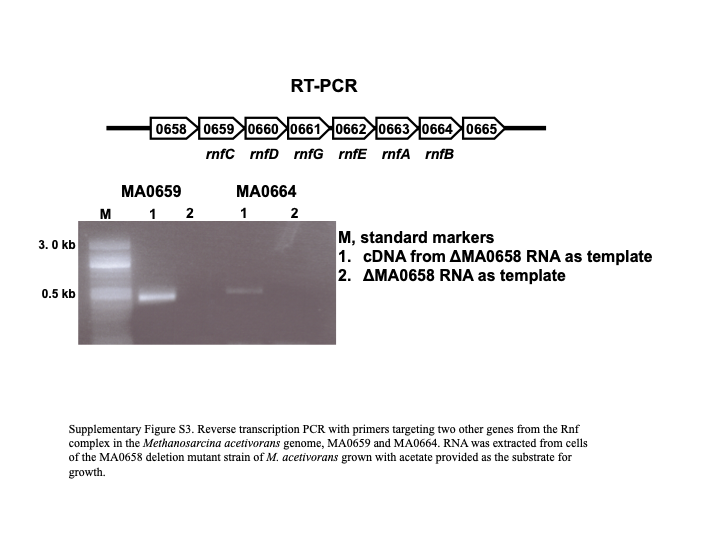

Supplement: FIG S3 [file mBio.00789-19-sf003.tif]

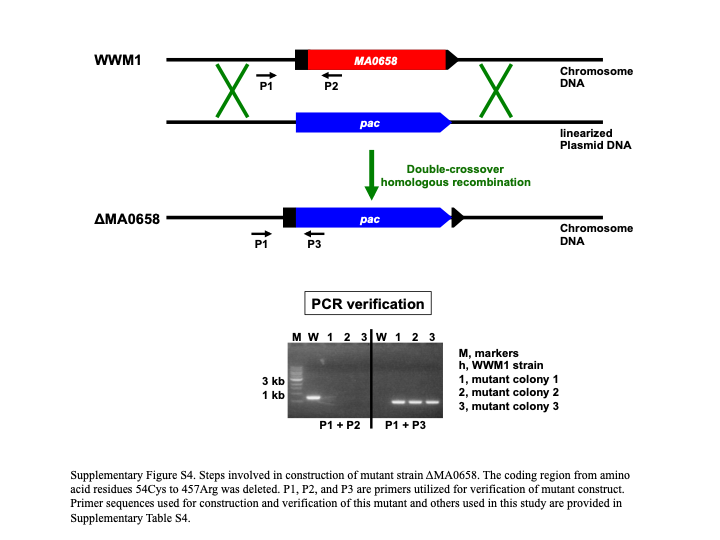

Supplement: FIG S4 [file mBio.00789-19-sf004.tif]

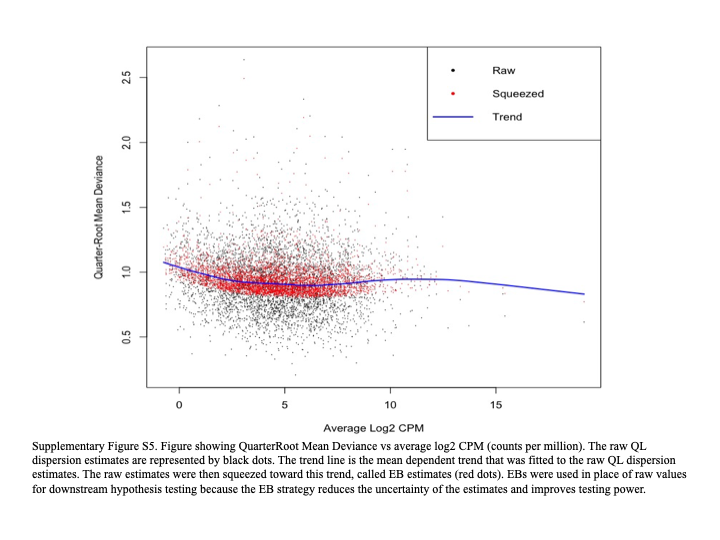

Supplement: FIG S5 [file mBio.00789-19-sf005.tif]
