# Supplementary material for: A Membrane-Bound Cytochrome Enables Methanosarcina acetivorans To Conserve Energy from Extracellular Electron Transfer
Source: mBio. 2019 Aug 20;10(4):e00789-19. doi: 10.1128/mBio.00789-19 (PMC6703419; doi:10.1128/mBio.00789-19)
Supplement: TABLE S2 [file mBio.00789-19-st002.docx]

**Supplementary Table S2**. Differential expression of genes coding for proteins from the oxidative branch of the methylotrophic methanogenesis pathway and methyl-coenzyme M reductase (Mcr) in *M. acetivorans* cells grown with methanol and AQDS in the presence of BES or cells grown via methanogenesis with methanol as the substrate. Negative values indicate that genes were more significantly expressed in methanogenic cells. Genes were only considered differentially expressed if the P-value and FDR (False Discovery Rate) were <0.05.

NS: no significant difference in read abundance between conditions

| Locus ID | Gene Annotation | Gene name | Fold up-regulated AQDS/BES vs methanogenesis | P-value | FDR |
| --- | --- | --- | --- | --- | --- |
| MA4546 | methyl-coenzyme M reductase, α-subunit | *mcrA* | NS | - | - |
| MA4547 | methyl-coenzyme M reductase, γ-subunit | *mcrG* | NS | - | - |
| MA4548 | methyl coenzyme M reductase, subunit D | *mcrD* | NS | - | - |
| MA4549 | methyl coenzyme M reductase, subunit C | *mcrC* | NS | - | - |
| MA4550 | methyl-coenzyme M reductase, β-subunit | *mcrB* | NS | - | - |
| MA0269 | H_4_MPT S-methyltransferase, subunit H | *mtrH* | -2.51 | 1.61x10^-5^ | 0.0001 |
| MA0270 | H_4_MPT S-methyltransferase, subunit G | *mtrG* | -4.08 | 3.42x10^-6^ | 5.21x10^-5^ |
| MA0271 | H_4_MPT S-methyltransferase, subunit F | *mtrF* | -3.32 | 1.38x10^-6^ | 2.84x10^-5^ |
| MA0272 | H_4_MPT S-methyltransferase, subunit A | *mtrA* | -2.90 | 9.51x10^-6^ | 0.0001 |
| MA0273 | H_4_MPT S-methyltransferase, subunit B | *mtrB* | -2.42 | 3.93x10^-5^ | 0.0002 |
| MA0274 | H_4_MPT S-methyltransferase, subunit C | *mtrC* | -2.57 | 2.78x10^-5^ | 0.0002 |
| MA0275 | H_4_MPT S-methyltransferase, subunit D | *mtrD* | -2.49 | 4.65x10^-5^ | 0.0003 |
| MA0276 | H_4_MPT S-methyltransferase, subunit E | *mtrE* | -2.98 | 5.69x10^-5^ | 0.0003 |
| MA3733 | methylenetetrahydromethanopterin reductase | *mer* | -2.32 | 6.40x10^-5^ | 0.0003 |
| MA4430 | methylenetetrahydromethanopterin dehydrogenase | *mtd* | -3.11 | 1.02x10^-6^ | 0.0001 |
| MA1710 | methenyltetrahydromethanopterin cyclohydrolase | *mch* | -1.77 | 0.001 | 0.003 |
| MA0010 | formylmethanofuran-tetrahydromethanopterin formyltransferase | *ftr* | NS | - | - |
| MA0304 | formylmethanofuran dehydrogenase, subunit E | *fmdE* | NS | - | - |
| MA0305 | formylmethanofuran dehydrogenase, subunit F | *fmdF* | NS | - | - |
| MA0306 | formylmethanofuran dehydrogenase, subunit A | *fmdA* | NS | - | - |
| MA0307 | formylmethanofuran dehydrogenase, subunit C | *fmdC* | -1.47 | 0.017 | 0.031 |
| MA0308 | formylmethanofuran dehydrogenase, subunit D | *fmdD* | -1.46 | 0.015 | 0.032 |
| MA0309 | formylmethanofuran dehydrogenase, subunit B | *fmdB* | NS | - | - |
| MA0975 | Coenzyme F_420_-reducing hydrogenase subunit alpha | *frhA* | 2.77 | 8.89x10^-6^ | 0.0001 |
| MA0976 | Coenzyme F_420_-reducing hydrogenase subunit delta | *frhD* | 2.67 | 5.38x10^-5^ | 0.0004 |
| MA0977 | Coenzyme F_420_-reducing hydrogenase subunit gamma | *frhG* | 2.35 | 4.89x10^-5^ | 0.0003 |
| MA0978 | Coenzyme F_420_-reducing hydrogenase subunit beta | *frhB* | 2.04 | 0.0004 | 0.001 |
| MA0687 | Heterodisulfide reductase subunit E | *hdrE* | -1.47 | 0.021 | 0.041 |
| MA0688 | Heterodisulfide reductase subunit D | *hdrD* | NS | - | - |
