# Supplementary material for: A Membrane-Bound Cytochrome Enables Methanosarcina acetivorans To Conserve Energy from Extracellular Electron Transfer
Source: mBio. 2019 Aug 20;10(4):e00789-19. doi: 10.1128/mBio.00789-19 (PMC6703419; doi:10.1128/mBio.00789-19)
Supplement: TABLE S3 [file mBio.00789-19-st003.docx]

**Supplementary Table S3**. Differential expression of genes coding for proteins from the proton-translocating F_420_:methanophenazine oxidoreductase (Fpo) complex in *M. acetivorans* cells grown with methanol as the electron donor and AQDS (in the presence of BES) as the acceptor vs cells grown via methylotrophic methanogenesis with methanol as the substrate. Negative values indicate that genes were more significantly expressed in methanogenic cells. Genes were only considered differentially expressed if the P-value and FDR (False Discovery Rate) were <0.05.

| Locus ID | Annotation | Gene | Fold up-regulated in AQDS/BES vs methanogenesis | P-value | FDR |
| --- | --- | --- | --- | --- | --- |
| MA1495 | F_420_H_2_ dehydrogenase subunit A | *fpoA* | -3.76 | 3.45x10^-6^ | 5.23x10^-5^ |
| MA1496 | F_420_H_2_ dehydrogenase subunit B | *fpoB* | -3.26 | 4.64x10^-6^ | 6.29x10^-5^ |
| MA1497 | F_420_H_2_ dehydrogenase subunit C | *fpoC* | -3.56 | 2.10x10^-6^ | 3.70x10^-5^ |
| MA1498 | F_420_H_2_ dehydrogenase subunit D | *fpoD* | -3.25 | 6.38x10^-6^ | 7.96x10^-5^ |
| MA1499 | F_420_H_2_ dehydrogenase subunit H | *fpoH* | -2.61 | 0.0001 | 0.0006 |
| MA1500 | F_420_H_2_ dehydrogenase subunit I | *fpoI* | -4.28 | 2.33x10^-6^ | 3.97x10^-5^ |
| MA1501 | F_420_H_2_ dehydrogenase subunit J | *fpoJ1* | -4.43 | 1.04x10^-5^ | 0.0001 |
| MA1502 | F_420_H_2_ dehydrogenase subunit J | *fpoJ2* | -4.20 | 8.98x10^-6^ | 0.0001 |
| MA1503 | F_420_H_2_ dehydrogenase subunit K | *fpoK* | -3.71 | 3.12x10^-5^ | 0.0002 |
| MA1504 | F_420_H_2_ dehydrogenase subunit L | *fpoL* | -3.62 | 3.60x10^-6^ | 5.35x10^-5^ |
| MA1505 | F_420_H_2_ dehydrogenase subunit M | *fpoM* | -2.99 | 3.66x10^-5^ | 0.0002 |
| MA1506 | F_420_H_2_ dehydrogenase subunit N | *fpoN* | -2.77 | 0.0003 | 0.001 |
| MA1507 | F_420_H_2_ dehydrogenase subunit 0 | *fpoO* | -2.76 | 0.0002 | 0.0009 |
| MA3732 | F_420_H_2_ dehydrogenase subunit F | *fpoF* | -2.77 | 1.25x10^-6^ | 0.0001 |
