# Supplementary material for: A Membrane-Bound Cytochrome Enables Methanosarcina acetivorans To Conserve Energy from Extracellular Electron Transfer
Source: mBio. 2019 Aug 20;10(4):e00789-19. doi: 10.1128/mBio.00789-19 (PMC6703419; doi:10.1128/mBio.00789-19)
Supplement: TABLE S5 [file mBio.00789-19-st005.docx]

Supplementary Table S5A. Summary of statistics from RNAseq libraries assembled from RNA extracted from *Methanosarcina* *acetivorans* cells grown via methylotrophic methanogenesis with methanol provided as the substrate (3 biological replicates).

|  | Mean | SEM | Max | Min | Units |
| --- | --- | --- | --- | --- | --- |
| Raw (unfiltered) reads | 142,105,839 | 15,909,653 | 169,739,812 | 114,627,819 | #reads/library |
| QC-filtered reads | 71,353,650 | 7,951,084 | 84,882,618 | 57,351,452 | #reads/library |
| QC-filtered merged paired end reads | 33,811,923 | 3,047,028 | 39,687,515 | 29,473,696 | #reads/library |
| QC-filtered merged paired mRNA reads | 26,502,161 | 2,768,286 | 31,708,914 | 22,268,595 | #mRNA reads/library |
| QC-filtered merged paired end read size | 154.37 | 1.05 | 190 | 100 | Bases/read |

Supplementary Table S5B. Summary of statistics from RNAseq libraries assembled from RNA extracted from *Methanosarcina* *acetivorans* cells grown with methanol provided as the electron donor and AQDS as the electron acceptor in the presence of BES (3 biological replicates).

|  | Mean | SEM | Max | Min | Units |
| --- | --- | --- | --- | --- | --- |
| Raw (unfiltered) reads | 126,269,117 | 5,436,726 | 137,002,905 | 119,397,788 | #reads/library |
| QC-filtered forward reads | 109,839,784 | 4,909,043 | 119,459,166 | 103,328,114 | #reads/library |
| QC-filtered merged paired end reads | 46,813,065 | 3,368,017 | 53,053,289 | 41,496,290 | #reads/library |
| QC-filtered merged paired mRNA reads | 34,856,940 | 2,774,940 | 40,072,214 | 30,605,661 | #mRNA reads/library |
| QC-filtered merged paired end read size | 154.37 | 1.05 | 190 | 100 | Bases/read |

Supplementary Table S5C. The glmQLFit() function was used to fit the negative binomial generalized log-linear model for each tag. Fitted values for each gene and biological replicate (3 AQDS/BES (AQB) and 3 Methanol (Met) samples) are provided in the table below. For differential expression analysis of these genes, the glmQLFTest() function was used which applies empirical Bayes quasi-likelihood (QL) F-tests to the fitted values.

| Locus ID | AQB1 | AQB2 | AQB3 | MET1 | MET2 | MET3 |
| --- | --- | --- | --- | --- | --- | --- |
| MA4660 | 268.2356328 | 331.0568484 | 399.6346386 | 2442.59489 | 4080.972737 | 2723.908174 |
| MA4659 | 182.3199262 | 225.0195455 | 271.631912 | 1313.25206 | 2194.119817 | 1464.499101 |
| MA4658 | 627.6000499 | 774.5849884 | 935.0387807 | 2893.968956 | 4835.107311 | 3227.266923 |
| MA4657 | 9.496174167 | 11.72019339 | 14.14800894 | 86.41641479 | 144.3804841 | 96.36898022 |
| MA4656 | 115.4633315 | 142.505029 | 172.024672 | 556.5308856 | 929.8256461 | 620.6264636 |
| MA4655 | 263.980791 | 325.8055158 | 393.2955025 | 1976.81285 | 3302.765997 | 2204.482087 |
| MA4654 | 832.5267758 | 1027.505882 | 1240.351752 | 5106.526202 | 8531.743966 | 5694.644053 |
| MA4653 | 1228.614984 | 1516.358584 | 1830.469352 | 1375.794793 | 2298.613276 | 1534.244871 |
| MA4652 | 2025.35002 | 2499.690245 | 3017.496277 | 8682.386065 | 14506.12256 | 9682.335156 |
| MA4651 | 36764.45355 | 45374.74755 | 54774.03934 | 37933.94994 | 63378.26061 | 42302.79722 |
| MA4650 | 26079.25281 | 32187.05566 | 38854.5424 | 35683.50823 | 59618.32839 | 39793.17248 |
| MA4649 | 10425.79678 | 12867.53512 | 15533.0203 | 18591.35649 | 31061.5646 | 20732.52021 |
| MA4648 | 4074.046799 | 5028.195096 | 6069.77605 | 4159.428305 | 6949.377313 | 4638.469034 |
| MA4647 | 5471.907915 | 6753.437527 | 8152.399137 | 5190.899979 | 8672.711706 | 5788.735145 |
| MA4645 | 2361.349347 | 2914.381152 | 3518.089609 | 11868.94251 | 19830.07129 | 13235.88682 |
| MA4644 | 217.3534845 | 268.2580193 | 323.8271527 | 799.4545395 | 1335.691069 | 891.527598 |
| MA4643 | 1599.354204 | 1973.925524 | 2382.820404 | 4637.742358 | 7748.521953 | 5171.870445 |
| MA4642 | 18.58199759 | 22.93393124 | 27.68465103 | 62.39839621 | 104.2523076 | 69.58481007 |
| MA4641 | 13.88854752 | 17.14126763 | 20.69204829 | 52.49013675 | 87.69805338 | 58.53541787 |
| MA4640 | 241.5482189 | 298.1191993 | 359.8740187 | 491.1786585 | 820.6382166 | 547.7476303 |
| MA4639 | 14.32490814 | 17.6798246 | 21.34216631 | 43.71393661 | 73.03519068 | 48.74846408 |
| MA4638 | 2270.802851 | 2802.628522 | 3383.187636 | 4530.878112 | 7569.978192 | 5052.698659 |
| MA4637 | 657.1606686 | 811.0687515 | 979.0800853 | 1752.998039 | 2928.826731 | 1954.890558 |
| MA4636 | 1636.69972 | 2020.017421 | 2438.460149 | 2007.845464 | 3354.61383 | 2239.088723 |
| MA4635 | 4067.094815 | 5019.614946 | 6059.418539 | 19647.52783 | 32826.16604 | 21910.33064 |
| MA4634 | 25329.5947 | 31261.82641 | 37737.6537 | 3823.001621 | 6387.291422 | 4263.29614 |
| MA4633 | 2727.267575 | 3365.998016 | 4063.258039 | 3384.685682 | 5654.973752 | 3774.49942 |
| MA4632 | 846.4139664 | 1044.645475 | 1261.041779 | 5614.676407 | 9380.737443 | 6261.31784 |
| MA4631 | 796.2309498 | 982.7095157 | 1186.275904 | 4649.474771 | 7768.123916 | 5184.954078 |
| MA4630 | 444.6842724 | 548.8300424 | 662.5191314 | 2458.8288 | 4108.095591 | 2742.011741 |
| MA4629 | 275.9219384 | 340.543299 | 411.0861892 | 4700.802448 | 7853.879786 | 5242.193155 |
| MA4628 | 288.9001933 | 356.5610821 | 430.4220252 | 839.5662126 | 1402.707767 | 936.2589264 |
| MA4627 | 185.0561128 | 228.3965513 | 275.7084582 | 660.6040228 | 1103.706152 | 736.6856882 |
| MA4626 | 118.5107394 | 146.2661447 | 176.5648956 | 518.5774241 | 866.4147864 | 578.3019077 |
| MA4625 | 17.57009905 | 21.68504444 | 26.17705974 | 126.9570641 | 212.1138955 | 141.5786899 |
| MA4624 | 231.0706099 | 285.1877175 | 344.2638053 | 456.0166809 | 761.8912372 | 508.5360532 |
| MA4623 | 763.6644564 | 942.5158973 | 1137.756255 | 2101.996857 | 3511.917551 | 2344.083517 |
| MA4622 | 34.18662655 | 42.19318934 | 50.93342745 | 151.6546249 | 253.3774192 | 169.1206651 |
| MA4621 | 212.2238113 | 261.9269684 | 316.1846367 | 820.630103 | 1371.070205 | 915.1419479 |
| MA4620 | 117.8123071 | 145.4041384 | 175.5243263 | 241.4807926 | 403.4547584 | 269.292099 |
| MA4619 | 1213.990759 | 1498.309343 | 1808.681245 | 3621.657822 | 6050.895667 | 4038.763606 |
| MA4618 | 831.4406558 | 1026.165391 | 1238.733581 | 3279.892111 | 5479.88958 | 3657.63679 |
| MA4617 | 570.9638165 | 704.6844584 | 850.6584902 | 2994.42559 | 5002.945534 | 3339.293133 |
| MA4616 | 647.8033559 | 799.5199412 | 965.138961 | 2638.304553 | 4407.955244 | 2942.15769 |
| MA4615 | 11696.9987 | 14436.45456 | 17426.93839 | 95241.6085 | 159125.203 | 106210.5702 |
| MA4611 | 947.9220565 | 1169.926923 | 1412.275037 | 2291.574195 | 3828.654459 | 2555.494449 |
| MA4610 | 1214.433787 | 1498.856128 | 1809.341296 | 1061.174617 | 1772.960674 | 1183.389938 |
| MA4609 | 200.727381 | 247.7380557 | 299.056518 | 920.527565 | 1537.97419 | 1026.544586 |
| MA4608 | 1244.404082 | 1535.845514 | 1853.992962 | 14715.1386 | 24585.36194 | 16409.87889 |
| MA4607 | 157.9434377 | 194.9340443 | 235.314257 | 2130.928836 | 3560.255743 | 2376.347588 |
| MA4606 | 43.78818085 | 54.04344306 | 65.23843847 | 253.4322981 | 423.422772 | 282.6200576 |
| MA4605 | 123.9927243 | 153.0320193 | 184.7323081 | 227.636006 | 380.3235396 | 253.8528104 |
| MA4604 | 484.4739483 | 597.9385243 | 721.8003409 | 2397.071934 | 4004.915122 | 2673.142346 |
| MA4603 | 10.40837803 | 12.84603686 | 15.50706872 | 69.36745031 | 115.8958756 | 77.35648908 |
| MA4602 | 818.9649462 | 1010.767851 | 1220.146469 | 12031.1073 | 20101.0086 | 13416.72811 |
| MA4601 | 1334.193435 | 1646.663678 | 1987.766896 | 12642.18063 | 21121.96119 | 14098.17866 |
| MA4600 | 182.858974 | 225.6848391 | 272.4350199 | 1426.646242 | 2383.573486 | 1590.952874 |
| MA4599 | 99.24519523 | 122.4885792 | 147.861853 | 608.3441174 | 1016.392758 | 678.4070175 |
| MA4598 | 150.6392862 | 185.9192487 | 224.4320639 | 1632.124612 | 2726.87709 | 1820.096157 |
| MA4597 | 72.29984656 | 89.23258662 | 107.7169455 | 617.7375229 | 1032.086819 | 688.8822601 |
| MA4596 | 2637.293337 | 3254.951667 | 3929.208652 | 3941.623025 | 6585.478488 | 4395.579153 |
| MA4595 | 535.2491336 | 660.6053395 | 797.4484664 | 2221.957924 | 3712.342864 | 2477.860482 |
| MA4594 | 685.6650309 | 846.2488812 | 1021.547711 | 2339.438829 | 3908.624438 | 2608.871645 |
| MA4593 | 1663.667333 | 2053.300892 | 2478.638227 | 3730.064363 | 6232.016219 | 4159.655311 |
| MA4592 | 5901.7784 | 7283.98437 | 8792.847739 | 13021.17758 | 21755.17148 | 14520.82463 |
| MA4591 | 4713.316091 | 5817.182282 | 7022.200416 | 8206.323326 | 13710.73931 | 9151.444343 |
| MA4590 | 537.412915 | 663.2758819 | 800.6722066 | 4256.53455 | 7111.617863 | 4746.758991 |
| MA4589 | 706.8885263 | 872.4429533 | 1053.167835 | 2347.056073 | 3921.350973 | 2617.366167 |
| MA4588 | 662.4222209 | 817.5625677 | 986.919083 | 2054.108889 | 3431.908586 | 2290.680299 |
| MA4587 | 2366.345023 | 2920.546824 | 3525.532488 | 9754.6264 | 16297.57131 | 10878.06525 |
| MA4586 | 387.0096807 | 477.6479688 | 576.591828 | 874.1014104 | 1460.407553 | 974.7715377 |
| MA4585 | 991.8400196 | 1224.130543 | 1477.706834 | 2581.409825 | 4312.898206 | 2878.710405 |
| MA4583 | 31.00738606 | 38.26936562 | 46.19679119 | 136.6401951 | 228.2920156 | 152.3770257 |
| MA4582 | 189.940958 | 234.4254351 | 282.9862136 | 535.8870525 | 895.3349 | 597.6050834 |
| MA4581 | 359.8174966 | 444.0873316 | 536.0791692 | 1123.130902 | 1876.474322 | 1252.481718 |
| MA4580 | 1465.815484 | 1809.111821 | 2183.86586 | 3220.160315 | 5380.092503 | 3591.025692 |
| MA4579 | 221.4857099 | 273.3580185 | 329.9836069 | 459.772307 | 768.1659606 | 512.7242141 |
| MA4578 | 260.52576 | 321.541311 | 388.1479759 | 510.7570381 | 853.348853 | 569.580849 |
| MA4577 | 1227.020679 | 1514.39089 | 1828.094054 | 5271.380998 | 8807.17561 | 5878.485151 |
| MA4576 | 9841.158923 | 12145.97413 | 14661.98934 | 52017.26154 | 86907.99571 | 58008.08169 |
| MA4575 | 26377.2401 | 32554.83205 | 39298.50297 | 151477.3373 | 253081.2155 | 168922.9593 |
| MA4574 | 7235.150068 | 8929.633822 | 10779.39031 | 68773.53631 | 114903.592 | 76694.1741 |
| MA4573 | 10738.44666 | 13253.40809 | 15998.82612 | 53884.10502 | 90027.03005 | 60089.92925 |
| MA4572 | 4753.984061 | 5867.374756 | 7082.790165 | 3545.625072 | 5923.863721 | 3953.974175 |
| MA4665 | 2970.343372 | 3666.002555 | 4425.407942 | 2228.507515 | 3723.285613 | 2485.164388 |
| MA4570 | 616.9239252 | 761.4084982 | 919.1328059 | 385.0180412 | 643.2700469 | 429.3605108 |
| MA4569 | 4796.231343 | 5919.516419 | 7145.732873 | 4021.729904 | 6719.317297 | 4484.911929 |
| MA4568 | 2130.712645 | 2629.728965 | 3174.472268 | 2333.378021 | 3898.498326 | 2602.112815 |
| MA4567 | 400.7112592 | 494.558479 | 597.0053178 | 339.1342045 | 566.6094891 | 378.1922395 |
| MA4566 | 976.9802903 | 1205.790642 | 1455.567857 | 867.4787959 | 1449.3428 | 967.3861977 |
| MA4565 | 50.87464102 | 62.78956357 | 75.79630104 | 146.2510459 | 244.3493734 | 163.0947567 |
| MA4564 | 82.11408875 | 101.3453401 | 122.3388326 | 270.1928453 | 451.4255065 | 301.3109144 |
| MA4563 | 154.5277906 | 190.7184471 | 230.225407 | 413.4932515 | 690.8450899 | 461.1152068 |
| MA4562 | 463.3110651 | 571.8192599 | 690.2705211 | 616.2747653 | 1029.642912 | 687.2510368 |
| MA4561 | 574.3940575 | 708.9180673 | 855.769083 | 2086.089971 | 3485.341075 | 2326.344638 |
| MA4560 | 138.6533852 | 171.1262305 | 206.5747004 | 645.2546834 | 1078.061197 | 719.5685678 |
| MA4559 | 877.3875212 | 1082.873086 | 1307.188166 | 1110.765059 | 1855.814054 | 1238.691703 |
| MA4558 | 329.8948464 | 407.1567487 | 491.4984871 | 447.4888488 | 747.6433361 | 499.0260719 |
| MA4557 | 53.57801246 | 66.12606894 | 79.82395709 | 64.12070887 | 107.1298666 | 71.50548121 |
| MA4556 | 365.5738351 | 451.1918138 | 544.6553313 | 585.5174751 | 978.2550773 | 652.9514342 |
| MA4555 | 1287.201134 | 1588.665705 | 1917.754754 | 3672.310868 | 6135.524395 | 4095.250356 |
| MA4554 | 569.4388167 | 702.8023012 | 848.3864477 | 2548.725623 | 4258.290978 | 2842.261968 |
| MA4553 | 251.0694327 | 309.8702967 | 374.0593334 | 1139.027883 | 1903.034252 | 1270.209552 |
| MA4552 | 1697.709378 | 2095.315638 | 2529.356247 | 8445.244349 | 14109.91732 | 9417.881864 |
| MA4551 | 768.10738 | 947.9993607 | 1144.375607 | 7406.583623 | 12374.5718 | 8259.598739 |
| MA4550 | 135162.1215 | 166817.3072 | 201373.1919 | 500067.4559 | 835489.2016 | 557660.1493 |
| MA4549 | 30073.03107 | 37116.18319 | 44804.72924 | 161238.6045 | 269389.882 | 179808.4302 |
| MA4548 | 46852.0178 | 57824.83553 | 69803.13913 | 285244.0919 | 476572.4219 | 318095.6108 |
| MA4547 | 48534.75761 | 59901.6757 | 72310.19275 | 255876.4511 | 427506.348 | 285345.7033 |
| MA4546 | 38652.19862 | 47704.60555 | 57586.52295 | 163791.995 | 273655.9668 | 182655.8943 |
| MA4545 | 561.0072263 | 692.3960187 | 835.8245239 | 1281.405484 | 2140.912054 | 1428.98476 |
| MA4544 | 1003.698351 | 1238.766115 | 1495.374136 | 2952.469441 | 4932.847173 | 3292.504901 |
| MA4543 | 1214.970174 | 1499.518137 | 1810.140439 | 3322.369322 | 5550.858508 | 3705.006095 |
| MA4542 | 9511.183774 | 11738.71827 | 14170.37121 | 19571.3946 | 32698.96623 | 21825.42916 |
| MA4541 | 287.4216646 | 354.7362796 | 428.2192184 | 946.8838647 | 1582.009056 | 1055.936336 |
| MA4540 | 72.78520471 | 89.83161642 | 108.4400632 | 416.7687918 | 696.3177087 | 464.7679906 |
| MA4539 | 17.66272427 | 21.79936263 | 26.31505872 | 87.91360394 | 146.8819174 | 98.03860042 |
| MA4538 | 188.9200225 | 233.1653948 | 281.4651584 | 351.314544 | 586.959828 | 391.775387 |
| MA4537 | 184.063473 | 227.1714336 | 274.2295597 | 620.1182408 | 1036.06441 | 691.5371648 |
| MA4536 | 144.4623351 | 178.2956458 | 215.2292463 | 1149.134276 | 1919.919538 | 1281.479897 |
| MA4535 | 60.78848537 | 75.02524617 | 90.56658179 | 281.5357441 | 470.3766887 | 313.9601732 |
| MA4534 | 167.0829229 | 206.2140115 | 248.9308478 | 733.4228598 | 1225.368442 | 817.8910597 |
| MA4533 | 83.25370461 | 102.7518558 | 124.0367053 | 264.4760836 | 441.8742098 | 294.9357542 |
| MA4532 | 38.63929441 | 47.68867916 | 57.56729743 | 106.9891548 | 178.7524512 | 119.3110796 |
| MA4531 | 73.38943271 | 90.57735559 | 109.3402808 | 333.2371342 | 556.7569412 | 371.6160045 |
| MA4528 | 70.77879975 | 87.3553082 | 105.4507925 | 629.0078364 | 1050.916729 | 701.4505738 |
| MA4527 | 589.7681536 | 727.8928014 | 878.6743969 | 989.3914983 | 1653.028813 | 1103.339568 |
| MA4526 | 74.24100792 | 91.62837108 | 110.6090121 | 110.8936966 | 185.2759761 | 123.6653068 |
| MA4525 | 208.9301984 | 257.8619861 | 311.2776012 | 1367.136002 | 2284.146575 | 1524.588848 |
| MA4524 | 2157.83124 | 2663.198778 | 3214.875288 | 11526.21499 | 19257.45825 | 12853.68742 |
| MA4523 | 1408.66728 | 1738.579417 | 2098.722804 | 7849.83299 | 13115.13201 | 8753.897069 |
| MA4522 | 495.9191888 | 612.0642584 | 738.8521938 | 3410.124719 | 5697.4761 | 3802.868268 |
| MA4521 | 30.21273707 | 37.28860857 | 45.0128722 | 114.9673486 | 192.0820424 | 128.2081208 |
| MA4520 | 1004.29862 | 1239.506968 | 1496.268456 | 5668.806313 | 9471.175147 | 6321.681879 |
| MA4519 | 1633.783398 | 2016.418091 | 2434.115225 | 10426.61626 | 17420.30039 | 11627.44808 |
| MA4518 | 1567.763594 | 1934.936343 | 2335.754689 | 7040.037512 | 11762.16378 | 7850.837568 |
| MA4517 | 1536.571582 | 1896.439111 | 2289.28283 | 22625.73138 | 37802.00856 | 25231.53345 |
| MA4516 | 3406.380779 | 4204.160623 | 5075.044419 | 12246.734 | 20461.26755 | 13657.1885 |
| MA4515 | 714.674223 | 882.0520726 | 1064.767465 | 3762.913313 | 6286.898703 | 4196.287471 |
| MA4514 | 192.0379393 | 237.0135328 | 286.1104308 | 1171.291511 | 1956.938805 | 1306.188978 |
| MA4513 | 763.854841 | 942.7508703 | 1138.039902 | 5410.201647 | 9039.110625 | 6033.293753 |
| MA4512 | 790.0997949 | 975.1424345 | 1177.141316 | 9560.453338 | 15973.15608 | 10661.5293 |
| MA4511 | 706.6871396 | 872.1944015 | 1052.867796 | 6807.578204 | 11373.7817 | 7591.605956 |
| MA4510 | 50.33820055 | 62.12748788 | 74.99707764 | 437.5879126 | 731.1013171 | 487.9848463 |
| MA4509 | 24.20602099 | 29.87510994 | 36.06368157 | 234.1015546 | 391.1258744 | 261.0629953 |
| MA4508 | 86.2743712 | 106.4799673 | 128.537088 | 157.2401122 | 262.709389 | 175.3494322 |
| MA4507 | 3762.44724 | 4643.61842 | 5605.535055 | 14734.4969 | 24617.70488 | 16431.46668 |
| MA4506 | 238.2143414 | 294.0045223 | 354.9069943 | 458.7940412 | 766.5315201 | 511.6332815 |
| MA4505 | 87.64058767 | 108.1661539 | 130.5725649 | 347.0324874 | 579.805569 | 387.0001665 |
| MA4504 | 1819.985587 | 2246.229131 | 2711.531181 | 7826.753145 | 13076.57129 | 8728.159121 |
| MA4503 | 654.9026361 | 808.2818842 | 975.7159237 | 1521.611052 | 2542.236229 | 1696.854767 |
| MA4502 | 1736.724238 | 2143.467841 | 2587.483086 | 5390.739649 | 9006.594435 | 6011.59032 |
| MA4501 | 583.8247994 | 720.5575041 | 869.8196069 | 2708.468825 | 4525.182411 | 3020.40277 |
| MA4500 | 551.6855358 | 680.8911734 | 821.936472 | 3161.189929 | 5281.567553 | 3525.263695 |
| MA4499 | 97.51869838 | 120.357734 | 145.2896074 | 418.0280882 | 698.4216818 | 466.1723199 |
| MA4498 | 84.06943447 | 103.7586309 | 125.252032 | 271.0053808 | 452.7830526 | 302.2170295 |
| MA4497 | 77.87962054 | 96.11915262 | 116.0300504 | 160.5680054 | 268.2694766 | 179.0605984 |
| MA4496 | 82.81794289 | 102.214038 | 123.3874796 | 165.1549166 | 275.9330722 | 184.1757834 |
| MA4494 | 74.67245892 | 92.1608686 | 111.2518155 | 131.0567897 | 218.9635244 | 146.1505804 |
| MA4493 | 49.77227138 | 61.42901719 | 74.15392009 | 89.10268708 | 148.8685816 | 99.36463008 |
| MA4492 | 58.7967381 | 72.5670285 | 87.59914904 | 141.1162772 | 235.7704433 | 157.3686175 |
| MA4491 | 59.22342328 | 73.09364403 | 88.23485197 | 142.9441065 | 238.8242946 | 159.4069577 |
| MA4490 | 73.87008521 | 91.17057768 | 110.0563877 | 205.043944 | 342.5777844 | 228.6588241 |
| MA4489 | 52.42500354 | 64.70302348 | 78.10613049 | 127.0245666 | 212.2266756 | 141.6539668 |
| MA4488 | 118.4928621 | 146.2440805 | 176.5382608 | 296.6742237 | 495.6693489 | 330.8421492 |
| MA4487 | 162.8190393 | 200.9515195 | 242.5782407 | 325.7032586 | 544.1696961 | 363.2144537 |
| MA4486 | 708.6399893 | 874.6046117 | 1055.777277 | 1022.670829 | 1708.630355 | 1140.451674 |
| MA4485 | 966.5312975 | 1192.894478 | 1440.000278 | 1373.987982 | 2295.594542 | 1532.229971 |
| MA4484 | 220.848683 | 272.5717989 | 329.0345234 | 416.2309268 | 695.419069 | 464.1681797 |
| MA4483 | 877.24617 | 1082.69863 | 1306.977572 | 1276.31382 | 2132.405142 | 1423.306689 |
| MA4482 | 9.74533681 | 12.02771033 | 14.51922741 | 32.02161703 | 53.50021268 | 35.70954182 |
| MA4481 | 355.0180731 | 438.1638754 | 528.9286805 | 1923.026314 | 3212.90198 | 2144.500964 |
| MA4480 | 141.8853919 | 175.1151784 | 211.3899512 | 1070.828902 | 1789.090599 | 1194.156104 |
| MA4479 | 7.452119825 | 9.197418243 | 11.10264576 | 95.4642977 | 159.4972617 | 106.4589064 |
| MA4478 | 333.3542252 | 411.4263196 | 496.6524913 | 685.4510809 | 1145.219449 | 764.3943783 |
| MA4477 | 31.86646747 | 39.32964528 | 47.47670574 | 119.5597722 | 199.7548479 | 133.3294531 |
| MA4476 | 49.41930719 | 60.99338822 | 73.62805142 | 309.58612 | 517.2419384 | 345.2411066 |
| MA4475 | 23.08198659 | 28.48782488 | 34.38902308 | 133.4829931 | 223.017111 | 148.8562093 |
| MA4474 | 1504.225992 | 1856.518132 | 2241.092297 | 19083.34009 | 31883.54768 | 21281.16548 |
| MA4473 | 3636.823237 | 4488.573074 | 5418.372364 | 33352.75675 | 55724.21835 | 37193.9887 |
| MA4472 | 195.6798999 | 241.5084464 | 291.5364571 | 3375.011517 | 5638.810612 | 3763.711083 |
| MA4470 | 358.910028 | 442.9673324 | 534.7271644 | 6152.017114 | 10278.50104 | 6860.543995 |
| MA4469 | 149.9311289 | 185.0452398 | 223.3770057 | 1102.827941 | 1842.55309 | 1229.840469 |
| MA4467n | 50.43686997 | 62.24926584 | 75.14408167 | 233.3408856 | 389.8549843 | 260.2147201 |
| MA4466 | 224.8458693 | 277.5051326 | 334.9897877 | 699.8802666 | 1169.327054 | 780.4853712 |
| MA4465 | 295.6748081 | 364.9223225 | 440.5152806 | 652.8226774 | 1090.705446 | 728.0081665 |
| MA4464 | 160.30977 | 197.8545753 | 238.8397705 | 1741.686189 | 2909.927423 | 1942.275924 |
| MA4462 | 11.48557036 | 14.17550936 | 17.11193889 | 294.5450108 | 492.1119601 | 328.4677152 |
| MA4461a | 45.30567379 | 55.91633528 | 67.49929671 | 441.5164485 | 737.6649303 | 492.3658311 |
| MA4461 | 724.5715822 | 894.2674093 | 1079.513185 | 3871.800877 | 6468.822925 | 4317.715599 |
| MA4460 | 630.3985312 | 778.0388786 | 939.2081376 | 9426.409221 | 15749.20147 | 10512.04735 |
| MA4459 | 1850.726447 | 2284.16955 | 2757.330884 | 6547.424559 | 10939.12922 | 7301.490455 |
| MA4458 | 16.31820672 | 20.13995691 | 24.3119103 | 75.45329159 | 126.0638132 | 84.14323577 |
| MA4457 | 1561.724474 | 1927.482852 | 2326.757222 | 9207.397253 | 15383.28657 | 10267.8118 |
| MA4456 | 382.3238036 | 471.8646518 | 569.6105079 | 1368.131494 | 2285.809797 | 1525.698992 |
| MA4455 | 74.29675529 | 91.69717458 | 110.6920681 | 197.0263105 | 329.1822991 | 219.7178009 |
| MA4454 | 159.9309292 | 197.3870094 | 238.2753491 | 565.2236779 | 944.3491548 | 630.3204034 |
| MA4453 | 443.7354854 | 547.659048 | 661.1055677 | 702.7706644 | 1174.156195 | 783.7086557 |
| MA4452 | 149.4917441 | 184.5029504 | 222.7223822 | 541.7247804 | 905.0883014 | 604.1151415 |
| MA4451 | 10.2914266 | 12.70169521 | 15.33282698 | 181.6059036 | 303.4186081 | 202.5214281 |
| MA4450 | 193.996724 | 239.431068 | 289.0287538 | 4175.016912 | 6975.422025 | 4655.852979 |
| MA4449 | 268.8642439 | 331.8326812 | 400.5711836 | 2074.036067 | 3465.201978 | 2312.902487 |
| MA4448 | 403.7863067 | 498.3537076 | 601.5867207 | 1018.069849 | 1700.943253 | 1135.3208 |
| MA4447 | 79.54411493 | 98.17347426 | 118.5099209 | 215.9344273 | 360.7730919 | 240.8035627 |
| MA4446 | 1682.241497 | 2076.225155 | 2506.311207 | 1221.086835 | 2040.134492 | 1361.719222 |
| MA4445 | 72.97365474 | 90.06420176 | 108.7208281 | 240.9654834 | 402.5938039 | 268.7174417 |
| MA4444 | 5255.030134 | 6485.766622 | 7829.280718 | 12541.45243 | 20953.66925 | 13985.84961 |
| MA4443 | 35.66807968 | 44.02160116 | 53.14059127 | 214.4870572 | 358.354894 | 239.1894992 |
| MA4442 | 5578.653518 | 6885.183123 | 8311.435579 | 20488.80593 | 34231.73395 | 22848.49861 |
| MA4441 | 49.6327385 | 61.25680549 | 73.94603506 | 450.4467812 | 752.5853104 | 502.3246689 |
| MA4440 | 308.1552966 | 380.3257615 | 459.1095123 | 390.1560423 | 651.8543777 | 435.0902547 |
| MA4439 | 104.9886511 | 129.5771616 | 156.4188217 | 801.1076715 | 1338.453044 | 893.3711209 |
| MA4438 | 422.7968962 | 521.8166076 | 629.9099155 | 3052.585458 | 5100.116307 | 3404.151263 |
| MA4437 | 92.44796361 | 114.099425 | 137.7349017 | 1729.622679 | 2889.772277 | 1928.823061 |
| MA4436 | 1760.740097 | 2173.108253 | 2623.263453 | 14071.79466 | 23510.49313 | 15692.44112 |
| MA4435 | 426.2513389 | 526.0800863 | 635.0565656 | 1759.195906 | 2939.181835 | 1961.802232 |
| MA4434 | 293.5546958 | 362.3056765 | 437.3566014 | 1114.536348 | 1862.114946 | 1242.897331 |
| MA4433 | 136.6573855 | 168.6627645 | 203.6009321 | 553.0217673 | 923.962776 | 616.7132006 |
| MA4432 | 507.3661912 | 626.1921671 | 755.9066716 | 1323.727143 | 2211.621093 | 1476.180598 |
| MA4431 | 1790.257982 | 2209.539274 | 2667.24109 | 1279.418359 | 2137.592061 | 1426.768779 |
| MA4430 | 6764.549649 | 8348.81803 | 10078.25965 | 85377.26062 | 142644.3142 | 95210.14682 |
| MA4429 | 1666.992282 | 2057.404549 | 2483.591948 | 29238.72617 | 48850.68942 | 32606.14584 |
| MA4428 | 2462.782793 | 3039.570475 | 3669.211659 | 44254.8837 | 73938.97965 | 49351.71196 |
| MA4427 | 19.35110411 | 23.88316374 | 28.83051523 | 344.4963919 | 575.5683798 | 384.1719893 |
| MA4426 | 52.78128397 | 65.14274536 | 78.63693992 | 1105.553328 | 1847.106538 | 1232.879738 |
| MA4425 | 17.17493782 | 21.19733581 | 25.5883232 | 225.0417466 | 375.989173 | 250.9597705 |
| MA4424 | 5531.634683 | 6827.152401 | 8241.383905 | 8737.195839 | 14597.69615 | 9743.457363 |
| MA4423 | 1288.211159 | 1589.912279 | 1919.259553 | 3281.416133 | 5482.435845 | 3659.336333 |
| MA4422 | 248.0369219 | 306.127567 | 369.5413044 | 1505.379948 | 2515.118062 | 1678.754329 |
| MA4421 | 837.0144883 | 1033.044624 | 1247.037834 | 5135.386659 | 8579.962661 | 5726.828365 |
| MA4420 | 804.298364 | 992.6663312 | 1198.295255 | 3584.768927 | 5989.263435 | 3997.626223 |
| MA4419 | 1548.891521 | 1911.6444 | 2307.637865 | 10149.00251 | 16956.47637 | 11317.86159 |
| MA4418 | 527.4728484 | 651.0078358 | 785.8628583 | 761.1442897 | 1271.684105 | 848.8051626 |
| MA4417 | 253.2308999 | 312.537983 | 377.2796257 | 1094.666436 | 1828.91723 | 1220.739004 |
| MA4416 | 900.4186148 | 1111.298099 | 1341.501365 | 3900.545656 | 6516.848351 | 4349.770909 |
| MA4415 | 1637.433254 | 2020.92275 | 2439.553015 | 5010.927298 | 8372.02181 | 5588.035038 |
| MA4414 | 312.2893022 | 385.4279578 | 465.2686189 | 1258.667748 | 2102.922915 | 1403.628322 |
| MA4413 | 3259.18037 | 4022.485641 | 4855.735814 | 104096.1157 | 173918.8974 | 116084.8496 |
| MA4412 | 8.261089525 | 10.19584995 | 12.30790067 | 53.6058089 | 89.56206597 | 59.77958181 |
| MA4411 | 1570.039755 | 1937.745584 | 2339.145859 | 7016.42546 | 11722.71388 | 7824.506119 |
| MA4410 | 1798.745394 | 2220.014451 | 2679.88618 | 9353.60129 | 15627.5574 | 10430.85414 |
| MA4409 | 250.0376267 | 308.59684 | 372.5220826 | 801.6398162 | 1339.342127 | 893.9645527 |
| MA4408 | 348.460573 | 430.0705983 | 519.1588965 | 969.5148365 | 1619.81982 | 1081.173714 |
| MA4407 | 6439.370242 | 7947.481084 | 9593.786522 | 11356.68354 | 18974.21308 | 12664.63107 |
| MA4406 | 3910.050547 | 4825.790658 | 5825.443922 | 8282.916323 | 13838.70728 | 9236.858544 |
| MA4405 | 375.8060148 | 463.8203864 | 559.8998883 | 4154.654095 | 6941.400788 | 4633.144979 |
| MA4404 | 1817.173147 | 2242.758013 | 2707.341027 | 8793.720894 | 14692.13555 | 9806.4924 |
| MA4403 | 66.82117133 | 82.47079686 | 99.554464 | 603.718248 | 1008.664073 | 673.2483874 |
| MA4402 | 426.3017605 | 526.1423168 | 635.1316869 | 3305.429365 | 5522.556024 | 3686.115166 |
| MA4401 | 907.5886817 | 1120.147407 | 1352.183791 | 5740.396662 | 9590.784937 | 6401.517278 |
| MA4400 | 266.3217767 | 328.6947641 | 396.783253 | 1195.832193 | 1997.940223 | 1333.556006 |
| MA4399 | 6369.186846 | 7860.860624 | 9489.222801 | 16284.2229 | 27206.91424 | 18159.67439 |
| MA4398 | 984.3787996 | 1214.921894 | 1466.59063 | 5721.687158 | 9559.525977 | 6380.653003 |
| MA4397 | 2357.780846 | 2909.976903 | 3512.773028 | 7908.767798 | 13213.59752 | 8819.61939 |
| MA4396 | 10756.98346 | 13276.28625 | 16026.44344 | 19209.1774 | 32093.79075 | 21421.49546 |
| MA4395 | 12385.75702 | 15286.52118 | 18453.09468 | 7752.121881 | 12951.88088 | 8644.932586 |
| MA4394 | 6310.221956 | 7788.086062 | 9401.373129 | 806.661646 | 1347.732364 | 899.5647459 |
| MA4393 | 115825.5662 | 142952.0996 | 172564.3525 | 9620.649325 | 16073.72871 | 10728.65805 |
| MA4392 | 1102999.022 | 1361323.162 | 1643318.64 | 65284.64666 | 109074.5192 | 72803.46955 |
| MA4391 | 1026186.717 | 1266521.293 | 1528878.745 | 86242.26336 | 144089.5201 | 96174.7718 |
| MA4390 | 159406.7884 | 196740.114 | 237494.4507 | 53825.90989 | 89929.80036 | 60025.0318 |
| MA4389 | 526.32087 | 649.5860622 | 784.146567 | 1179.459179 | 1970.58496 | 1315.297314 |
| MA4388 | 119.551654 | 147.5508432 | 178.1157169 | 377.851933 | 631.2972502 | 421.369083 |
| MA4387 | 159.8190733 | 197.2489566 | 238.1086991 | 312.6848751 | 522.4191927 | 348.6967449 |
| MA4386 | 2502.78962 | 3088.946964 | 3728.816393 | 4361.839858 | 7287.557022 | 4864.192295 |
| MA4385 | 185.3866414 | 228.8044902 | 276.2009008 | 168.3949491 | 281.3463661 | 187.7889699 |
| MA4384 | 671.8734657 | 829.2273093 | 1001.000153 | 948.7260361 | 1585.08687 | 1057.99067 |
| MA4383 | 3784.039204 | 4670.267256 | 5637.704146 | 3978.680929 | 6647.393093 | 4436.90501 |
| MA4382 | 2438.913767 | 3010.111284 | 3633.650054 | 5482.963085 | 9160.677017 | 6114.435115 |
| MA4381 | 479.3489906 | 591.6132933 | 714.1648504 | 2646.190705 | 4421.131056 | 2950.952089 |
| MA4380 | 4661.379022 | 5753.081467 | 6944.821241 | 13708.79052 | 22904.00287 | 15287.62985 |
| MA4379 | 16758.29568 | 20683.11542 | 24967.58304 | 114559.3572 | 191400.3896 | 127753.1411 |
| MA4378 | 4502.197273 | 5556.619097 | 6707.662069 | 5707.907259 | 9536.50317 | 6365.286076 |
| MA4377 | 292.1922719 | 360.6241708 | 435.326775 | 1912.969067 | 3196.098805 | 2133.285425 |
| MA4376 | 453.1280223 | 559.2513321 | 675.0991712 | 2917.926434 | 4875.13434 | 3253.98358 |
| MA4375 | 1966.781588 | 2427.404991 | 2930.237272 | 4621.826768 | 7721.930932 | 5154.121858 |
| MA4374 | 478.7445481 | 590.8672894 | 713.2643133 | 823.704107 | 1376.206107 | 918.5699845 |
| MA4373 | 14.72575205 | 18.17454681 | 21.93936926 | 98.89928983 | 165.2362851 | 110.2895061 |
| MA4372 | 35.78144098 | 44.16151187 | 53.30948421 | 295.2089516 | 493.2212411 | 329.2081219 |
| MA4371 | 257.5489169 | 317.8672864 | 383.7128841 | 519.9638195 | 868.7311107 | 579.847974 |
| MA4370 | 51.02132056 | 62.97059569 | 76.01483362 | 223.6525216 | 373.6681212 | 249.4105487 |
| MA4369 | 42.57438341 | 52.54537231 | 63.43004523 | 307.7219761 | 514.1274143 | 343.1622695 |
| MA4368 | 112.6926079 | 139.0853975 | 167.8966704 | 955.4413461 | 1596.306494 | 1065.479382 |
| MA4367 | 323.1265593 | 398.8033179 | 481.4146591 | 432.5232173 | 722.6394623 | 482.3368509 |
| MA4366 | 695.1589522 | 857.966294 | 1035.692364 | 1630.436903 | 2724.057345 | 1818.214075 |
| MA4365 | 437.7458571 | 540.2666392 | 652.1818356 | 810.4451458 | 1354.053658 | 903.7839907 |
| MA4364 | 59.1683367 | 73.02565608 | 88.15278045 | 352.5459696 | 589.017236 | 393.1486357 |
| MA4363 | 255.4206842 | 315.2406182 | 380.5421067 | 1451.532634 | 2425.152501 | 1618.705428 |
| MA4362 | 1223.836346 | 1510.460782 | 1823.349831 | 2543.156663 | 4248.986622 | 2836.051632 |
| MA4361 | 84.85396473 | 104.7268994 | 126.4208754 | 298.0821955 | 498.0217219 | 332.4122769 |
| MA4360 | 81.56113749 | 100.6628867 | 121.5150103 | 233.088422 | 389.4331801 | 259.9331804 |
| MA4359 | 206.7526489 | 255.1744509 | 308.0333482 | 1016.094168 | 1697.642378 | 1133.11758 |
| MA4358 | 93.65230102 | 115.5858202 | 139.5292008 | 338.4054318 | 565.39189 | 377.3795343 |
| MA4357 | 100.1015763 | 123.5455261 | 149.1377444 | 303.0583671 | 506.3356757 | 337.9615535 |
| MA4356 | 79.61920114 | 98.26614578 | 118.6217891 | 216.488575 | 361.6989359 | 241.4215315 |
| MA4355a | 78.59004602 | 96.99596088 | 117.0884878 | 441.6982962 | 737.968753 | 492.5686223 |
| MA4355 | 1324.139295 | 1634.254843 | 1972.787594 | 2573.357492 | 4299.444747 | 2869.730686 |
| MA4354 | 277.3237808 | 342.2734551 | 413.1747438 | 666.0934646 | 1112.877653 | 742.8073482 |
| MA4353 | 79.68126269 | 98.34274226 | 118.7142524 | 518.206679 | 865.795363 | 577.8884639 |
| MA4352 | 412.0876124 | 508.5991924 | 613.954538 | 1317.814974 | 2201.743319 | 1469.587525 |
| MA4351 | 834.3485818 | 1029.754358 | 1243.065995 | 12163.17092 | 20321.65429 | 13564.00148 |
| MA4350 | 826.4414118 | 1019.995316 | 1231.285387 | 7058.059435 | 11792.27397 | 7870.935073 |
| MA4349 | 889.2591259 | 1097.52504 | 1324.875243 | 7020.007212 | 11728.6981 | 7828.500382 |
| MA4348 | 21.61140976 | 26.67283659 | 32.19806345 | 67.26621633 | 112.3852326 | 75.01325629 |
| MA4347 | 428.0607951 | 528.31332 | 637.7524093 | 626.50214 | 1046.730329 | 698.6562968 |
| MA4346 | 6592.433616 | 8136.392145 | 9821.830147 | 7934.292995 | 13256.24381 | 8848.08432 |
| MA4345 | 1788.552796 | 2207.434731 | 2664.700594 | 4292.020955 | 7170.906881 | 4786.33236 |
| MA4344 | 424.9830483 | 524.51476 | 633.1669849 | 648.5396926 | 1083.549636 | 723.231911 |
| MA4343 | 78.66816688 | 97.09237778 | 117.2048773 | 250.4018807 | 418.3596931 | 279.2406275 |
| MA4342 | 246.6236327 | 304.3832831 | 367.4356955 | 1435.531986 | 2398.41937 | 1600.861988 |
| MA4341 | 762.597776 | 941.1993986 | 1136.167046 | 2412.428613 | 4030.572339 | 2690.267652 |
| MA4340 | 330.9955797 | 408.5152755 | 493.1384301 | 616.2561568 | 1029.611822 | 687.2302853 |
| MA4339 | 199.2196959 | 245.8772683 | 296.810272 | 1584.5815 | 2647.444293 | 1767.077512 |
| MA4338 | 712.2717345 | 879.0869174 | 1061.188084 | 3420.342441 | 5714.54739 | 3814.262763 |
| MA4337 | 635.3471123 | 784.1464253 | 946.5808509 | 2357.321179 | 3938.501429 | 2628.813503 |
| MA4336 | 321.172028 | 396.3910322 | 478.5026731 | 1361.233356 | 2274.284711 | 1518.006396 |
| MA4335 | 2285.051747 | 2820.214532 | 3404.416555 | 6375.888161 | 10652.53426 | 7110.198237 |
| MA4334 | 4302.999748 | 5310.769192 | 6410.884828 | 20710.61258 | 34602.31808 | 23095.8507 |
| MA4333 | 347.3705072 | 428.7252373 | 517.5348465 | 1570.491273 | 2623.903004 | 1751.364516 |
| MA4332 | 209.0023596 | 257.9510476 | 311.3851115 | 1317.906908 | 2201.896919 | 1469.690048 |
| MA4331 | 1213.573292 | 1497.794104 | 1808.059275 | 8034.933876 | 13424.38987 | 8960.315996 |
| MA4330 | 960.7032304 | 1185.701468 | 1431.31725 | 9282.146384 | 15508.17391 | 10351.16978 |
| MA4329 | 6876.626405 | 8487.143342 | 10245.23878 | 157680.0715 | 263444.4524 | 175840.0615 |
| MA4328 | 808.5183442 | 997.8746376 | 1204.582452 | 4820.442239 | 8053.768326 | 5375.611843 |
| MA4327 | 870.1112995 | 1073.892762 | 1296.347584 | 3884.80784 | 6490.554347 | 4332.220571 |
| MA4326 | 1232.116548 | 1520.680221 | 1835.686207 | 6452.151485 | 10779.95144 | 7195.244797 |
| MA4325 | 1164.009373 | 1436.622235 | 1734.215771 | 7652.125149 | 12784.81104 | 8533.419246 |
| MA4324 | 8.9007186 | 10.98528118 | 13.26086107 | 99.87386111 | 166.8645529 | 111.3763186 |
| MA4323 | 24.85984625 | 30.68206212 | 37.03779235 | 145.4033765 | 242.9331273 | 162.1494614 |
| MA4322 | 127.7467892 | 157.6652922 | 190.3253546 | 899.2327296 | 1502.395779 | 1002.797227 |
| MA4321 | 239.5738158 | 295.6823878 | 356.9324264 | 1541.068125 | 2574.744191 | 1718.552708 |
| MA4320 | 176.8018434 | 218.2091187 | 263.4107185 | 1082.428439 | 1808.470561 | 1207.091559 |
| MA4319 | 95.94964638 | 118.4212075 | 142.9519331 | 350.771854 | 586.053127 | 391.1701955 |
| MA4318 | 874.6848052 | 1079.53739 | 1303.161486 | 531.1804657 | 887.471356 | 592.3564397 |
| MA4317 | 185.3337137 | 228.7391669 | 276.1220458 | 551.1263285 | 920.795966 | 614.5994644 |
| MA4315 | 2331.974034 | 2878.126094 | 3474.324385 | 6457.392786 | 10788.70836 | 7201.089737 |
| MA4314 | 210.4134326 | 259.6925961 | 313.4874185 | 443.2646619 | 740.585763 | 494.315386 |
| MA4312 | 2868.025542 | 3539.721725 | 4272.96828 | 8796.796026 | 14697.27334 | 9809.921694 |
| MA4311 | 9.281955954 | 11.45580493 | 13.82885291 | 102.4863632 | 171.2293987 | 114.2897022 |
| MA4310 | 322.0121888 | 397.4279601 | 479.7543985 | 798.5140968 | 1334.119822 | 890.4788446 |
| MA4309a | 9.280225973 | 11.45366978 | 13.82627548 | 45.05091101 | 75.26894467 | 50.23941762 |
| MA4309 | 1349.657086 | 1665.748941 | 2010.805634 | 2979.078653 | 4977.304593 | 3322.178691 |
| MA4307 | 16.04239846 | 19.79955392 | 23.90099347 | 75.19071987 | 125.6251207 | 83.85042371 |
| MA4306 | 2282.582099 | 2817.166489 | 3400.737115 | 14456.3435 | 24152.97925 | 16121.27839 |
| MA4305 | 2637.219541 | 3254.860589 | 3929.098707 | 12584.68778 | 21025.90485 | 14034.06437 |
| MA4304 | 161.5808186 | 199.4233056 | 240.7334602 | 936.2538132 | 1564.248866 | 1044.082023 |
| MA4302 | 647.698585 | 799.3906328 | 964.9828667 | 2640.419217 | 4411.488324 | 2944.515899 |
| MA4301 | 577.7462678 | 713.0553707 | 860.7634208 | 2560.282963 | 4277.600438 | 2855.150365 |
| MA4299 | 583.3823447 | 720.0114257 | 869.1604094 | 2734.797874 | 4569.17175 | 3049.764132 |
| MA4298 | 92.88742905 | 114.641814 | 138.3896455 | 359.0473906 | 599.8795046 | 400.3988243 |
| MA4297 | 1194.03187 | 1473.676049 | 1778.945212 | 2998.130428 | 5009.135403 | 3343.424656 |
| MA4296 | 99.89048281 | 123.2849941 | 148.8232438 | 142.0110755 | 237.2654302 | 158.3664696 |
| MA4295 | 671.3782474 | 828.6161101 | 1000.262345 | 2814.34126 | 4702.0691 | 3138.468518 |
| MA4294 | 106.990386 | 132.047706 | 159.4011347 | 392.0886 | 655.0832043 | 437.2453848 |
| MA4293 | 82.73112457 | 102.1068867 | 123.2581321 | 331.4691404 | 553.8030604 | 369.6443912 |
| MA4292 | 1033.439114 | 1275.472213 | 1539.683831 | 4008.844964 | 6697.789745 | 4470.543032 |
| MA4291 | 1529.56421 | 1887.790602 | 2278.842798 | 4886.529716 | 8164.184176 | 5449.310605 |
| MA4290 | 425.6906637 | 525.3881001 | 634.2212357 | 782.6419413 | 1307.60137 | 872.7786954 |
| MA4289 | 597.187928 | 737.0502989 | 889.7288524 | 1736.396748 | 2901.090074 | 1936.377299 |
| MA4288 | 90.85573699 | 112.1342964 | 135.3627004 | 131.8193754 | 220.2376168 | 147.000993 |
| MA4287 | 1341.914842 | 1656.193451 | 1999.270742 | 6116.874093 | 10219.78573 | 6821.353558 |
| MA4286 | 9.580401531 | 11.82414694 | 14.27349626 | 32.71295774 | 54.65527223 | 36.48050413 |
| MA4285 | 1040.831778 | 1284.59625 | 1550.697895 | 2845.77557 | 4754.588068 | 3173.523113 |
| MA4284a | 17.70266215 | 21.84865403 | 26.37456074 | 54.07867417 | 90.35210704 | 60.3069069 |
| MA4284 | 1484.088863 | 1831.664856 | 2211.090713 | 4975.416181 | 8312.691505 | 5548.434111 |
| MA4283 | 47.0975364 | 58.12785499 | 70.16892848 | 204.6730386 | 341.958093 | 228.2452015 |
| MA4281 | 932.1866926 | 1150.506312 | 1388.831484 | 1457.013373 | 2434.309462 | 1624.817383 |
| MA4280 | 402.640895 | 496.9400386 | 599.8802129 | 599.6240629 | 1001.823701 | 668.6826756 |
| MA4279 | 1664.321699 | 2054.108511 | 2479.613143 | 2403.947969 | 4016.403279 | 2680.810294 |
| MA4278 | 123.9005492 | 152.9182566 | 184.5949796 | 413.6287443 | 691.0714649 | 461.2663042 |
| MA4277 | 7797.871237 | 9624.145191 | 11617.76838 | 96634.55549 | 161452.4734 | 107763.9426 |
| MA4276 | 7346.369988 | 9066.901625 | 10945.09288 | 95323.60796 | 159262.2038 | 106302.0135 |
| MA4275 | 6354.316244 | 7842.507303 | 9467.067624 | 70493.11631 | 117776.5855 | 78611.79786 |
| MA4274 | 5465.083693 | 6745.015062 | 8142.231975 | 51229.13579 | 85591.23224 | 57129.18761 |
| MA4273 | 3878.119405 | 4786.381192 | 5777.87086 | 13941.40825 | 23292.64964 | 15547.03813 |
| MA4272 | 1400.204815 | 1728.135029 | 2086.114881 | 5047.63786 | 8433.356091 | 5628.97355 |
| MA4271 | 5999.959882 | 7405.160113 | 8939.124804 | 20574.50698 | 34374.91924 | 22944.06984 |
| MA4270 | 196.2861569 | 242.2566898 | 292.4396977 | 306.8416747 | 512.6566481 | 342.180584 |
| MA4269 | 784.7375264 | 968.5243141 | 1169.152265 | 1027.94466 | 1717.441623 | 1146.332891 |
| MA4268 | 46037.76279 | 56819.8807 | 68590.00983 | 28623.55436 | 47822.88928 | 31920.12479 |
| MA4267 | 3241.550488 | 4000.726812 | 4829.469686 | 11453.64411 | 19136.21025 | 12772.75858 |
| MA4266 | 797.3577027 | 984.1001559 | 1187.954613 | 2060.671604 | 3442.873262 | 2297.998841 |
| MA4265 | 2278.01072 | 2811.524485 | 3393.926381 | 16935.46286 | 28294.97536 | 18885.91756 |
| MA4264 | 3416.815828 | 4217.039577 | 5090.591224 | 7132.727559 | 11917.02596 | 7954.202714 |
| MA4263 | 401.5661133 | 495.6135413 | 598.2789343 | 1805.74322 | 3016.9509 | 2013.710394 |
| MA4262 | 1013.074341 | 1250.337976 | 1509.343086 | 11198.76511 | 18710.37039 | 12488.52519 |
| MA4261 | 1532.046672 | 1890.85446 | 2282.541329 | 11158.11586 | 18642.45553 | 12443.19438 |
| MA4260a | 639.5123252 | 789.2871378 | 952.7864521 | 3904.578446 | 6523.586147 | 4354.268155 |
| MA4260b | 690.758757 | 852.5355662 | 1029.136671 | 4568.912457 | 7633.524189 | 5095.113415 |
| MA4259 | 1719.023235 | 2121.621234 | 2561.110997 | 14949.14505 | 24976.32892 | 16670.83582 |
| MA4258 | 862.1674913 | 1064.088501 | 1284.51239 | 8673.47554 | 14491.23527 | 9672.398407 |
| MA4257 | 160.797325 | 198.4563165 | 239.5661612 | 1395.191837 | 2331.020945 | 1555.87587 |
| MA4256 | 82.05917253 | 101.2775624 | 122.2570149 | 727.941909 | 1216.211127 | 811.7788686 |
| MA4255 | 536.9160483 | 662.6626483 | 799.9319428 | 2855.079739 | 4770.133035 | 3183.898841 |
| MA4254 | 4052.402954 | 5001.482227 | 6037.529662 | 13851.74809 | 23142.84966 | 15447.05183 |
| MA4253 | 61.93122839 | 76.43562144 | 92.26911358 | 207.3069608 | 346.3587264 | 231.1824721 |
| MA4252 | 69.13281749 | 85.32383426 | 102.9985026 | 325.0499374 | 543.0781577 | 362.4858896 |
| MA4251 | 36.55790699 | 45.11982745 | 54.46631303 | 195.5062321 | 326.6426236 | 218.0226553 |
| MA4250 | 57.71594774 | 71.23311529 | 85.98891828 | 559.3760591 | 934.5792282 | 623.7993153 |
| MA4249 | 185.9190158 | 229.4615476 | 276.9940663 | 867.8837 | 1450.019294 | 967.8377346 |
| MA4248 | 107.602672 | 132.8033904 | 160.3133576 | 956.5142459 | 1598.099045 | 1066.675847 |
| MA4247 | 277.7619283 | 342.8142176 | 413.8275241 | 4103.720477 | 6856.303294 | 4576.345344 |
| MA4246 | 1108.477215 | 1368.084357 | 1651.480403 | 10579.06774 | 17675.00915 | 11797.45737 |
| MA4245 | 653.8928287 | 807.0355783 | 974.2114479 | 8461.512102 | 14137.09672 | 9436.023172 |
| MA4244 | 73.14655831 | 90.27759962 | 108.9784309 | 1105.754067 | 1847.441923 | 1233.103596 |
| MA4243 | 151.2617734 | 186.6875234 | 225.3594852 | 2002.171855 | 3345.13463 | 2232.761684 |
| MA4242 | 243.389864 | 300.3921607 | 362.6178197 | 2432.570881 | 4064.225094 | 2712.729702 |
| MA4241 | 103.6465406 | 127.9207267 | 154.4192594 | 762.3037378 | 1273.621256 | 850.0981441 |
| MA4240 | 2276.592542 | 2809.774168 | 3391.813489 | 2010.542068 | 3359.119189 | 2242.095894 |
| MA4239 | 546.2207752 | 674.1465571 | 813.7947213 | 942.8549555 | 1575.277744 | 1051.443418 |
| MA4238 | 385.2323585 | 475.4543949 | 573.9438594 | 800.512719 | 1337.459026 | 892.7076478 |
| MA4237 | 3262.524798 | 4026.61334 | 4860.718558 | 3781.360791 | 6317.71988 | 4216.859541 |
| MA4236 | 5108.850783 | 6305.351833 | 7611.493352 | 5788.654017 | 9671.411057 | 6455.332425 |
| MA4235 | 880.8262371 | 1087.117155 | 1312.311385 | 4203.339059 | 7022.741337 | 4687.436983 |
| MA4234 | 81.76695851 | 100.9169114 | 121.8216557 | 156.3627046 | 261.2434576 | 174.3709737 |
| MA4233 | 68.80182448 | 84.91532216 | 102.505368 | 206.0220103 | 344.2118916 | 229.7495341 |
| MA4232 | 865.4965067 | 1068.197177 | 1289.47217 | 1796.352965 | 3001.262104 | 2003.238665 |
| MA4231a | 33.79426 | 41.70892991 | 50.34885461 | 115.9797814 | 193.7735675 | 129.3371553 |
| MA4231 | 57.10616077 | 70.48051524 | 85.08041858 | 150.4270124 | 251.3263821 | 167.7516687 |
| MA4230 | 343.4194312 | 423.8488129 | 511.6482803 | 1381.526642 | 2308.18978 | 1540.636857 |
| MA4229 | 51.43684857 | 63.48344103 | 76.63391388 | 386.2625222 | 645.3492673 | 430.7483185 |
| MA4228 | 243.5022999 | 300.5309292 | 362.7853338 | 725.8177753 | 1212.662225 | 809.4100988 |
| MA4227 | 330.7315931 | 408.1894628 | 492.7451259 | 5478.473166 | 9153.175471 | 6109.428093 |
| MA4226 | 49.07999555 | 60.57460925 | 73.12252319 | 227.9954799 | 380.9241318 | 254.2536849 |
| MA4224n | 17.70242593 | 21.84836249 | 26.3742088 | 87.12947748 | 145.5718358 | 97.16416623 |
| MA4223 | 103.5144492 | 127.7576992 | 154.2224612 | 460.0169147 | 768.5746397 | 512.9969932 |
| MA4222 | 79.95095419 | 98.67559594 | 119.1160561 | 635.6428557 | 1062.002207 | 708.8497473 |
| MA4221 | 86.40612482 | 106.6425779 | 128.733383 | 403.2228986 | 673.6858669 | 449.6620188 |
| MA4220 | 916.5200222 | 1131.170482 | 1365.490274 | 16577.51737 | 27696.93685 | 18486.74754 |
| MA4219 | 1050.29792 | 1296.279378 | 1564.801161 | 19371.95584 | 32365.75331 | 21603.02107 |
| MA4218 | 1559.218062 | 1924.389433 | 2323.023007 | 32033.55743 | 53520.16214 | 35722.8574 |
| MA4217 | 1084.473522 | 1338.45896 | 1615.718163 | 29925.10581 | 49997.46028 | 33371.57573 |
| MA4216 | 2311.842416 | 2853.279619 | 3444.331011 | 9898.286375 | 16537.59164 | 11038.27052 |
| MA4214 | 104.0563396 | 128.4265013 | 155.0298044 | 229.0257629 | 382.6454802 | 255.4026254 |
| MA4213 | 538.8181691 | 665.0102487 | 802.7658442 | 3759.082898 | 6280.499024 | 4192.015907 |
| MA4212 | 913.5194139 | 1127.467126 | 1361.019776 | 813.673001 | 1359.446606 | 907.3835976 |
| MA4211 | 157.7860732 | 194.7398247 | 235.0798052 | 288.8981076 | 482.6773796 | 322.170459 |
| MA4210 | 63.63441731 | 78.53769994 | 94.80663361 | 469.3246691 | 784.1255983 | 523.3767203 |
| MA4209a | 15.88917474 | 19.61044496 | 23.67271096 | 32.18297958 | 53.76980965 | 35.88948848 |
| MA4209 | 128.1131223 | 158.1174211 | 190.8711411 | 432.2826821 | 722.2375874 | 482.0686132 |
| MA4208 | 504.1956063 | 622.2790263 | 751.1829311 | 2477.392838 | 4139.111513 | 2762.713797 |
| MA4207 | 1175.548962 | 1450.864415 | 1751.408191 | 5785.014518 | 9665.330353 | 6451.273765 |
| MA4206 | 276.7137484 | 341.5205522 | 412.2658785 | 1083.655093 | 1810.519996 | 1208.459486 |
| MA4205 | 234.3305526 | 289.2111439 | 349.1206768 | 730.3550111 | 1220.242825 | 814.4698874 |
| MA4204 | 486.0081422 | 599.8320288 | 724.0860813 | 1436.808298 | 2400.551773 | 1602.285293 |
| MA4203 | 635.0318406 | 783.7573165 | 946.111139 | 1825.73402 | 3050.35059 | 2036.003532 |
| MA4202 | 220.4751171 | 272.1107433 | 328.477961 | 716.5062059 | 1197.104892 | 799.0261173 |
| MA4201 | 18.34313721 | 22.63912937 | 27.32878153 | 79.8449874 | 133.4012521 | 89.04072253 |
| MA4200 | 1345.69518 | 1660.85915 | 2004.902932 | 1528.172466 | 2553.198729 | 1704.171857 |
| MA4199 | 254.8431551 | 314.5278309 | 379.6816669 | 18655.53297 | 31168.78765 | 20804.08788 |
| MA4198 | 134.131097 | 165.5448151 | 199.8371056 | 9089.884779 | 15186.95225 | 10136.76544 |
| MA4197 | 143.4711509 | 177.0723247 | 213.7525165 | 10769.26352 | 17992.77931 | 12009.55797 |
| MA4196 | 60.27400771 | 74.39027701 | 89.80007999 | 5004.197303 | 8360.777651 | 5580.52995 |
| MA4195 | 1773.952097 | 2189.414524 | 2642.947537 | 32662.74706 | 54571.38259 | 36424.5107 |
| MA4194 | 346.5064426 | 427.6588073 | 516.2475077 | 6655.512009 | 11119.71665 | 7422.026322 |
| MA4193 | 529.6740445 | 653.7245555 | 789.1423414 | 4082.406718 | 6820.693266 | 4552.576883 |
| MA4192 | 377.2035702 | 465.5452515 | 561.982056 | 1608.870755 | 2688.025639 | 1794.164157 |
| MA4192a | 492.7515586 | 608.1547641 | 734.1328552 | 2549.6216 | 4259.787934 | 2843.261134 |
| MA4191 | 474.969008 | 586.2075116 | 707.6392715 | 1158.14969 | 1934.982067 | 1291.533615 |
| MA4190 | 617.7740871 | 762.4577694 | 920.3994316 | 3449.567506 | 5763.375255 | 3846.853674 |
| MA4189 | 248.3800494 | 306.5510554 | 370.0525176 | 2056.818613 | 3436.435866 | 2293.702102 |
| MA4188 | 139.2518673 | 171.864878 | 207.4663573 | 532.9882606 | 890.4917347 | 594.372438 |
| MA4187 | 68.53895564 | 84.59088901 | 102.1137292 | 397.6276072 | 664.3375171 | 443.4223186 |
| MA4186 | 160.3618447 | 197.918846 | 238.9173547 | 774.7957785 | 1294.49237 | 864.0288912 |
| MA4185 | 152.8653448 | 188.6666538 | 227.7485887 | 651.3915121 | 1088.314322 | 726.4121741 |
| MA4184 | 235.8306273 | 291.0625385 | 351.3555842 | 1164.706374 | 1945.936667 | 1298.845431 |
| MA4182 | 23.48111451 | 28.9804292 | 34.98366943 | 93.72868026 | 156.5974736 | 104.5233982 |
| MA4181 | 684.3596089 | 844.6377273 | 1019.60281 | 1602.983614 | 2678.189679 | 1787.598994 |
| MA4180 | 534.2237212 | 659.3397738 | 795.9207412 | 1125.075548 | 1879.723345 | 1254.650328 |
| MA4179 | 70.02555521 | 86.42565259 | 104.3285605 | 244.3388453 | 408.2298585 | 272.4793132 |
| MA4178 | 90.6210667 | 111.8446659 | 135.0130736 | 382.2535694 | 638.6512974 | 426.2776552 |
| MA4177 | 144.2078244 | 177.9815283 | 214.8500599 | 348.1560314 | 581.6827334 | 388.2531089 |
| MA4176 | 183.2982042 | 226.2269378 | 273.0894133 | 529.7960379 | 885.158319 | 590.8125675 |
| MA4175 | 195.8116306 | 241.6710286 | 291.7327179 | 861.3638137 | 1439.126175 | 960.5669539 |
| MA4174 | 100.6798649 | 124.2592508 | 149.9993159 | 403.729518 | 674.5323029 | 450.2269856 |
| MA4173 | 1559.573775 | 1924.828456 | 2323.552973 | 7391.499988 | 12349.37077 | 8242.777924 |
| MA4172 | 92.22539592 | 113.8247316 | 137.4033061 | 375.4374062 | 627.2631722 | 418.6764755 |
| MA4171 | 318.8602477 | 393.5378293 | 475.0584346 | 1119.850931 | 1870.994301 | 1248.823994 |
| MA4170 | 142.1760314 | 175.473886 | 211.8229644 | 365.2796357 | 610.2920468 | 407.3488362 |
| MA4169 | 8429.974078 | 10404.28753 | 12559.51571 | 23855.3463 | 39856.39136 | 26602.76292 |
| MA4168 | 873.5446561 | 1078.130216 | 1301.462819 | 1388.856044 | 2320.4354 | 1548.810385 |
| MA4167 | 1827.372056 | 2255.345524 | 2722.536016 | 2866.425351 | 4789.08875 | 3196.551125 |
| MA4166 | 101.3898428 | 125.1355067 | 151.0570865 | 256.2931103 | 428.2024826 | 285.8103491 |
| MA4165 | 1111.088943 | 1371.307756 | 1655.371524 | 897.4353833 | 1499.392858 | 1000.792881 |
| MA4164 | 797.0933078 | 983.7738393 | 1187.5607 | 529.702843 | 885.0026135 | 590.7086394 |
| MA4163 | 2571.719461 | 3174.020285 | 3831.51249 | 14043.06586 | 23462.4944 | 15660.40363 |
| MA4162 | 777.569001 | 959.6769085 | 1158.472137 | 894.5024443 | 1494.492641 | 997.5221557 |
| MA4161 | 886.2547691 | 1093.817058 | 1320.39916 | 2295.831745 | 3835.767773 | 2560.242341 |
| MA4160a | 63.9821036 | 78.96681491 | 95.32463892 | 167.796608 | 280.346686 | 187.121718 |
| MA4160 | 7898.622355 | 9748.492382 | 11767.87385 | 31495.90456 | 52621.877 | 35123.28313 |
| MA4159 | 13570.19891 | 16748.36126 | 20217.75212 | 52828.1146 | 88262.73089 | 58912.32058 |
| MA4158 | 24752.4091 | 30549.46302 | 36877.7256 | 104084.3554 | 173899.2488 | 116071.7348 |
| MA4157 | 5006.828781 | 6179.436114 | 7459.494434 | 23757.60391 | 39693.08797 | 26493.76356 |
| MA4156 | 14394.59853 | 17765.8366 | 21445.99553 | 70042.20545 | 117023.2248 | 78108.95566 |
| MA4155 | 10120.02043 | 12490.14545 | 15077.45509 | 48426.8035 | 80909.22716 | 54004.11114 |
| MA4154 | 4953.233157 | 6113.28831 | 7379.644239 | 25234.19949 | 42160.11447 | 28140.41843 |
| MA4153 | 45410.49339 | 56045.7038 | 67655.46367 | 172896.7966 | 288867.8414 | 192809.2946 |
| MA4152 | 6741.072178 | 8319.842097 | 10043.2814 | 22196.67436 | 37085.16024 | 24753.06199 |
| MA4151 | 958.5636971 | 1183.060853 | 1428.129636 | 5611.601647 | 9375.60028 | 6257.888961 |
| MA4150 | 1398.988095 | 1726.633351 | 2084.302133 | 5909.018164 | 9872.509817 | 6589.558893 |
| MA4149 | 2309.040703 | 2849.82174 | 3440.156839 | 7763.159207 | 12970.32152 | 8657.24108 |
| MA4148 | 80.41552007 | 99.24896388 | 119.8081961 | 185.1734117 | 309.3790329 | 206.4998055 |
| MA4147 | 344.9361339 | 425.7207298 | 513.9079612 | 599.490712 | 1001.600905 | 668.5339667 |
| MA4146 | 18.8008198 | 23.20400196 | 28.01066639 | 77.47476818 | 129.4412013 | 86.39752552 |
| MA4145 | 84.64097805 | 104.4640309 | 126.1035542 | 278.3206782 | 465.0051077 | 310.3748286 |
| MA4144 | 838.6435601 | 1035.055227 | 1249.464929 | 1917.205972 | 3203.177627 | 2138.010294 |
| MA4143 | 21.22047685 | 26.19034657 | 31.61562655 | 38.36666882 | 64.10122698 | 42.78535227 |
| MA4140 | 17.15620384 | 21.1742143 | 25.56041211 | 59.55739794 | 99.5057012 | 66.41661446 |
| MA4139 | 32.55775637 | 40.18283514 | 48.50663225 | 279.5368442 | 467.0370206 | 311.7310602 |
| MA4138 | 17.99384236 | 22.2080291 | 26.80837967 | 259.73961 | 433.9607323 | 289.6537816 |
| MA4137 | 187.7650776 | 231.73996 | 279.7444475 | 1124.916739 | 1879.458015 | 1254.47323 |
| MA4136 | 7.559496517 | 9.32994273 | 11.26262244 | 62.18570161 | 103.8969474 | 69.34761947 |
| MA4135 | 24.82011461 | 30.63302528 | 36.97859761 | 204.3847888 | 341.4764988 | 227.9237541 |
| MA4134 | 125.8525742 | 155.327449 | 187.5032317 | 380.8044142 | 636.230117 | 424.6616011 |
| MA4132 | 66.83452925 | 82.48728323 | 99.57436548 | 64.85207745 | 108.3518029 | 72.32108141 |
| MA4131 | 387.991187 | 478.8593455 | 578.054139 | 1971.787387 | 3294.369689 | 2198.877842 |
| MA4130 | 64.01061319 | 79.00200149 | 95.36711433 | 601.7026268 | 1005.296468 | 671.0006274 |
| MA4129 | 52.75489298 | 65.11017356 | 78.59762093 | 519.8247862 | 868.4988203 | 579.6929282 |
| MA4128 | 5.583294881 | 6.8909115 | 8.318350579 | 41.5789291 | 69.46812047 | 46.36756808 |
| MA4127 | 47.69123542 | 58.86059927 | 71.05345934 | 237.5496107 | 396.8867244 | 264.9081635 |
| MA4126 | 258.947482 | 319.5933977 | 385.7965561 | 598.4446757 | 999.8532365 | 667.3674586 |
| MA4125 | 238.1252436 | 293.8945577 | 354.7742507 | 586.0748385 | 979.186294 | 653.5729891 |
| MA4124 | 96.07139429 | 118.571469 | 143.133321 | 178.0731902 | 297.5163165 | 198.5818526 |
| MA4121n | 572.4072318 | 706.4659238 | 852.8089827 | 3447.45253 | 5759.841652 | 3844.495116 |
| MA4120 | 357.6356842 | 441.3945352 | 532.8285652 | 4284.440194 | 7158.241302 | 4777.878523 |
| MA4119 | 31.70753941 | 39.13349601 | 47.23992453 | 571.0534305 | 954.0892314 | 636.8215677 |
| MA4118 | 1504.346346 | 1856.666674 | 2241.271609 | 20307.33366 | 33928.5386 | 22646.12621 |
| MA4117 | 2107.480574 | 2601.055905 | 3139.859639 | 47590.16581 | 79511.41224 | 53071.11801 |
| MA4116 | 1563.360184 | 1929.501647 | 2329.194206 | 26267.52701 | 43886.54953 | 29292.75413 |
| MA4115 | 1446.458867 | 1785.221854 | 2155.027132 | 30829.99045 | 51509.299 | 34380.6758 |
| MA4114 | 2190.075868 | 2702.99515 | 3262.915403 | 28614.87065 | 47808.38095 | 31910.44098 |
| MA4113 | 1302.599627 | 1607.670549 | 1940.696415 | 10999.09368 | 18376.76875 | 12265.85764 |
| MA4112 | 2636.174764 | 3253.571123 | 3927.542131 | 19044.35342 | 31818.41058 | 21237.68872 |
| MA4111 | 1663.271141 | 2052.81191 | 2478.047954 | 20779.4879 | 34717.39172 | 23172.65838 |
| MA4110 | 2407.84233 | 2971.762867 | 3587.357835 | 21494.46777 | 35911.94648 | 23969.98237 |
| MA4109 | 1887.151474 | 2329.125377 | 2811.599224 | 7224.185706 | 12069.82993 | 8056.194082 |
| MA4106 | 40.80251894 | 50.35853433 | 60.79020798 | 93.5190457 | 156.247226 | 104.2896201 |
| MA4105 | 132.3141612 | 163.30235 | 197.1301181 | 304.9967684 | 509.5742654 | 340.1232001 |
| MA4104 | 429.4373838 | 530.012308 | 639.8033394 | 419.2898108 | 700.5297088 | 467.579355 |
| MA4103 | 3572.1265 | 4408.724258 | 5321.983018 | 5886.177281 | 9834.348344 | 6564.087429 |
| MA4102 | 206.4037566 | 254.7438475 | 307.5135461 | 2231.25585 | 3727.877402 | 2488.229249 |
| MA4101 | 950.5803891 | 1173.207841 | 1416.235592 | 9599.218103 | 16037.92243 | 10704.7586 |
| MA4100 | 1252.052986 | 1545.285804 | 1865.38879 | 6231.810203 | 10411.81558 | 6949.526843 |
| MA4099 | 4776.747196 | 5895.469053 | 7116.704142 | 6320.188044 | 10559.47313 | 7048.083146 |
| MA4098 | 269.4727998 | 332.5837618 | 401.4778491 | 570.5295272 | 953.2139185 | 636.2373267 |
| MA4097 | 387.5033181 | 478.2572169 | 577.3272806 | 819.614228 | 1369.372929 | 914.0090747 |
| MA4096 | 80851.97787 | 99787.6408 | 120458.4589 | 54987.23861 | 91870.0938 | 61320.11057 |
| MA4095 | 4684.946407 | 5782.168372 | 6979.933442 | 32509.87606 | 54315.97292 | 36254.03358 |
| MA4094 | 6418.614667 | 7921.86452 | 9562.863537 | 14752.95311 | 24648.54065 | 16452.04849 |
| MA4093 | 4979.153555 | 6145.279306 | 7418.262109 | 14106.78846 | 23568.95912 | 15731.46515 |
| MA4092 | 152.4801917 | 188.1912972 | 227.1747629 | 1308.309283 | 2185.861659 | 1458.987066 |
| MA4091a | 109.6233519 | 135.2973168 | 163.323896 | 1220.541965 | 2039.224148 | 1361.111598 |
| MA4091 | 183.8742885 | 226.9379418 | 273.9477005 | 1797.481273 | 3003.147227 | 2004.49692 |
| MA4090a | 130.5657781 | 161.1444929 | 194.525265 | 4416.611623 | 7379.067113 | 4925.272116 |
| MA4090 | 11.86637836 | 14.64550321 | 17.67929106 | 136.7218018 | 228.4283603 | 152.468031 |
| MA4089 | 27.57647832 | 34.03493379 | 41.08520494 | 203.2912132 | 339.649404 | 226.7042315 |
| MA4088 | 147.6192644 | 182.1919329 | 219.9326418 | 861.3337663 | 1439.075973 | 960.533446 |
| MA4087 | 785.5616869 | 969.5414943 | 1170.380152 | 3645.757991 | 6091.161097 | 4065.639388 |
| MA4086 | 296.8470484 | 366.3691034 | 442.2617594 | 2077.583935 | 3471.12959 | 2316.858963 |
| MA4085 | 404.8480443 | 499.6641058 | 603.1685654 | 1169.506192 | 1953.955978 | 1304.198044 |
| MA4084 | 232.494061 | 286.9445431 | 346.3845539 | 787.2947972 | 1315.375143 | 877.9674199 |
| MA4083 | 141.5173682 | 174.6609629 | 210.8416459 | 350.6931557 | 585.9216415 | 391.0824334 |
| MA4082 | 2736.515537 | 3377.411867 | 4077.036246 | 4378.941797 | 7316.130138 | 4883.263862 |
| MA4081 | 441.3030672 | 544.6569535 | 657.4815951 | 2375.244548 | 3968.446951 | 2648.801103 |
| MA4080a | 140.3474108 | 173.2169996 | 209.0985683 | 63.39404947 | 105.9157983 | 70.69513257 |
| MA4080 | 1094.146248 | 1350.397055 | 1630.12921 | 672.1847035 | 1123.054609 | 749.6001142 |
| MA4079 | 1008.883677 | 1245.165853 | 1503.099567 | 3317.652056 | 5542.977122 | 3699.745542 |
| MA4078 | 302.6359767 | 373.513808 | 450.8864758 | 1301.037854 | 2173.712896 | 1450.878187 |
| MA4077 | 573.7258037 | 708.0933074 | 854.7734757 | 4093.963088 | 6840.001106 | 4565.464198 |
| MA4076 | 135.5626497 | 167.3116397 | 201.9699245 | 861.3086021 | 1439.03393 | 960.5053837 |
| MA4075 | 5013.949146 | 6188.22408 | 7470.10281 | 19590.82684 | 32731.4327 | 21847.09941 |
| MA4074 | 601.1294961 | 741.9149885 | 895.6012531 | 815.2938785 | 1362.154692 | 909.1911512 |
| MA4073a | 222.3976004 | 274.4834752 | 331.3421998 | 252.5997656 | 422.0318158 | 281.6916424 |
| MA4072 | 86.08974665 | 106.2521034 | 128.2620225 | 257.475566 | 430.178074 | 287.128988 |
| MA4071 | 88.66530201 | 109.430858 | 132.09925 | 288.8076556 | 482.5262566 | 322.0695896 |
| MA4070 | 29.3556615 | 36.23080452 | 43.73594608 | 176.4612418 | 294.8231489 | 196.7842563 |
| MA4069 | 49.3793701 | 60.9440978 | 73.56855058 | 217.8307747 | 363.9414201 | 242.9183121 |
| MA4068 | 1767.262198 | 2181.15784 | 2632.980496 | 1454.28959 | 2429.758694 | 1621.779902 |
| MA4067 | 1708.132861 | 2108.180317 | 2544.88582 | 1086.591856 | 1815.426602 | 1211.734476 |
| MA4066a | 119.2110774 | 147.1305029 | 177.608304 | 662.5606002 | 1106.97511 | 738.8676044 |
| MA4066 | 130.8355974 | 161.4775043 | 194.9272591 | 232.2903037 | 388.0997214 | 259.0431428 |
| MA4065 | 395.526798 | 488.1598087 | 589.2811752 | 1945.885367 | 3251.093812 | 2169.992691 |
| MA4064 | 2504.502039 | 3091.060435 | 3731.367665 | 9039.487229 | 15102.7504 | 10080.56361 |
| MA4063 | 1907.739766 | 2354.53548 | 2842.272981 | 13294.81721 | 22212.35572 | 14825.9793 |
| MA4062 | 335.6244815 | 414.2282736 | 500.0348647 | 1755.416587 | 2932.867527 | 1957.58765 |
| MA4061 | 337.8197824 | 416.9377174 | 503.3055646 | 2440.4276 | 4077.351731 | 2721.491278 |
| MA4060 | 2096.68236 | 2587.728733 | 3123.771769 | 11853.79996 | 19804.77182 | 13219.00031 |
| MA4059 | 2368.057511 | 2922.660379 | 3528.083862 | 14260.46824 | 23825.72008 | 15902.84422 |
| MA4058 | 1926.398214 | 2377.563766 | 2870.071532 | 3753.687343 | 6271.484385 | 4185.998947 |
| MA4056 | 18.85371834 | 23.26928942 | 28.08947802 | 74.60423082 | 124.6452424 | 83.19638881 |
| MA4055 | 1301.780829 | 1606.659987 | 1939.476517 | 12393.81943 | 20707.01098 | 13821.21374 |
| MA4054 | 14.27067558 | 17.61289069 | 21.26136717 | 403.7002649 | 674.4834282 | 450.1943634 |
| MA4053 | 7922.943139 | 9778.509134 | 11804.10852 | 17919.20349 | 29938.56296 | 19982.95544 |
| MA4052 | 2956.123801 | 3648.452738 | 4404.222713 | 6759.525115 | 11293.49686 | 7538.0186 |
| MA4051 | 2582.4869 | 3187.309475 | 3847.55451 | 5049.413554 | 8436.322838 | 5630.95375 |
| MA4050 | 4037.767694 | 4983.419366 | 6015.725114 | 7377.377126 | 12325.77496 | 8227.028534 |
| MA4049 | 40.02354031 | 49.39711766 | 59.62963569 | 226.6833611 | 378.7319053 | 252.7904495 |
| MA4048 | 1450.16642 | 1789.797722 | 2160.550882 | 17230.79469 | 28788.4019 | 19215.26271 |
| MA4047 | 4702.738271 | 5804.127119 | 7006.440901 | 22657.23889 | 37854.64982 | 25266.66967 |
| MA4046 | 2247.681397 | 2774.091986 | 3348.739812 | 20401.39329 | 34085.68901 | 22751.01867 |
| MA4045 | 311.2407891 | 384.1338812 | 463.7064768 | 3586.778699 | 5992.621268 | 3999.86746 |
| MA4044 | 402.3382399 | 496.5665012 | 599.429298 | 1159.45925 | 1937.170019 | 1292.993997 |
| MA4043 | 1462.841396 | 1805.441198 | 2179.434874 | 2929.374165 | 4894.26067 | 3266.749744 |
| MA4042 | 5260.892951 | 6493.002521 | 7838.01552 | 11698.8161 | 19545.83208 | 13046.16698 |
| MA4041 | 3055.698638 | 3771.348162 | 4552.575688 | 6525.442962 | 10902.4034 | 7276.977242 |
| MA4040 | 1852.16375 | 2285.943472 | 2759.472271 | 3688.267447 | 6162.183898 | 4113.044652 |
| MA4039 | 214.4401969 | 264.6624351 | 319.48675 | 364.0957745 | 608.3141071 | 406.0286299 |
| MA4038 | 55.11244124 | 68.01986339 | 82.11004742 | 580.2492822 | 969.4532282 | 647.0765043 |
| MA4037 | 46.59721148 | 57.51035316 | 69.42351234 | 184.565048 | 308.3626075 | 205.8213767 |
| MA4036 | 30.0223381 | 37.05361784 | 44.7292036 | 145.4910411 | 243.0795931 | 162.2472223 |
| MA4035 | 139.0718975 | 171.6427591 | 207.1982269 | 751.457061 | 1255.499138 | 838.0022571 |
| MA4034 | 276.5388621 | 341.3047073 | 412.0053217 | 1017.55482 | 1700.082767 | 1134.746455 |
| MA4033 | 132.0471872 | 162.9728502 | 196.7323631 | 906.4753818 | 1514.496462 | 1010.874014 |
| MA4032 | 37.1668173 | 45.87134554 | 55.37350664 | 414.377723 | 692.3228234 | 462.1015428 |
| MA4031 | 48.22478746 | 59.51911006 | 71.84837937 | 128.5576437 | 214.7880687 | 143.3636081 |
| MA4030 | 24.49911688 | 30.23684935 | 36.50035461 | 37.04771841 | 61.89758663 | 41.31449854 |
| MA4029 | 695.8255777 | 858.7890443 | 1036.685545 | 3716.511291 | 6209.372385 | 4144.541335 |
| MA4028 | 953.912147 | 1177.319902 | 1421.199458 | 516.8786207 | 863.5765055 | 576.4074533 |
| MA4027 | 811.006255 | 1000.945221 | 1208.289101 | 1356.159182 | 2265.807021 | 1512.34783 |
| MA4026 | 1456.7294 | 1797.897762 | 2170.32883 | 4695.997554 | 7845.851995 | 5236.834882 |
| MA4025 | 21.08655252 | 26.02505695 | 31.4160975 | 109.4266563 | 182.8249141 | 122.0293077 |
| MA4024 | 545.8769705 | 673.7222328 | 813.282499 | 1316.770026 | 2199.99747 | 1468.422231 |
| MA4023 | 124.3447843 | 153.4665322 | 185.2568296 | 943.6421893 | 1576.593018 | 1052.321318 |
| MA4022 | 93.6476076 | 115.5800275 | 139.5222082 | 510.0465758 | 852.161846 | 568.7885629 |
| MA4021 | 355.0910005 | 438.2538825 | 529.0373324 | 1241.320797 | 2073.940444 | 1384.283525 |
| MA4020a | 33.7475975 | 41.65133897 | 50.27933382 | 124.2724964 | 207.628646 | 138.584941 |
| MA4020 | 835.5682065 | 1031.259621 | 1244.88307 | 3940.399871 | 6583.4349 | 4394.215128 |
| MA4019 | 255.1953018 | 314.962451 | 380.2063176 | 1449.544284 | 2421.830458 | 1616.48808 |
| MA4018 | 412.2039097 | 508.7427267 | 614.1278052 | 252.8399237 | 422.4330607 | 281.9594595 |
| MA4017 | 684.9631766 | 845.3826515 | 1020.502044 | 192.526025 | 321.6634336 | 214.6992182 |
| MA4016 | 111.8768574 | 138.0785969 | 166.6813129 | 298.9923876 | 499.5424281 | 333.4272956 |
| MA4015a | 673.4090239 | 831.1224977 | 1003.287926 | 3502.665078 | 5852.088183 | 3906.066486 |
| MA4015b | 768.5396881 | 948.5329161 | 1145.019687 | 4237.234792 | 7079.372736 | 4725.236483 |
| MA4014 | 296.8518864 | 366.3750745 | 442.2689673 | 862.0951154 | 1440.347999 | 961.3824795 |
| MA4013 | 262.7645409 | 324.3044179 | 391.4834551 | 740.9387431 | 1237.925627 | 826.2725461 |
| MA4012 | 11461.65269 | 14145.99014 | 17076.30482 | 22309.58282 | 37273.8024 | 24878.9741 |
| MA4011 | 186.5991712 | 230.3009964 | 278.0074054 | 1756.616237 | 2934.871846 | 1958.925464 |
| MA4010 | 1604.232112 | 1979.945847 | 2390.087825 | 3800.127833 | 6349.074973 | 4237.787982 |
| MA4009a | 18.16620326 | 22.4207572 | 27.06517399 | 107.0377811 | 178.8336937 | 119.3653062 |
| MA4009 | 119.3670129 | 147.3229588 | 177.8406267 | 273.2967919 | 456.6114345 | 304.7723421 |
| MA4008 | 2583.887523 | 3189.038127 | 3849.641248 | 1223.485351 | 2044.141819 | 1364.393974 |
| MA4007 | 1270.246092 | 1567.739765 | 1892.494046 | 7027.748858 | 11741.63248 | 7837.133632 |
| MA4006 | 310.9987274 | 383.8351283 | 463.3458377 | 2294.081985 | 3832.844355 | 2558.29106 |
| MA4005 | 254.4082008 | 313.9910096 | 379.033644 | 669.077188 | 1117.862718 | 746.1347066 |
| MA4004 | 1055.980975 | 1303.293414 | 1573.268141 | 1809.204688 | 3022.734158 | 2017.570519 |
| MA4003 | 811.6981089 | 1001.799108 | 1209.319869 | 2595.149446 | 4335.853719 | 2894.032416 |
| MA4002 | 635.1253358 | 783.8727084 | 946.250434 | 2331.069712 | 3894.641712 | 2599.538659 |
| MA4001a | 553.3894053 | 682.9940919 | 824.4750059 | 2568.055023 | 4290.58563 | 2863.817531 |
| MA4001 | 116.7212033 | 144.0574964 | 173.89873 | 510.2595084 | 852.5176038 | 569.0260189 |
| MA4000 | 305.167341 | 376.6380219 | 454.6578646 | 1805.532822 | 3016.599376 | 2013.475764 |
| MA3999 | 669.8986885 | 826.7900361 | 998.0580038 | 2086.857101 | 3486.62276 | 2327.200118 |
| MA3998 | 3443.416682 | 4249.870393 | 5130.222881 | 20660.23423 | 34518.1483 | 23039.67029 |
| MA3997 | 3211.574137 | 3963.729952 | 4784.808997 | 20319.15994 | 33948.2974 | 22659.31452 |
| MA3996 | 637.4603068 | 786.7545334 | 949.7292235 | 3675.052998 | 6140.105818 | 4098.308297 |
| MA3995 | 621.6305722 | 767.2174495 | 926.1450703 | 4697.227559 | 7847.90703 | 5238.206547 |
| MA3994 | 1743.720895 | 2152.103127 | 2597.907155 | 10183.06475 | 17013.38597 | 11355.84678 |
| MA3993 | 691.7703693 | 853.7840996 | 1030.643836 | 5096.771182 | 8515.445736 | 5683.765548 |
| MA3992 | 646.6919974 | 798.1483008 | 963.4831879 | 4645.007439 | 7760.660108 | 5179.972244 |
| MA3991 | 1533.022745 | 1892.059132 | 2283.995546 | 2099.722463 | 3508.117602 | 2341.547182 |
| MA4674 | 428.850551 | 529.2880381 | 638.9290384 | 760.3265614 | 1270.317883 | 847.8932566 |
| MA3990 | 405.8258073 | 500.8708625 | 604.6252995 | 680.5505857 | 1137.031932 | 758.9294938 |
| MA3989 | 954.6104067 | 1178.181696 | 1422.23977 | 3156.906165 | 5274.410442 | 3520.486571 |
| MA3987 | 1395.184931 | 1721.939481 | 2078.635936 | 14613.45403 | 24415.47214 | 16296.48332 |
| MA3986 | 164.3707095 | 202.8665934 | 244.8900185 | 2947.870334 | 4925.1632 | 3287.376114 |
| MA3985 | 331.3488784 | 408.9513173 | 493.664797 | 2291.151684 | 3827.948546 | 2555.023277 |
| MA3984a | 513.8058478 | 634.1400017 | 765.5008848 | 1450.659513 | 2423.693731 | 1617.73175 |
| MA3984 | 28.46922434 | 35.13676236 | 42.41527518 | 443.4616835 | 740.9149375 | 494.5350985 |
| MA3983 | 33.79884501 | 41.71458873 | 50.35568565 | 781.1790101 | 1305.157174 | 871.1472787 |
| MA3982 | 113.8708897 | 140.5396348 | 169.6521501 | 157.8650753 | 263.7535479 | 176.0463721 |
| MA3981 | 2645.507282 | 3265.089332 | 3941.446315 | 1790.082653 | 2990.785961 | 1996.246202 |
| MA3979 | 807.9416284 | 997.162854 | 1203.723224 | 11328.96926 | 18927.90936 | 12633.72493 |
| MA3978 | 455.8790227 | 562.6466213 | 679.197788 | 6320.661261 | 10560.26376 | 7048.610863 |
| MA3977 | 218.5554266 | 269.7414582 | 325.6178831 | 1649.223107 | 2755.444452 | 1839.163883 |
| MA3976 | 224.2787937 | 276.805247 | 334.1449222 | 1016.861007 | 1698.923577 | 1133.972735 |
| MA3975 | 779.3477119 | 961.8721964 | 1161.122174 | 3902.267488 | 6519.725108 | 4351.691045 |
| MA3974 | 26.02641709 | 32.12184573 | 38.77582437 | 138.9467218 | 232.1456522 | 154.9491948 |
| MA3972 | 5305.831689 | 6548.465983 | 7904.968131 | 8164.394743 | 13640.687 | 9104.686851 |
| MA3971 | 1994.675025 | 2461.831116 | 2971.794702 | 8153.016676 | 13621.67706 | 9091.998374 |
| MA3970 | 359.1558274 | 443.2706984 | 535.093372 | 9566.928532 | 15983.97453 | 10668.75024 |
| MA3969 | 871.591288 | 1075.719366 | 1298.552566 | 15459.3821 | 25828.80901 | 17239.8368 |
| MA3968 | 56.88591679 | 70.20868977 | 84.75228498 | 118.6174214 | 198.1804124 | 132.2785718 |
| MA3967 | 1730.637764 | 2135.955905 | 2578.415067 | 13266.46848 | 22164.99199 | 14794.36565 |
| MA3966 | 378.4464341 | 467.0791961 | 563.8337543 | 1949.490156 | 3257.116523 | 2174.012642 |
| MA3965 | 1730.554324 | 2135.852923 | 2578.290753 | 2849.094473 | 4760.133134 | 3177.224253 |
| MA3963 | 350.66804 | 432.7950576 | 522.4477223 | 341.785574 | 571.0392727 | 381.1489669 |
| MA3962 | 1531.794586 | 1890.543335 | 2282.165755 | 5998.739807 | 10022.41252 | 6689.613764 |
| MA3961 | 711.9896533 | 878.7387724 | 1060.767821 | 2892.360904 | 4832.420653 | 3225.473672 |
| MA3960 | 1163.060013 | 1435.450534 | 1732.801355 | 3327.195254 | 5558.921449 | 3710.387829 |
| MA3959 | 195.8639614 | 241.7356154 | 291.8106838 | 469.8442218 | 784.9936426 | 523.9561099 |
| MA3958 | 329.3468094 | 406.4803606 | 490.6819864 | 1132.88854 | 1892.776926 | 1263.363142 |
| MA3957 | 312.5173854 | 385.7094585 | 465.6084318 | 751.9105886 | 1256.256871 | 838.5080174 |
| MA3956 | 869.9884131 | 1073.741095 | 1296.1645 | 20990.98465 | 35070.75055 | 23408.51318 |
| MA3955 | 86.98705728 | 107.3595657 | 129.5988934 | 312.8447372 | 522.6862828 | 348.8750183 |
| MA3955a | 306.6896422 | 378.5168484 | 456.9258864 | 635.3307749 | 1061.480797 | 708.5017242 |
| MA3954 | 182.6212479 | 225.3914372 | 272.0808404 | 1151.360516 | 1923.639036 | 1283.962533 |
| MA3953 | 61.85925113 | 76.34678699 | 92.16187725 | 312.207229 | 521.6211641 | 348.1640884 |
| MA3952 | 91.28948945 | 112.6696343 | 136.0089326 | 318.8560453 | 532.7296939 | 355.5786479 |
| MA3951 | 116.8365905 | 144.1999075 | 174.0706413 | 660.1617279 | 1102.967187 | 736.1924543 |
| MA3950 | 318.0222593 | 392.5035827 | 473.8099458 | 1535.949179 | 2566.191697 | 1712.844214 |
| MA3949 | 448.6877616 | 553.7711553 | 668.4837862 | 1604.908931 | 2681.406409 | 1789.74605 |
| MA3948 | 181.2029922 | 223.6410237 | 269.9678321 | 523.7958428 | 875.133475 | 584.1213308 |
| MA3947 | 542.5377141 | 669.6009172 | 808.3074608 | 859.1984113 | 1435.508322 | 958.1521625 |
| MA3946 | 67.70061506 | 83.55620772 | 100.8647156 | 112.0442349 | 187.1982415 | 124.9483525 |
| MA3945 | 171.122711 | 211.1999245 | 254.9495831 | 189.7016159 | 316.9445436 | 211.5495223 |
| MA3944 | 1115.864493 | 1377.201747 | 1662.486443 | 998.7375546 | 1668.643764 | 1113.762008 |
| MA3943 | 210.1212036 | 259.3319266 | 313.052037 | 659.3310863 | 1101.57939 | 735.2661479 |
| MA3942 | 190.1619676 | 234.6982056 | 283.315488 | 903.9738751 | 1510.317063 | 1008.08441 |
| MA3941 | 716.6144274 | 884.4466761 | 1067.658106 | 2181.16417 | 3644.186577 | 2432.368517 |
| MA3940 | 642.0292699 | 792.3935549 | 956.5363576 | 1292.266033 | 2159.05735 | 1441.096117 |
| MA3939 | 61.70106 | 76.15154724 | 91.92619395 | 129.5248228 | 216.4039861 | 144.442177 |
| MA3938 | 144.6803744 | 178.5647502 | 215.5540952 | 718.3106849 | 1200.119731 | 801.0384179 |
| MA3937 | 324.0283403 | 399.9162974 | 482.7581902 | 975.4846846 | 1629.793962 | 1087.831108 |
| MA3936 | 460.4608768 | 568.3015529 | 686.0241279 | 1834.337371 | 3064.724663 | 2045.597728 |
| MA3935 | 310.4049752 | 383.1023184 | 462.4612277 | 1013.927069 | 1694.02169 | 1130.700896 |
| MA3934 | 399.0496997 | 492.5077796 | 594.5298199 | 1175.5569 | 1964.065217 | 1310.945611 |
| MA3933 | 666.2946021 | 822.3418669 | 992.6884049 | 3200.079191 | 5346.541904 | 3568.631828 |
| MA3932 | 694.8928898 | 857.6379194 | 1035.295967 | 3122.72259 | 5217.298131 | 3482.366078 |
| MA3930n | 686.1342011 | 846.8279318 | 1022.246711 | 4786.035999 | 7996.283998 | 5337.243042 |
| MA3929a | 9.217239682 | 11.375932 | 13.73243446 | 48.11052613 | 80.3808058 | 53.65140815 |
| MA3929b | 26.79201989 | 33.06675393 | 39.91646849 | 62.24971368 | 104.0038958 | 69.4190038 |
| MA3929 | 82.98505776 | 102.4202914 | 123.636458 | 208.3259462 | 348.0611993 | 232.318814 |
| MA3928 | 13.73568429 | 16.95260359 | 20.46430286 | 70.85047303 | 118.3736403 | 79.0103113 |
| MA3927 | 65.15967292 | 80.42017286 | 97.07905717 | 223.2555098 | 373.0048125 | 248.9678131 |
| MA3926 | 112.8921618 | 139.3316873 | 168.1939786 | 294.2335993 | 491.5916683 | 328.1204384 |
| MA3925 | 237.3182842 | 292.8986071 | 353.5719909 | 630.7670932 | 1053.856013 | 703.412444 |
| MA3924 | 434.277343 | 535.9857934 | 647.0142209 | 855.8044875 | 1429.837914 | 954.3673609 |
| MA3923 | 85.61510727 | 105.6663028 | 127.5548744 | 889.9750855 | 1486.928543 | 992.4733816 |
| MA3922 | 25.88032032 | 31.94153287 | 38.55816004 | 837.8252707 | 1399.799084 | 934.3174805 |
| MA3921 | 185.4434826 | 228.8746438 | 276.2855866 | 920.3051345 | 1537.602563 | 1026.296538 |
| MA3920 | 38.77661367 | 47.85815881 | 57.77188446 | 362.8565584 | 606.243683 | 404.6466933 |
| MA3919 | 37.76292401 | 46.6070614 | 56.26162463 | 227.9543074 | 380.8553426 | 254.2077705 |
| MA3918 | 224.820284 | 277.4735553 | 334.9516692 | 2537.241159 | 4239.103275 | 2829.45484 |
| MA3917 | 498.6703528 | 615.4597494 | 742.9510542 | 6916.287897 | 11555.40874 | 7712.835729 |
| MA3916 | 460.0422255 | 567.7848528 | 685.4003943 | 11329.25871 | 18928.39296 | 12634.04772 |
| MA3915 | 363.2590566 | 448.3349104 | 541.2066258 | 1276.294845 | 2132.37344 | 1423.285529 |
| MA3914 | 417.9677639 | 515.8564847 | 622.7151645 | 926.4173398 | 1547.814548 | 1033.112685 |
| MA3913a | 32.47035635 | 40.0749659 | 48.3764181 | 76.96340987 | 128.5868478 | 85.82727415 |
| MA3912 | 171.8069607 | 212.0444266 | 255.9690222 | 775.692417 | 1295.990432 | 865.0287955 |
| MA3911 | 95.60494721 | 117.9957792 | 142.4383782 | 399.8239904 | 668.007131 | 445.871659 |
| MA3910 | 79.84099721 | 98.53988685 | 118.9522351 | 451.6541869 | 754.6025871 | 503.6711313 |
| MA3909 | 142.9329238 | 176.4080439 | 212.9506313 | 521.7847907 | 871.7735038 | 581.8786662 |
| MA3908 | 97.36994545 | 120.1741429 | 145.0679857 | 362.9726304 | 606.4376108 | 404.7761334 |
| MA3907 | 83.95466351 | 103.6169804 | 125.0810388 | 250.8350982 | 419.0834926 | 279.7237386 |
| MA3906 | 144.703997 | 178.5939053 | 215.5892897 | 204.2882906 | 341.315274 | 227.8161421 |
| MA3905 | 527.2760133 | 650.7649015 | 785.5696007 | 783.2501814 | 1308.617589 | 873.4569864 |
| MA3904 | 162.5887331 | 200.6672752 | 242.2351157 | 227.5416552 | 380.1659028 | 253.7475933 |
| MA3903 | 87.78002469 | 108.3382473 | 130.7803071 | 333.1896068 | 556.6775347 | 371.5630034 |
| MA3902 | 70.64258282 | 87.18718905 | 105.2478478 | 347.9926597 | 581.4097797 | 388.0709217 |
| MA3901 | 204.1159488 | 251.9202315 | 304.1050235 | 946.9329868 | 1582.091127 | 1055.991116 |
| MA3900 | 156.0085139 | 192.5459582 | 232.4314835 | 1014.496602 | 1694.97324 | 1131.336023 |
| MA3899 | 862.9173949 | 1065.014033 | 1285.629644 | 2834.88655 | 4736.395205 | 3161.380006 |
| MA3898 | 385.9650786 | 476.3587192 | 575.0355127 | 1818.273946 | 3037.886648 | 2027.684281 |
| MA3897 | 61.35577807 | 75.72539973 | 91.41177081 | 468.2960322 | 782.407 | 522.2296154 |
| MA3896 | 46.84984653 | 57.82215575 | 69.79990423 | 459.2801879 | 767.3437512 | 512.1754177 |
| MA3895 | 130.7766855 | 161.4047951 | 194.8394884 | 804.9951984 | 1344.948142 | 897.7063737 |
| MA3894 | 523.7583396 | 646.4233831 | 780.328745 | 1446.012611 | 2415.929905 | 1612.549664 |
| MA3893 | 1248.429056 | 1540.813144 | 1859.989627 | 1033.964631 | 1727.499508 | 1153.046182 |
| MA3892 | 781.6083488 | 964.662278 | 1164.490216 | 986.7453771 | 1648.607798 | 1100.388693 |
| MA3891 | 3159.516741 | 3899.480631 | 4707.250553 | 16205.73632 | 27075.78255 | 18072.14852 |
| MA3890 | 2005.578677 | 2475.288421 | 2988.039663 | 8343.456115 | 13939.85432 | 9304.370694 |
| MA3889 | 1519.316811 | 1875.143246 | 2263.575567 | 5247.066336 | 8766.551815 | 5851.370172 |
| MA3888 | 4756.737368 | 5870.772891 | 7086.892217 | 7821.006882 | 13066.9707 | 8721.751062 |
| MA3887 | 3506.750509 | 4328.037105 | 5224.581676 | 20597.63348 | 34413.5579 | 22969.85981 |
| MA3886 | 934.6884134 | 1153.593939 | 1392.558708 | 8012.85952 | 13387.50907 | 8935.69934 |
| MA3885 | 1262.084134 | 1557.666263 | 1880.333837 | 10048.41403 | 16788.41788 | 11205.68836 |
| MA3884 | 2092.395348 | 2582.437696 | 3117.384704 | 14386.35967 | 24036.05357 | 16043.23454 |
| MA3883 | 486.6911051 | 600.6749428 | 725.1036032 | 2172.261073 | 3629.311701 | 2422.440052 |
| MA3882 | 233.290991 | 287.9281154 | 347.5718714 | 1431.736879 | 2392.078685 | 1596.629799 |
| MA3881 | 167.1666389 | 206.3173339 | 249.0555733 | 1982.903649 | 3312.942218 | 2211.274362 |
| MA3880 | 240.3085852 | 296.5892414 | 358.0271329 | 5463.44585 | 9128.068538 | 6092.670083 |
| MA3879 | 7809.908325 | 9639.001385 | 11635.702 | 25060.22638 | 41869.44838 | 27946.40887 |
| MA3878 | 619.4963011 | 764.5833287 | 922.9652964 | 1408.076808 | 2352.548549 | 1570.244801 |
| MA3877 | 1450.583885 | 1790.312958 | 2161.172848 | 3136.195506 | 5239.808047 | 3497.390669 |
| MA3876 | 4927.744054 | 6081.829617 | 7341.668938 | 23226.40556 | 38805.58674 | 25901.38718 |
| MA3875 | 158.2319457 | 195.2901213 | 235.7440947 | 690.2288481 | 1153.201918 | 769.7224002 |
| MA3874 | 3461.32295 | 4271.970338 | 5156.900787 | 25187.26559 | 42081.69951 | 28088.07916 |
| MA3873 | 6222.025822 | 7679.234252 | 9269.972875 | 18219.08948 | 30439.59839 | 20317.37927 |
| MA4682 | 1288.294898 | 1590.015631 | 1919.384314 | 8027.247623 | 13411.54804 | 8951.744518 |
| MA3871 | 1728.887466 | 2133.795684 | 2575.807361 | 6631.878333 | 11080.23061 | 7395.670758 |
| MA3870 | 1366.060492 | 1685.994051 | 2035.24448 | 7175.89897 | 11989.15472 | 8002.34617 |
| MA3869 | 2301.419831 | 2840.416048 | 3428.802776 | 11721.71445 | 19584.08956 | 13071.70253 |
| MA3868 | 159.8435416 | 197.2791555 | 238.1451536 | 2007.46028 | 3353.970283 | 2238.659177 |
| MA3866 | 42.08665242 | 51.94341394 | 62.70339234 | 149.9531633 | 250.5346972 | 167.2232464 |
| MA3865 | 38584.48282 | 47621.0306 | 57485.6356 | 52737.49964 | 88111.33567 | 58811.2695 |
| MA3864 | 30847.41365 | 38071.92742 | 45958.45405 | 46384.67923 | 77497.34191 | 51726.79572 |
| MA3863 | 44072.67267 | 54394.56332 | 65662.29261 | 78629.24713 | 131370.0504 | 87684.96564 |
| MA3862 | 50743.93798 | 62628.24967 | 75601.57127 | 77352.97176 | 129237.7095 | 86261.70182 |
| MA3861 | 19532.73281 | 24107.33017 | 29101.1173 | 26552.99132 | 44363.48988 | 29611.09532 |
| MA3860 | 74169.15449 | 91539.68947 | 110501.9603 | 98091.8422 | 163887.2395 | 109389.0648 |
| MA3859 | 316.21242 | 390.269876 | 471.1135311 | 1263.345021 | 2110.737483 | 1408.844277 |
| MA3858 | 219.1812337 | 270.5138303 | 326.5502506 | 1253.906936 | 2094.968773 | 1398.319208 |
| MA3857 | 1917.2378 | 2366.257969 | 2856.423761 | 5747.54583 | 9602.729431 | 6409.489814 |
| MA3856 | 432.3535761 | 533.6114773 | 644.1480697 | 4154.040552 | 6940.375708 | 4632.460774 |
| MA3855 | 109.8207126 | 135.5408997 | 163.6179367 | 1797.807274 | 3003.691894 | 2004.860466 |
| MA3854 | 1099.81107 | 1357.388587 | 1638.569025 | 7497.776024 | 12526.93178 | 8361.293755 |
| MA3853 | 2842.871619 | 3508.676713 | 4235.492354 | 17823.84626 | 29779.24458 | 19876.61594 |
| MA3852 | 29.29817545 | 36.15985514 | 43.65029967 | 189.5145524 | 316.6320066 | 211.3409147 |
| MA3851 | 1125.296569 | 1388.842831 | 1676.538955 | 8530.212828 | 14251.87866 | 9512.636151 |
| MA3850 | 2673.953812 | 3300.198085 | 3983.827778 | 18378.02975 | 30705.14831 | 20494.62466 |
| MA3849 | 1368.594011 | 1689.120924 | 2039.019078 | 6272.194978 | 10479.28857 | 6994.562725 |
| MA3847n | 392.4440305 | 484.355052 | 584.6882706 | 2025.916787 | 3384.806548 | 2259.241317 |
| MA3846 | 307.6829943 | 379.7428451 | 458.405846 | 1586.697392 | 2650.979426 | 1769.437091 |
| MA3845 | 62.18345402 | 76.74691872 | 92.6448955 | 141.7766918 | 236.873833 | 158.105092 |
| MA3844 | 112.3586449 | 138.6732198 | 167.3991109 | 1382.279992 | 2309.448442 | 1541.47697 |
| MA3843 | 14.22366606 | 17.55487147 | 21.19132938 | 56.30401217 | 94.07009718 | 62.78853674 |
| MA3842 | 456.3825012 | 563.2680152 | 679.9479025 | 1830.366096 | 3058.089644 | 2041.169083 |
| MA3841 | 37.56831814 | 46.36687852 | 55.97168833 | 56.59901934 | 94.56298129 | 63.11751984 |
| MA3840 | 168.1267674 | 207.502326 | 250.4860343 | 941.3131562 | 1572.701778 | 1049.72405 |
| MA3839 | 338.4662322 | 417.7355668 | 504.268687 | 3749.012273 | 6263.673498 | 4180.78545 |
| MA3838 | 1253.916744 | 1547.586057 | 1868.165536 | 308.2948798 | 515.0845949 | 343.8011545 |
| MA3837 | 219.6673978 | 271.1138547 | 327.2745689 | 30.52781057 | 51.00443105 | 34.04369389 |
| MA3836 | 467.0134347 | 576.3887304 | 695.7865486 | 184.1951 | 307.7445156 | 205.4088218 |
| MA3835 | 345.2199671 | 426.0710372 | 514.330834 | 132.3687366 | 221.1554636 | 147.613624 |
| MA3834 | 406.235648 | 501.3766885 | 605.2359063 | 238.3298095 | 398.1902439 | 265.7782176 |
| MA3833 | 234.4595805 | 289.3703904 | 349.3129109 | 189.455813 | 316.5338677 | 211.2754104 |
| MA3832 | 79.03925765 | 97.5503786 | 117.7577521 | 110.3576593 | 184.3803902 | 123.0675342 |
| MA3831 | 32.58254752 | 40.21343242 | 48.54356769 | 42.51940023 | 71.03941544 | 47.41635313 |
| MA3830 | 53.44052047 | 65.9563761 | 79.6191127 | 76.94085806 | 128.5491693 | 85.80212506 |
| MA3829 | 928.1664166 | 1145.54448 | 1382.841819 | 2573.986361 | 4300.495432 | 2870.431981 |
| MA3828 | 15.96534851 | 19.70445875 | 23.78619953 | 120.1967749 | 200.8191222 | 134.0398193 |
| MA3827 | 13.36527055 | 16.49543835 | 19.91243672 | 109.7688148 | 183.3965765 | 122.4108726 |
| MA3826 | 621.3577218 | 766.8806971 | 925.7385603 | 2175.848592 | 3635.305559 | 2426.440745 |
| MA3825 | 21.57305904 | 26.62550408 | 32.1409261 | 158.1188336 | 264.1775154 | 176.3293558 |
| MA3824 | 91.17173321 | 112.5242994 | 135.8334919 | 698.243904 | 1166.593096 | 778.6605489 |
| MA3823 | 148.3665575 | 183.1142433 | 221.046007 | 744.841953 | 1244.446926 | 830.6252881 |
| MA3822 | 942.2196356 | 1162.888986 | 1403.779205 | 256.1370814 | 427.9417968 | 285.6363503 |
| MA3821 | 49.81139775 | 61.47730702 | 74.21221307 | 118.8360973 | 198.5457659 | 132.5224326 |
| MA3819 | 43.54116817 | 53.73857961 | 64.87042313 | 108.1946738 | 180.7665757 | 120.655438 |
| MA3818 | 87.21988594 | 107.6469232 | 129.9457765 | 293.2793271 | 489.9973152 | 327.0562629 |
| MA3817 | 99.74386029 | 123.1040324 | 148.6047962 | 282.2371972 | 471.5486435 | 314.7424125 |
| MA3816 | 158.9885166 | 196.2238822 | 236.8712825 | 474.3295645 | 792.4875422 | 528.9580287 |
| MA3815 | 21.99119042 | 27.14156249 | 32.76388502 | 81.49576266 | 136.1592898 | 90.88161732 |
| MA3814 | 62.14194354 | 76.69568642 | 92.58305052 | 190.6121595 | 318.465837 | 212.5649331 |
| MA3813 | 35.28500776 | 43.54881319 | 52.56986618 | 151.6038546 | 253.2925947 | 169.0640476 |
| MA3812 | 47.22828391 | 58.28922377 | 70.3637245 | 230.5625463 | 385.2130657 | 257.1163999 |
| MA3811 | 8.400153095 | 10.36748243 | 12.5150865 | 90.06999945 | 150.4847217 | 100.4433477 |
| MA3810 | 18.40922446 | 22.72069437 | 27.42724256 | 49.15510439 | 82.12603807 | 54.81629034 |
| MA3809 | 10.79704087 | 13.32572517 | 16.08612354 | 45.76072233 | 76.45486405 | 51.0309778 |
| MA3808 | 83.07012836 | 102.5252857 | 123.7632017 | 373.2761075 | 623.652176 | 416.2662604 |
| MA3807 | 23.750818 | 29.31329769 | 35.38549099 | 89.74177622 | 149.9363417 | 100.077323 |
| MA3806 | 19.15265398 | 23.63823627 | 28.53485151 | 90.96884403 | 151.986469 | 101.445712 |
| MA3805 | 87.39003891 | 107.8569262 | 130.1992813 | 217.0436108 | 362.6262636 | 242.0404907 |
| MA3803 | 8.810302438 | 10.8736894 | 13.12615327 | 42.99509883 | 71.8341903 | 47.94683786 |
| MA3802 | 50.00378134 | 61.71474716 | 74.49883847 | 370.2432614 | 618.5850392 | 412.8841218 |
| MA3801 | 57.39704393 | 70.8395237 | 85.51379494 | 121.2427693 | 202.5667204 | 135.2062807 |
| MA3800 | 289.6764218 | 357.5191045 | 431.5785002 | 2098.959377 | 3506.842673 | 2340.696211 |
| MA3797 | 75.68371143 | 93.40895808 | 112.7584443 | 226.2152523 | 377.9498111 | 252.2684287 |
| MA3796 | 73.61407723 | 90.85461222 | 109.6749706 | 153.3355408 | 256.1858146 | 170.9951719 |
| MA3795 | 44.84898946 | 55.35269474 | 66.81889912 | 95.82763126 | 160.1043023 | 106.8640851 |
| MA3794 | 179.6459236 | 221.7192871 | 267.6480115 | 992.5886523 | 1658.370467 | 1106.904937 |
| MA3793 | 8934.177052 | 11026.57565 | 13310.70961 | 53912.6363 | 90074.69877 | 60121.74648 |
| MA3792 | 11998.10869 | 14808.085 | 17875.55136 | 68763.35562 | 114886.5826 | 76682.8209 |
| MA3791 | 4706.343654 | 5808.576888 | 7011.812432 | 25471.24797 | 42556.16392 | 28404.76775 |
| MA3790 | 3132.844867 | 3866.562161 | 4667.513085 | 25787.74513 | 43084.95251 | 28757.71584 |
| MA3789 | 300.6701508 | 371.0875825 | 447.9576623 | 1808.347876 | 3021.302636 | 2016.615027 |
| MA3788 | 124.5562683 | 153.7275461 | 185.571912 | 587.7620885 | 982.0052719 | 655.4545594 |
| MA3787 | 1531.024456 | 1889.59284 | 2281.018367 | 9090.263895 | 15187.58566 | 10137.18821 |
| MA3786 | 1618.183756 | 1997.164988 | 2410.873878 | 10482.58318 | 17513.80727 | 11689.86069 |
| MA3785 | 4902.209133 | 6050.314377 | 7303.625377 | 4337.400156 | 7246.724317 | 4836.937876 |
| MA3784 | 219.4935811 | 270.89933 | 327.0156058 | 2342.78677 | 3914.218022 | 2612.605168 |
| MA3783 | 267.0988162 | 329.6537874 | 397.9409362 | 2112.824216 | 3530.007395 | 2356.157862 |
| MA3782 | 649.3191681 | 801.390759 | 967.3973152 | 995.4932167 | 1663.223277 | 1110.14402 |
| MA3781 | 1216.068608 | 1500.873827 | 1811.776957 | 7501.075426 | 12532.44426 | 8364.973149 |
| MA3780 | 166.882572 | 205.9667381 | 248.6323522 | 1625.04537 | 2715.049425 | 1812.2016 |
| MA3779 | 525.1763311 | 648.1734705 | 782.4413597 | 1623.620332 | 2712.668538 | 1810.61244 |
| MA3778 | 286.2095837 | 353.2403274 | 426.4133827 | 1196.544954 | 1999.131069 | 1334.350855 |
| MA3777 | 355.0331434 | 438.1824751 | 528.9511331 | 1763.273493 | 2945.994477 | 1966.349435 |
| MA3776 | 169.1391406 | 208.7517987 | 251.9943328 | 839.0873776 | 1401.907752 | 935.7249441 |
| MA3773n | 611.1692152 | 754.3060259 | 910.5590701 | 1538.666113 | 2570.731023 | 1715.874057 |
| MA3771n | 278.2113734 | 343.3689234 | 414.4971362 | 2045.684489 | 3417.833494 | 2281.285662 |
| MA3769b | 84.78925269 | 104.6470317 | 126.3244633 | 574.8904754 | 960.4999857 | 641.1005245 |
| MA3769a | 137.7598869 | 170.0234734 | 205.2435093 | 537.9471579 | 898.7768272 | 599.9024508 |
| MA3768 | 549.4867397 | 678.1774157 | 818.6605646 | 1488.417145 | 2486.777409 | 1659.837923 |
| MA3767 | 779.841235 | 962.4813034 | 1161.857456 | 3513.271061 | 5869.808161 | 3917.893958 |
| MA3766 | 1053.244823 | 1299.91645 | 1569.191645 | 6820.534058 | 11395.42773 | 7606.053934 |
| MA3765 | 1166.925549 | 1440.221384 | 1738.560477 | 5937.109627 | 9919.443713 | 6620.885645 |
| MA3764 | 359.1737522 | 443.2928212 | 535.1200776 | 2441.477399 | 4079.105686 | 2722.661982 |
| MA3763 | 221.9988006 | 273.9912759 | 330.7480423 | 1901.087232 | 3176.247194 | 2120.035161 |
| MA3762 | 6.150484375 | 7.590937683 | 9.163385841 | 119.7022107 | 199.9928274 | 133.4882962 |
| MA3759 | 72.44013972 | 89.40573667 | 107.9259633 | 77.09402427 | 128.8050722 | 85.9729314 |
| MA3758 | 718.4685467 | 886.7350331 | 1070.420492 | 2188.330497 | 3656.15974 | 2440.36019 |
| MA3757 | 979.5373881 | 1208.946616 | 1459.377585 | 4554.512464 | 7609.465358 | 5079.054977 |
| MA3756 | 1228.082594 | 1515.701508 | 1829.676164 | 5034.759598 | 8411.839697 | 5614.6121 |
| MA3755 | 738.8676969 | 911.9116969 | 1100.812453 | 2722.648534 | 4548.873202 | 3036.215554 |
| MA3754 | 423.6247332 | 522.8383254 | 631.14328 | 1679.207769 | 2805.541415 | 1872.601873 |
| MA3753 | 567.3834258 | 700.2655344 | 845.3241946 | 1019.613933 | 1703.523037 | 1137.042716 |
| MA3752 | 2529.026045 | 3121.328002 | 3767.905101 | 9028.179683 | 15083.85828 | 10067.95377 |
| MA3751 | 177.053126 | 218.5192522 | 263.7850954 | 1856.549752 | 3101.836065 | 2070.368306 |
| MA3750 | 174.9552562 | 215.9300578 | 260.6595545 | 1354.201094 | 2262.535539 | 1510.164229 |
| MA3749 | 86.4212882 | 106.6612925 | 128.7559744 | 1161.478895 | 1940.544346 | 1295.246243 |
| MA3748 | 332.5898502 | 410.4829267 | 495.5136764 | 2813.218163 | 4700.192682 | 3137.216074 |
| MA3747 | 70.06675868 | 86.47650597 | 104.3899481 | 220.1506898 | 367.8174252 | 245.5054115 |
| MA3744n | 3519.927865 | 4344.300618 | 5244.214145 | 11754.20878 | 19638.37957 | 13107.93923 |
| MA3743 | 2641.715649 | 3260.409692 | 3935.797296 | 2822.573505 | 4715.823148 | 3147.648869 |
| MA3742 | 2136.653191 | 2637.060796 | 3183.322874 | 3474.18968 | 5804.512826 | 3874.311581 |
| MA3741 | 1969.833491 | 2431.171655 | 2934.784193 | 2797.901112 | 4674.601673 | 3120.13496 |
| MA3740 | 2818.971214 | 3479.178795 | 4199.883998 | 3386.145 | 5657.411913 | 3776.126808 |
| MA3739 | 5343.447335 | 6594.891274 | 7961.010332 | 5234.823636 | 8746.097287 | 5837.717482 |
| MA3738 | 541.8800729 | 668.7892554 | 807.3276648 | 848.8270763 | 1418.180383 | 946.5863624 |
| MA3737 | 7517.063546 | 9277.571889 | 11199.40308 | 14998.90561 | 25059.46653 | 16726.32731 |
| MA3736 | 6212.585929 | 7667.583521 | 9255.908717 | 14992.84646 | 25049.34318 | 16719.57032 |
| MA3735 | 1556.779757 | 1921.380073 | 2319.390264 | 3165.844051 | 5289.343442 | 3530.453832 |
| MA3734 | 1984.405463 | 2449.156407 | 2956.494451 | 4535.998185 | 7578.53257 | 5058.40841 |
| MA4664 | 1200.607585 | 1481.791808 | 1788.742134 | 2843.801228 | 4751.289429 | 3171.321385 |
| MA3733 | 8612.838215 | 10629.97874 | 12831.95842 | 81190.30543 | 135648.9463 | 90540.98063 |
| MA3732 | 5869.502951 | 7244.149959 | 8744.761706 | 65906.7851 | 110113.9589 | 73497.25958 |
| MA3731 | 628.1898462 | 775.312916 | 935.9174971 | 6779.48521 | 11326.84524 | 7560.277497 |
| MA3730 | 457.5281111 | 564.6819287 | 681.6547054 | 1851.597121 | 3093.561443 | 2064.845282 |
| MA3729 | 87.83246251 | 108.4029661 | 130.8584323 | 64.50357055 | 107.7695339 | 71.93243702 |
| MA3728 | 176.6991981 | 218.0824338 | 263.257791 | 271.5141737 | 453.63312 | 302.7844201 |
| MA3727 | 263.9828911 | 325.8081077 | 393.2986313 | 374.4671544 | 625.6421211 | 417.5944799 |
| MA3726 | 2918.740161 | 3602.31379 | 4348.526171 | 2128.347888 | 3555.943616 | 2373.469392 |
| MA3725 | 298.5073549 | 368.4182563 | 444.7353903 | 288.5761174 | 482.1394135 | 321.8113852 |
| MA3724 | 5.438676074 | 6.712422736 | 8.102888211 | 89.2705917 | 149.1491088 | 99.55187225 |
| MA3723 | 2737.424486 | 3378.533692 | 4078.390456 | 13046.23477 | 21797.03586 | 14548.76765 |
| MA3722 | 64.42746498 | 79.51648064 | 95.98816685 | 1281.978808 | 2141.869937 | 1429.624113 |
| MA3720 | 435.2746339 | 537.2166515 | 648.5000488 | 1255.059139 | 2096.89382 | 1399.60411 |
| MA3719 | 93.17642049 | 114.9984877 | 138.8202035 | 429.6184671 | 717.7863422 | 479.097561 |
| MA3718 | 179.9350649 | 222.0761458 | 268.0787928 | 273.1366551 | 456.3438855 | 304.5937623 |
| MA3717 | 1069.575342 | 1320.071604 | 1593.5219 | 1635.420283 | 2732.383342 | 1823.77139 |
| MA3716 | 323.5477849 | 399.3231953 | 482.042228 | 581.1124639 | 970.8953917 | 648.0390986 |
| MA3715 | 392.4257485 | 484.3324884 | 584.6610329 | 1144.502612 | 1912.181171 | 1276.314805 |
| MA3714 | 303.6672547 | 374.7866129 | 452.4229398 | 841.0351136 | 1405.161938 | 937.8970005 |
| MA3713 | 195.7441367 | 241.5877275 | 291.6321612 | 539.9099609 | 902.0561863 | 602.0913095 |
| MA3712 | 15.09536596 | 18.63072488 | 22.49004375 | 50.21633954 | 83.89909987 | 55.99974778 |
| MA3711a | 17.90459744 | 22.09788289 | 26.67541688 | 53.59136057 | 89.53792639 | 59.76346947 |
| MA3711 | 547.635789 | 675.8929694 | 815.9028995 | 4566.085648 | 7628.801289 | 5091.961042 |
| MA3710 | 647.6407127 | 799.3192067 | 964.8966448 | 1147.478476 | 1917.153105 | 1279.633399 |
| MA3709 | 455.6876755 | 562.4104602 | 678.9127065 | 571.9223855 | 955.5410407 | 637.7906002 |
| MA3708 | 39.34085204 | 48.5545427 | 58.61252294 | 94.23339221 | 157.4407226 | 105.0862378 |
| MA3707 | 64.27618331 | 79.32976857 | 95.76277773 | 471.0052888 | 786.9334987 | 525.2508968 |
| MA3706 | 306.263836 | 377.9913177 | 456.291493 | 2830.891752 | 4729.720884 | 3156.925127 |
| MA3705 | 904.6648857 | 1116.538853 | 1347.827732 | 6015.30654 | 10050.09144 | 6708.088485 |
| MA3704 | 844.7199947 | 1042.554773 | 1258.517991 | 1897.766794 | 3170.699563 | 2116.332309 |
| MA3703 | 312.8336182 | 386.0997535 | 466.0795758 | 1748.840621 | 2921.880712 | 1950.254331 |
| MA3702 | 242.4407308 | 299.220739 | 361.2037402 | 1226.358605 | 2048.942318 | 1367.598141 |
| MA3701 | 29.96278675 | 36.98011948 | 44.64048018 | 75.45663762 | 126.0694036 | 84.14696717 |
| MA3700 | 3314.638355 | 4090.931976 | 4938.360675 | 11508.38242 | 19227.66444 | 12833.80109 |
| MA3699 | 2672.316275 | 3298.177034 | 3981.38807 | 8399.371529 | 14033.27517 | 9366.725878 |
| MA3698 | 1115.349151 | 1376.565712 | 1661.718655 | 3172.88006 | 5301.098876 | 3538.300177 |
| MA3697 | 99.06014625 | 122.2601914 | 147.5861552 | 833.6343052 | 1392.797015 | 929.6438421 |
| MA3696 | 60.56979973 | 74.755344 | 90.2407699 | 762.9035666 | 1274.623422 | 850.7670551 |
| MA3695 | 665.3582061 | 821.1861655 | 991.2933021 | 9357.478177 | 15634.03472 | 10435.17752 |
| MA3694 | 1493.227224 | 1842.943435 | 2224.705627 | 19709.16903 | 32929.15327 | 21979.07105 |
| MA3693 | 254.4784919 | 314.077763 | 379.1383683 | 2211.286851 | 3694.514137 | 2465.960423 |
| MA3692 | 1441.445242 | 1779.034031 | 2147.557513 | 15737.94507 | 26294.21892 | 17550.48182 |
| MA3691 | 479.1002979 | 591.3063564 | 713.7943321 | 4304.450439 | 7191.673478 | 4800.193345 |
| MA3690 | 3991.515298 | 4926.334585 | 5946.81533 | 35180.99427 | 58778.75167 | 39232.78406 |
| MA3689 | 1017.116598 | 1255.326937 | 1515.3655 | 10562.95687 | 17648.09186 | 11779.49101 |
| MA3688 | 555.9207491 | 686.1182805 | 828.2463642 | 2610.155148 | 4360.924541 | 2910.766322 |
| MA3687 | 372.3138464 | 459.5103466 | 554.6970321 | 3605.045362 | 6023.14035 | 4020.237892 |
| MA3686 | 758.8377827 | 936.5588088 | 1130.565167 | 2328.190078 | 3889.830554 | 2596.327377 |
| MA3685 | 2047.117969 | 2526.556282 | 3049.927562 | 10181.77253 | 17011.22699 | 11354.40573 |
| MA3684 | 3205.65016 | 3956.418571 | 4775.983077 | 11770.08485 | 19664.90456 | 13125.64375 |
| MA3683 | 370.0179728 | 456.6767756 | 551.2764924 | 2673.131355 | 4466.142227 | 2980.995489 |
| MA3682 | 7868.986375 | 9711.915608 | 11723.72027 | 20003.23817 | 33420.47016 | 22307.00809 |
| MA3681 | 2375.616208 | 2931.989336 | 3539.345294 | 5950.875638 | 9942.443317 | 6636.237085 |
| MA3680 | 626.0369523 | 772.6558109 | 932.7099778 | 643.4523069 | 1075.049871 | 717.5586118 |
| MA3679 | 5520.267939 | 6813.123547 | 8224.449001 | 3499.252433 | 5846.386495 | 3902.260806 |
| MA3678 | 2988.426232 | 3688.32045 | 4452.348946 | 321.6954024 | 537.4735582 | 358.7450133 |
| MA3677 | 69.91942819 | 86.29467045 | 104.1704457 | 393.0908145 | 656.7576571 | 438.3630242 |
| MA3676 | 116.3372823 | 143.5836605 | 173.3267399 | 2017.823727 | 3371.285043 | 2250.216181 |
| MA3675 | 32.03420987 | 39.53667321 | 47.72661911 | 168.2409486 | 281.0890694 | 187.6172332 |
| MA3674 | 253.4025053 | 312.7497786 | 377.5352945 | 1256.430544 | 2099.1851 | 1401.13346 |
| MA3673 | 757.1770789 | 934.5091656 | 1128.090944 | 2030.861916 | 3393.068636 | 2264.755976 |
| MA3672 | 180.3780785 | 222.6229138 | 268.7388228 | 335.0301772 | 559.7526732 | 373.615552 |
| MA3671 | 130.2109566 | 160.7065715 | 193.996629 | 327.0402875 | 546.4035411 | 364.705468 |
| MA3670 | 96.18126546 | 118.7070721 | 143.2970141 | 414.2830094 | 692.1645803 | 461.995921 |
| MA3669 | 28.51519741 | 35.19350239 | 42.4837688 | 101.8286185 | 170.1304698 | 113.5562051 |
| MA3668 | 658.0945989 | 812.2214098 | 980.4715145 | 1017.864139 | 1700.599562 | 1135.091398 |
| MA3667 | 565.5099516 | 697.9532895 | 842.5329726 | 1161.189813 | 1940.061363 | 1294.923869 |
| MA3666 | 200.1667072 | 247.0460711 | 298.2211901 | 456.1683901 | 762.1447057 | 508.7052347 |
| MA3665 | 111.9263985 | 138.1397406 | 166.7551225 | 550.0685222 | 919.0286329 | 613.4198307 |
| MA3664 | 3503.262205 | 4323.731833 | 5219.384577 | 9908.8442 | 16555.23116 | 11050.04429 |
| MA3662 | 139.5714136 | 172.2592626 | 207.942438 | 566.2865476 | 946.1249475 | 631.5056836 |
| MA3661 | 124.8492191 | 154.0891065 | 186.0083689 | 461.7404168 | 771.454186 | 514.9189908 |
| MA3660 | 184.6778469 | 227.9296949 | 275.1448934 | 708.0539312 | 1182.983229 | 789.6003955 |
| MA3659 | 60.80223087 | 75.04221089 | 90.58706072 | 253.5805025 | 423.6703849 | 282.7853306 |
| MA3658 | 287.3334201 | 354.627368 | 428.087746 | 1987.615183 | 3320.814027 | 2216.528523 |
| MA3657 | 2293.440526 | 2830.567976 | 3416.914695 | 4483.194875 | 7490.311281 | 4999.523751 |
| MA3656 | 58.39999912 | 72.07737261 | 87.00806188 | 143.6201688 | 239.9538277 | 160.1608819 |
| MA3655 | 154.304189 | 190.4424776 | 229.892271 | 365.4895027 | 610.6426827 | 407.5828735 |
| MA3654 | 31.71541397 | 39.1432148 | 47.25165655 | 109.412906 | 182.8019408 | 122.0139738 |
| MA3653 | 66.75785773 | 82.39265511 | 99.46013533 | 60.46977463 | 101.0300573 | 67.43406944 |
| MA3652 | 167.595112 | 206.8461562 | 249.69394 | 172.1787826 | 287.6682174 | 192.008587 |
| MA3651 | 1488.860913 | 1837.554527 | 2218.200418 | 652.2196205 | 1089.697887 | 727.3356556 |
| MA3650 | 127.9127386 | 157.8701072 | 190.5725966 | 605.7153069 | 1012.000665 | 675.4754472 |
| MA3649 | 59.34165745 | 73.23956882 | 88.41100481 | 341.0726653 | 569.8481784 | 380.3539527 |
| MA3647n | 81.47812426 | 100.5604317 | 121.3913319 | 138.2940745 | 231.0552397 | 154.2213821 |
| MA3646 | 55.32862289 | 68.28667514 | 82.43212871 | 175.2839544 | 292.8561924 | 195.4713809 |
| MA3645 | 285.8010182 | 352.7360752 | 425.8046755 | 1322.742506 | 2209.976008 | 1475.08256 |
| MA3644 | 220.52003 | 272.1661748 | 328.544875 | 580.8071369 | 970.3852657 | 647.6986071 |
| MA3643 | 1609.579672 | 1986.545813 | 2398.054962 | 2398.04413 | 4006.539421 | 2674.22651 |
| MA3642 | 184.9265078 | 228.2365926 | 275.5153644 | 751.9888909 | 1256.387694 | 838.5953378 |
| MA3641 | 105.319803 | 129.9858698 | 156.912193 | 608.7360864 | 1017.047642 | 678.8441294 |
| MA3640 | 104.4848229 | 128.955336 | 155.6681861 | 852.6280018 | 1424.530791 | 950.82504 |
| MA3639 | 103.02455 | 127.1530648 | 153.4925779 | 375.7517548 | 627.7883712 | 419.0270277 |
| MA3638 | 307.4954238 | 379.5113454 | 458.1263915 | 826.5910523 | 1381.029479 | 921.7894188 |
| MA3637 | 6688.44782 | 8254.89303 | 9964.878262 | 1619.484046 | 2705.757826 | 1805.999779 |
| MA3636 | 459.8493527 | 567.5468088 | 685.11304 | 1993.621474 | 3330.849056 | 2223.226558 |
| MA3635 | 421.3858898 | 520.0751412 | 627.8077076 | 1848.913294 | 3089.07743 | 2061.852358 |
| MA3634 | 194.9258952 | 240.5778526 | 290.4130925 | 219.6316953 | 366.9503136 | 244.9266445 |
| MA3633 | 625.6478613 | 772.1755941 | 932.130285 | 754.9057702 | 1261.261079 | 841.8481537 |
| MA3632a | 90.32393255 | 111.4779425 | 134.5703841 | 492.2228808 | 822.3828542 | 548.9121156 |
| MA3632 | 539.1578802 | 665.4295207 | 803.2719676 | 6612.448839 | 11047.76873 | 7374.003572 |
| MA3631 | 226.3783261 | 279.3964932 | 337.27294 | 4394.72868 | 7342.506121 | 4900.868918 |
| MA3630 | 513.5584629 | 633.8346788 | 765.1323149 | 11587.79571 | 19360.34443 | 12922.36039 |
| MA3629 | 34.56100621 | 42.65524931 | 51.49120226 | 459.8541054 | 768.3026256 | 512.8154332 |
| MA3628 | 949.3738754 | 1171.71876 | 1414.43805 | 5007.910633 | 8366.981707 | 5584.670943 |
| MA3627 | 1023.68359 | 1263.431929 | 1525.149426 | 4116.750948 | 6878.073991 | 4590.876532 |
| MA3626 | 997.9257671 | 1231.641583 | 1486.773771 | 3809.432954 | 6364.621532 | 4248.164772 |
| MA3625 | 100.0959881 | 123.5386292 | 149.1294189 | 706.0553947 | 1179.644168 | 787.3716878 |
| MA3624 | 336.0394215 | 414.7403932 | 500.6530689 | 1142.850217 | 1909.420428 | 1274.472104 |
| MA3623 | 111.5663248 | 137.6953371 | 166.2186616 | 686.378754 | 1146.769362 | 765.4288914 |
| MA3622 | 339.6248179 | 419.1654951 | 505.9948221 | 2084.525116 | 3482.726588 | 2324.599558 |
| MA3621 | 493.5026836 | 609.0818039 | 735.2519295 | 554.547939 | 926.512632 | 618.4151413 |
| MA3620 | 179.9978238 | 222.1536029 | 268.172295 | 314.2551698 | 525.0427673 | 350.4478902 |
| MA3619 | 1575.714989 | 1944.749968 | 2347.601188 | 2324.795554 | 3884.159143 | 2592.541906 |
| MA3618 | 96.35842336 | 118.9257207 | 143.5609553 | 58.44539233 | 97.64781449 | 65.17653933 |
| MA3617 | 112.4050479 | 138.7304904 | 167.468245 | 879.3701022 | 1469.210235 | 980.647024 |
| MA3616 | 130.0330492 | 160.486998 | 193.7315713 | 178.1909245 | 297.7130215 | 198.7131464 |
| MA3615 | 47.78299719 | 58.97385179 | 71.19017189 | 151.2736759 | 252.7409477 | 168.6958423 |
| MA3614 | 233.7691851 | 288.5183033 | 348.2843156 | 468.2068204 | 782.2579492 | 522.1301291 |
| MA3613 | 221.6464286 | 273.5563778 | 330.2230559 | 448.5997309 | 749.4993457 | 500.2648941 |
| MA3612 | 1830.623944 | 2259.359009 | 2727.380887 | 2955.382795 | 4937.714667 | 3295.753785 |
| MA3611 | 471.5076048 | 581.9354423 | 702.4822512 | 315.9750483 | 527.9162595 | 352.3658469 |
| MA3610 | 641.0699749 | 791.2095915 | 955.1071384 | 508.6689979 | 849.8602535 | 567.2523295 |
| MA3609 | 616.1534583 | 760.4575867 | 917.9849149 | 4756.085783 | 7946.244584 | 5303.843464 |
| MA3608 | 202.9614714 | 250.4953736 | 302.3850091 | 2129.702637 | 3558.207068 | 2374.980168 |
| MA3607 | 30979.1687 | 38234.53971 | 46154.75115 | 22878.74717 | 38224.73544 | 25513.68903 |
| MA3606 | 34483.42028 | 42559.49264 | 51375.60978 | 19576.45409 | 32707.41937 | 21831.07134 |
| MA3604 | 1210.747772 | 1494.306843 | 1803.849635 | 1950.818764 | 3259.3363 | 2175.494266 |
| MA3603 | 189.2953658 | 233.6286442 | 282.0243691 | 509.775429 | 851.7088268 | 568.4861882 |
| MA3602 | 419.3957628 | 517.6189232 | 624.8426888 | 770.1379842 | 1286.710346 | 858.8346594 |
| MA3601 | 154.0425955 | 190.1196185 | 229.5025323 | 629.0418821 | 1050.973611 | 701.4885406 |
| MA3600 | 157.9363299 | 194.9252717 | 235.3036673 | 811.4332115 | 1355.704471 | 904.8858518 |
| MA3599 | 307.3136972 | 379.2870582 | 457.8556436 | 1070.628192 | 1788.755262 | 1193.932279 |
| MA3598 | 145.9025136 | 180.0731163 | 217.3749165 | 932.1690083 | 1557.424166 | 1039.526772 |
| MA3597 | 99.58831237 | 122.9120549 | 148.3730509 | 342.8153941 | 572.7598477 | 382.2973912 |
| MA3596 | 392.1587441 | 484.0029512 | 584.2632327 | 2100.691392 | 3509.736442 | 2342.627702 |
| MA3595 | 111.8058181 | 137.9909201 | 166.5754741 | 434.6854129 | 726.2519571 | 484.7480662 |
| MA3594 | 385.6198556 | 475.9326445 | 574.5211775 | 1294.585589 | 2162.932754 | 1443.682815 |
| MA3593 | 187.6540999 | 231.6029912 | 279.5791059 | 1250.370502 | 2089.060266 | 1394.375484 |
| MA3592 | 1193.857857 | 1473.461282 | 1778.685956 | 6608.860801 | 11041.774 | 7370.0023 |
| MA3591 | 1252.528909 | 1545.873189 | 1866.09785 | 2213.807256 | 3698.725111 | 2468.771103 |
| MA4663 | 269.747622 | 332.9229479 | 401.8872969 | 212.6137626 | 355.2250815 | 237.1004575 |
| MA3589 | 12494.17534 | 15420.33125 | 18614.62325 | 139946.7848 | 233816.5104 | 156064.4349 |
| MA3588 | 176.6485273 | 218.0198958 | 263.1822983 | 275.0559513 | 459.5505555 | 306.7341037 |
| MA3587 | 58.41742622 | 72.09888116 | 87.03402589 | 351.7031766 | 587.609137 | 392.2087783 |
| MA3586 | 439.7832948 | 542.781248 | 655.2173408 | 4479.233595 | 7483.692961 | 4995.106251 |
| MA3585 | 238.3080111 | 294.1201296 | 355.0465494 | 3458.03811 | 5777.527542 | 3856.299837 |
| MA3584 | 361.2265874 | 445.8264337 | 538.178523 | 4669.051716 | 7800.832156 | 5206.785697 |
| MA3583 | 559.6671988 | 690.7421547 | 833.8280651 | 5959.046054 | 9956.094065 | 6645.348488 |
| MA3582 | 166.5861704 | 205.6009187 | 248.1907541 | 1469.907654 | 2455.85262 | 1639.196696 |
| MA3581 | 9.857344397 | 12.16595027 | 14.6861035 | 60.24872908 | 100.6607448 | 67.1875661 |
| MA3580 | 6.552566191 | 8.087187705 | 9.762433102 | 32.68717018 | 54.61218759 | 36.45174662 |
| MA3579 | 1.932181523 | 2.384701536 | 2.878687878 | 85.21561067 | 142.3742371 | 95.02987967 |
| MA3578 | 42.03323589 | 51.87748718 | 62.62380898 | 228.1736453 | 381.2218022 | 254.4523696 |
| MA3577 | 558.2612541 | 689.0069355 | 831.7333985 | 1232.313023 | 2058.890678 | 1374.238327 |
| MA3576 | 112.2708449 | 138.5648569 | 167.2683008 | 2851.869416 | 4764.769378 | 3180.318785 |
| MA3575 | 235.8015674 | 291.0266727 | 351.3122889 | 885.6129005 | 1479.640409 | 987.6088044 |
| MA3573n | 101.9257837 | 125.7969657 | 151.8555655 | 271.9628994 | 454.3828298 | 303.2848255 |
| MA3572 | 993.3756902 | 1226.025871 | 1479.994774 | 3721.958037 | 6218.472552 | 4150.615382 |
| MA3571 | 166.6228998 | 205.6462503 | 248.245476 | 546.1060787 | 912.4083686 | 609.0010332 |
| MA3570 | 261.3083202 | 322.507148 | 389.3138841 | 715.3433415 | 1195.162033 | 797.729326 |
| MA3569 | 417.6343678 | 515.4450068 | 622.2184497 | 154.9875233 | 258.9458693 | 172.8374129 |
| MA3568 | 218.2397946 | 269.3519047 | 325.1476343 | 279.7919032 | 467.4631613 | 312.0154944 |
| MA3567 | 36.03693556 | 44.47684368 | 53.69013641 | 155.5367078 | 259.8634209 | 173.4498469 |
| MA3566 | 1163.641568 | 1436.16829 | 1733.667792 | 3631.136814 | 6066.732721 | 4049.334292 |
| MA3565 | 4004.778788 | 4942.704405 | 5966.576128 | 7616.793815 | 12725.78111 | 8494.018807 |
| MA3564 | 3576.725988 | 4414.400953 | 5328.835629 | 5549.84042 | 9272.412524 | 6189.014702 |
| MA3563 | 2555.694384 | 3154.242109 | 3807.637302 | 5583.050616 | 9327.898558 | 6226.049711 |
| MA3562 | 639.271314 | 788.9896814 | 952.4273782 | 1993.966536 | 3331.425569 | 2223.61136 |
| MA3561 | 194.3435059 | 239.8590668 | 289.5454117 | 772.0186526 | 1289.852479 | 860.9319241 |
| MA3560 | 326.9270136 | 403.4938447 | 487.0768195 | 1864.593163 | 3115.274619 | 2079.338076 |
| MA3559 | 257.3058326 | 317.5672714 | 383.3507216 | 1847.41809 | 3086.579314 | 2060.184952 |
| MA3558 | 51.19264073 | 63.18203932 | 76.27007739 | 196.2881998 | 327.9490984 | 218.8946821 |
| MA3557 | 116.300095 | 143.5377639 | 173.2713359 | 412.3469452 | 688.9298953 | 459.8368806 |
| MA3556 | 16073.68786 | 19838.17135 | 23947.61043 | 14771.30979 | 24679.21013 | 16472.51931 |
| MA3555 | 54.11886683 | 66.79359227 | 80.62975659 | 108.2887519 | 180.923757 | 120.7603511 |
| MA3554 | 3024.930634 | 3733.374242 | 4506.735544 | 2039.611415 | 3407.686888 | 2274.513153 |
| MA3553 | 749.601839 | 925.1597923 | 1116.804866 | 450.7474865 | 753.0877148 | 502.6600064 |
| MA3552 | 213.1360779 | 263.0528892 | 317.5437899 | 1185.101023 | 1980.011088 | 1321.588929 |
| MA3551 | 453.181729 | 559.317617 | 675.1791868 | 2265.321746 | 3784.7931 | 2526.218509 |
| MA3550 | 586.1725804 | 723.4551392 | 873.3174814 | 888.2006598 | 1483.963916 | 990.4945955 |
| MA3549 | 265.1851816 | 327.2919766 | 395.0898807 | 853.2723062 | 1425.607264 | 951.5435488 |
| MA3548 | 257.5200325 | 317.8316373 | 383.6698504 | 1541.913795 | 2576.157096 | 1719.495773 |
| MA3547 | 269.4544895 | 332.5611633 | 401.4505694 | 913.9931298 | 1527.056763 | 1019.25758 |
| MA3546 | 616.447461 | 760.8204453 | 918.422939 | 4026.452531 | 6727.20764 | 4490.17846 |
| MA3545 | 5049.123237 | 6231.636 | 7522.507426 | 14934.72378 | 24952.23455 | 16654.75367 |
| MA3544 | 159.5148688 | 196.873507 | 237.6554757 | 842.1185521 | 1406.972096 | 939.1052185 |
| MA3543 | 381.6866319 | 471.0782534 | 568.6612086 | 1366.112565 | 2282.436665 | 1523.447543 |
| MA3542 | 1746.801597 | 2155.905333 | 2602.496981 | 3761.567817 | 6284.650711 | 4194.787014 |
| MA3541 | 197.0489409 | 243.198119 | 293.5761422 | 1452.069009 | 2426.048651 | 1619.303577 |
| MA3540 | 10.79171315 | 13.31914968 | 16.07818595 | 135.3927203 | 226.2077934 | 150.9858794 |
| MA3539 | 145.5529402 | 179.6416723 | 216.8540996 | 723.2635363 | 1208.394722 | 806.5616884 |
| MA3538 | 59.89113309 | 73.91773255 | 89.22964884 | 359.4708382 | 600.5869809 | 400.8710403 |
| MA3537 | 43.51743281 | 53.70928539 | 64.83506068 | 522.4056104 | 872.8107402 | 582.5709856 |
| MA3536 | 26.74355692 | 33.00694086 | 39.84426525 | 136.5681059 | 228.1715725 | 152.2966341 |
| MA3535a | 87.5364396 | 108.0376142 | 130.4173984 | 689.3704932 | 1151.767819 | 768.7651887 |
| MA3535b | 25.33668372 | 31.27057569 | 37.74821537 | 241.0520176 | 402.7383813 | 268.8139421 |
| MA3535 | 59.83763324 | 73.85170294 | 89.14994133 | 334.8057802 | 559.3777612 | 373.3653113 |
| MA3534 | 67.74490647 | 83.61087225 | 100.9307038 | 426.0173239 | 711.7697215 | 475.0816748 |
| MA3533 | 99.86148374 | 123.2492034 | 148.7800392 | 368.0481214 | 614.9175025 | 410.4361679 |
| MA3532 | 344.2353437 | 424.8558135 | 512.8638791 | 1083.101717 | 1809.595442 | 1207.842378 |
| MA3531 | 436.5021006 | 538.7315929 | 650.3288074 | 2442.960768 | 4081.584028 | 2724.31619 |
| MA3529 | 29.29817545 | 36.15985514 | 43.65029967 | 189.5145524 | 316.6320066 | 211.3409147 |
| MA3528 | 1280.847441 | 1580.823967 | 1908.288615 | 5646.696686 | 9434.235422 | 6297.02589 |
| MA3527 | 12844.18122 | 15852.30906 | 19136.08443 | 32343.47251 | 54037.95369 | 36068.46535 |
| MA3526 | 616.0225834 | 760.2960607 | 917.7899291 | 1839.409379 | 3073.198736 | 2051.25388 |
| MA3525 | 389.8818704 | 481.1928299 | 580.8710002 | 3303.30335 | 5519.003977 | 3683.744297 |
| MA3524 | 5528.860939 | 6823.729041 | 8237.251403 | 8822.989915 | 14741.03686 | 9839.13233 |
| MA3523 | 32.94427876 | 40.6598817 | 49.08249808 | 169.0288954 | 282.4055339 | 188.4959278 |
| MA3522 | 361.8347389 | 446.5770153 | 539.0845861 | 3852.009248 | 6435.756003 | 4295.644571 |
| MA3521 | 121.0151232 | 149.3570592 | 180.2960871 | 1008.36907 | 1684.735647 | 1124.502783 |
| MA3520 | 914.2224583 | 1128.334825 | 1362.067216 | 8278.847564 | 13831.90939 | 9232.321186 |
| MA3519 | 678.0278123 | 836.8230137 | 1010.169293 | 6358.583454 | 10623.62237 | 7090.90055 |
| MA3517 | 681.6320274 | 841.2713417 | 1015.539084 | 242.4334605 | 405.0464311 | 270.3544855 |
| MA3516 | 5572.044948 | 6877.026815 | 8301.589708 | 4745.150296 | 7927.974086 | 5291.648538 |
| MA3515 | 1456.577427 | 1797.710197 | 2170.102411 | 1722.311195 | 2877.556594 | 1920.669515 |
| MA3514 | 1967.512362 | 2428.306914 | 2931.326027 | 4048.038853 | 6763.273052 | 4514.250876 |
| MA3513 | 328.3138679 | 405.2055024 | 489.1430438 | 593.483484 | 991.5643106 | 661.8348871 |
| MA3512 | 68.14119911 | 84.09997726 | 101.521126 | 1084.576666 | 1812.059718 | 1209.487197 |
| MA3510n | 2240.820136 | 2765.623806 | 3338.517465 | 12788.30303 | 21366.09563 | 14261.12995 |
| MA3509 | 54.17568264 | 66.86371443 | 80.71440442 | 336.7331346 | 562.5978945 | 375.5146389 |
| MA3508 | 40.76627887 | 50.31380678 | 60.7362152 | 475.1577913 | 793.8713047 | 529.8816423 |
| MA3507 | 525.2961791 | 648.3213871 | 782.6199169 | 3491.88805 | 5834.082431 | 3894.048269 |
| MA3506 | 506.9901498 | 625.7280563 | 755.3464211 | 1291.956427 | 2158.540074 | 1440.750853 |
| MA3504 | 767.0881562 | 946.7414331 | 1142.857102 | 4639.693507 | 7751.78184 | 5174.046307 |
| MA3503 | 61.70218333 | 76.15293366 | 91.92786757 | 357.6254767 | 597.5038377 | 398.8131488 |
| MA3502 | 267.0976308 | 329.6523244 | 397.9391702 | 2664.546836 | 4451.799617 | 2971.422293 |
| MA3501 | 139.1709938 | 171.7650638 | 207.3458668 | 364.504486 | 608.9969631 | 406.4844126 |
| MA3500 | 23.87776242 | 29.46997269 | 35.57462092 | 85.1050479 | 142.189514 | 94.9065834 |
| MA3499 | 31.06053547 | 38.33496271 | 46.27597659 | 98.29524188 | 164.2270701 | 109.61589 |
| MA3498 | 1861.641904 | 2297.641424 | 2773.593432 | 2037.644659 | 3404.400924 | 2272.319886 |
| MA3497 | 1538.007478 | 1898.211297 | 2291.42212 | 3738.681222 | 6246.412862 | 4169.264573 |
| MA3496 | 545.066192 | 672.7215687 | 812.0745493 | 5305.270187 | 8863.796074 | 5916.277352 |
| MA3495 | 454.9783491 | 561.5350084 | 677.8559066 | 643.6596381 | 1075.396271 | 717.7898214 |
| MA3494 | 64.46242308 | 79.55962599 | 96.04024967 | 260.7084546 | 435.5794323 | 290.7342079 |
| MA3492 | 590.3095481 | 728.5609913 | 879.481001 | 2449.148491 | 4091.922186 | 2731.216552 |
| MA3491 | 957.816517 | 1182.138682 | 1427.016439 | 2241.508576 | 3745.007176 | 2499.66278 |
| MA3490 | 4556.472176 | 5623.60527 | 6788.524298 | 17816.09496 | 29766.29408 | 19867.97192 |
| MA3489 | 137.9187973 | 170.2196009 | 205.4802642 | 693.7401558 | 1159.068446 | 773.6381047 |
| MA4667 | 424.3599235 | 523.7456984 | 632.2386136 | 1615.235488 | 2698.659535 | 1801.261915 |
| MA4666 | 90.50323487 | 111.6992377 | 134.8375202 | 322.896778 | 539.4807601 | 360.0847511 |
| MA3486 | 1036.09262 | 1278.747174 | 1543.637195 | 4317.708964 | 7213.8252 | 4814.978853 |
| MA3485 | 1006.905892 | 1242.724868 | 1500.152937 | 3052.027259 | 5099.183696 | 3403.528778 |
| MA3484 | 206.0948332 | 254.3625738 | 307.0532923 | 732.3699634 | 1223.609313 | 816.7169014 |
| MA3483 | 174.3820685 | 215.2226287 | 259.8055827 | 507.0412826 | 847.1407434 | 565.4371505 |
| MA3482 | 3363.583739 | 4151.340447 | 5011.282645 | 1491.659544 | 2492.194656 | 1663.453748 |
| MA3481 | 817.755185 | 1009.274761 | 1218.344089 | 859.1013413 | 1435.346142 | 958.043913 |
| MA3480 | 54.40209711 | 67.14315553 | 81.05173121 | 99.19801377 | 165.735379 | 110.622634 |
| MA3479 | 17.58391042 | 21.70209045 | 26.19763679 | 103.5471936 | 173.0017842 | 115.4727082 |
| MA3478 | 15.86567355 | 19.58143975 | 23.63769738 | 22.41924046 | 37.45701324 | 25.001261 |
| MA3477 | 171.4306607 | 211.5799965 | 255.4083863 | 439.3556898 | 734.0548362 | 489.9562182 |
| MA3476 | 80.12674961 | 98.89256292 | 119.3779674 | 193.3152965 | 322.9821114 | 215.57939 |
| MA3475 | 34.76359458 | 42.90528421 | 51.7930314 | 61.24497647 | 102.3252281 | 68.29855116 |
| MA3474 | 30.23953236 | 37.32167935 | 45.05279353 | 129.2587459 | 215.9594373 | 144.1454561 |
| MA3473b | 230.7347244 | 284.7731671 | 343.7633816 | 1027.549623 | 1716.781615 | 1145.892358 |
| MA3473a | 301.0405413 | 371.5447191 | 448.5094939 | 1484.80196 | 2480.737328 | 1655.806378 |
| MA3472 | 136.6815883 | 168.6926355 | 203.6369909 | 525.1473199 | 877.3914594 | 585.6284573 |
| MA3471 | 4512.811942 | 5569.719739 | 6723.476483 | 10288.77569 | 17190.00284 | 11473.73244 |
| MA3470 | 60.69410246 | 74.90875863 | 90.42596407 | 165.4805543 | 276.4771323 | 184.5389247 |
| MA3469 | 211.4466827 | 260.967835 | 315.0268207 | 329.029181 | 549.7264909 | 366.9234221 |
| MA3468 | 822.4705085 | 1015.094421 | 1225.36928 | 4145.111635 | 6925.457695 | 4622.503516 |
| MA3467 | 860.9935655 | 1062.63964 | 1282.7634 | 1678.325172 | 2804.066815 | 1871.617629 |
| MA3466 | 56.41198535 | 69.62376283 | 84.04619154 | 273.1532536 | 456.3716175 | 304.6122725 |
| MA3465 | 12.28283915 | 15.15949979 | 18.29976104 | 119.3075134 | 199.3333857 | 133.0481416 |
| MA3464 | 17.1091765 | 21.11617309 | 25.49034778 | 237.9485359 | 397.5532298 | 265.3530328 |
| MA3463 | 32.26875886 | 39.82615396 | 48.07606524 | 255.7298733 | 427.2614526 | 285.1822441 |
| MA3462 | 87.94885961 | 108.5466236 | 131.0318482 | 650.2680615 | 1086.437314 | 725.1593359 |
| MA3461 | 151.3913346 | 186.8474281 | 225.5525138 | 2347.838477 | 3922.658176 | 2618.238679 |
| MA3459 | 98.58382292 | 121.6723124 | 146.8764982 | 4308.965519 | 7199.217062 | 4805.228426 |
| MA4673 | 142.377528 | 175.7225735 | 212.123167 | 2147.659247 | 3588.208127 | 2395.004838 |
| MA3458 | 71.95864042 | 88.81146946 | 107.2085947 | 477.9934333 | 798.608962 | 533.0438648 |
| MA3457 | 83.77278872 | 103.3925102 | 124.8100701 | 642.9576495 | 1074.223421 | 717.0069848 |
| MA3456 | 115.5561827 | 142.6196261 | 172.1630076 | 931.9543075 | 1557.065454 | 1039.287344 |
| MA3455 | 149.780718 | 184.8596024 | 223.152914 | 1102.18048 | 1841.471342 | 1229.11844 |
| MA3454 | 173.2053158 | 213.7702786 | 258.0523811 | 1223.317675 | 2043.861673 | 1364.206986 |
| MA3453a | 379.4057963 | 468.2632425 | 565.2630736 | 987.2795112 | 1649.500204 | 1100.984343 |
| MA3453 | 37.80764793 | 46.66225974 | 56.3282572 | 383.4173671 | 640.5957159 | 427.5754873 |
| MA3452 | 35.51156628 | 43.82843208 | 52.90740759 | 600.050478 | 1002.536135 | 669.1582008 |
| MA3451 | 84.17684415 | 103.8911961 | 125.4120578 | 1719.007724 | 2872.03731 | 1916.985584 |
| MA3450 | 76.69302098 | 94.65464954 | 114.2621783 | 1555.654464 | 2599.114361 | 1734.818954 |
| MA3449 | 170.4464156 | 210.3652395 | 253.9419948 | 2247.910758 | 3755.703641 | 2506.802301 |
| MA3448 | 92.30329113 | 113.92087 | 137.5193594 | 246.3897554 | 411.6564226 | 274.7664262 |
| MA3447 | 32.79094012 | 40.4706309 | 48.85404435 | 52.84438448 | 88.28991383 | 58.93046425 |
| MA3446 | 1134.334925 | 1399.997984 | 1690.004877 | 2679.929065 | 4477.499521 | 2988.57609 |
| MA3445 | 606.2343632 | 748.2154237 | 903.2068113 | 938.1370296 | 1567.395256 | 1046.18213 |
| MA3444 | 139.3870261 | 172.0316912 | 207.6677256 | 354.0776201 | 591.5762456 | 394.8566861 |
| MA3443 | 193.9634675 | 239.3900228 | 288.9792061 | 409.7443336 | 684.5815741 | 456.9345266 |
| MA3442 | 142.840336 | 176.2937719 | 212.8126881 | 506.5418312 | 846.3062835 | 564.8801774 |
| MA3441 | 582.9837276 | 719.5194519 | 868.5665242 | 683.5680624 | 1142.07339 | 762.2944929 |
| MA3440 | 309.4780791 | 381.9583417 | 461.0802784 | 2039.008351 | 3406.679316 | 2273.840634 |
| MA3439 | 485.3691242 | 599.0433518 | 723.1340314 | 9340.427916 | 15605.54795 | 10416.16359 |
| MA3438 | 61.83711253 | 76.31946349 | 92.12889373 | 361.3585676 | 603.7409104 | 402.9761792 |
| MA3437 | 509.0879442 | 628.3171575 | 758.4718496 | 3742.340146 | 6252.526022 | 4173.344895 |
| MA3436 | 391.763851 | 483.5155734 | 583.6748956 | 351.6802032 | 587.5707541 | 392.183159 |
| MA3435 | 28.80981615 | 35.55712131 | 42.92271068 | 38.14951114 | 63.73841013 | 42.54318457 |
| MA3434 | 18.83430416 | 23.24532841 | 28.06055354 | 45.20965381 | 75.53416467 | 50.4164428 |
| MA3432 | 1243.439469 | 1534.654988 | 1852.55582 | 6657.709579 | 11123.38824 | 7424.476985 |
| MA3431 | 76.09575499 | 93.91750289 | 113.3723332 | 936.0776282 | 1563.954504 | 1043.885547 |
| MA3430 | 464.0775643 | 572.7652744 | 691.4125007 | 1064.830969 | 1779.069534 | 1187.467391 |
| MA3429 | 391.2579653 | 482.8912084 | 582.9211946 | 359.5105562 | 600.6533398 | 400.9153326 |
| MA3428 | 1251.889076 | 1545.083506 | 1865.144586 | 2336.80872 | 3904.230176 | 2605.938627 |
| MA3427 | 404.3811063 | 499.0878102 | 602.4728913 | 575.5736604 | 961.6414191 | 641.8623918 |
| MA3426 | 231.3497295 | 285.5322073 | 344.6796555 | 386.0351882 | 644.9694484 | 430.4948025 |
| MA3425 | 6.624016369 | 8.17537163 | 9.868884157 | 65.27483149 | 109.0581205 | 72.79252397 |
| MA3424 | 162.871081 | 201.0157495 | 242.6557758 | 1027.929693 | 1717.416617 | 1146.3162 |
| MA3423 | 42.80023717 | 52.82412139 | 63.76653665 | 71.52849509 | 119.5064477 | 79.76642106 |
| MA3422 | 19.92158013 | 24.58724615 | 29.68044696 | 127.7858778 | 213.4986385 | 142.502958 |
| MA3420 | 1895.478208 | 2339.402245 | 2824.004926 | 4813.242253 | 8041.738928 | 5367.582636 |
| MA3419 | 563.6755751 | 695.6892991 | 839.8000009 | 859.9517553 | 1436.766974 | 958.9922689 |
| MA3418a | 35.44480336 | 43.74603318 | 52.80793991 | 115.2510128 | 192.5559753 | 128.5244546 |
| MA3418 | 1440.891778 | 1778.350945 | 2146.732926 | 7199.809911 | 12029.104 | 8029.01093 |
| MA3417 | 1305.846942 | 1611.678391 | 1945.534473 | 19172.64152 | 32032.74831 | 21380.75175 |
| MA3416 | 2368.525459 | 2923.237922 | 3528.781042 | 36630.85972 | 61201.11873 | 40849.6303 |
| MA3415 | 262.6051624 | 324.1077127 | 391.2460028 | 1284.781081 | 2146.551844 | 1432.749124 |
| MA3414 | 34.86341335 | 43.02848068 | 51.9417478 | 141.2039427 | 235.9169107 | 157.4663794 |
| MA3414a | 106.4641599 | 131.3982369 | 158.6171292 | 412.4272915 | 689.0641341 | 459.9264804 |
| MA3413 | 35.40824091 | 43.70090774 | 52.75346683 | 152.4123663 | 254.6434181 | 169.9656755 |
| MA3412a | 123.7688787 | 152.7557487 | 184.3988086 | 257.2692203 | 429.8333212 | 286.8988775 |
| MA3412 | 1171.766829 | 1446.1965 | 1745.773327 | 1329.093882 | 2220.587589 | 1482.165424 |
| MA3411 | 964.356702 | 1190.210589 | 1436.760426 | 2019.482629 | 3374.056657 | 2252.066138 |
| MA3410 | 937.8610074 | 1157.50956 | 1397.285442 | 2731.37983 | 4563.461042 | 3045.952432 |
| MA3409 | 280.3874651 | 346.0546592 | 417.7392171 | 817.1905197 | 1365.323511 | 911.3062284 |
| MA3408 | 16743.74773 | 20665.16031 | 24945.90856 | 9467.116776 | 15817.21374 | 10557.44318 |
| MA3407 | 39.89335192 | 49.23643894 | 59.43567268 | 462.9896559 | 773.5413559 | 516.3121046 |
| MA3406 | 1493.921611 | 1843.800449 | 2225.74017 | 9075.752531 | 15163.34076 | 10121.00558 |
| MA3405 | 164.0761114 | 202.5029999 | 244.4511073 | 637.3889233 | 1064.919455 | 710.7969093 |
| MA3404 | 143.7024336 | 177.3577741 | 214.0970963 | 476.1241308 | 795.4858192 | 530.9592749 |
| MA3403 | 78.57229527 | 96.97405287 | 117.0620416 | 260.7950637 | 435.7241348 | 290.8307918 |
| MA3402 | 87.92328728 | 108.5150622 | 130.9937489 | 259.8264362 | 434.1057975 | 289.7506076 |
| MA3401 | 150.1307697 | 185.2916368 | 223.6744434 | 295.9966556 | 494.5372999 | 330.0865456 |
| MA3400 | 106.6010089 | 131.5671363 | 158.8210158 | 385.8343592 | 644.6339126 | 430.270844 |
| MA3399 | 351.617037 | 433.9663111 | 523.8615988 | 593.5863013 | 991.7360929 | 661.9495459 |
| MA3398 | 86.14510004 | 106.3204207 | 128.3444916 | 119.440598 | 199.5557372 | 133.1965536 |
| MA3397 | 199.10531 | 245.736093 | 296.6398526 | 830.6219113 | 1387.764049 | 926.2845113 |
| MA3396 | 159.8130829 | 197.2415633 | 238.0997742 | 806.2636779 | 1347.067458 | 899.1209438 |
| MA3395 | 56.05247934 | 69.18005993 | 83.51057644 | 273.829078 | 457.5007532 | 305.3659315 |
| MA3394a | 340.058289 | 419.7004858 | 506.6406352 | 2955.599073 | 4938.076014 | 3295.994971 |
| MA3393 | 141.5375183 | 174.6858322 | 210.8716667 | 346.4128546 | 578.7703156 | 386.3091707 |
| MA3392 | 252.1338524 | 311.1840051 | 375.6451739 | 1255.279021 | 2097.261189 | 1399.849317 |
| MA3391a | 526.572334 | 649.8964195 | 784.5212143 | 423.7094887 | 707.9138989 | 472.5080465 |
| MA3391 | 38.78774209 | 47.87189352 | 57.7884643 | 146.4792631 | 244.7306681 | 163.3492577 |
| MA3390a | 1827.58845 | 2255.612597 | 2722.858413 | 2545.134677 | 4252.291393 | 2838.257453 |
| MA3389 | 458.9924648 | 566.4892364 | 683.8363935 | 1630.395241 | 2723.987738 | 1818.167614 |
| MA3388 | 739.8812908 | 913.1626762 | 1102.32257 | 1477.406508 | 2468.381353 | 1647.559192 |
| MA3387 | 1669.269057 | 2060.214548 | 2486.984034 | 12534.88405 | 20942.69512 | 13978.52476 |
| MA3385n | 163.2219112 | 201.4487447 | 243.1784652 | 243.5929931 | 406.9837237 | 271.6475613 |
| MA3384 | 1931.327026 | 2383.646913 | 2877.414792 | 4128.340542 | 6897.43734 | 4603.800898 |
| MA3383 | 327.8377332 | 404.6178563 | 488.433668 | 593.8202304 | 992.1269307 | 662.2104166 |
| MA3382 | 2506.361093 | 3093.354882 | 3734.137403 | 77738.05519 | 129881.0888 | 86691.13526 |
| MA3381 | 395.5008112 | 488.1277357 | 589.2424584 | 1141.475231 | 1907.123166 | 1272.938761 |
| MA3380 | 47.55771442 | 58.69580744 | 70.85453119 | 202.6377024 | 338.5575489 | 225.9754559 |
| MA3379 | 648.4327819 | 800.2967798 | 966.0767203 | 693.1371654 | 1158.060998 | 772.9656679 |
| MA3376a | 582.5013418 | 718.9240905 | 867.8478348 | 5908.295782 | 9871.302895 | 6588.753314 |
| MA3376 | 301.4844344 | 372.0925726 | 449.1708342 | 768.8084409 | 1284.489008 | 857.3519928 |
| MA3374n | 17.70242593 | 21.84836249 | 26.3742088 | 87.12947748 | 145.5718358 | 97.16416623 |
| MA3373 | 1341.403204 | 1655.561987 | 1998.508472 | 3416.489185 | 5708.109551 | 3809.965728 |
| MA3372 | 660.938911 | 815.7318643 | 984.7091529 | 2278.554926 | 3806.902475 | 2540.975752 |
| MA3371 | 348.3929373 | 429.9871222 | 519.0581285 | 1456.384897 | 2433.259433 | 1624.116525 |
| MA3370 | 648.4685124 | 800.3408784 | 966.1299539 | 1967.663732 | 3287.480081 | 2194.279267 |
| MA3369 | 1581.078545 | 1951.369677 | 2355.592157 | 3452.276139 | 5767.900712 | 3849.87426 |
| MA3368 | 1103.436491 | 1361.863088 | 1643.97041 | 3196.521423 | 5340.597753 | 3564.664312 |
| MA3367 | 168.1910645 | 207.5816817 | 250.5818283 | 384.0942361 | 641.7265967 | 428.3303112 |
| MA3366 | 642.9523807 | 793.5328598 | 957.9116673 | 596.6189156 | 996.8028424 | 665.331426 |
| MA3365 | 98.34586705 | 121.3786269 | 146.5219763 | 299.8537771 | 500.9815972 | 334.3878913 |
| MA3364 | 31.64736826 | 39.05923269 | 47.1502777 | 108.5458686 | 181.3533354 | 121.0470798 |
| MA3362 | 22.55074765 | 27.83216893 | 33.59754925 | 101.1263249 | 168.9571105 | 112.7730284 |
| MA3361 | 1168.374313 | 1442.009452 | 1740.71894 | 2825.863403 | 4721.319755 | 3151.317664 |
| MA3360 | 670.6786276 | 827.7526382 | 999.220007 | 1561.471854 | 2608.833782 | 1741.306331 |
| MA3359a | 257.3939232 | 317.675993 | 383.4819646 | 269.4196888 | 450.1337529 | 300.4487136 |
| MA3359 | 522.1043211 | 644.3819908 | 777.8644822 | 1129.845154 | 1887.692179 | 1259.969249 |
| MA3358 | 394.3219482 | 486.6727811 | 587.4861128 | 907.3489879 | 1515.956042 | 1011.848234 |
| MA3357 | 1112.970112 | 1373.629498 | 1658.174209 | 1711.396985 | 2859.321644 | 1908.498317 |
| MA3356 | 324.2463715 | 400.1853919 | 483.083027 | 810.6579698 | 1354.409234 | 904.0213257 |
| MA3355a | 16.78880063 | 20.72076467 | 25.01303126 | 158.2294933 | 264.3624005 | 176.4527602 |
| MA3355 | 226.8044189 | 279.9223776 | 337.9077604 | 626.243794 | 1046.298697 | 698.3681971 |
| MA3354 | 34.43486302 | 42.49956318 | 51.30326605 | 116.9179714 | 195.3410514 | 130.3833965 |
| MA3352 | 4.469832399 | 5.516674318 | 6.659442804 | 45.27576021 | 75.64461215 | 50.49016267 |
| MA3351a | 4.472258162 | 5.519668198 | 6.663056862 | 46.51048999 | 77.70754062 | 51.86709609 |
| MA3351 | 70.91852653 | 87.52775922 | 105.6589664 | 176.1805869 | 294.3542439 | 196.4712785 |
| MA3350 | 453.3743178 | 559.5553103 | 675.4661179 | 1000.704134 | 1671.929433 | 1115.955078 |
| MA3349 | 190.2550143 | 234.8130439 | 283.4541148 | 1173.893409 | 1961.285934 | 1309.090536 |
| MA3348 | 129.9450347 | 160.3783704 | 193.6004417 | 1037.55558 | 1733.499097 | 1157.0507 |
| MA3347 | 474.8356243 | 586.0428894 | 707.440548 | 4390.657099 | 7335.703515 | 4896.328414 |
| MA3346 | 1118.762996 | 1380.779085 | 1666.804819 | 4899.515274 | 8185.879836 | 5463.791707 |
| MA3345 | 7860.468343 | 9701.40264 | 11711.02956 | 13675.7847 | 22848.8583 | 15250.82276 |
| MA3344 | 1560.030058 | 1925.391601 | 2324.232772 | 6285.121223 | 10500.88513 | 7008.977684 |
| MA3343 | 403.2123467 | 497.6453252 | 600.7315982 | 1631.077626 | 2725.127835 | 1818.92859 |
| MA3342 | 1512.534909 | 1866.773011 | 2253.471454 | 11591.57873 | 19366.66493 | 12926.5791 |
| MA3341 | 220.9940849 | 272.7512542 | 329.2511525 | 368.9801569 | 616.4747037 | 411.4755458 |
| MA3340 | 44.20271528 | 54.55506212 | 65.85603843 | 304.0373999 | 507.9713976 | 339.0533413 |
| MA3339 | 441.9419379 | 545.4454488 | 658.4334256 | 1965.732558 | 3284.253566 | 2192.12568 |
| MA3337 | 330.1218401 | 407.4369047 | 491.8366768 | 2851.613007 | 4764.340983 | 3180.032846 |
| MA3336 | 57.19626909 | 70.59172707 | 85.21466772 | 221.3531992 | 369.8265213 | 246.8464137 |
| MA3335 | 322.9924843 | 398.6378423 | 481.2149055 | 1991.574114 | 3327.428424 | 2220.943404 |
| MA3334a | 280.7551673 | 346.508478 | 418.2870435 | 1568.241731 | 2620.144575 | 1748.855894 |
| MA3334 | 2245.766994 | 2771.729227 | 3345.887612 | 1501.589687 | 2508.785473 | 1674.527545 |
| MA3332 | 302.7043472 | 373.598191 | 450.9883385 | 773.9893263 | 1293.144988 | 863.12956 |
| MA3331 | 294.4506717 | 363.4114914 | 438.6914838 | 247.7125945 | 413.8665602 | 276.2416166 |
| MA3330 | 150.6561581 | 185.940072 | 224.4572007 | 422.0734274 | 705.1804445 | 470.6835603 |
| MA3329 | 56.99354051 | 70.34151913 | 84.91262969 | 154.3520471 | 257.8841456 | 172.128749 |
| MA3328a | 22.20090826 | 27.40039659 | 33.07633611 | 54.92353041 | 91.76365314 | 61.24906511 |
| MA3328 | 645.551257 | 796.7403971 | 961.7836398 | 742.671934 | 1240.821361 | 828.2053484 |
| MA3325 | 2601.186587 | 3210.388659 | 3875.414502 | 12659.10158 | 21150.23192 | 14117.04839 |
| MA3324 | 913.0882944 | 1126.935038 | 1360.377466 | 5776.494693 | 9651.09583 | 6441.772713 |
| MA3323 | 896.4459183 | 1106.394991 | 1335.582587 | 4649.036491 | 7767.391659 | 5184.465321 |
| MA3322 | 313.1415892 | 386.4798518 | 466.5384106 | 983.1793762 | 1642.649891 | 1096.411996 |
| MA3321 | 403.2501566 | 497.6919902 | 600.7879299 | 826.2887673 | 1380.524436 | 921.4523198 |
| MA3320 | 175.0809221 | 216.0851549 | 260.8467797 | 345.0204058 | 576.4438777 | 384.7563538 |
| MA3319 | 1181.225656 | 1457.8706 | 1759.865694 | 1362.134471 | 2275.790252 | 1519.011292 |
| MA3318 | 19.64675094 | 24.24805154 | 29.27098883 | 134.4794968 | 224.6820225 | 149.9674801 |
| MA3317 | 1246.279559 | 1538.160232 | 1856.787169 | 2788.34537 | 4658.636389 | 3109.478685 |
| MA3316 | 12.77398727 | 15.76567558 | 19.03150499 | 37.03240788 | 61.87200652 | 41.2974247 |
| MA3315 | 24.12820674 | 29.77907147 | 35.94774891 | 139.2163183 | 232.5960813 | 155.2498406 |
| MA3314 | 125.9110302 | 155.3995955 | 187.5903231 | 321.8549247 | 537.7400807 | 358.9229078 |
| MA3313 | 276.6638221 | 341.4589332 | 412.1914952 | 3552.285401 | 5934.991486 | 3961.401574 |
| MA3311n | 182.671159 | 225.4530375 | 272.1552011 | 1470.02022 | 2456.04069 | 1639.322226 |
| MA3310 | 1770.861929 | 2185.600634 | 2638.343607 | 10385.15533 | 17351.02942 | 11581.2121 |
| MA3309 | 1839.382725 | 2270.16911 | 2740.43028 | 9462.808002 | 15810.01485 | 10552.63817 |
| MA3308 | 231.898039 | 286.2089317 | 345.4965621 | 1037.928709 | 1734.122503 | 1157.466802 |
| MA3307 | 198.6329559 | 245.1531129 | 295.9361092 | 2506.307341 | 4187.420507 | 2794.958379 |
| MA3306 | 177.9510666 | 219.6274919 | 265.122905 | 2170.627456 | 3626.582331 | 2420.618292 |
| MA3305 | 527.8940246 | 651.5276521 | 786.4903536 | 1509.753282 | 2522.424824 | 1683.63134 |
| MA3304 | 262.8863423 | 324.4547455 | 391.6649227 | 992.9960462 | 1659.051122 | 1107.359251 |
| MA3303 | 16.02694689 | 19.78048356 | 23.87797273 | 166.2778243 | 277.8091736 | 185.4280162 |
| MA3302 | 9883.253235 | 12197.927 | 14724.70414 | 1641.826958 | 2743.087314 | 1830.915921 |
| MA3301 | 482.1057865 | 595.0157352 | 718.2721016 | 631.5692773 | 1055.196264 | 704.3070156 |
| MA3300 | 12706.13842 | 15681.93642 | 18930.41942 | 3823.466724 | 6388.068495 | 4263.814809 |
| MA3299 | 673.9512013 | 831.7916539 | 1004.095697 | 2562.001082 | 4280.470991 | 2857.06636 |
| MA3298 | 1124.408014 | 1387.746175 | 1675.215129 | 7944.654674 | 13273.55562 | 8859.639352 |
| MA3297 | 4285.907561 | 5289.673988 | 6385.419793 | 33947.69815 | 56718.21848 | 37857.44941 |
| MA3296 | 339.5188393 | 419.0346962 | 505.8369285 | 1896.761678 | 3169.020262 | 2115.211434 |
| MA3295 | 121.5981659 | 150.0766515 | 181.1647415 | 1211.249128 | 2023.698112 | 1350.748507 |
| MA3294 | 450.5720906 | 556.096797 | 671.2911802 | 2057.814548 | 3438.099826 | 2294.812738 |
| MA3293 | 2145.864591 | 2648.42952 | 3197.046608 | 6747.016436 | 11272.59794 | 7524.069299 |
| MA3292 | 166.1776351 | 205.0967038 | 247.582092 | 1014.267983 | 1694.591274 | 1131.081074 |
| MA3291 | 257.3511892 | 317.6232506 | 383.4182968 | 716.5319762 | 1197.147948 | 799.0548556 |
| MA3290 | 380.4922952 | 469.6042012 | 566.8818093 | 1637.230681 | 2735.40807 | 1825.790291 |
| MA3289 | 14154.46001 | 17469.45725 | 21088.22176 | 51480.45272 | 86011.12075 | 57409.44868 |
| MA3288 | 2447.328854 | 3020.497198 | 3646.18739 | 24644.30668 | 41174.54929 | 27482.58776 |
| MA3287 | 925.5667396 | 1142.335955 | 1378.968653 | 10176.00323 | 17001.58792 | 11347.97199 |
| MA3286 | 140.8528325 | 173.8407919 | 209.851578 | 269.9717875 | 451.0561735 | 301.0643974 |
| MA3285 | 274.7463346 | 339.092367 | 409.3346994 | 518.3115952 | 865.970652 | 578.0054633 |
| MA3284 | 2251.197381 | 2778.43142 | 3353.978151 | 2451.445202 | 4095.759422 | 2733.777775 |
| MA3283 | 58.44741176 | 72.13588936 | 87.07870026 | 529.6715786 | 884.9503783 | 590.6737743 |
| MA3282 | 338.0183382 | 417.1827753 | 503.6013858 | 2562.531428 | 4281.357068 | 2857.657785 |
| MA3281 | 389.7619364 | 481.0448072 | 580.6923149 | 1491.134526 | 2491.31748 | 1662.868263 |
| MA3280 | 1924.880417 | 2375.690499 | 2867.810222 | 5868.289221 | 9804.461813 | 6544.139203 |
| MA3279 | 2539.284863 | 3133.989452 | 3783.189347 | 17568.65769 | 29352.88752 | 19592.03734 |
| MA3278 | 212.2904322 | 262.009192 | 316.2838927 | 5050.639864 | 8438.371698 | 5632.321293 |
| MA3277 | 29.34381184 | 36.21617966 | 43.7182917 | 82.32513434 | 137.5449651 | 91.80650762 |
| MA3275 | 55.60864144 | 68.63227448 | 82.84931829 | 245.5775848 | 410.2994862 | 273.8607181 |
| MA3272n | 315.9482414 | 389.9438264 | 470.7199409 | 998.8015129 | 1668.750623 | 1113.833332 |
| MA3271 | 1135.28104 | 1401.165681 | 1691.41446 | 3273.110935 | 5468.55991 | 3650.074627 |
| MA3270 | 1328.003565 | 1639.024131 | 1978.544831 | 6186.724995 | 10336.48934 | 6899.249178 |
| MA3269 | 577.5935419 | 712.8668761 | 860.53588 | 2338.11169 | 3906.407117 | 2607.39166 |
| MA3268 | 435.0725745 | 536.9672695 | 648.1990078 | 1576.520974 | 2633.977144 | 1758.088656 |
| MA3267 | 122.4438518 | 151.1203983 | 182.4246985 | 1080.473343 | 1805.204078 | 1204.911295 |
| MA3266 | 340.8205414 | 420.641259 | 507.7762877 | 1357.20889 | 2267.560824 | 1513.518433 |
| MA3265a | 29.32209302 | 36.18937425 | 43.6859336 | 74.77406909 | 124.9290002 | 83.38578732 |
| MA3265 | 381.7162615 | 471.1148224 | 568.7053527 | 437.3009574 | 730.6218859 | 487.6648425 |
| MA3264 | 2800.933898 | 3456.917111 | 4173.010849 | 2150.066046 | 3592.229294 | 2397.688828 |
| MA3263 | 67.83601582 | 83.72331955 | 101.0664444 | 1088.574752 | 1818.739531 | 1213.945741 |
| MA3262 | 108.5860665 | 134.0170975 | 161.7784818 | 1355.485147 | 2264.680874 | 1511.596166 |
| MA3261 | 795.8985384 | 982.299253 | 1185.780656 | 2292.765146 | 3830.644243 | 2556.822561 |
| MA3260 | 14059.38574 | 17352.11644 | 20946.57403 | 9026.751261 | 15081.47174 | 10066.36084 |
| MA3259 | 49.79111281 | 61.45227131 | 74.18199126 | 172.6460163 | 288.4488494 | 192.5296319 |
| MA3258 | 176.4128948 | 217.7290778 | 262.831238 | 567.2616385 | 947.7540836 | 632.5930757 |
| MA3257 | 484.0550107 | 597.4214708 | 721.1761808 | 1954.654074 | 3265.744155 | 2179.771288 |
| MA3256 | 49.43480854 | 61.01252002 | 73.65114633 | 210.3518373 | 351.4459629 | 234.5780266 |
| MA3255 | 55.35263404 | 68.31630974 | 82.46790206 | 608.2818998 | 1016.288808 | 678.3376343 |
| MA3254 | 6854.356343 | 8459.657596 | 10212.05941 | 4254.976318 | 7109.014444 | 4745.021298 |
| MA3253 | 523.0199567 | 645.5120696 | 779.2286548 | 2266.105019 | 3786.101757 | 2527.091992 |
| MA3252 | 3210.834538 | 3962.817138 | 4783.707095 | 2823.421789 | 4717.240421 | 3148.59485 |
| MA3250 | 3391.407847 | 4185.681006 | 5052.736785 | 13837.11013 | 23118.39324 | 15430.72801 |
| MA3249 | 8.228554998 | 10.15569578 | 12.25942865 | 76.33366792 | 127.5347046 | 85.12500492 |
| MA3248 | 1821.741222 | 2248.395939 | 2714.146839 | 3701.841833 | 6184.863343 | 4128.182397 |
| MA3247 | 727.8339237 | 898.2937964 | 1084.373631 | 1218.162914 | 2035.249342 | 1358.458553 |
| MA3246 | 571.3080045 | 705.1092558 | 851.1712835 | 687.5716124 | 1148.762333 | 766.7591311 |
| MA3245 | 361.5227849 | 446.1920012 | 538.6198171 | 516.0635659 | 862.2147502 | 575.4985287 |
| MA3244 | 33.83826546 | 41.76324151 | 50.41441676 | 128.1581569 | 214.120625 | 142.9181125 |
| MA3243 | 227.618327 | 280.9269044 | 339.1203729 | 653.8302528 | 1092.388855 | 729.1317842 |
| MA3242 | 2536.049333 | 3129.996156 | 3778.368847 | 11713.70465 | 19570.70716 | 13062.77024 |
| MA3241 | 92.36239844 | 113.9938203 | 137.6074212 | 813.6167316 | 1359.352594 | 907.3208477 |
| MA3239 | 428.5941432 | 528.9715792 | 638.5470256 | 770.3127769 | 1287.002382 | 859.029583 |
| MA3238 | 14.68330785 | 18.12216211 | 21.87613317 | 55.54308982 | 92.79878388 | 61.93997908 |
| MA3237 | 134.4582637 | 165.9486048 | 200.3245395 | 802.192833 | 1340.266081 | 894.5812603 |
| MA3236 | 77.36356718 | 95.48223873 | 115.2612011 | 239.8285323 | 400.6942396 | 267.4495482 |
| MA3235 | 59.43429184 | 73.35389835 | 88.54901747 | 467.5454115 | 781.1528983 | 521.3925459 |
| MA3232 | 121.0716431 | 149.4268162 | 180.3802941 | 252.0171739 | 421.058449 | 281.0419537 |
| MA3231 | 125.0656913 | 154.3562769 | 186.3308831 | 320.2659353 | 535.0852717 | 357.1509146 |
| MA3230 | 10.0198038 | 12.36645792 | 14.92814592 | 43.68186105 | 72.98160034 | 48.71269438 |
| MA3229 | 171.9985817 | 212.2809254 | 256.2545113 | 536.4648409 | 896.3002417 | 598.2494156 |
| MA3228 | 7.404987043 | 9.139246888 | 11.03242432 | 31.35162709 | 52.38082497 | 34.96238924 |
| MA3227 | 9.216555972 | 11.37508817 | 13.73141583 | 93.05267812 | 155.468041 | 103.769541 |
| MA3221n | 176.18754 | 217.4509445 | 262.49549 | 844.8156924 | 1411.478351 | 942.112988 |
| MA3220 | 15.37158956 | 18.9716405 | 22.90157938 | 26.93075381 | 44.99463769 | 30.03236466 |
| MA3219 | 56.62538745 | 69.88714404 | 84.36413167 | 339.6468688 | 567.4660245 | 378.7639473 |
| MA3218 | 29.70872285 | 36.66655341 | 44.26195951 | 130.4830702 | 218.0049808 | 145.5107856 |
| MA3217 | 74.30665131 | 91.70938826 | 110.7068119 | 254.4120684 | 425.0597262 | 283.7126679 |
| MA3216 | 49.72571509 | 61.37155734 | 74.08455754 | 281.0283674 | 469.5289875 | 313.394362 |
| MA3215 | 47.26181477 | 58.33060761 | 70.41368092 | 294.2331574 | 491.5909301 | 328.1199457 |
| MA3214 | 649.3152684 | 801.385946 | 967.3915051 | 946.7499267 | 1581.785279 | 1055.786973 |
| MA3213 | 1261.186795 | 1556.558766 | 1878.996924 | 1902.204227 | 3178.113417 | 2121.280801 |
| MA3212 | 1660.04784 | 2048.833707 | 2473.245674 | 3857.162592 | 6444.365968 | 4301.391425 |
| MA3211 | 178.0379553 | 219.7347301 | 265.2523573 | 1284.10854 | 2145.428194 | 1431.999126 |
| MA3210 | 7.045147258 | 8.695132048 | 10.49631195 | 44.60481334 | 74.52362564 | 49.74194295 |
| MA3209 | 20.47780028 | 25.27373396 | 30.5091394 | 68.07443954 | 113.735574 | 75.91456246 |
| MA3208 | 128.9060949 | 159.0961092 | 192.0525625 | 407.1211334 | 680.1988545 | 454.0092128 |
| MA3207 | 602.8216207 | 744.0034114 | 898.1222888 | 1126.045192 | 1881.343382 | 1255.731647 |
| MA3206 | 739.1519122 | 912.2624759 | 1101.235895 | 7687.990296 | 12844.73284 | 8573.414977 |
| MA3205 | 571.4417584 | 705.2743351 | 851.3705587 | 3921.440839 | 6551.75904 | 4373.072587 |
| MA3204 | 489.1775426 | 603.7437079 | 728.8080571 | 2803.064246 | 4683.227995 | 3125.892732 |
| MA3203 | 389.9501191 | 481.2770626 | 580.9726816 | 3436.595883 | 5741.702876 | 3832.388111 |
| MA3201 | 389.9015659 | 481.2171382 | 580.900344 | 4441.299921 | 7420.315162 | 4952.803762 |
| MA3200 | 398.864134 | 492.2787541 | 594.2533523 | 3161.52222 | 5282.12273 | 3525.634256 |
| MA3199 | 353.5704516 | 436.3772187 | 526.7719212 | 251.1828164 | 419.6644437 | 280.1115034 |
| MA3198 | 1845.748389 | 2278.025623 | 2749.914255 | 4330.122582 | 7234.565288 | 4828.822145 |
| MA3197 | 583.0431896 | 719.59284 | 868.6551145 | 1975.660732 | 3300.841093 | 2203.19728 |
| MA3196 | 95.29560087 | 117.6139835 | 141.9774942 | 544.4415959 | 909.6274289 | 607.1448522 |
| MA3195 | 877.5518753 | 1083.075932 | 1307.433031 | 8651.989336 | 14455.33713 | 9648.43764 |
| MA3194 | 461.6057389 | 569.7145435 | 687.7298167 | 5669.062278 | 9471.602801 | 6321.967324 |
| MA3193 | 1432.216453 | 1767.643844 | 2133.80787 | 31918.84336 | 53328.50326 | 35594.93173 |
| MA3192 | 184.1847416 | 227.3211035 | 274.4102334 | 388.2807471 | 648.7212225 | 432.998982 |
| MA3191 | 419.4904866 | 517.7358315 | 624.9838144 | 1668.029691 | 2786.865608 | 1860.136418 |
| MA3190 | 431.9901767 | 533.1629691 | 643.6066539 | 2143.727284 | 3581.638788 | 2390.620031 |
| MA3189 | 667.3651802 | 823.6631761 | 994.2834207 | 5268.300345 | 8802.0286 | 5875.0497 |
| MA3187 | 34.31182213 | 42.34770592 | 51.11995185 | 132.0812172 | 220.6750898 | 147.2929911 |
| MA3186 | 268.6144863 | 331.5244299 | 400.1990787 | 546.2761051 | 912.6924407 | 609.1906416 |
| MA3185 | 160.3004416 | 197.8430622 | 238.8258724 | 774.0442833 | 1293.236807 | 863.1908464 |
| MA3184 | 43.48492121 | 53.66915953 | 64.78662282 | 340.3059078 | 568.5671159 | 379.4988877 |
| MA3182 | 75.54980537 | 93.243691 | 112.5589425 | 279.342313 | 466.7120071 | 311.514125 |
| MA3181 | 185.5051705 | 228.9507791 | 276.3774931 | 286.9391929 | 479.404517 | 319.9859364 |
| MA3180 | 29.00551713 | 35.79865578 | 43.21427855 | 107.9564291 | 180.3685276 | 120.3897547 |
| MA3179 | 364.5260849 | 449.8986788 | 543.0943259 | 804.7001716 | 1344.455225 | 897.3773687 |
| MA3178 | 13.81090786 | 17.04544464 | 20.57637575 | 93.72786451 | 156.5961107 | 104.5224885 |
| MA3177 | 113.800043 | 140.4521957 | 169.5465982 | 101.4513865 | 169.5002082 | 113.1355274 |
| MA3176 | 230.8470729 | 284.9118277 | 343.9307655 | 909.5146719 | 1519.574365 | 1014.263339 |
| MA3175 | 108.3790636 | 133.7616142 | 161.4700756 | 318.4810795 | 532.1032186 | 355.1604973 |
| MA3174 | 410.1109566 | 506.1596006 | 611.0095895 | 402.3068643 | 672.1553999 | 448.640485 |
| MA3173 | 430.1722134 | 530.9192359 | 640.8981357 | 2581.734409 | 4313.440505 | 2879.07237 |
| MA3172 | 460.5942602 | 568.4661749 | 686.222851 | 2343.78896 | 3915.892434 | 2613.722781 |
| MA3171 | 299.5807832 | 369.7430833 | 446.3346527 | 999.909558 | 1670.601892 | 1115.068991 |
| MA3170a | 83.01724546 | 102.4600175 | 123.6844133 | 306.1291472 | 511.4661906 | 341.3859948 |
| MA3170 | 161.8172393 | 199.7150963 | 241.0856949 | 440.0482474 | 735.211929 | 490.7285376 |
| MA3169 | 3928.752725 | 4848.872917 | 5853.307626 | 3164.703078 | 5287.437158 | 3529.181453 |
| MA3168 | 3782.751295 | 4668.677718 | 5635.785338 | 2492.167004 | 4163.795495 | 2779.189501 |
| MA3167 | 1223.669336 | 1510.254658 | 1823.101009 | 2290.955297 | 3827.620433 | 2554.804273 |
| MA3166 | 330.9580203 | 408.4689196 | 493.0824716 | 763.0529597 | 1274.873021 | 850.9336538 |
| MA3165 | 11794.09385 | 14556.28955 | 17571.59697 | 3828.872975 | 6397.101005 | 4269.843697 |
| MA3164 | 3073.877975 | 3793.785128 | 4579.660429 | 759.8023683 | 1269.442085 | 847.3086922 |
| MA3163 | 6016.846766 | 7426.001932 | 8964.283966 | 1923.672826 | 3213.982142 | 2145.221934 |
| MA3162 | 4160.35602 | 5134.718075 | 6198.365059 | 1453.43803 | 2428.335948 | 1620.830269 |
| MA3161 | 2254.412492 | 2782.399514 | 3358.768229 | 687.8812915 | 1149.279731 | 767.1044759 |
| MA3160 | 1535.311765 | 1894.884244 | 2287.405874 | 601.4303616 | 1004.84158 | 670.6970055 |
| MA3159 | 271.941115 | 335.6301604 | 405.1553034 | 397.296586 | 663.7844625 | 443.0531738 |
| MA3157 | 53.8441107 | 66.45448781 | 80.2204073 | 120.6907254 | 201.6443913 | 134.590658 |
| MA3156 | 89.12282866 | 109.9955381 | 132.7809025 | 223.6549075 | 373.6721074 | 249.4132094 |
| MA3155 | 299.8399513 | 370.062949 | 446.720778 | 3170.662788 | 5297.394361 | 3535.827542 |
| MA3154 | 376.5563961 | 464.7465082 | 561.0178546 | 2386.266586 | 3986.862055 | 2661.092549 |
| MA3153 | 691.2062858 | 853.0879069 | 1029.803428 | 4002.758321 | 6687.620468 | 4463.755392 |
| MA3152 | 1482.466873 | 1829.662993 | 2208.674167 | 1327.341207 | 2217.659303 | 1480.210894 |
| MA3151 | 209.1376428 | 258.1180143 | 311.5866651 | 511.3757557 | 854.3825773 | 570.2708242 |
| MA3150 | 456.9853582 | 564.0120622 | 680.8460774 | 1900.198578 | 3174.762472 | 2119.044161 |
| MA3149 | 1234.910732 | 1524.128806 | 1839.849161 | 5687.905612 | 9503.085357 | 6342.980841 |
| MA3148 | 1252.149766 | 1545.40525 | 1865.532978 | 7030.012698 | 11745.4148 | 7839.658198 |
| MA3147 | 676.3474895 | 834.7491567 | 1007.665841 | 4606.031503 | 7695.540946 | 5136.507455 |
| MA3146 | 856.6845261 | 1057.321417 | 1276.343517 | 5770.834197 | 9641.638539 | 6435.460299 |
| MA3145 | 1059.798273 | 1308.004729 | 1578.955396 | 5781.987605 | 9660.27313 | 6447.898244 |
| MA3144 | 9.8320848 | 12.13477483 | 14.64847013 | 67.79186721 | 113.2634654 | 75.59944631 |
| MA3143 | 150.0531197 | 185.1958011 | 223.5587555 | 384.9877357 | 643.2194139 | 429.326715 |
| MA3142 | 875.4388821 | 1080.468073 | 1304.284958 | 3731.166489 | 6233.857601 | 4160.88437 |
| MA3141 | 123.1035392 | 151.9345856 | 183.4075431 | 739.3543912 | 1235.278566 | 824.5057246 |
| MA3140 | 1648.656473 | 2034.774464 | 2456.274085 | 8881.8558 | 14839.38721 | 9904.77779 |
| MA3139 | 192.3723713 | 237.4262893 | 286.608689 | 1904.45827 | 3181.879366 | 2123.794442 |
| MA3138 | 305.2940917 | 376.7944577 | 454.8467058 | 2404.273439 | 4016.947058 | 2681.173248 |
| MA3137 | 372.3473668 | 459.5517174 | 554.7469729 | 2893.173063 | 4833.77757 | 3226.379367 |
| MA3136 | 878.0215583 | 1083.655616 | 1308.132795 | 9109.273875 | 15219.34664 | 10158.38757 |
| MA3135 | 140.5447258 | 173.4605261 | 209.3925408 | 420.7515339 | 702.9718871 | 469.2094245 |
| MA3134 | 170.0716462 | 209.9026986 | 253.3836394 | 134.2997126 | 224.3816476 | 149.7669902 |
| MA3133 | 1597.65429 | 1971.827489 | 2380.287765 | 4974.886846 | 8311.807116 | 5547.843812 |
| MA3132 | 52.32516971 | 64.57980841 | 77.95739166 | 355.2420945 | 593.5217947 | 396.1552729 |
| MA3131 | 22.12197182 | 27.30297311 | 32.95873153 | 48.4274213 | 80.91025936 | 54.00480009 |
| MA3130 | 33246.34251 | 41032.68926 | 49532.53201 | 17556.21017 | 29332.09078 | 19578.15624 |
| MA3129 | 3432.744454 | 4236.698712 | 5114.322711 | 14606.27653 | 24403.48031 | 16288.47919 |
| MA3128 | 2417.038869 | 2983.11325 | 3601.059429 | 28503.46353 | 47622.24717 | 31786.20312 |
| MA3127 | 1059.055445 | 1307.087929 | 1577.848682 | 12169.61305 | 20332.4175 | 13571.18555 |
| MA3126 | 700.9077773 | 865.0615033 | 1044.257332 | 7275.747715 | 12155.97731 | 8113.69448 |
| MA3125 | 30.02364694 | 37.05523322 | 44.73115359 | 245.9287229 | 410.8861513 | 274.2522967 |
| MA3124 | 34.43486302 | 42.49956318 | 51.30326605 | 116.9179714 | 195.3410514 | 130.3833965 |
| MA3123 | 61.4781788 | 75.87646691 | 91.59413127 | 270.290876 | 451.5892917 | 301.4202354 |
| MA3122 | 247.0964487 | 304.9668334 | 368.1401271 | 824.2455391 | 1377.110706 | 919.1737732 |
| MA3121 | 201.732888 | 248.979054 | 300.5545867 | 1128.347383 | 1885.189774 | 1258.298981 |
| MA3120 | 462.5718133 | 570.9068741 | 689.1691363 | 1272.261014 | 2125.633904 | 1418.787123 |
| MA3119 | 65.65371773 | 81.02992375 | 97.81511679 | 277.8473314 | 464.2142622 | 309.8469666 |
| MA3118 | 286.4043439 | 353.4807007 | 426.7035488 | 1235.797354 | 2064.712135 | 1378.123949 |
| MA3117 | 30.62704607 | 37.79994939 | 45.63013629 | 258.9520238 | 432.6448703 | 288.7754892 |
| MA3116a | 5.115458077 | 6.313506566 | 7.621337322 | 36.36238557 | 60.75256473 | 40.55023602 |
| MA3116 | 5.390590427 | 6.653075353 | 8.031247131 | 76.14911227 | 127.2263577 | 84.91919402 |
| MA3114 | 70.51297755 | 87.02723001 | 105.0547535 | 263.2325773 | 439.7966179 | 293.5490335 |
| MA3113 | 1406.611577 | 1736.042264 | 2095.660084 | 1314.127061 | 2195.581728 | 1465.474876 |
| MA3112 | 128.9872514 | 159.1962726 | 192.1734746 | 206.9738443 | 345.802171 | 230.8109907 |
| MA3110 | 8.996671624 | 11.10370655 | 13.40381803 | 39.90332026 | 66.66859199 | 44.49897963 |
| MA3109 | 201.6439089 | 248.8692359 | 300.42202 | 1783.267477 | 2979.399485 | 1988.646123 |
| MA3108 | 740.6123905 | 914.0650006 | 1103.411809 | 4370.164792 | 7301.465932 | 4873.476011 |
| MA3107 | 134.4934295 | 165.9920065 | 200.3769317 | 414.8298396 | 693.0781984 | 462.6057295 |
| MA3105 | 402.9973613 | 497.3799898 | 600.4112992 | 252.5797536 | 421.9983808 | 281.6693257 |
| MA3104 | 149.3289366 | 184.3020132 | 222.4798211 | 1722.591231 | 2878.024465 | 1920.981803 |
| MA3103 | 17.60926794 | 21.73338675 | 26.23541606 | 130.8583039 | 218.6319036 | 145.9292349 |
| MA3102 | 42.29754514 | 52.20369807 | 63.01759383 | 128.8265962 | 215.2374219 | 143.6635358 |
| MA3101 | 260.7239963 | 321.7859746 | 388.443321 | 875.4966712 | 1462.738689 | 976.3274905 |
| MA3100 | 55.01818154 | 67.90352792 | 81.96961328 | 429.2454492 | 717.1631214 | 478.6815827 |
| MA3099 | 74.44512058 | 91.88028726 | 110.9131123 | 1297.834739 | 2168.361282 | 1447.30617 |
| MA3098 | 46.77801174 | 57.73349714 | 69.69288016 | 268.1001899 | 447.9291962 | 298.9772482 |
| MA3097 | 27.41417512 | 33.83461892 | 40.84339523 | 133.5602793 | 223.1462371 | 148.9423965 |
| MA3096 | 41.79100957 | 51.57853106 | 62.26292468 | 327.7311893 | 547.5578673 | 365.4759409 |
| MA3095 | 24.1983406 | 29.86563079 | 36.05223884 | 64.46077267 | 107.6980292 | 71.88471012 |
| MA3094 | 167.3928255 | 206.5964937 | 249.3925605 | 802.1074975 | 1340.123507 | 894.4860968 |
| MA3093 | 119.6323069 | 147.6503851 | 178.2358787 | 530.6674486 | 886.614231 | 591.7843384 |
| MA3092 | 92.0568554 | 113.6167186 | 137.1522036 | 297.7043801 | 497.3904858 | 331.9909486 |
| MA3091 | 184.3723981 | 227.5527095 | 274.6898162 | 981.8051672 | 1640.353927 | 1094.87952 |
| MA3090 | 8.664220675 | 10.69339506 | 12.9085113 | 40.69951224 | 67.99883212 | 45.38686891 |
| MA3089 | 33.00633244 | 40.73646845 | 49.17494964 | 153.7584025 | 256.8923121 | 171.4667345 |
| MA3088 | 124.3824926 | 153.513072 | 185.3130099 | 668.800549 | 1117.400523 | 745.8262072 |
| MA3087 | 726.4921179 | 896.6377375 | 1082.374522 | 2112.483203 | 3529.437647 | 2355.777575 |
| MA3086 | 131.8973047 | 162.7878651 | 196.5090586 | 790.3452986 | 1320.471778 | 881.3692472 |
| MA3085 | 626.4385088 | 773.1514126 | 933.3082424 | 3738.928277 | 6246.825631 | 4169.540082 |
| MA3084a | 1338.542847 | 1652.031729 | 1994.246928 | 1031.968494 | 1724.164455 | 1150.820149 |
| MA3084 | 101.2418656 | 124.9528729 | 150.8366205 | 743.4900614 | 1242.18825 | 829.1176994 |
| MA3083 | 144.4872411 | 178.3263848 | 215.2663528 | 427.0824276 | 713.5492466 | 476.2694462 |
| MA3082 | 86.75519607 | 107.0734022 | 129.2534517 | 365.5603841 | 610.761108 | 407.6619183 |
| MA3081 | 48.85712659 | 60.29954402 | 72.79047872 | 232.4140712 | 388.3065064 | 259.1811647 |
| MA3080 | 15.29041917 | 18.87145987 | 22.78064652 | 87.02579846 | 145.3986138 | 97.04854652 |
| MA3079 | 12.23548229 | 15.10105187 | 18.22920575 | 79.16988922 | 132.2733298 | 88.28787339 |
| MA3078 | 25.92451429 | 31.99607713 | 38.62400304 | 108.9468649 | 182.0233012 | 121.4942588 |
| MA3077 | 18.42472166 | 22.73982103 | 27.45033127 | 66.63123345 | 111.3243331 | 74.30514253 |
| MA3076 | 745.4698952 | 920.0601406 | 1110.648831 | 1307.617691 | 2184.706179 | 1458.215823 |
| MA3075 | 4262.636809 | 5260.953188 | 6350.749535 | 7043.377141 | 11767.74348 | 7854.561821 |
| MA3074 | 295.1683763 | 364.2972836 | 439.760766 | 273.3129635 | 456.6384534 | 304.7903762 |
| MA3073 | 1411.130274 | 1741.619248 | 2102.39233 | 5040.043359 | 8420.667556 | 5620.504391 |
| MA3072 | 649.8536679 | 802.0504396 | 968.1936473 | 5904.574443 | 9865.085456 | 6584.603389 |
| MA3071 | 259.9290142 | 320.8048064 | 387.2589057 | 2107.644136 | 3521.35276 | 2350.381192 |
| MA3070 | 27.0975472 | 33.44383623 | 40.37166266 | 224.1423336 | 374.4864761 | 249.9567723 |
| MA3069 | 39.32254737 | 48.53195105 | 58.58525147 | 492.8981756 | 823.5111049 | 549.665184 |
| MA3068 | 45.05252254 | 55.6038956 | 67.12213576 | 510.1165173 | 852.2787009 | 568.8665595 |
| MA3067 | 92.14749244 | 113.728583 | 137.2872405 | 278.6351683 | 465.5305429 | 310.7255385 |
| MA3066 | 270.6080463 | 333.9848848 | 403.1692121 | 1098.441209 | 1835.223943 | 1224.948517 |
| MA3065 | 108.8738877 | 134.3723269 | 162.2072963 | 564.7502718 | 943.5582101 | 629.7924752 |
| MA3064 | 80.15486925 | 98.92726823 | 119.4198618 | 388.7566148 | 649.5162799 | 433.5296553 |
| MA3063 | 43.28476627 | 53.42212797 | 64.48841915 | 234.2178618 | 391.3201949 | 261.1926975 |
| MA3062 | 8.93526742 | 11.02792139 | 13.31233411 | 99.39170847 | 166.058995 | 110.8386365 |
| MA3061 | 49.68699571 | 61.32376982 | 74.02687093 | 129.9001699 | 217.0310983 | 144.8607528 |
| MA3060 | 66.0594451 | 81.53067312 | 98.41959543 | 107.5264817 | 179.6501917 | 119.9102903 |
| MA3059 | 22.32504089 | 27.55360129 | 33.26127686 | 100.4411223 | 167.8123063 | 112.0089112 |
| MA3058 | 29.04756527 | 35.85055166 | 43.27692457 | 75.18451463 | 125.6147533 | 83.84350381 |
| MA3057 | 11.66166244 | 14.39284249 | 17.37429217 | 88.60947922 | 148.0445531 | 98.81461955 |
| MA3056 | 187.2758925 | 231.1362069 | 279.0156282 | 796.1440016 | 1330.159978 | 887.8357859 |
| MA3055 | 124.0166541 | 153.0615535 | 184.7679602 | 620.1483046 | 1036.11464 | 691.5706911 |
| MA3054 | 54.94494385 | 67.81313785 | 81.86049908 | 251.4920551 | 420.1811052 | 280.456357 |
| MA3052 | 1385.716911 | 1710.254034 | 2064.529872 | 18319.44262 | 30607.2637 | 20429.29006 |
| MA3051 | 70.61512511 | 87.15330071 | 105.2069395 | 217.9317226 | 364.110079 | 243.0308861 |
| MA3050 | 36.4583532 | 44.99695801 | 54.31799142 | 202.6119751 | 338.5145651 | 225.9467657 |
| MA3049 | 69.96698981 | 86.35337108 | 104.241306 | 325.6491635 | 544.0793165 | 363.1541285 |
| MA3048 | 205.0131887 | 253.0276064 | 305.4417889 | 1782.891061 | 2978.770587 | 1988.226355 |
| MA3047 | 172.9347908 | 213.4363963 | 257.6493357 | 647.2091878 | 1081.326691 | 721.7481721 |
| MA3046 | 164.1870784 | 202.6399555 | 244.6164331 | 853.8553501 | 1426.581387 | 952.1937417 |
| MA3045 | 406.541174 | 501.7537692 | 605.6910986 | 1430.934772 | 2390.738562 | 1595.735314 |
| MA3044 | 527.2569262 | 650.7413442 | 785.5411636 | 1911.470574 | 3193.595194 | 2131.614352 |
| MA3043 | 2307.125365 | 2847.457827 | 3437.303246 | 4035.434237 | 6742.213852 | 4500.194588 |
| MA3042 | 2094.783458 | 2585.385104 | 3120.942662 | 3731.607698 | 6234.594752 | 4161.376393 |
| MA3041 | 12.24326945 | 15.1106628 | 18.24080757 | 50.19027794 | 83.85555738 | 55.97068467 |
| MA3040 | 198.8895926 | 245.4698543 | 296.318463 | 1371.9807 | 2292.240869 | 1529.99151 |
| MA3039 | 177.6519352 | 219.2583034 | 264.6772397 | 623.7234823 | 1042.087879 | 695.5576215 |
| MA3038 | 261.9961932 | 323.356122 | 390.3387215 | 1200.82447 | 2006.281084 | 1339.123243 |
| MA3036 | 469.1999292 | 579.0873054 | 699.0441282 | 2767.284662 | 4623.449147 | 3085.992418 |
| MA3035 | 1150.31257 | 1419.717619 | 1713.809397 | 4875.954878 | 8146.516235 | 5437.517865 |
| MA3034a | 13.18610861 | 16.2743164 | 19.64550978 | 144.4757331 | 241.3832643 | 161.1149814 |
| MA3034 | 540.7449765 | 667.3883175 | 805.6365254 | 4061.548204 | 6785.843841 | 4529.316096 |
| MA3033 | 677.3969752 | 836.0444336 | 1009.229432 | 5180.404917 | 8655.17705 | 5777.031368 |
| MA3032 | 85.68402748 | 105.7513642 | 127.6575561 | 230.9901666 | 385.9275136 | 257.5932692 |
| MA3031 | 193.4001552 | 238.6947819 | 288.1399474 | 1466.743761 | 2450.566537 | 1635.668419 |
| MA3030 | 1071.449296 | 1322.38444 | 1596.313837 | 11043.30673 | 18450.63783 | 12315.16269 |
| MA3029 | 4449.729448 | 5491.863223 | 6629.492139 | 8763.465753 | 14641.58669 | 9772.752779 |
| MA3028 | 280.8760037 | 346.6576144 | 418.4670732 | 1296.394315 | 2165.954689 | 1445.699852 |
| MA3027 | 15.62643479 | 19.28617089 | 23.28126414 | 54.00167142 | 90.22345446 | 60.22103575 |
| MA3026 | 2544.89252 | 3140.910431 | 3791.543993 | 5686.90363 | 9501.411294 | 6341.863462 |
| MA3025 | 1270.240649 | 1567.733047 | 1892.485936 | 8922.891016 | 14907.94692 | 9950.539025 |
| MA3024 | 1915.056401 | 2363.565683 | 2853.173773 | 12186.74898 | 20361.0474 | 13590.29502 |
| MA3023 | 2831.010286 | 3494.037437 | 4217.820579 | 13374.88604 | 22346.13096 | 14915.26964 |
| MA3022 | 3158.476337 | 3898.196563 | 4705.700492 | 15031.88635 | 25114.56919 | 16763.10643 |
| MA3021 | 521.815976 | 644.0261148 | 777.4348872 | 1518.26293 | 2536.642343 | 1693.121042 |
| MA3020 | 856.3132516 | 1056.863189 | 1275.790368 | 4316.610023 | 7211.98914 | 4813.753347 |
| MA3019 | 4855.498748 | 5992.664345 | 7234.033251 | 11450.19367 | 19130.44541 | 12768.91074 |
| MA3018 | 1943.149606 | 2398.238361 | 2895.02883 | 5584.548114 | 9330.400509 | 6227.719677 |
| MA3017 | 800.4801505 | 987.953886 | 1192.606636 | 694.6118955 | 1160.524907 | 774.6102425 |
| MA3016 | 575.2529262 | 709.9780844 | 857.0486806 | 1976.546756 | 3302.321419 | 2204.185347 |
| MA3015 | 4294.597089 | 5300.398617 | 6398.366008 | 8150.28694 | 13617.11635 | 9088.954255 |
| MA3014 | 2174.135794 | 2683.321885 | 3239.166859 | 5389.001605 | 9003.690591 | 6009.652105 |
| MA3013 | 2842.974178 | 3508.803292 | 4235.645153 | 18110.74591 | 30258.58304 | 20196.55778 |
| MA3012 | 859.7784535 | 1061.139947 | 1280.953048 | 6259.255173 | 10457.66935 | 6980.132645 |
| MA3011 | 50.29169467 | 62.07009025 | 74.9277902 | 279.2513779 | 466.5600771 | 311.4127169 |
| MA3010 | 812.0639346 | 1002.250611 | 1209.8649 | 3603.418448 | 6020.422178 | 4018.423606 |
| MA3009 | 549.1457903 | 677.7566154 | 818.1525962 | 2902.808821 | 4849.876542 | 3237.124874 |
| MA3008 | 92.45225246 | 114.1047183 | 137.7412915 | 1639.321685 | 2738.90162 | 1828.122115 |
| MA3007 | 80.93076067 | 99.88487466 | 120.5758346 | 1497.744917 | 2502.361811 | 1670.239972 |
| MA3006 | 41.10038763 | 50.72616436 | 61.23399186 | 1945.608621 | 3250.631437 | 2169.684071 |
| MA3004n | 176.18754 | 217.4509445 | 262.49549 | 844.8156924 | 1411.478351 | 942.112988 |
| MA3003 | 517.3699046 | 638.5387663 | 770.8108451 | 578.3818995 | 966.3332929 | 644.9940554 |
| MA3002 | 40.24603751 | 49.67172406 | 59.96112627 | 298.7866786 | 499.1987393 | 333.1978952 |
| MA3001 | 162.3238085 | 200.3403048 | 241.8404141 | 617.4970671 | 1031.685077 | 688.614111 |
| MA3000 | 23.61034707 | 29.13992823 | 35.17620838 | 181.0979749 | 302.5699847 | 201.9550014 |
| MA2999 | 83.41407086 | 102.9497801 | 124.2756293 | 315.782632 | 527.5947793 | 352.15127 |
| MA2998 | 19.13658631 | 23.61840553 | 28.51091286 | 37.7386424 | 63.05194995 | 42.08499614 |
| MA2997 | 143.3377498 | 176.9076808 | 213.553767 | 118.5704407 | 198.1019194 | 132.2261804 |
| MA2995 | 27.74644924 | 34.24471216 | 41.3384385 | 35.3035548 | 58.98352005 | 39.36945987 |
| MA2994 | 277.5509609 | 342.5538413 | 413.5132114 | 605.0869351 | 1010.950811 | 674.7747059 |
| MA2993 | 21.69289306 | 26.77340342 | 32.3194625 | 69.86215369 | 116.7224028 | 77.90816737 |
| MA2992 | 683.7725992 | 843.913239 | 1018.728245 | 3327.977842 | 5560.228959 | 3711.260547 |
| MA2991 | 649.0365863 | 801.0419961 | 966.9763067 | 4886.21103 | 8163.651731 | 5448.955216 |
| MA2990 | 317.6801942 | 392.0814054 | 473.3003153 | 3127.414419 | 5225.137019 | 3487.598265 |
| MA2989 | 229.7030006 | 283.4998118 | 342.2262532 | 2023.571139 | 3380.887549 | 2256.625521 |
| MA2988 | 142.6890308 | 176.1070307 | 212.5872638 | 1018.099879 | 1700.993426 | 1135.354289 |
| MA2987 | 450.0711974 | 555.4785939 | 670.5449175 | 2720.176137 | 4544.742438 | 3033.458412 |
| MA2986 | 394.0934375 | 486.3907528 | 587.1456628 | 656.6330595 | 1097.071653 | 732.2573897 |
| MA2985 | 5634.934959 | 6954.645767 | 8395.287278 | 8699.520454 | 14534.74989 | 9701.44291 |
| MA2984 | 1298.600289 | 1602.73456 | 1934.737945 | 1045.593237 | 1746.928036 | 1166.014052 |
| MA2982 | 1328.373708 | 1639.480961 | 1979.096293 | 1743.063773 | 2912.229025 | 1943.812164 |
| MA2981 | 40.87641227 | 50.4497336 | 60.900299 | 243.7961707 | 407.3231833 | 271.8741388 |
| MA2980 | 448.7700943 | 553.8727704 | 668.6064508 | 997.3719012 | 1666.362095 | 1112.239072 |
| MA2979 | 196.7245754 | 242.7977866 | 293.0928817 | 1139.667531 | 1904.102946 | 1270.922869 |
| MA2977 | 32.95777331 | 40.67653669 | 49.10260312 | 25.2402981 | 42.17030374 | 28.14721942 |
| MA2976 | 323.718339 | 399.5336933 | 482.2963303 | 315.581412 | 527.2585904 | 351.9268755 |
| MA2975 | 3434.342813 | 4238.67141 | 5116.704049 | 2310.123331 | 3859.645483 | 2576.179886 |
| MA2974 | 55.6030768 | 68.62540659 | 82.84102773 | 59.33041918 | 99.12647576 | 66.16349459 |
| MA2973 | 89.88509956 | 110.9363341 | 133.9165826 | 192.8777783 | 322.2511265 | 215.0914829 |
| MA2972 | 363.4744086 | 448.6006982 | 541.5274711 | 3254.061592 | 5436.733163 | 3628.831374 |
| MA2971 | 120.3354061 | 148.5181513 | 179.283401 | 1273.900642 | 2128.373317 | 1420.615586 |
| MA2970 | 80.24140561 | 99.03407154 | 119.5487892 | 134.5378319 | 224.7794861 | 150.0325337 |
| MA2969 | 442.0621053 | 545.5937596 | 658.6124588 | 600.6719559 | 1003.574472 | 669.8512542 |
| MA2968 | 925.9948123 | 1142.864283 | 1379.606424 | 5716.953904 | 9551.617879 | 6375.374621 |
| MA2967 | 105.5569329 | 130.2785359 | 157.2654844 | 819.8422927 | 1369.753969 | 914.2634057 |
| MA2966 | 185.9483268 | 229.4977232 | 277.0377357 | 729.4862667 | 1218.791368 | 813.5010898 |
| MA2965 | 113.8619675 | 140.5286229 | 169.6388572 | 432.3227074 | 722.3044598 | 482.1132483 |
| MA2964 | 5653.161958 | 6977.141558 | 8422.443028 | 3168.39009 | 5293.597244 | 3533.293097 |
| MA2963 | 617.9171633 | 762.6343543 | 920.6125957 | 772.4589833 | 1290.588163 | 861.4229676 |
| MA2962 | 1861.223034 | 2297.124455 | 2772.969372 | 2012.889172 | 3363.04062 | 2244.713314 |
| MA2961 | 789.8873059 | 974.8801802 | 1176.824737 | 1745.701125 | 2916.63539 | 1946.75326 |
| MA2960 | 29.70522481 | 36.66223612 | 44.2567479 | 126.007605 | 210.527584 | 140.5198819 |
| MA2959 | 3493.39791 | 4311.557305 | 5204.688118 | 30889.78553 | 51609.20181 | 34447.35746 |
| MA2958 | 24.85142154 | 30.67166433 | 37.02524068 | 65.54362093 | 109.5072012 | 73.09226985 |
| MA2957 | 104.1605521 | 128.5551205 | 155.1850668 | 379.8917421 | 634.7052674 | 423.6438167 |
| MA2956 | 10.5934353 | 13.07443485 | 15.78277891 | 72.57076637 | 121.2478255 | 80.92873057 |
| MA2953 | 190.2817643 | 234.8460587 | 283.4939687 | 752.9793994 | 1258.04259 | 839.699923 |
| MA2952 | 157.9743431 | 194.9721877 | 235.3603018 | 811.7341191 | 1356.207214 | 905.2214149 |
| MA2951 | 464.3330937 | 573.0806492 | 691.7932047 | 1217.936125 | 2034.870434 | 1358.205645 |
| MA2950 | 119.7237726 | 147.7632722 | 178.3721502 | 414.5241783 | 692.5675139 | 462.2648652 |
| MA2949 | 115.4479111 | 142.4859971 | 172.0016977 | 343.6970348 | 574.2328516 | 383.2805704 |
| MA2948 | 812.7431099 | 1003.08885 | 1210.876778 | 1223.661547 | 2044.436199 | 1364.590462 |
| MA2946 | 87.61816034 | 108.1384741 | 130.5391512 | 568.2655351 | 949.4313469 | 633.7125909 |
| MA2945 | 67.89942038 | 83.80157356 | 101.1609085 | 670.5556602 | 1120.33288 | 747.7834542 |
| MA2944 | 18.00327338 | 22.21966889 | 26.82243062 | 90.2696444 | 150.8182791 | 100.6659857 |
| MA2943a | 9.515998163 | 11.7446602 | 14.177544 | 76.67727238 | 128.1087828 | 85.50818226 |
| MA2943 | 25.32698317 | 31.25860326 | 37.73376287 | 247.9813208 | 414.3155354 | 276.541292 |
| MA2942 | 176.4826769 | 217.815203 | 262.9352038 | 1510.097078 | 2522.999222 | 1684.014731 |
| MA2941 | 16.16637503 | 19.952566 | 24.08570172 | 104.7604371 | 175.0288145 | 116.8256808 |
| MA2939 | 360.4589619 | 444.8790291 | 537.0348654 | 1352.638013 | 2259.924016 | 1508.421128 |
| MA2938 | 228.4302662 | 281.929001 | 340.3300519 | 1449.197894 | 2421.251728 | 1616.101797 |
| MA2937 | 139.2361523 | 171.8454825 | 207.4429441 | 522.8340548 | 873.5265649 | 583.0487739 |
| MA2936 | 462.2643037 | 570.5273453 | 688.7109889 | 1248.179132 | 2085.399027 | 1391.931733 |
| MA2935 | 761.8496123 | 940.2760137 | 1135.052384 | 3669.707082 | 6131.174112 | 4092.346692 |
| MA2934 | 902.396859 | 1113.739651 | 1344.44868 | 1453.700598 | 2428.774633 | 1621.123076 |
| MA2933 | 1138.057384 | 1404.592249 | 1695.550835 | 4321.184505 | 7219.63197 | 4818.854671 |
| MA2932 | 35.91294714 | 44.32381697 | 53.50541051 | 113.8191453 | 190.1636783 | 126.9276791 |
| MA2930 | 19.92373651 | 24.58990757 | 29.68365968 | 35.54803676 | 59.39198902 | 39.64209878 |
| MA2929 | 301.45983 | 372.0622058 | 449.134177 | 161.7594072 | 270.2600147 | 180.3892137 |
| MA2926 | 324.1516387 | 400.0684725 | 482.941888 | 1113.447642 | 1860.295987 | 1241.683239 |
| MA2925 | 461.9781363 | 570.1741571 | 688.2846383 | 1548.417997 | 2587.024011 | 1726.749063 |
| MA2923 | 558.844129 | 689.7263206 | 832.6018029 | 504.7068572 | 843.2404952 | 562.8338697 |
| MA2922 | 139.3178132 | 171.9462685 | 207.5646077 | 1120.555611 | 1872.171647 | 1249.609831 |
| MA2921 | 49.28155532 | 60.82337464 | 73.42281985 | 241.4875762 | 403.466092 | 269.2996638 |
| MA2920 | 228.7028926 | 282.265477 | 340.7362283 | 1311.551868 | 2191.279216 | 1462.603098 |
| MA2919 | 243.7946673 | 300.8917695 | 363.2209216 | 1111.080482 | 1856.341047 | 1239.043453 |
| MA2918 | 57.11621211 | 70.49292062 | 85.0953937 | 263.4401906 | 440.1434885 | 293.7805576 |
| MA2917 | 177.6764632 | 219.288576 | 264.7137832 | 401.8964071 | 671.4696274 | 448.1827556 |
| MA2916 | 3671.944227 | 4531.919456 | 5470.697865 | 6616.78754 | 11055.01762 | 7378.841961 |
| MA2914 | 218.8281622 | 270.0780689 | 326.024222 | 4646.422989 | 7763.025142 | 5181.550822 |
| MA2913 | 68.13352799 | 84.09050956 | 101.5096971 | 2152.190333 | 3595.778453 | 2400.057768 |
| MA2912 | 701.9479411 | 866.345275 | 1045.807034 | 16738.82486 | 27966.44183 | 18666.63279 |
| MA2911 | 257.1348076 | 317.356192 | 383.0959175 | 2408.484685 | 4023.983009 | 2685.869502 |
| MA2910 | 1558.20028 | 1923.133285 | 2321.506651 | 12689.49047 | 21201.00426 | 14150.93717 |
| MA2909 | 1326.530381 | 1637.205924 | 1976.349986 | 10271.23983 | 17160.70475 | 11454.17697 |
| MA2908 | 264.6593049 | 326.6429387 | 394.3063959 | 1042.691616 | 1742.080145 | 1162.778252 |
| MA2907 | 291.4053845 | 359.652993 | 434.1544196 | 1014.92832 | 1695.694534 | 1131.817461 |
| MA2906 | 175.4883006 | 216.5879422 | 261.4537183 | 525.8428724 | 878.5535559 | 586.4041165 |
| MA2905 | 25.66260904 | 31.67283325 | 38.23379981 | 102.6276635 | 171.4654765 | 114.447276 |
| MA2904 | 103.113507 | 127.2628557 | 153.6251118 | 506.7596694 | 846.6702372 | 565.1231039 |
| MA2903 | 64.29982588 | 79.35894826 | 95.79800193 | 310.7853186 | 519.245503 | 346.5784167 |
| MA2902 | 65.00476959 | 80.22899092 | 96.84827227 | 189.0193622 | 315.8046662 | 210.7886936 |
| MA2901 | 90.57723946 | 111.7905743 | 134.947777 | 193.0814605 | 322.5914292 | 215.3186232 |
| MA2900 | 67.39446589 | 83.17835793 | 100.408595 | 201.4094404 | 336.5054264 | 224.6057352 |
| MA2899 | 302.5434817 | 373.3996505 | 450.7486708 | 978.1551295 | 1634.255616 | 1090.809108 |
| MA2898 | 2.759885617 | 3.406255256 | 4.111854489 | 73.01667004 | 121.9928204 | 81.42598889 |
| MA2897 | 7584.504165 | 9360.807208 | 11299.88044 | 10981.84155 | 18347.9447 | 12246.61858 |
| MA2896 | 1330.797858 | 1642.472851 | 1982.707947 | 13433.04651 | 22443.30275 | 14980.12845 |
| MA2895 | 845.0667481 | 1042.982736 | 1259.034606 | 1985.356557 | 3317.040421 | 2214.009771 |
| MA2894 | 2095.233621 | 2585.940697 | 3121.613345 | 6152.583349 | 10279.44708 | 6861.175444 |
| MA2893 | 451.2840968 | 556.9755563 | 672.3519728 | 2964.98729 | 4953.761406 | 3306.464428 |
| MA2892 | 710.4217488 | 876.8036621 | 1058.431856 | 1548.522614 | 2587.198801 | 1726.865729 |
| MA2891 | 14.7977473 | 18.26340346 | 22.0466324 | 132.926401 | 222.0871831 | 148.2355145 |
| MA2890 | 1740.637711 | 2148.297856 | 2593.31363 | 4209.893384 | 7033.691995 | 4694.746169 |
| MA2889 | 1805.07839 | 2227.830644 | 2689.321483 | 7849.060305 | 13113.84104 | 8753.035394 |
| MA2888 | 875.0708574 | 1080.013856 | 1303.736652 | 4253.274381 | 7106.170927 | 4743.123349 |
| MA2886 | 238.1192714 | 293.8871868 | 354.765353 | 1659.663062 | 2772.887038 | 1850.806206 |
| MA2885 | 718.5482267 | 886.8333742 | 1070.539204 | 2374.460958 | 3967.137766 | 2647.927268 |
| MA2884 | 44.4782907 | 54.89517774 | 66.26660835 | 143.0046758 | 238.9254909 | 159.4745027 |
| MA2883 | 137.0756293 | 169.1789616 | 204.2240585 | 426.2797277 | 712.2081334 | 475.3742995 |
| MA2882 | 344.1974498 | 424.8090448 | 512.8074223 | 310.6215778 | 518.9719325 | 346.3958179 |
| MA2881 | 59.35049566 | 73.25047696 | 88.42417254 | 193.9557953 | 324.0522266 | 216.2936549 |
| MA2880 | 35.63859862 | 43.98521559 | 53.09666851 | 57.88691954 | 96.7147444 | 64.55374731 |
| MA2879; | 469.8225665 | 579.8557653 | 699.9717731 | 613.6250645 | 1025.215917 | 684.2961704 |
| MA2878 | 3275.958423 | 4043.193142 | 4880.732833 | 3767.635988 | 6294.789125 | 4201.554055 |
| MA2877 | 2914.413721 | 3596.97409 | 4342.080362 | 3110.872093 | 5197.498877 | 3469.150761 |
| MA2876a | 1008.075596 | 1244.168518 | 1501.895636 | 750.369807 | 1253.682605 | 836.7897842 |
| MA2876 | 570.1534961 | 703.6843597 | 849.4512229 | 531.591216 | 888.1576182 | 592.814496 |
| MA2875 | 3007.310236 | 3711.627118 | 4480.483545 | 2431.273225 | 4062.057031 | 2711.282595 |
| MA2874 | 4286.450197 | 5290.34371 | 6386.228247 | 3800.519461 | 6349.729286 | 4238.224713 |
| MA4662 | 58.78347652 | 72.55066103 | 87.57939108 | 430.6938877 | 719.5831044 | 480.2968377 |
| MA2870 | 895.5121573 | 1105.242541 | 1334.19141 | 4295.510267 | 7176.736659 | 4790.223535 |
| MA2869 | 213.5314844 | 263.5409006 | 318.1328918 | 147.9471331 | 247.1831163 | 164.9861821 |
| MA2868 | 5621.768011 | 6938.395099 | 8375.670314 | 8810.658078 | 14720.4334 | 9825.380237 |
| MA2867 | 2195.596748 | 2709.809029 | 3271.140764 | 3023.654485 | 5051.779798 | 3371.888315 |
| MA2866 | 463.9305665 | 572.5838494 | 691.1934939 | 260.199626 | 434.7293054 | 290.1667776 |
| MA2865 | 4886.716026 | 6031.192759 | 7280.542753 | 501.4673403 | 837.8280625 | 559.2212581 |
| MA2864 | 678.7277137 | 837.6868332 | 1011.212051 | 78.925688 | 131.8653299 | 88.01554755 |
| MA2863 | 426.8801441 | 526.8561587 | 635.9933998 | 130.1751076 | 217.4904514 | 145.1673551 |
| MA2862 | 6746.9154 | 8327.053811 | 10051.98701 | 23073.4445 | 38550.02658 | 25730.80961 |
| MA2861 | 966.857144 | 1193.296638 | 1440.485744 | 2425.9907 | 4053.231237 | 2705.391682 |
| MA2860 | 1752.158179 | 2162.516435 | 2610.47756 | 2172.877825 | 3630.342143 | 2423.127836 |
| MA2859 | 352.7069662 | 435.3115036 | 525.4854453 | 698.7986463 | 1167.519934 | 779.2791807 |
| MA2857 | 219.2183348 | 270.5596205 | 326.6055261 | 827.9855231 | 1383.359296 | 923.3444906 |
| MA2856 | 334.2468921 | 412.5280505 | 497.9824437 | 1097.038622 | 1832.880567 | 1223.384394 |
| MA2855a | 44.43317709 | 54.83949845 | 66.1993952 | 71.71065324 | 119.8107889 | 79.96955833 |
| MA2854 | 25.15870776 | 31.05091749 | 37.48305539 | 74.91065332 | 125.1571987 | 83.53810194 |
| MA2853 | 115.0296368 | 141.9697623 | 171.3785258 | 261.4150195 | 436.7599277 | 291.5221477 |
| MA2852 | 55.40006961 | 68.3748548 | 82.53857462 | 214.0945982 | 357.699192 | 238.7518408 |
| MA2851 | 234.0265655 | 288.8359626 | 348.6677774 | 1746.958029 | 2918.735365 | 1948.154921 |
| MA2850 | 301.6219694 | 372.2623185 | 449.3757427 | 1241.800737 | 2074.742305 | 1384.81874 |
| MA2849 | 147.6171521 | 182.189326 | 219.9294949 | 165.0946464 | 275.8323755 | 184.1085719 |
| MA2848 | 112.8325251 | 139.2580834 | 168.1051279 | 489.5938482 | 817.9903901 | 545.9802977 |
| MA2847 | 952.413747 | 1175.470575 | 1418.967046 | 4824.450431 | 8060.465026 | 5380.081658 |
| MA2846 | 528.3524183 | 652.0934023 | 787.1732979 | 1365.781199 | 2281.883034 | 1523.078014 |
| MA4676 | 120.6320869 | 148.8843152 | 179.725415 | 66.59276227 | 111.2600573 | 74.26224064 |
| MA2845 | 234.9055853 | 289.9208502 | 349.9773973 | 644.6086606 | 1076.981853 | 718.8481426 |
| MA2844 | 143.0166404 | 176.5113671 | 213.0753577 | 318.8000262 | 532.6360997 | 355.516177 |
| MA2843 | 105.9713678 | 130.7900321 | 157.882936 | 362.2889606 | 605.2953673 | 404.0137254 |
| MA2842 | 357.3022605 | 440.9830232 | 532.3318093 | 1202.721763 | 2009.450992 | 1341.239047 |
| MA2841 | 153.8894212 | 189.9305706 | 229.2743234 | 497.9447648 | 831.9427091 | 555.2929882 |
| MA2840 | 70.59487842 | 87.12831221 | 105.1767747 | 394.6512646 | 659.3647839 | 440.1031911 |
| MA2839 | 221.1434217 | 272.9355659 | 329.473644 | 1997.975933 | 3338.124281 | 2228.082519 |
| MA2838 | 408.7086268 | 504.4288429 | 608.9203087 | 2612.850555 | 4365.4279 | 2913.772159 |
| MA2837 | 218.6101613 | 269.8090119 | 325.6994303 | 680.901346 | 1137.617966 | 759.3206511 |
| MA2836 | 718.3901932 | 886.638329 | 1070.303756 | 4719.375603 | 7884.910941 | 5262.905378 |
| MA2835 | 90.62453998 | 111.8489526 | 135.0182483 | 439.5994758 | 734.4621424 | 490.228081 |
| MA2834 | 215.9480408 | 266.5234185 | 321.7332326 | 890.3431577 | 1487.543501 | 992.8838446 |
| MA2833 | 234.0027164 | 288.806528 | 348.6322455 | 632.947457 | 1057.498862 | 705.84392 |
| MA2832 | 18.2509217 | 22.52531685 | 27.19139296 | 59.33591507 | 99.13565804 | 66.16962344 |
| MA2831 | 527.9401049 | 651.5845244 | 786.5590069 | 729.1652134 | 1218.254967 | 813.1430608 |
| MA2830 | 378.6908887 | 467.3809024 | 564.1979584 | 509.1812287 | 850.7160648 | 567.8235539 |
| MA2829 | 84.61379561 | 104.4304823 | 126.0630561 | 969.1807871 | 1619.261706 | 1080.801192 |
| MA2828 | 280.5080298 | 346.2034604 | 417.9188421 | 1894.025229 | 3164.44833 | 2112.159828 |
| MA2827 | 193.8676329 | 239.2717435 | 288.8364255 | 1652.827651 | 2761.466754 | 1843.183562 |
| MA2826 | 14215.55598 | 17544.862 | 21179.24646 | 22412.22155 | 37445.28637 | 24993.43372 |
| MA2825 | 287.1596079 | 354.4128488 | 427.8287896 | 625.9198973 | 1045.757545 | 698.0069973 |
| MA2824 | 40.62815242 | 50.1433309 | 60.53042557 | 286.3879739 | 478.4835662 | 319.3712336 |
| MA2820 | 1047.221008 | 1292.481848 | 1560.21698 | 6774.942602 | 11319.25567 | 7555.211718 |
| MA2819 | 133.8093205 | 165.1476781 | 199.3577023 | 453.163823 | 757.1248162 | 505.354632 |
| MA2818 | 24.82034014 | 30.63330362 | 36.97893361 | 55.89727427 | 93.39053861 | 62.3349549 |
| MA2817 | 192.0337122 | 237.0083157 | 286.1041329 | 644.332856 | 1076.521051 | 718.5405736 |
| MA2816 | 2153.425558 | 2657.761279 | 3208.311422 | 6443.890845 | 10766.14995 | 7186.03278 |
| MA2815 | 495.7705588 | 611.8808191 | 738.6307553 | 1309.516907 | 2187.879299 | 1460.333771 |
| MA2814 | 1863.382064 | 2299.789133 | 2776.186034 | 2798.312757 | 4675.28943 | 3120.594014 |
| MA2813 | 1031.999064 | 1273.6949 | 1537.538352 | 23614.97884 | 39454.79671 | 26334.71238 |
| MA2812 | 70.58277875 | 87.11337877 | 105.1587478 | 282.5461387 | 472.0648085 | 315.0869348 |
| MA2811 | 88.01730451 | 108.6310984 | 131.1338218 | 165.1669671 | 275.9532056 | 184.1892218 |
| MA2810 | 12.65073166 | 15.61355331 | 18.84787088 | 45.33238248 | 75.73921397 | 50.55330612 |
| MA2809 | 15.66824543 | 19.33777365 | 23.34355631 | 95.38351908 | 159.3623005 | 106.3688246 |
| MA2808 | 116.1311752 | 143.3292827 | 173.0196683 | 648.6626127 | 1083.755005 | 723.3689878 |
| MA4677 | 2.471505804 | 3.050336428 | 3.682207759 | 53.79777175 | 89.88278847 | 59.99365299 |
| MA2807 | 46.57288782 | 57.48033287 | 69.3872734 | 399.5156216 | 667.4919229 | 445.5277754 |
| MA2806 | 152.4082154 | 188.102464 | 227.067528 | 316.6797061 | 529.0935687 | 353.15166 |
| MA2805 | 18.0718717 | 22.30433304 | 26.92463278 | 82.73881355 | 138.2361208 | 92.2678302 |
| MA2804 | 53.738125 | 66.32368009 | 80.06250301 | 198.3551845 | 331.4025193 | 221.1997211 |
| MA2803 | 68.72602206 | 84.82176669 | 102.3924327 | 120.8494326 | 201.9095519 | 134.7676435 |
| MA2802 | 75.86293131 | 93.63015153 | 113.0254576 | 115.5752655 | 193.0977214 | 128.8860513 |
| MA2801 | 46.74267559 | 57.6898852 | 69.64023409 | 59.30495942 | 99.08393879 | 66.13510263 |
| MA2800 | 259.9269757 | 320.8022905 | 387.2558687 | 1688.833011 | 2821.622818 | 1883.335654 |
| MA2799 | 20.6353127 | 25.46813604 | 30.74381149 | 126.6138349 | 211.5404443 | 141.1959311 |
| MA2798 | 15.07048063 | 18.60001137 | 22.452968 | 64.43070735 | 107.6477975 | 71.85118218 |
| MA2796 | 605.8893661 | 747.7896277 | 902.6928125 | 4849.835118 | 8102.876566 | 5408.389895 |
| MA2795 | 69.98397471 | 86.37433387 | 104.2666112 | 419.6485182 | 701.1290202 | 467.9793746 |
| MA2794 | 640.4621606 | 790.4594262 | 954.2015777 | 2766.259527 | 4621.7364 | 3084.849218 |
| MA2793 | 1430.199448 | 1765.154453 | 2130.802807 | 2672.06078 | 4464.35356 | 2979.801616 |
| MA2792 | 177.0957871 | 218.5719046 | 263.8486547 | 1261.596984 | 2107.816945 | 1406.894918 |
| MA2791 | 275.6877541 | 340.2542684 | 410.7372864 | 635.0226112 | 1060.965932 | 708.1580693 |
| MA2790 | 760.1422287 | 938.1687582 | 1132.508614 | 1975.51103 | 3300.590978 | 2203.030337 |
| MA2789 | 23.07079411 | 28.47401111 | 34.37234781 | 100.7748905 | 168.3699505 | 112.3811194 |
| MA2788 | 146.3087126 | 180.5744478 | 217.9800978 | 1754.099714 | 2930.667357 | 1956.119113 |
| MA2787 | 81.46509491 | 100.5443508 | 121.37192 | 735.7060916 | 1229.183159 | 820.437251 |
| MA2785n | 82.53341401 | 101.862872 | 122.9635702 | 416.3681071 | 695.6482635 | 464.321159 |
| MA2784 | 885.6072699 | 1093.017913 | 1319.434474 | 2378.042515 | 3973.121663 | 2651.921312 |
| MA2783 | 230.6252311 | 284.6380302 | 343.6002513 | 1549.893735 | 2589.489606 | 1728.394762 |
| MA2782 | 563.3864607 | 695.3324737 | 839.3692598 | 2639.406948 | 4409.797073 | 2943.387047 |
| MA2781 | 106.7925642 | 131.8035541 | 159.1064071 | 192.0699145 | 320.9013857 | 214.1905775 |
| MA2780 | 73.67584596 | 90.93084729 | 109.7669976 | 190.6672361 | 318.5578565 | 212.6263529 |
| MA2779 | 71.22356315 | 87.9042359 | 106.1134295 | 123.4502543 | 206.2548826 | 137.6680014 |
| MA2778 | 32.99678954 | 40.72469059 | 49.160732 | 104.9394299 | 175.3278671 | 117.0252881 |
| MA2777 | 146.5903503 | 180.9220456 | 218.3996997 | 593.9617144 | 992.3633155 | 662.3681953 |
| MA2776 | 49.52715606 | 61.12649547 | 73.78873158 | 134.3180756 | 224.4123276 | 149.7874681 |
| MA2774n | 82.16231022 | 101.4048551 | 122.410676 | 200.0024949 | 334.1547682 | 223.0367521 |
| MA2773 | 31.75302618 | 39.18963586 | 47.30769364 | 96.93064469 | 161.9471653 | 108.0941324 |
| MA2772 | 41.96906558 | 51.79828808 | 62.52820394 | 195.2834908 | 326.2704779 | 217.7742609 |
| MA2771 | 469.4931481 | 579.4491966 | 699.4809845 | 2156.919984 | 3603.680531 | 2405.332132 |
| MA4670 | 7.223300474 | 8.915009033 | 10.76173603 | 51.92911306 | 86.76072137 | 57.90978116 |
| MA2770 | 24.26544132 | 29.94844661 | 36.15220979 | 98.93946655 | 165.3034104 | 110.33431 |
| MA2769 | 23.53231322 | 29.04361873 | 35.05994856 | 171.4230428 | 286.4055629 | 191.1658088 |
| MA2768 | 41.42543085 | 51.12733321 | 61.71826207 | 214.926474 | 359.0890509 | 239.6795235 |
| MA2766 | 104.9251979 | 129.4988476 | 156.324285 | 895.0224899 | 1495.361509 | 998.1020948 |
| MA2765 | 211.7013422 | 261.2821362 | 315.4062288 | 1706.889959 | 2851.791518 | 1903.472218 |
| MA2764 | 140.829657 | 173.8121886 | 209.8170496 | 838.518808 | 1400.957813 | 935.0908924 |
| MA2763 | 255.5655717 | 315.4194386 | 380.7579694 | 1576.97327 | 2634.73282 | 1758.593043 |
| MA2762 | 55.32862289 | 68.28667514 | 82.43212871 | 175.2839544 | 292.8561924 | 195.4713809 |
| MA2761 | 142.3066691 | 175.6351193 | 212.0175969 | 1204.230836 | 2011.972281 | 1342.921919 |
| MA2760 | 102.0330421 | 125.9293442 | 152.015366 | 575.2226805 | 961.0550184 | 641.4709897 |
| MA2757 | 244.7398512 | 302.058317 | 364.629117 | 991.5745446 | 1656.676144 | 1105.774035 |
| MA2756 | 308.7152003 | 381.0167955 | 459.9436927 | 1112.951843 | 1859.467629 | 1241.130339 |
| MA2755 | 40.07991258 | 49.46669241 | 59.71362271 | 189.044947 | 315.8474122 | 210.817225 |
| MA2754 | 19.9984051 | 24.68206366 | 29.79490574 | 140.9372505 | 235.471334 | 157.1689724 |
| MA2753 | 8.202784468 | 10.12388975 | 12.22103406 | 85.6116176 | 143.0358668 | 95.47149467 |
| MA2751 | 401.2497499 | 495.223085 | 597.8075957 | 2286.115011 | 3819.53351 | 2549.406531 |
| MA2750 | 33.51599898 | 41.36549971 | 49.93428351 | 127.7229935 | 213.3935744 | 142.4328314 |
| MA2749 | 37.45636235 | 46.2287025 | 55.80488942 | 217.0036666 | 362.5595267 | 241.9959461 |
| MA2748 | 1201.317035 | 1482.667412 | 1789.799118 | 8642.470394 | 14439.43333 | 9637.822403 |
| MA2747 | 46.62017735 | 57.53869768 | 69.45772837 | 309.9122008 | 517.7867389 | 345.6047422 |
| MA2746 | 314.096878 | 387.6588708 | 467.9616612 | 2094.088462 | 3498.704577 | 2335.264314 |
| MA2745 | 120.2392625 | 148.3994907 | 179.1401601 | 594.25147 | 992.8474255 | 662.691322 |
| MA2744 | 19.3611753 | 23.89559362 | 28.84551993 | 126.9993822 | 212.1845987 | 141.6258819 |
| MA2743 | 37.04032666 | 45.7152306 | 55.18505279 | 82.36963017 | 137.6193066 | 91.85612803 |
| MA2742 | 575.7371572 | 710.5757232 | 857.7701191 | 1703.726915 | 2846.506852 | 1899.944886 |
| MA2741 | 73.50545387 | 90.7205491 | 109.5131366 | 233.3113681 | 389.8056679 | 260.1818031 |
| MA2739 | 12.29160347 | 15.17031672 | 18.31281869 | 207.5935259 | 346.8375058 | 231.5020409 |
| MA2737b | 107.9500931 | 133.2321781 | 160.8309678 | 304.5654291 | 508.853604 | 339.6421836 |
| MA2737a | 9.919841115 | 12.24308382 | 14.77921511 | 71.99339821 | 120.2831859 | 80.284867 |
| MA2737 | 10.08873966 | 12.45153867 | 15.03085098 | 82.85110635 | 138.4237344 | 92.39305575 |
| MA2736 | 483.6365525 | 596.905009 | 720.5527349 | 1432.633144 | 2393.576121 | 1597.629287 |
| MA2733n | 82.53341401 | 101.862872 | 122.9635702 | 416.3681071 | 695.6482635 | 464.321159 |
| MA2732 | 11448.49337 | 14129.74888 | 17056.69922 | 10691.36827 | 17862.6356 | 11922.69154 |
| MA2731 | 994.0009778 | 1226.797602 | 1480.926368 | 2451.700112 | 4096.185314 | 2734.062043 |
| MA2730 | 250.9848514 | 309.7659063 | 373.9333187 | 766.3244306 | 1280.33884 | 854.5818995 |
| MA2729 | 53.94929252 | 66.58430338 | 80.37711392 | 172.2498019 | 287.786873 | 192.0877855 |
| MA2728 | 180.029893 | 222.1931828 | 268.2200737 | 4608.471436 | 7699.617472 | 5139.228395 |
| MA2727 | 72.18982067 | 89.09679249 | 107.5530219 | 330.0647097 | 551.4566035 | 368.0782124 |
| MA2726 | 67.03614066 | 82.73611235 | 99.87473908 | 253.4561833 | 423.4626783 | 282.6466936 |
| MA2724 | 330.2987165 | 407.6552058 | 492.1001985 | 975.7968703 | 1630.315547 | 1088.179248 |
| MA2723 | 125.0210161 | 154.3011386 | 186.264323 | 855.8202626 | 1429.86427 | 954.3849529 |
| MA2722 | 192.6611 | 237.7826388 | 287.0388556 | 1230.061042 | 2055.128174 | 1371.726986 |
| MA2721 | 6305.8716 | 7782.716845 | 9394.891688 | 13348.22436 | 22301.58588 | 14885.53735 |
| MA2720 | 28570.71066 | 35262.01693 | 42566.47599 | 74136.6779 | 123864.0769 | 82674.98789 |
| MA2719 | 520.9607158 | 642.9705513 | 776.1606658 | 1219.619091 | 2037.682254 | 1360.082438 |
| MA2718 | 27619.0848 | 34087.51877 | 41148.6828 | 130293.4488 | 217688.1704 | 145299.3256 |
| MA2717 | 3072.134368 | 3791.633166 | 4577.062692 | 22527.32707 | 37637.59925 | 25121.79592 |
| MA2716 | 2228.196417 | 2750.043593 | 3319.70984 | 25199.68743 | 42102.45334 | 28101.93163 |
| MA2715 | 10830.75032 | 13367.32942 | 16136.34603 | 44798.41149 | 74847.08034 | 49957.83777 |
| MA2714 | 12257.82285 | 15128.62462 | 18262.49015 | 66448.89096 | 111019.6838 | 74101.79969 |
| MA2713a | 225.628693 | 278.4712948 | 336.1560886 | 829.3931791 | 1385.711141 | 924.9142662 |
| MA2713 | 389.1297469 | 480.2645579 | 579.7504384 | 2203.755062 | 3681.930378 | 2457.561199 |
| MA2712 | 478.4605178 | 590.5167387 | 712.8411467 | 2191.944061 | 3662.197113 | 2444.389927 |
| MA2711 | 1585.613107 | 1956.96624 | 2362.348037 | 9539.04279 | 15937.38435 | 10637.65291 |
| MA2710 | 617.3682624 | 761.9568999 | 919.794808 | 4781.584362 | 7988.846411 | 5332.27871 |
| MA2709 | 45.44202813 | 56.08462403 | 67.70244615 | 160.9191976 | 268.8562319 | 179.4522373 |
| MA2708 | 190.6337886 | 235.2805278 | 284.0184371 | 510.0752115 | 852.2096892 | 568.8204965 |
| MA2706n | 1929.109951 | 2380.910597 | 2874.111653 | 3017.911515 | 5042.184714 | 3365.483928 |
| MA2705 | 397.2335854 | 490.2663284 | 591.8240564 | 999.0650607 | 1669.190946 | 1114.127233 |
| MA2704 | 66.31934958 | 81.85144766 | 98.80681779 | 336.2275656 | 561.7532136 | 374.9508436 |
| MA2703a | 164.1859176 | 202.6385229 | 244.6147036 | 731.4263659 | 1222.032794 | 815.6646299 |
| MA2703 | 346.8902688 | 428.1325263 | 516.8193567 | 1095.007484 | 1829.487037 | 1221.11933 |
| MA2702 | 270.0520169 | 333.2986325 | 402.3408039 | 508.617138 | 849.7736085 | 567.1944969 |
| MA2701 | 41.57872005 | 51.316523 | 61.94664215 | 475.3050286 | 794.1173019 | 530.0458369 |
| MA2700 | 25.85834033 | 31.91440512 | 38.52541284 | 144.073942 | 240.7119707 | 160.6669162 |
| MA2699 | 240.1271908 | 296.3653641 | 357.7568799 | 1138.177907 | 1901.614151 | 1269.261684 |
| MA2698 | 99.22595747 | 122.4648359 | 147.8331914 | 462.9949914 | 773.5502703 | 516.3180546 |
| MA2697 | 45.05792864 | 55.61056782 | 67.13019011 | 333.368486 | 556.9763976 | 371.7624841 |
| MA2696 | 174.8192579 | 215.7622086 | 260.4569357 | 767.0519008 | 1281.554263 | 855.3931523 |
| MA2695 | 525.0525363 | 648.0206827 | 782.2569222 | 1161.148303 | 1939.992009 | 1294.877578 |
| MA2694 | 31.25949752 | 38.58052199 | 46.57240301 | 94.30478621 | 157.5600044 | 105.1658542 |
| MA4675 | 272.9377377 | 336.8601938 | 406.6401358 | 381.0333141 | 636.6125522 | 424.9168633 |
| MA4661 | 786.7893013 | 971.056618 | 1172.209131 | 5960.796337 | 9959.018354 | 6647.30035 |
| MA2691a | 15.89343926 | 19.61570823 | 23.67906451 | 26.65989952 | 44.54210706 | 29.73031611 |
| MA2691 | 222.710768 | 274.8699871 | 331.8087769 | 306.4949558 | 512.0773666 | 341.7939336 |
| MA2690 | 680.1845053 | 839.4848076 | 1013.382473 | 7593.94078 | 12687.59933 | 8468.533792 |
| MA2689 | 2806.731122 | 3464.072054 | 4181.647925 | 2893.896669 | 4834.986537 | 3227.18631 |
| MA4681 | 252.3158748 | 311.4086574 | 375.9163626 | 228.7005894 | 382.1021956 | 255.0400017 |
| MA4680 | 24.85142154 | 30.67166433 | 37.02524068 | 65.54362093 | 109.5072012 | 73.09226985 |
| MA2688 | 94.65581758 | 116.8243619 | 141.0243041 | 348.9459622 | 583.0025128 | 389.134016 |
| MA4679 | 44.71515116 | 55.1875113 | 66.61949825 | 203.8436784 | 340.572437 | 227.3203241 |
| MA2687 | 430.3812541 | 531.1772343 | 641.2095779 | 1271.603002 | 2124.534529 | 1418.053327 |
| MA2686 | 114.1260219 | 140.8545193 | 170.0322624 | 425.0211155 | 710.1053033 | 473.9707332 |
| MA2685 | 93.06514068 | 114.8611459 | 138.6544117 | 309.2427348 | 516.6682265 | 344.8581738 |
| MA2684 | 13.96601169 | 17.23687403 | 20.80745937 | 49.47390521 | 82.65867549 | 55.1718074 |
| MA2683 | 1522.440042 | 1878.997943 | 2268.228757 | 5093.883595 | 8510.621291 | 5680.545398 |
| MA2682 | 19.74812904 | 24.37317254 | 29.42202841 | 139.7370127 | 233.466033 | 155.8305033 |
| MA2681a | 33.54401705 | 41.40007966 | 49.97602663 | 361.176197 | 603.4362142 | 402.772805 |
| MA2681 | 91.09413125 | 112.4285229 | 135.7178755 | 936.366086 | 1564.436446 | 1044.207227 |
| MA2680 | 268.9685943 | 331.9614706 | 400.7266515 | 2658.661329 | 4441.966388 | 2964.858952 |
| MA2679 | 75.20830999 | 92.82221685 | 112.0501608 | 847.2059433 | 1415.47187 | 944.7785238 |
| MA2678 | 7.137876405 | 8.809578511 | 10.63446578 | 57.57025813 | 96.1856814 | 64.20061605 |
| MA2677 | 994.5472651 | 1227.47183 | 1481.740262 | 1367.707739 | 2285.101806 | 1525.226432 |
| MA2676 | 213.2016572 | 263.1338273 | 317.6414941 | 1695.976622 | 2833.558027 | 1891.301993 |
| MA2673n | 452.9374333 | 559.0161068 | 674.8152194 | 1702.337183 | 2844.184953 | 1898.395099 |
| MA2672 | 1070.854715 | 1321.650607 | 1595.427992 | 4892.907885 | 8174.840521 | 5456.423347 |
| MA2671a | 48.09643035 | 59.36069151 | 71.65714472 | 108.6339602 | 181.5005147 | 121.1453169 |
| MA2671 | 363.9884388 | 449.2351151 | 542.2933062 | 875.5664748 | 1462.855313 | 976.4053334 |
| MA2669 | 228.999475 | 282.6315195 | 341.1780958 | 1538.843199 | 2571.026889 | 1716.071538 |
| MA2668a | 60.69456921 | 74.9093347 | 90.42665948 | 99.93265516 | 166.9627833 | 111.4418839 |
| MA2668 | 7.304777222 | 9.015567766 | 10.88312531 | 39.80419713 | 66.50298172 | 44.3884405 |
| MA2667 | 636.8124482 | 785.9549452 | 948.7640022 | 2205.283907 | 3684.4847 | 2459.266121 |
| MA2666 | 82.90781758 | 102.3249615 | 123.5213807 | 292.6521364 | 488.9494345 | 326.3568387 |
| MA2665 | 291.3277062 | 359.5571224 | 434.0386896 | 1114.563986 | 1862.161123 | 1242.928152 |
| MA2664 | 419.7944809 | 518.1110218 | 625.4367246 | 854.1479031 | 1427.07017 | 952.519988 |
| MA2663 | 1618.582192 | 1997.656738 | 2411.467493 | 2113.056397 | 3530.395312 | 2356.416783 |
| MA2661 | 34.99362149 | 43.18918379 | 52.13574024 | 109.2073974 | 182.4585866 | 121.7847968 |
| MA2660 | 30.22273635 | 37.30094969 | 45.02776976 | 96.88572047 | 161.872108 | 108.0440343 |
| MA2659 | 148.6847848 | 183.5069999 | 221.5201224 | 736.4795311 | 1230.475385 | 821.2997674 |
| MA2658a | 152.044616 | 187.653709 | 226.5258143 | 569.9131565 | 952.1841153 | 635.5499687 |
| MA2658 | 6.203282695 | 7.656101454 | 9.242048162 | 29.90222806 | 49.9592372 | 33.34606315 |
| MA2657 | 181.5299088 | 224.0445047 | 270.4548935 | 270.6961691 | 452.2664363 | 301.872206 |
| MA2656 | 430.1244437 | 530.8602785 | 640.8269654 | 1977.530079 | 3303.96431 | 2205.28192 |
| MA2655 | 311.2883205 | 384.1925445 | 463.7772921 | 863.2012603 | 1442.196094 | 962.616019 |
| MA2654 | 76.4070442 | 94.30169654 | 113.8361118 | 305.5272107 | 510.4605035 | 340.7147334 |
| MA2653 | 1055.387577 | 1302.561041 | 1572.384058 | 4457.471626 | 7447.334087 | 4970.83796 |
| MA2652 | 128.8047932 | 158.9710824 | 191.9016367 | 677.6218517 | 1132.138741 | 755.663458 |
| MA2651 | 59.83505906 | 73.84852589 | 89.14610615 | 180.7259831 | 301.9484782 | 201.5401673 |
| MA2650 | 58.17756301 | 71.8028416 | 86.6766623 | 181.6614706 | 303.5114468 | 202.5833947 |
| MA2649 | 39.95787154 | 49.31606916 | 59.53179814 | 149.0530807 | 249.0308815 | 166.2195014 |
| MA2648 | 211.8964393 | 261.5229253 | 315.6968969 | 1072.31129 | 1791.567304 | 1195.80922 |
| MA2647 | 33.30622868 | 41.10660087 | 49.62175427 | 88.4040787 | 147.7013796 | 98.58556309 |
| MA2646 | 30.04533053 | 37.08199514 | 44.7634592 | 101.1868837 | 169.0582894 | 112.8405618 |
| MA2645 | 359.2666207 | 443.4074397 | 535.258439 | 605.443703 | 1011.546882 | 675.1725628 |
| MA2644 | 229.5996143 | 283.3722123 | 342.0722217 | 181.8958624 | 303.9030575 | 202.8447813 |
| MA2641n | 33.61292882 | 41.48513068 | 50.0786958 | 97.29872349 | 162.562134 | 108.5046028 |
| MA2640 | 288.8436681 | 356.4913185 | 430.3378103 | 552.1883303 | 922.5703086 | 615.7837768 |
| MA2639 | 123.1528268 | 151.9954165 | 183.480975 | 350.7321961 | 585.9868683 | 391.1259701 |
| MA2638 | 47.23477947 | 58.29724061 | 70.373402 | 76.3477228 | 127.5581868 | 85.1406785 |
| MA2637 | 57.24848753 | 70.65617516 | 85.29246611 | 100.7140342 | 168.2682746 | 112.3132542 |
| MA2636 | 386.469317 | 476.9810511 | 575.7867595 | 1134.375861 | 1895.261872 | 1265.021758 |
| MA2635 | 80.57439029 | 99.44504177 | 120.0448911 | 272.0801141 | 454.5786665 | 303.4155397 |
| MA2633 | 54.11225858 | 66.78543635 | 80.61991119 | 185.6412302 | 310.1606421 | 207.0215026 |
| MA2632 | 30.23310433 | 37.31374587 | 45.04321665 | 77.6262202 | 129.6942402 | 86.56642024 |
| MA2631 | 287.3587183 | 354.6585911 | 428.125437 | 519.5113327 | 867.975117 | 579.3433744 |
| MA2630 | 379.9454643 | 468.9293016 | 566.0671055 | 1231.050084 | 2056.780619 | 1372.829936 |
| MA2629 | 321.3704037 | 396.6358678 | 478.7982259 | 1224.418966 | 2045.701659 | 1365.435113 |
| MA2628 | 493.59296 | 609.1932231 | 735.386429 | 675.1800088 | 1128.059024 | 752.9403883 |
| MA2627 | 6546.349145 | 8079.514618 | 9753.17055 | 8379.179031 | 13999.53849 | 9344.207813 |
| MA2626a | 20.93030165 | 25.83221188 | 31.18330494 | 116.5723154 | 194.7635456 | 129.9979314 |
| MA2624n | 364.106056 | 449.3802785 | 542.4685399 | 1310.626279 | 2189.732785 | 1461.570909 |
| MA2623 | 96.80962845 | 119.4825987 | 144.2331895 | 390.1597222 | 651.8605259 | 435.0943584 |
| MA2622 | 888.3466815 | 1096.3989 | 1323.515825 | 3855.032278 | 6440.806739 | 4299.015763 |
| MA2620n | 53.14071617 | 65.58635716 | 79.17244505 | 227.0056274 | 379.2703327 | 253.1498312 |
| MA2619 | 42.82530476 | 52.85505984 | 63.80388394 | 194.874376 | 325.5869481 | 217.3180285 |
| MA2618 | 60.41415169 | 74.56324295 | 90.00887548 | 410.4359848 | 685.7371523 | 457.7058351 |
| MA2617 | 26.32841608 | 32.49457337 | 39.22576181 | 202.0047624 | 337.5000625 | 225.2696204 |
| MA2616 | 9.972122402 | 12.30760947 | 14.85710713 | 134.6143539 | 224.9073354 | 150.1178687 |
| MA2615 | 483.0240648 | 596.1490757 | 719.6402115 | 6364.638661 | 10633.73913 | 7097.653134 |
| MA2614 | 105.9916728 | 130.8150926 | 157.9131878 | 510.1663747 | 852.3620004 | 568.922159 |
| MA2612n | 51.64981317 | 63.74628228 | 76.95120219 | 199.3589104 | 333.0794973 | 222.3190459 |
| MA2611 | 25.649484 | 31.6566343 | 38.21424528 | 84.23148621 | 140.7300082 | 93.93241375 |
| MA2610a | 19.8529859 | 24.50258705 | 29.57825088 | 213.4897097 | 356.6885727 | 238.0772873 |
| MA2610 | 270.431213 | 333.766637 | 402.9057547 | 2608.59368 | 4358.315713 | 2909.025019 |
| MA2609 | 707.3387076 | 872.9985677 | 1053.838544 | 5445.269408 | 9097.700193 | 6072.400263 |
| MA2608 | 62.50328365 | 77.14165296 | 93.12139818 | 16.5251786 | 27.60949171 | 18.42838093 |
| MA2607 | 28.03693973 | 34.603236 | 41.77122986 | 18.95104449 | 31.66251441 | 21.13363342 |
| MA2606 | 29.53562096 | 36.45291078 | 44.00406121 | 67.09452364 | 112.0983766 | 74.82178978 |
| MA2604 | 34.64494971 | 42.75885251 | 51.61626667 | 122.4616132 | 204.6031075 | 136.5654987 |
| MA2603 | 310.7138739 | 383.4835617 | 462.9214448 | 1336.274578 | 2232.584758 | 1490.173119 |
| MA2602 | 1918.503447 | 2367.820032 | 2858.309403 | 15253.33857 | 25484.56116 | 17010.06327 |
| MA2601 | 281.1234985 | 346.9630728 | 418.8358068 | 1311.494617 | 2191.183564 | 1462.539254 |
| MA2600 | 116.5032237 | 143.7884656 | 173.57397 | 531.1194708 | 887.3694486 | 592.28842 |
| MA2599 | 1386.93934 | 1711.762759 | 2066.351126 | 5251.349528 | 8773.707972 | 5856.14666 |
| MA2598 | 134.1653529 | 165.5870938 | 199.8881422 | 637.2090345 | 1064.618905 | 710.5963027 |
| MA2597 | 55.30312656 | 68.25520753 | 82.39414265 | 258.3460993 | 431.6325201 | 288.0997805 |
| MA2596 | 89.47188682 | 110.4263463 | 133.3009517 | 237.2593946 | 396.401845 | 264.5845232 |
| MA2595 | 291.3525678 | 359.5878066 | 434.0757299 | 429.0287289 | 716.8010354 | 478.4399028 |
| MA2594 | 172.8362448 | 213.3147706 | 257.5025156 | 145.5115894 | 243.1139244 | 162.2701372 |
| MA2593 | 71.67805826 | 88.46517451 | 106.7905655 | 100.5498661 | 167.9939903 | 112.130179 |
| MA2592 | 29.09719502 | 35.91180478 | 43.35086615 | 35.75071076 | 59.73060721 | 39.86811471 |
| MA2591 | 69.25473318 | 85.4743028 | 103.1801404 | 125.2580409 | 209.2752475 | 139.6839906 |
| MA2589 | 92.69723907 | 114.4070812 | 138.1062883 | 178.9878322 | 299.0444573 | 199.6018337 |
| MA2586 | 106.1755658 | 131.0420536 | 158.1871633 | 613.7086413 | 1025.355553 | 684.3893728 |
| MA2585 | 23.07399403 | 28.47796045 | 34.37711524 | 110.9391042 | 185.351841 | 123.7159441 |
| MA2584 | 567.2225158 | 700.066939 | 845.0844606 | 2279.868013 | 3809.09632 | 2542.440068 |
| MA2583 | 24.8654773 | 30.68901198 | 37.04618186 | 116.1049263 | 193.9826537 | 129.4767131 |
| MA2582 | 361.8860447 | 446.640337 | 539.1610248 | 1159.444842 | 1937.145947 | 1292.97793 |
| MA2580 | 331.0023406 | 408.5236199 | 493.1485029 | 1434.525755 | 2396.738207 | 1599.73987 |
| MA2579 | 34.99110506 | 43.186078 | 52.1319911 | 27.64495586 | 46.18789291 | 30.82882125 |
| MA2578 | 8.275240631 | 10.21331526 | 12.3289839 | 67.46241147 | 112.7130263 | 75.2320472 |
| MA2577 | 77.88056414 | 96.12031721 | 116.0314563 | 363.6047809 | 607.4937781 | 405.4810886 |
| MA2576 | 682.5644447 | 842.4221327 | 1016.928259 | 1306.98957 | 2183.656744 | 1457.515362 |
| MA2575 | 3046.80347 | 3760.369731 | 4539.323097 | 5474.943764 | 9147.278711 | 6105.492209 |
| MA2574 | 1945.515111 | 2401.157872 | 2898.553112 | 4224.714023 | 7058.453624 | 4711.273699 |
| MA2573 | 1443.017166 | 1780.974101 | 2149.899464 | 7663.738278 | 12804.21371 | 8546.369857 |
| MA2572 | 704.1078105 | 869.0109893 | 1049.024946 | 4151.445042 | 6936.039253 | 4629.56634 |
| MA2571 | 981.9537088 | 1211.928843 | 1462.977574 | 5938.378111 | 9921.563036 | 6622.30022 |
| MA2570 | 172.7333953 | 213.1878335 | 257.3492837 | 1078.598309 | 1802.071359 | 1202.820313 |
| MA2569 | 278.9415807 | 344.2701465 | 415.585046 | 979.3125158 | 1636.189323 | 1092.09979 |
| MA2568 | 1859.168663 | 2294.588946 | 2769.908638 | 6793.131279 | 11349.64446 | 7575.495182 |
| MA2567 | 254.5490096 | 314.1647961 | 379.2434301 | 1723.276771 | 2879.169833 | 1921.746296 |
| MA2564n | 530.8569913 | 655.1845504 | 790.9047713 | 6219.067827 | 10390.52622 | 6935.316928 |
| MA2563 | 2549.197175 | 3146.223243 | 3797.957344 | 3138.688124 | 5243.972595 | 3500.170361 |
| MA2561 | 436.6194093 | 538.8763754 | 650.5035814 | 1512.388214 | 2526.827143 | 1686.569736 |
| MA2560 | 3578.536989 | 4416.636093 | 5331.533774 | 2932.092286 | 4898.80198 | 3269.780912 |
| MA2559 | 293.5696836 | 362.3241745 | 437.3789312 | 1657.393808 | 2769.095674 | 1848.275602 |
| MA2558 | 5922.046849 | 7308.999722 | 8823.044973 | 4530.128641 | 7568.726011 | 5051.862872 |
| MA2557 | 1773.69766 | 2189.100498 | 2642.568461 | 1278.221625 | 2135.592612 | 1425.434216 |
| MA2556 | 493.988211 | 609.6810426 | 735.9752993 | 688.9392067 | 1151.047245 | 768.284231 |
| MA2555 | 3368.068462 | 4156.875499 | 5017.964273 | 12720.70113 | 21253.14956 | 14185.74235 |
| MA2554 | 326.1970389 | 402.5929088 | 485.9892564 | 1226.28296 | 2048.815934 | 1367.513784 |
| MA2553 | 395.2731084 | 487.8467046 | 588.9032122 | 1116.760594 | 1865.831112 | 1245.377743 |
| MA2552 | 247.5281042 | 305.4995833 | 368.7832351 | 732.6918792 | 1224.147154 | 817.0758922 |
| MA2551 | 44.52189801 | 54.94899795 | 66.33157731 | 347.9527975 | 581.3431798 | 388.0264686 |
| MA2550 | 221.3023015 | 273.1316556 | 329.7103534 | 1509.173874 | 2521.456775 | 1682.985201 |
| MA2549 | 226.9707766 | 280.1276967 | 338.1556109 | 1405.123061 | 2347.613568 | 1566.950872 |
| MA2548 | 45.93103635 | 56.68815875 | 68.43100193 | 372.5988911 | 622.5207146 | 415.511049 |
| MA2547 | 10.598703 | 13.08093625 | 15.79062706 | 144.9101334 | 242.10904 | 161.5994116 |
| MA2546 | 2927.200232 | 3612.755223 | 4361.130526 | 6591.322772 | 11012.47229 | 7350.444419 |
| MA2545 | 7251.377901 | 8949.66224 | 10803.56758 | 19554.56426 | 32670.84687 | 21806.66047 |
| MA2544 | 119.2268438 | 147.1499619 | 177.6317938 | 541.449123 | 904.6277459 | 603.8077367 |
| MA2543 | 113.4028702 | 139.9620044 | 168.9548647 | 351.4838176 | 587.2426423 | 391.9641558 |
| MA2542 | 63.13471684 | 77.92096882 | 94.06214782 | 541.5471053 | 904.7914501 | 603.9170035 |
| MA2541 | 1645.574552 | 2030.970752 | 2451.682441 | 5030.677327 | 8405.019229 | 5610.059674 |
| MA2540 | 505.4488248 | 623.8257505 | 753.0500563 | 1675.057872 | 2798.607962 | 1867.974034 |
| MA2537 | 46.92535358 | 57.91534668 | 69.91239948 | 352.7058253 | 589.2843153 | 393.3269019 |
| MA2536 | 232.5334743 | 286.993187 | 346.4432744 | 907.4498912 | 1516.124627 | 1011.960758 |
| MA2535 | 87.49947948 | 107.991998 | 130.3623329 | 765.0935512 | 1278.282345 | 853.2092599 |
| MA2534 | 1.362067664 | 1.681066097 | 2.029295709 | 67.84408099 | 113.3507018 | 75.65767355 |
| MA2533 | 461.754517 | 569.8981657 | 687.9514759 | 3720.896097 | 6216.698311 | 4149.431138 |
| MA2532 | 1399.493608 | 1727.257257 | 2085.055279 | 1067.045028 | 1782.76868 | 1189.936443 |
| MA2531 | 199.0096974 | 245.6180878 | 296.4974028 | 339.1002358 | 566.5527359 | 378.1543588 |
| MA2530 | 43.5632151 | 53.76578997 | 64.90327008 | 134.434732 | 224.6072316 | 149.9175598 |
| MA2529 | 192.893971 | 238.0700485 | 287.3858017 | 590.415706 | 986.4388113 | 658.4137936 |
| MA2528 | 253.2807081 | 312.5994562 | 377.3538331 | 1143.637413 | 1910.735638 | 1275.349961 |
| MA2527 | 295.9230183 | 365.2286639 | 440.88508 | 326.9742356 | 546.2931846 | 364.6318089 |
| MA2526 | 76.70343036 | 94.66749682 | 114.2776869 | 388.3354937 | 648.8126906 | 433.0600338 |
| MA2525 | 177.8005526 | 219.4417273 | 264.8986595 | 816.2459455 | 1363.745361 | 910.2528677 |
| MA2524 | 36.14822931 | 44.61420261 | 53.85594898 | 83.45594772 | 139.4342749 | 93.0675566 |
| MA2523 | 133.3058713 | 164.5263202 | 198.6076314 | 433.5054306 | 724.2804981 | 483.4321855 |
| MA2522 | 74.77318765 | 92.28518816 | 111.4018876 | 330.1706836 | 551.6336597 | 368.1963913 |
| MA2521 | 156.45316 | 193.0947411 | 233.0939458 | 847.5337249 | 1416.019512 | 945.1440559 |
| MA2520 | 335.2608574 | 413.7794881 | 499.4931142 | 1104.219183 | 1844.87751 | 1231.391939 |
| MA2519 | 1215.345246 | 1499.981052 | 1810.699245 | 2594.64123 | 4335.004616 | 2893.465668 |
| MA2518 | 1105.497754 | 1364.407102 | 1647.041412 | 4945.303318 | 8262.380347 | 5514.853153 |
| MA2517 | 1346.287345 | 1661.590001 | 2005.785178 | 2863.704429 | 4784.542763 | 3193.516835 |
| MA2516 | 65.32591999 | 80.62535526 | 97.32674272 | 167.9543524 | 280.6102379 | 187.2976298 |
| MA2515 | 54.28874004 | 67.00325006 | 80.88284459 | 181.3967019 | 303.0690836 | 202.2881326 |
| MA2514 | 762.9888327 | 941.6820414 | 1136.749667 | 1736.249186 | 2900.843535 | 1936.212743 |
| MA2513 | 470.1071659 | 580.2070183 | 700.3957876 | 2196.106649 | 3669.151769 | 2449.031919 |
| MA2512 | 175.1120015 | 216.1235132 | 260.8930838 | 1678.255152 | 2803.949828 | 1871.539545 |
| MA2511 | 17.93390331 | 22.13405223 | 26.71907863 | 44.87685936 | 74.97814735 | 50.04532047 |
| MA2510 | 6166.286516 | 7610.440711 | 9186.928884 | 30515.63459 | 50984.088 | 34030.11562 |
| MA2509 | 637.4481478 | 786.7395267 | 949.7111082 | 2084.676418 | 3482.979377 | 2324.768286 |
| MA2508 | 431.3703162 | 532.3979363 | 642.683146 | 1175.00076 | 1963.136043 | 1310.32542 |
| MA2507 | 374.0154381 | 461.6104537 | 557.2321724 | 841.1850447 | 1405.412436 | 938.0641991 |
| MA2506 | 81.59338799 | 100.7026904 | 121.5630592 | 176.6182667 | 295.0854988 | 196.9593658 |
| MA2505 | 95.7261209 | 118.145332 | 142.6189106 | 204.3343924 | 341.3922988 | 227.8675535 |
| MA2504 | 45.13224192 | 55.70228538 | 67.24090679 | 209.345622 | 349.7648257 | 233.4559256 |
| MA2503 | 95.91559019 | 118.3791753 | 142.901194 | 440.3096909 | 735.6487364 | 491.0200914 |
| MA2502 | 69.63876286 | 85.94827285 | 103.7522925 | 284.364368 | 475.1026205 | 317.1145693 |
| MA2501 | 240.2468533 | 296.5130518 | 357.9351609 | 794.530236 | 1327.463774 | 886.0361632 |
| MA2500 | 37.38011997 | 46.13460404 | 55.69129863 | 164.7986643 | 275.3378626 | 183.7785016 |
| MA2499 | 121.6606916 | 150.1538207 | 181.2578962 | 288.7564967 | 482.4407828 | 322.0125388 |
| MA2498 | 163.5045682 | 201.7976004 | 243.5995856 | 1022.970375 | 1709.130822 | 1140.785719 |
| MA2497 | 104.1702278 | 128.5670623 | 155.1994822 | 537.9506488 | 898.7826597 | 599.9063437 |
| MA2496 | 276.8405281 | 341.6770239 | 412.454763 | 793.8081214 | 1326.257299 | 885.2308828 |
| MA2495 | 1269.359221 | 1566.645187 | 1891.172727 | 3993.702392 | 6672.490247 | 4453.656493 |
| MA2493 | 151.9038525 | 187.4799785 | 226.3160959 | 1012.504076 | 1691.644221 | 1129.114017 |
| MA2491 | 6.892880618 | 8.507204318 | 10.26945535 | 104.1118466 | 173.94518 | 116.1023923 |
| MA2490 | 15.01745706 | 18.5345696 | 22.37397009 | 134.2383075 | 224.2790549 | 149.6985131 |
| MA2489 | 78.79774203 | 97.25229964 | 117.3979267 | 258.1747072 | 431.3461663 | 287.9086492 |
| MA2488 | 64.20361156 | 79.2402004 | 95.65465568 | 365.0218179 | 609.8612968 | 407.0613255 |
| MA2487 | 10.61859645 | 13.10548877 | 15.8202656 | 59.41625525 | 99.26988663 | 66.25921638 |
| MA2486 | 484.7286462 | 598.2528728 | 722.1798061 | 1934.825643 | 3232.615745 | 2157.65922 |
| MA2485 | 254.6422736 | 314.2799026 | 379.3823807 | 852.7039411 | 1424.657667 | 950.9097251 |
| MA2484 | 163.371555 | 201.6334353 | 243.401414 | 603.8234299 | 1008.839806 | 673.365683 |
| MA2483 | 26.74465974 | 33.00830195 | 39.84590829 | 107.1204487 | 178.9718109 | 119.4574946 |
| MA2482 | 511.4258637 | 631.2026215 | 761.9550319 | 1526.987118 | 2551.218306 | 1702.849993 |
| MA2481 | 178.1533834 | 219.8771916 | 265.4243294 | 416.847422 | 696.4490802 | 464.8556765 |
| MA2480 | 129.549541 | 159.8902513 | 193.0112097 | 405.6907069 | 677.8089651 | 452.4140443 |
| MA2479 | 206.7103034 | 255.1221881 | 307.9702593 | 776.6439319 | 1297.580178 | 866.0898961 |
| MA2478 | 83.76754362 | 103.3860367 | 124.8022556 | 187.7062792 | 313.6108288 | 209.324383 |
| MA2477 | 139.5010866 | 172.1724649 | 207.8376603 | 588.3495798 | 982.9868248 | 656.109712 |
| MA2476 | 345.3672681 | 426.2528364 | 514.5502926 | 1449.917296 | 2422.453671 | 1616.904052 |
| MA2475 | 17.33387655 | 21.39349825 | 25.82512031 | 28.76892093 | 48.06576092 | 32.08223321 |
| MA2474 | 17.8229927 | 21.99716618 | 26.5538369 | 71.60241071 | 119.6299424 | 79.84884953 |
| MA2473 | 391.9813593 | 483.7840225 | 583.9989534 | 2348.540265 | 3923.83069 | 2619.021292 |
| MA2472 | 210.6709178 | 260.0103847 | 313.8710364 | 1515.224261 | 2531.565478 | 1689.73241 |
| MA2471 | 22.455675 | 27.71483011 | 33.45590393 | 150.1724806 | 250.9011223 | 167.4678225 |
| MA2470 | 20.85861431 | 25.74373525 | 31.07650055 | 170.3703904 | 284.6468406 | 189.9919225 |
| MA2469 | 39.94544666 | 49.30073435 | 59.51328676 | 42.66086277 | 71.27576441 | 47.57410789 |
| MA2468 | 40.00041689 | 49.3685787 | 59.59518494 | 94.02960522 | 157.1002449 | 104.8589806 |
| MA2467 | 73.37343579 | 90.55761217 | 109.3164476 | 216.3378007 | 361.4470292 | 241.2533925 |
| MA2466a | 113.225088 | 139.7425852 | 168.6899933 | 1120.214199 | 1871.601232 | 1249.229099 |
| MA2466 | 476.4359721 | 588.0180411 | 709.8248487 | 575.6701445 | 961.8026202 | 641.969988 |
| MA2465 | 39.17803651 | 48.35359552 | 58.36994994 | 160.9015487 | 268.8267449 | 179.4325557 |
| MA2464 | 189.5166636 | 233.9017703 | 282.3540727 | 1111.006049 | 1856.216688 | 1238.960448 |
| MA2463 | 202.3335241 | 249.7203601 | 301.4494529 | 1330.220235 | 2222.469447 | 1483.421499 |
| MA2462 | 123.7952664 | 152.7883165 | 184.4381227 | 712.9191895 | 1191.111874 | 795.0259848 |
| MA2461 | 231.4043252 | 285.5995894 | 344.7609956 | 1023.122454 | 1709.384908 | 1140.955313 |
| MA2460 | 11.01070361 | 13.58942806 | 16.40445197 | 55.35997319 | 92.49284122 | 61.73577293 |
| MA2459 | 20.5698539 | 25.38734669 | 30.64628678 | 208.9475037 | 349.0996684 | 233.0119561 |
| MA2458 | 407.8149242 | 503.3258337 | 607.5888132 | 760.1196393 | 1269.972167 | 847.6625033 |
| MA2457 | 161.8287079 | 199.7292509 | 241.1027815 | 540.9897599 | 903.8602637 | 603.2954688 |
| MA2456 | 1026.909937 | 1267.413892 | 1529.956244 | 2036.627264 | 3402.701108 | 2271.185318 |
| MA2455 | 675.3138274 | 833.4734092 | 1006.125825 | 2015.353493 | 3367.157892 | 2247.46145 |
| MA2454 | 492.0416586 | 607.2786044 | 733.0752007 | 875.363213 | 1462.515713 | 976.1786619 |
| MA2453 | 292.0241244 | 360.4166428 | 435.076258 | 518.9812774 | 867.0895255 | 578.7522727 |
| MA2452 | 190.5421514 | 235.167429 | 283.8819101 | 884.7885024 | 1478.263044 | 986.6894605 |
| MA2451 | 131.5740067 | 162.3888502 | 196.0273886 | 906.8737429 | 1515.162025 | 1011.318255 |
| MA2450 | 67.3009241 | 83.0629085 | 100.2692304 | 191.2984334 | 319.6124313 | 213.3302451 |
| MA2449 | 2869.400727 | 3541.418981 | 4275.01712 | 7633.705224 | 12754.03589 | 8512.877901 |
| MA2448 | 1296.022015 | 1599.55245 | 1930.896667 | 1989.723386 | 3324.336314 | 2218.879528 |
| MA2447 | 272.062914 | 335.7804849 | 405.3367673 | 797.8854395 | 1333.069491 | 889.777785 |
| MA2445 | 320.0745831 | 395.036564 | 476.8676293 | 1561.491455 | 2608.866532 | 1741.32819 |
| MA2444 | 545.2448041 | 672.942012 | 812.340657 | 1943.358072 | 3246.871326 | 2167.174327 |
| MA2443 | 51.76539654 | 63.88893546 | 77.1234057 | 269.8392204 | 450.8346867 | 300.9165627 |
| MA2442 | 122.3458427 | 150.9994354 | 182.2786783 | 198.2902947 | 331.2941045 | 221.127358 |
| MA2441 | 1986.674846 | 2451.957283 | 2959.875524 | 1141.091785 | 1906.482522 | 1272.511154 |
| MA2440 | 828.0847714 | 1022.023553 | 1233.733769 | 658.8613128 | 1100.794514 | 734.7422706 |
| MA2439 | 306.0561095 | 377.7349413 | 455.9820087 | 128.87876 | 215.3245747 | 143.7217073 |
| MA2438 | 216.2976769 | 266.9549399 | 322.2541429 | 130.5943887 | 218.1909666 | 145.6349247 |
| MA2437 | 232.3912181 | 286.8176143 | 346.2313321 | 145.1767459 | 242.5544836 | 161.8967298 |
| MA2436 | 82.29649715 | 101.5704688 | 122.6105963 | 79.91696621 | 133.5215109 | 89.12099113 |
| MA2435 | 430.2599685 | 531.0275434 | 641.0288788 | 433.7796037 | 724.7385737 | 483.7379351 |
| MA2434 | 283.5269119 | 349.9293696 | 422.4165662 | 419.6472725 | 701.1269389 | 467.9779854 |
| MA2433 | 113.5582155 | 140.1537318 | 169.186308 | 196.9468597 | 329.0495565 | 219.6291998 |
| MA2432 | 2646.342225 | 3266.119819 | 3942.690266 | 2746.601408 | 4588.892539 | 3062.927077 |
| MA2431 | 3280.873742 | 4049.259636 | 4888.055991 | 24794.82595 | 41426.02981 | 27650.44231 |
| MA2430 | 156.530189 | 193.1898104 | 233.2087085 | 1028.466246 | 1718.313065 | 1146.914549 |
| MA2429 | 478.3559479 | 590.3876783 | 712.6853517 | 1524.017073 | 2546.256094 | 1699.537888 |
| MA2428 | 43.53821158 | 53.73493058 | 64.86601822 | 48.21734371 | 80.55927159 | 53.7705279 |
| MA2427 | 3902.008112 | 4815.864672 | 5813.461787 | 4005.103846 | 6691.539262 | 4466.371051 |
| MA2426 | 37.26366611 | 45.9908765 | 55.51779821 | 132.0105359 | 220.5569988 | 147.2141694 |
| MA2425 | 22.84723458 | 28.19809357 | 34.03927448 | 75.00896689 | 125.3214564 | 83.64773827 |
| MA2423 | 424.360927 | 523.7469369 | 632.2401087 | 3303.460577 | 5519.266665 | 3683.919632 |
| MA2422 | 118.3962667 | 146.1248623 | 176.3943468 | 128.4215795 | 214.5607391 | 143.2118734 |
| MA2421 | 152.0909355 | 187.7108767 | 226.5948242 | 316.5384373 | 528.8575434 | 352.9941213 |
| MA2420 | 40.65511437 | 50.17660738 | 60.57059521 | 94.65384594 | 158.1431969 | 105.5551151 |
| MA2419 | 6.468165313 | 7.983020006 | 9.636687265 | 77.95526562 | 130.2439938 | 86.93336178 |
| MA2418 | 200.674946 | 247.6733402 | 298.9783969 | 1052.910223 | 1759.152913 | 1174.173734 |
| MA2417 | 170.8496002 | 210.8628506 | 254.5426851 | 829.447725 | 1385.802274 | 924.9750942 |
| MA2416 | 51.92950622 | 64.09147989 | 77.36790683 | 401.6455319 | 671.0504768 | 447.9029871 |
| MA2415 | 57.63592879 | 71.13435578 | 85.86970092 | 227.5249694 | 380.138025 | 253.7289858 |
| MA2414 | 272.4857733 | 336.3023786 | 405.9667702 | 1600.258756 | 2673.637116 | 1784.560316 |
| MA2413 | 456.5836894 | 563.516322 | 680.2476455 | 1680.888896 | 2808.350163 | 1874.476616 |
| MA2412 | 658.2557578 | 812.4203123 | 980.7116193 | 579.5420142 | 968.2715581 | 646.2877803 |
| MA2411 | 66.97863295 | 82.66513624 | 99.78906041 | 55.78725425 | 93.20672233 | 62.21226389 |
| MA2410 | 1656.098259 | 2043.959128 | 2467.361335 | 805.9628791 | 1346.564897 | 898.7855021 |
| MA2409 | 312.8553985 | 386.1266347 | 466.1120253 | 340.2623719 | 568.4943781 | 379.4503378 |
| MA2408 | 56.42056756 | 69.634355 | 84.05897786 | 78.30549938 | 130.8291479 | 87.32393191 |
| MA2407a | 636.1189786 | 785.0990639 | 947.7308268 | 529.8727785 | 885.2865337 | 590.8981464 |
| MA2407 | 1160.869274 | 1432.746721 | 1729.537451 | 253.5222879 | 423.5731228 | 282.7204115 |
| MA2406 | 1184.97142 | 1462.493628 | 1765.446373 | 3507.716513 | 5860.527884 | 3911.699694 |
| MA2405 | 18.62082274 | 22.9818493 | 27.74249524 | 167.2658492 | 279.4599191 | 186.5298317 |
| MA2404 | 405.1667276 | 500.0574252 | 603.6433602 | 1849.276114 | 3089.683613 | 2062.256965 |
| MA2403 | 701.6602169 | 865.9901654 | 1045.378365 | 3135.97852 | 5239.445516 | 3497.148692 |
| MA2402 | 126.039587 | 155.5582605 | 187.7818554 | 153.4761444 | 256.4207285 | 171.1519688 |
| MA2401 | 333.1252874 | 411.1437641 | 496.311405 | 345.5432067 | 577.3173489 | 385.3393656 |
| MA2400 | 674.7990894 | 832.8381188 | 1005.358935 | 3383.359502 | 5652.758034 | 3773.020505 |
| MA2399 | 604.2187421 | 745.7277409 | 900.2038098 | 786.8427045 | 1314.619808 | 877.4632598 |
| MA2398 | 434.087509 | 535.7515001 | 646.7313941 | 410.6785945 | 686.1424932 | 457.9763861 |
| MA2397 | 1338.763325 | 1652.303844 | 1994.575411 | 3022.464766 | 5049.792072 | 3370.561576 |
| MA2396 | 43.23539434 | 53.36119306 | 64.41486169 | 200.8800814 | 335.6209984 | 224.01541 |
| MA2395 | 21.18402188 | 26.14535379 | 31.56131361 | 113.8495486 | 190.2144748 | 126.9615839 |
| MA2394 | 12.63460689 | 15.59365209 | 18.82384716 | 85.63245137 | 143.0706749 | 95.49472785 |
| MA2392 | 106.5453525 | 131.498445 | 158.7380952 | 494.9236094 | 826.8951046 | 551.9238867 |
| MA2391 | 1261.905861 | 1557.446238 | 1880.068234 | 4447.455179 | 7430.599077 | 4959.66792 |
| MA2390 | 284.6410712 | 351.3044668 | 424.0765122 | 2435.415531 | 4068.977802 | 2715.90197 |
| MA2389 | 4933.158281 | 6088.511865 | 7349.735401 | 8237.033321 | 13762.04812 | 9185.691204 |
| MA2388 | 2516.559506 | 3105.941779 | 3749.331652 | 4404.788131 | 7359.312978 | 4912.086914 |
| MA2387 | 414.9377676 | 512.1168584 | 618.2008818 | 1014.030668 | 1694.19478 | 1130.816427 |
| MA2386 | 87.42450766 | 107.8994676 | 130.2506351 | 249.6998989 | 417.1868549 | 278.4577985 |
| MA2385 | 17.30792392 | 21.36146747 | 25.78645442 | 51.99372198 | 86.86866692 | 57.98183109 |
| MA2384 | 242.4207617 | 299.196093 | 361.1739888 | 764.7332427 | 1277.680359 | 852.8074547 |
| MA2383 | 179.4928551 | 221.5303698 | 267.4199603 | 1333.308625 | 2227.629384 | 1486.865578 |
| MA2382 | 97.65352062 | 120.5241318 | 145.4904742 | 683.7685065 | 1142.408283 | 762.518022 |
| MA2381 | 122.5575842 | 151.260767 | 182.5941443 | 595.2934218 | 994.5882695 | 663.8532752 |
| MA2380 | 125.6909138 | 155.1279274 | 187.2623796 | 528.6407476 | 883.2281143 | 589.5242226 |
| MA2377 | 44.76317916 | 55.24678754 | 66.69105344 | 89.81902209 | 150.0654005 | 100.1634653 |
| MA2376 | 1009.130963 | 1245.471054 | 1503.46799 | 1924.043519 | 3214.601479 | 2145.63532 |
| MA2375 | 880.9213668 | 1087.234564 | 1312.453115 | 1537.797033 | 2569.279005 | 1714.904885 |
| MA2374 | 317.9622914 | 392.4295702 | 473.7206017 | 1132.401723 | 1891.963575 | 1262.820259 |
| MA2373 | 2052.225839 | 2532.860423 | 3057.537594 | 9802.60278 | 16377.72799 | 10931.56706 |
| MA2372 | 777.2091584 | 959.2327902 | 1157.93602 | 5880.315128 | 9824.554133 | 6557.550132 |
| MA2371 | 173.873831 | 214.5953612 | 259.048378 | 873.2462224 | 1458.978745 | 973.8178578 |
| MA2370 | 814.2989302 | 1005.009046 | 1213.194739 | 2606.84169 | 4355.38857 | 2907.071253 |
| MA2368 | 1442.317103 | 1780.110082 | 2148.856466 | 5561.373065 | 9291.680726 | 6201.87556 |
| MA2365 | 1183.706241 | 1460.932141 | 1763.561428 | 9216.105215 | 15397.83542 | 10277.52266 |
| MA2364 | 953.8664525 | 1177.263506 | 1421.13138 | 4808.773908 | 8034.273428 | 5362.599673 |
| MA2363 | 584.6251126 | 721.5452519 | 871.0119647 | 6267.27875 | 10471.07477 | 6989.080296 |
| MA2362 | 1492.324301 | 1841.829047 | 2223.360395 | 14071.7779 | 23510.46512 | 15692.42243 |
| MA2361 | 11.76861528 | 14.52484387 | 17.53363737 | 178.6753519 | 298.52238 | 199.2533652 |
| MA2360 | 22.52975476 | 27.80625947 | 33.5662727 | 82.13251026 | 137.2231378 | 91.59169906 |
| MA2359 | 132.1671098 | 163.120859 | 196.9110316 | 935.9852561 | 1563.800173 | 1043.782537 |
| MA2358 | 395.6149404 | 488.2685941 | 589.4124954 | 1400.172398 | 2339.342233 | 1561.430042 |
| MA2357 | 44.99897806 | 55.53781091 | 67.04236175 | 511.6875689 | 854.9035402 | 570.6185489 |
| MA2356 | 44.83446654 | 55.33477054 | 66.79726195 | 921.6200522 | 1539.799466 | 1027.762894 |
| MA2355 | 12.27795824 | 15.15347576 | 18.29248915 | 109.0826579 | 182.2501778 | 121.6456911 |
| MA2354 | 446.3126106 | 550.8397401 | 664.945134 | 1189.33262 | 1987.081041 | 1326.307878 |
| MA2353 | 600.9151065 | 741.6503885 | 895.2818417 | 2595.75697 | 4336.868742 | 2894.709908 |
| MA2352 | 136.0698329 | 167.9376061 | 202.7255586 | 537.2791329 | 897.6607225 | 599.1574895 |
| MA2351 | 171.491968 | 211.655662 | 255.4997258 | 376.1928229 | 628.525287 | 419.5188935 |
| MA2350 | 3882.597457 | 4791.908011 | 5784.54255 | 7779.55339 | 12997.71216 | 8675.523379 |
| MA2349 | 125.2198948 | 154.546595 | 186.5606253 | 1724.155408 | 2880.637819 | 1922.726126 |
| MA2348 | 249.3815431 | 307.7871006 | 371.5446072 | 2922.044584 | 4882.014751 | 3258.576017 |
| MA2347 | 401.5919833 | 495.64547 | 598.317477 | 1430.874292 | 2390.637514 | 1595.667868 |
| MA2346 | 208.7354768 | 257.6216604 | 310.9874924 | 1729.648424 | 2889.815291 | 1928.851771 |
| MA2345 | 908.6692713 | 1121.481073 | 1353.793722 | 1857.338256 | 3103.153461 | 2071.247622 |
| MA2344 | 259.2813153 | 320.0054153 | 386.2939223 | 753.7149813 | 1259.271565 | 840.5202217 |
| MA2343 | 50.53366272 | 62.3687276 | 75.28828971 | 261.1906921 | 436.385132 | 291.2719846 |
| MA2342 | 71.11484757 | 87.77005897 | 105.9514581 | 267.6989104 | 447.2587572 | 298.5297534 |
| MA2341 | 25.02266292 | 30.88301072 | 37.28036706 | 115.3978016 | 192.8012231 | 128.688149 |
| MA2340 | 51.35031803 | 63.3766449 | 76.50499514 | 294.5605623 | 492.1379428 | 328.4850577 |
| MA2339 | 694.0208067 | 856.561593 | 1033.996681 | 1093.321038 | 1826.669402 | 1219.238656 |
| MA2338 | 26.51141011 | 32.7204249 | 39.49839805 | 233.5840844 | 390.2613095 | 260.4859281 |
| MA2337 | 156.8902724 | 193.6342259 | 233.7451838 | 766.1850341 | 1280.105943 | 854.4264486 |
| MA2335 | 166.7802557 | 205.8404591 | 248.4799148 | 410.4901896 | 685.8277152 | 457.7662827 |
| MA2334a | 3.998383527 | 4.934811364 | 5.957048057 | 38.18083811 | 63.79074976 | 42.57811946 |
| MA2334 | 13.93340519 | 17.19663103 | 20.75888012 | 43.17639716 | 72.13709503 | 48.1490163 |
| MA2333 | 68.86049376 | 84.98773187 | 102.5927773 | 1702.31296 | 2844.144482 | 1898.368086 |
| MA2332 | 529.5291147 | 653.5456829 | 788.9264156 | 981.4478481 | 1639.756935 | 1094.481048 |
| MA2331 | 414.587466 | 511.6845156 | 617.67898 | 302.7996048 | 505.9033477 | 337.6729895 |
| MA2330 | 12.63802731 | 15.59787358 | 18.82894312 | 72.24624018 | 120.7056224 | 80.56682874 |
| MA2329 | 77.10617995 | 95.16457101 | 114.8777291 | 355.5480129 | 594.0329088 | 396.4964238 |
| MA2328 | 191.9326965 | 236.8836419 | 285.9536333 | 750.8668568 | 1254.513053 | 837.3440792 |
| MA2327 | 226.7246995 | 279.8239879 | 337.7889894 | 482.0353864 | 805.3620674 | 537.551329 |
| MA2326 | 254.9771576 | 314.693217 | 379.8813123 | 809.6539253 | 1352.731724 | 902.9016455 |
| MA2325 | 1518.146311 | 1873.698612 | 2261.831681 | 3054.478644 | 5103.279355 | 3406.262488 |
| MA2324 | 106.2995761 | 131.1951073 | 158.3719218 | 297.7064661 | 497.393971 | 331.9932748 |
| MA2323 | 942.8755243 | 1163.698485 | 1404.75639 | 3304.656424 | 5521.264631 | 3685.253205 |
| MA2322 | 693.2068433 | 855.5569982 | 1032.783987 | 1516.741763 | 2534.10085 | 1691.424683 |
| MA2321 | 83.81728144 | 103.4474232 | 124.8763582 | 305.1616017 | 509.8496611 | 340.3070172 |
| MA2320 | 1449.918622 | 1789.491889 | 2160.181696 | 1827.401653 | 3053.136791 | 2037.863225 |
| MA2319 | 400.8594449 | 494.74137 | 597.2260944 | 2265.078827 | 3784.387243 | 2525.947614 |
| MA2318 | 298.3148553 | 368.180673 | 444.4485921 | 338.7197186 | 565.9169857 | 377.7300175 |
| MA2316n | 281.8463785 | 347.8552525 | 419.9127998 | 549.4505201 | 917.9961041 | 612.7306534 |
| MA2315 | 53.13806103 | 65.58308018 | 79.16848925 | 308.8941124 | 516.0857645 | 344.4694006 |
| MA2314 | 46.26212102 | 57.09678398 | 68.92427309 | 245.987302 | 410.9840225 | 274.3176224 |
| MA2313 | 165.5648403 | 204.3403915 | 246.6691111 | 636.3021023 | 1063.103646 | 709.5849192 |
| MA2312 | 13.82595375 | 17.0640143 | 20.59879208 | 87.03459986 | 145.4133187 | 97.05836158 |
| MA2311 | 346.8324075 | 428.0611137 | 516.7331511 | 1575.03021 | 2631.486447 | 1756.426201 |
| MA2310 | 1648.738294 | 2034.875448 | 2456.395988 | 2724.801049 | 4552.469524 | 3038.615974 |
| MA2309 | 457.39509 | 564.5177538 | 681.4565221 | 536.227079 | 895.9030003 | 597.9842707 |
| MA2308 | 376.5458589 | 464.7335031 | 561.0021555 | 588.3666596 | 983.0153608 | 656.1287588 |
| MA2307 | 640.3782466 | 790.3558594 | 954.0765573 | 1566.407787 | 2617.080507 | 1746.810735 |
| MA2306 | 84.8971095 | 104.7801487 | 126.4851553 | 203.6857188 | 340.3085254 | 227.1441722 |
| MA2301n | 1167.797822 | 1441.297945 | 1739.860046 | 5774.650642 | 9648.014876 | 6439.716283 |
| MA2300 | 737.3776636 | 910.0726954 | 1098.592506 | 3431.017022 | 5732.381978 | 3826.166734 |
| MA2299 | 2154.699346 | 2659.33339 | 3210.209192 | 2413.578571 | 4032.493635 | 2691.55005 |
| MA2298 | 2396.737242 | 2958.05695 | 3570.812764 | 4065.574283 | 6792.570425 | 4533.805858 |
| MA2297 | 460.2113072 | 567.9935337 | 685.652303 | 669.2505825 | 1118.152418 | 746.3280709 |
| MA2296 | 117.0796214 | 144.4998566 | 174.4327243 | 87.35899969 | 145.9553107 | 97.42012248 |
| MA2295 | 981.0299564 | 1210.788746 | 1461.601309 | 1282.035252 | 2141.964242 | 1429.687059 |
| MA2294 | 401.1789669 | 495.1357246 | 597.7021387 | 1048.538322 | 1751.848546 | 1169.298322 |
| MA2292 | 1699.905028 | 2098.025513 | 2532.627467 | 2601.238208 | 4346.026536 | 2900.822419 |
| MA2291 | 295.1649372 | 364.2930391 | 439.7556423 | 930.2025023 | 1554.13862 | 1037.333784 |
| MA2290a | 47.61666907 | 58.76856937 | 70.94236561 | 182.6751698 | 305.2050877 | 203.7138415 |
| MA2290 | 34.61343423 | 42.71995606 | 51.56931288 | 160.5787831 | 268.2874834 | 179.0726173 |
| MA2289 | 428.2417971 | 528.5367131 | 638.0220778 | 1468.668286 | 2453.781943 | 1637.81459 |
| MA2288 | 11.79427442 | 14.55651241 | 17.57186599 | 281.9749544 | 471.1105007 | 314.4499673 |
| MA2287a | 29.36143396 | 36.2379289 | 43.74454626 | 50.82704072 | 84.9194307 | 56.68078332 |
| MA2287 | 571.6702134 | 705.5562946 | 851.7109256 | 639.8078301 | 1068.960851 | 713.4944012 |
| MA2286 | 855.6106474 | 1055.996034 | 1274.743583 | 1555.275248 | 2598.480784 | 1734.396063 |
| MA2284 | 2003.200784 | 2472.353622 | 2984.496925 | 4422.881951 | 7389.543282 | 4932.264596 |
| MA2283 | 21.73309216 | 26.82301722 | 32.3793537 | 181.4901481 | 303.2252092 | 202.392341 |
| MA2282 | 77.82783131 | 96.05523427 | 115.9528915 | 753.1057945 | 1258.253765 | 839.840875 |
| MA2281 | 201.0570193 | 248.1448958 | 299.5476343 | 1692.176996 | 2827.209791 | 1887.064765 |
| MA2280 | 240.4888045 | 296.8116683 | 358.2956352 | 2954.510911 | 4936.257964 | 3294.781486 |
| MA2279 | 54.76679391 | 67.59326491 | 81.59507989 | 165.2638259 | 276.1150326 | 184.2972357 |
| MA2278 | 939.8605686 | 1159.977422 | 1400.264516 | 1068.970655 | 1785.985928 | 1192.083844 |
| MA2277 | 112.1217644 | 138.3808615 | 167.0461911 | 378.935562 | 633.1077266 | 422.5775134 |
| MA4668 | 57.14733347 | 70.53133065 | 85.1417603 | 174.9258404 | 292.2578724 | 195.072023 |
| MA2275 | 493.0606627 | 608.5362609 | 734.5933783 | 3585.139116 | 5989.881928 | 3998.039046 |
| MA2274 | 73.26601849 | 90.42503757 | 109.1564104 | 718.1173966 | 1199.796794 | 800.8228686 |
| MA2273 | 426.9457614 | 526.9371436 | 636.0911607 | 3943.342495 | 6588.351298 | 4397.496654 |
| MA2272 | 615.3427687 | 759.4570324 | 916.7770977 | 4754.066939 | 7942.871594 | 5301.59211 |
| MA2271 | 158.1477112 | 195.1861589 | 235.6185967 | 535.2567859 | 894.2818801 | 596.902229 |
| MA2270 | 59.07018784 | 72.9045206 | 88.00655198 | 158.6888462 | 265.1298657 | 176.9650166 |
| MA2269 | 32.57650891 | 40.20597956 | 48.53457098 | 190.3927767 | 318.0993025 | 212.320284 |
| MA2268 | 422.9710613 | 522.0315624 | 630.1693977 | 641.5948714 | 1071.946555 | 715.4872557 |
| MA2267 | 215.056728 | 265.4233587 | 320.4052976 | 466.4037358 | 779.2454401 | 520.1193835 |
| MA2266 | 524.7005023 | 647.5862017 | 781.7324393 | 1225.991761 | 2048.329411 | 1367.189047 |
| MA2265 | 175.4677357 | 216.5625609 | 261.4230793 | 349.8377326 | 584.4924408 | 390.1284913 |
| MA2264 | 82.15519029 | 101.3960677 | 122.4000683 | 203.5889856 | 340.1469082 | 227.0362983 |
| MA2263 | 103.2036303 | 127.3740861 | 153.7593834 | 101.3717868 | 169.3672169 | 113.0467602 |
| MA2262 | 262.3089392 | 323.7421136 | 390.8046705 | 654.5576002 | 1093.604073 | 729.9429001 |
| MA2261 | 8.802868213 | 10.86451407 | 13.11507729 | 49.99232596 | 83.52482851 | 55.74993458 |
| MA2260 | 348.8274054 | 430.5233434 | 519.7054269 | 663.4756812 | 1108.503984 | 739.8880751 |
| MA2259 | 477.1412168 | 588.8884553 | 710.8755675 | 1087.888585 | 1817.593115 | 1213.180548 |
| MA2258 | 139.8808989 | 172.6412299 | 208.403529 | 389.4591433 | 650.6900316 | 434.313094 |
| MA2257 | 1885.03897 | 2326.518121 | 2808.451881 | 12803.06671 | 21390.7621 | 14277.59397 |
| MA2256 | 128.8830938 | 159.0677211 | 192.0182939 | 361.8382527 | 604.5423457 | 403.5111096 |
| MA4671 | 29.22691614 | 36.0719068 | 43.54413299 | 95.37867442 | 159.3542063 | 106.3634219 |
| MA2255 | 947.7658893 | 1169.734181 | 1412.04237 | 4008.496161 | 6697.206981 | 4470.154058 |
| MA2254 | 76.94066082 | 94.96028703 | 114.6311281 | 352.5072066 | 588.9524724 | 393.1054083 |
| MA2253 | 276.7336207 | 341.5450786 | 412.2954855 | 500.5662071 | 836.3224916 | 558.2163415 |
| MA2252 | 46.03717875 | 56.81915987 | 68.58913969 | 435.707223 | 727.9591494 | 485.887558 |
| MA2251 | 411.1219117 | 507.407323 | 612.5157752 | 1254.55179 | 2096.046164 | 1399.03833 |
| MA2250 | 257.5763096 | 317.9010946 | 383.7536956 | 520.6664947 | 869.9051074 | 580.6315762 |
| MA2249 | 94.7368783 | 116.9244072 | 141.1450735 | 284.2145278 | 474.8522747 | 316.9474721 |
| MA2248 | 51.58612758 | 63.66768142 | 76.85631932 | 573.4195041 | 958.042356 | 639.4601417 |
| MA2247 | 95.77527534 | 118.2059985 | 142.692144 | 258.8188906 | 432.4224377 | 288.6270232 |
| MA2246 | 377.4124148 | 465.8030078 | 562.2932061 | 1441.162547 | 2407.826647 | 1607.14102 |
| MA2245 | 18.01186609 | 22.23027402 | 26.83523258 | 33.81551185 | 56.49736782 | 37.71003924 |
| MA2244 | 611.2385841 | 754.3916411 | 910.6624203 | 5109.963915 | 8537.487535 | 5698.477687 |
| MA2243 | 496.784633 | 613.1323911 | 740.141588 | 2400.707774 | 4010.989713 | 2677.196926 |
| MA2241 | 233.2835658 | 287.9189512 | 347.5608088 | 725.0968921 | 1211.457807 | 808.6061915 |
| MA2240 | 59.27257027 | 73.15430133 | 88.30807431 | 97.88589516 | 163.5431529 | 109.1593989 |
| MA2239 | 122.3127849 | 150.9586354 | 182.2294267 | 1424.34407 | 2379.727126 | 1588.385561 |
| MA2238 | 1667.495877 | 2058.026087 | 2484.342237 | 3112.840664 | 5200.787874 | 3471.346053 |
| MA2237 | 5.426267406 | 6.697107939 | 8.084400982 | 49.9937903 | 83.52727507 | 55.75156758 |
| MA2236 | 32.16401577 | 39.69687986 | 47.92001227 | 203.1114168 | 339.3490088 | 226.503728 |
| MA2235 | 247.2409649 | 305.1451955 | 368.3554366 | 1964.657412 | 3282.457263 | 2190.92671 |
| MA2234 | 159.7811269 | 197.2021231 | 238.0521641 | 714.6132031 | 1193.942152 | 796.9150977 |
| MA2233 | 2845.875315 | 3512.38388 | 4239.967453 | 12228.1614 | 20430.23733 | 13636.4769 |
| MA2232 | 206.2443133 | 254.5470624 | 307.2759974 | 2251.484243 | 3761.674051 | 2510.787343 |
| MA2231 | 168.6080082 | 208.0962742 | 251.2030177 | 1421.324865 | 2374.682781 | 1585.018635 |
| MA2230 | 38.19604565 | 47.14162082 | 56.90691701 | 179.6018928 | 300.070401 | 200.2866157 |
| MA2229 | 38.03034229 | 46.93710947 | 56.66004153 | 215.3263477 | 359.7571412 | 240.1254507 |
| MA2228 | 128.4146892 | 158.4896154 | 191.3204347 | 708.9171373 | 1184.425433 | 790.5630168 |
| MA2226 | 13.55541437 | 16.73011416 | 20.19572517 | 59.20309391 | 98.91374669 | 66.02150529 |
| MA2225 | 207.151716 | 255.6669803 | 308.6279041 | 1223.161429 | 2043.600625 | 1364.032745 |
| MA2224 | 400.0133096 | 493.6970684 | 595.9654677 | 6595.924675 | 11020.16093 | 7355.576323 |
| MA2222 | 15828.96566 | 19536.13481 | 23583.00761 | 311.5117954 | 520.4592663 | 347.3885618 |
| MA2221 | 144764.5509 | 178668.6409 | 215679.5067 | 1635.396678 | 2732.343905 | 1823.745067 |
| MA2220 | 1834.819501 | 2264.537172 | 2733.631696 | 640.989974 | 1070.935921 | 714.8126925 |
| MA2219a | 1826.196918 | 2253.895166 | 2720.78522 | 2970.205096 | 4962.479071 | 3312.283168 |
| MA2218 | 43.98129964 | 54.28179058 | 65.52615922 | 63.95630959 | 106.855196 | 71.3221481 |
| MA2217 | 231.8443942 | 286.1427233 | 345.4166388 | 729.5996979 | 1218.980883 | 813.6275849 |
| MA2216 | 12.54366458 | 15.48141095 | 18.68835548 | 50.33990636 | 84.10554951 | 56.13754577 |
| MA2215 | 354.896872 | 438.0142888 | 528.7481073 | 689.7228094 | 1152.356452 | 769.1580811 |
| MA2214 | 99.74252887 | 123.1023892 | 148.6028126 | 1106.742416 | 1849.093211 | 1234.205773 |
| MA2213 | 42.54015277 | 52.5031248 | 63.37904624 | 878.0763377 | 1467.048674 | 979.2042567 |
| MA2212 | 84.548229 | 104.3495599 | 125.9653707 | 391.014262 | 653.2882509 | 436.0473155 |
| MA2211 | 216.1423168 | 266.7631941 | 322.0226773 | 1060.991123 | 1772.6541 | 1183.18531 |
| MA2210 | 149.9400448 | 185.0562438 | 223.3902892 | 589.5476833 | 984.9885598 | 657.4458009 |
| MA2209 | 169.236047 | 208.8714007 | 252.1387101 | 713.7296746 | 1192.465994 | 795.9298133 |
| MA2208 | 126.4495655 | 156.0642566 | 188.3926675 | 880.1521181 | 1470.516791 | 981.5191045 |
| MA2207 | 9.223144893 | 11.38322022 | 13.74123243 | 199.6151079 | 333.5075401 | 222.6047497 |
| MA2206 | 59.14770453 | 73.00019182 | 88.12204132 | 459.6672734 | 767.9904754 | 512.6070838 |
| MA2205 | 371.8543842 | 458.9432775 | 554.0124958 | 1653.171075 | 2762.040531 | 1843.566538 |
| MA2204 | 288.0735807 | 355.5408755 | 429.1904848 | 1254.721301 | 2096.329376 | 1399.227363 |
| MA2203 | 375.3935355 | 463.3113038 | 559.2853503 | 1632.695435 | 2727.830794 | 1820.732722 |
| MA2202 | 34.99110506 | 43.186078 | 52.1319911 | 27.64495586 | 46.18789291 | 30.82882125 |
| MA2200 | 805.7512404 | 994.4594737 | 1200.459843 | 2062.384307 | 3445.734766 | 2299.908796 |
| MA2199 | 116.112656 | 143.3064263 | 172.9920773 | 652.0289955 | 1089.3794 | 727.1230764 |
| MA2198 | 72.2874649 | 89.21730515 | 107.6984985 | 169.8941185 | 283.851108 | 189.4607985 |
| MA2197 | 64.69558238 | 79.84739157 | 96.38762534 | 176.0983973 | 294.2169254 | 196.3796231 |
| MA2196 | 36.5057942 | 45.05550977 | 54.38867207 | 53.93616674 | 90.11401233 | 60.14798691 |
| MA2195 | 26.39281116 | 32.57404988 | 39.32170174 | 79.83938231 | 133.3918874 | 89.0344719 |
| MA2194 | 145.1401782 | 179.1322408 | 216.2391403 | 371.7130224 | 621.0406468 | 414.523155 |
| MA2193 | 397.9226173 | 491.1167327 | 592.8506204 | 638.8395314 | 1067.343063 | 712.4145837 |
| MA2191 | 449.2261255 | 554.4356048 | 669.2858752 | 597.2479498 | 997.8538031 | 666.0329059 |
| MA2190 | 29.1276498 | 35.94939211 | 43.39623963 | 248.3022367 | 414.8517065 | 276.8991677 |
| MA2189 | 131.694117 | 162.5370904 | 196.2063365 | 333.9899544 | 558.0147179 | 372.455527 |
| MA2188 | 561.6837336 | 693.2309652 | 836.8324279 | 2494.891802 | 4168.347959 | 2782.228113 |
| MA2186 | 281.2957604 | 347.1756788 | 419.0924537 | 466.639364 | 779.6391168 | 520.382149 |
| MA2185 | 73.42962258 | 90.626958 | 109.4001582 | 157.0942478 | 262.4656856 | 175.1867685 |
| MA2184 | 155.8349392 | 192.3317319 | 232.1728807 | 1320.587716 | 2206.375887 | 1472.679604 |
| MA2183 | 137.8798264 | 170.171503 | 205.4222029 | 1553.335809 | 2595.240461 | 1732.233259 |
| MA2182 | 35.35323354 | 43.63301755 | 52.67151332 | 440.7425828 | 736.3719919 | 491.5028395 |
| MA2181 | 113.0954163 | 139.5825442 | 168.4968001 | 580.6483297 | 970.1199381 | 647.5215101 |
| MA2180 | 315.5462025 | 389.4476293 | 470.1209576 | 1543.037314 | 2578.034219 | 1720.748688 |
| MA2179 | 1124.485973 | 1387.842392 | 1675.331277 | 5585.267079 | 9331.601722 | 6228.521445 |
| MA2178 | 85.08021171 | 105.0061338 | 126.7579527 | 1367.906929 | 2285.434604 | 1525.448563 |
| MA2177 | 43.92196275 | 54.20855689 | 65.43775531 | 353.5807464 | 590.7460923 | 394.3025875 |
| MA2176 | 36.98594795 | 45.64811629 | 55.10403589 | 282.4523798 | 471.9081606 | 314.9823777 |
| MA2175 | 66.50591749 | 82.08171007 | 99.08477862 | 469.3140654 | 784.107882 | 523.3648953 |
| MA2174 | 38.69424007 | 47.75649318 | 57.649159 | 314.9295936 | 526.1695629 | 351.1999873 |
| MA2173 | 83.164013 | 102.6411583 | 123.903077 | 688.5861738 | 1150.457415 | 767.8905393 |
| MA2172 | 13.21791139 | 16.31356745 | 19.6928916 | 122.9266123 | 205.3800062 | 137.0840517 |
| MA2171 | 16.74323885 | 20.66453226 | 24.94515041 | 52.27569772 | 87.33977872 | 58.29628192 |
| MA2170 | 16.22079638 | 20.01973291 | 24.16678213 | 132.1168422 | 220.7346103 | 147.332719 |
| MA2169 | 196.4282494 | 242.4320606 | 292.6513962 | 270.9408959 | 452.6753144 | 302.145118 |
| MA2168 | 117.3938514 | 144.8876798 | 174.9008843 | 383.4849443 | 640.7086206 | 427.6508474 |
| MA2167 | 239.4732471 | 295.5582658 | 356.7825928 | 391.9177659 | 654.7977826 | 437.0548758 |
| MA2165 | 59.48914772 | 73.42160156 | 88.63074528 | 216.6029202 | 361.8899784 | 241.5490457 |
| MA2164 | 136.8271857 | 168.8723321 | 203.8539113 | 529.281732 | 884.2990409 | 590.239029 |
| MA2163 | 202.4519059 | 249.8664671 | 301.6258258 | 359.9984265 | 601.4684506 | 401.4593908 |
| MA2161n | 681.1968346 | 840.7342262 | 1014.890706 | 2111.746947 | 3528.207544 | 2354.956523 |
| MA2160 | 576.2009578 | 711.1481466 | 858.461119 | 630.7522303 | 1053.83118 | 703.3958693 |
| MA2159 | 938.4096569 | 1158.186704 | 1398.102855 | 1629.956473 | 2723.254666 | 1817.678314 |
| MA2158 | 27.54041883 | 33.99042911 | 41.0314812 | 242.1795947 | 404.6222841 | 270.0713821 |
| MA2157 | 162.8005197 | 200.9286626 | 242.550649 | 51.1248104 | 85.41693029 | 57.01284709 |
| MA2156 | 243.8270958 | 300.9317928 | 363.2692355 | 3983.80117 | 6655.947752 | 4442.614949 |
| MA2155 | 148.1264951 | 182.817958 | 220.6883467 | 1615.82524 | 2699.644866 | 1801.919589 |
| MA2154 | 61.60051263 | 76.02745152 | 91.77639204 | 483.6472139 | 808.055033 | 539.3487905 |
| MA2153 | 159.5208861 | 196.8809336 | 237.6644408 | 2282.072494 | 3812.779462 | 2544.898438 |
| MA2152 | 99.514193 | 122.8205767 | 148.2626232 | 948.3715981 | 1584.494691 | 1057.595412 |
| MA2151 | 72.78178845 | 89.82740008 | 108.4349734 | 776.5998087 | 1297.506459 | 866.0406913 |
| MA2150 | 128.1675143 | 158.1845518 | 190.9521778 | 887.3284518 | 1482.506672 | 989.5219355 |
| MA2149 | 148.2907753 | 183.0207127 | 220.9331018 | 669.2042077 | 1118.074937 | 746.2763552 |
| MA2148 | 510.6360401 | 630.22782 | 760.7783022 | 2550.767424 | 4261.702323 | 2844.538923 |
| MA2147 | 342.9891752 | 423.3177903 | 511.0072573 | 6828.603705 | 11408.91012 | 7615.052962 |
| MA2146 | 497.8514165 | 614.4490169 | 741.7309504 | 4101.855677 | 6853.187673 | 4574.265775 |
| MA2145 | 920.5928516 | 1136.197174 | 1371.558236 | 7950.98398 | 13284.13033 | 8866.697603 |
| MA2144 | 764.5304251 | 943.5846773 | 1139.04643 | 10952.61933 | 18299.1216 | 12214.03085 |
| MA2143 | 1609.242912 | 1986.130184 | 2397.553236 | 24135.23229 | 40324.01173 | 26914.88335 |
| MA2142 | 1585.041949 | 1956.261317 | 2361.497091 | 10719.20103 | 17909.13726 | 11953.72979 |
| MA2141 | 16.15389278 | 19.93716039 | 24.06710486 | 66.74527139 | 111.5148623 | 74.43231421 |
| MA2140 | 85.4896065 | 105.5114095 | 127.3678953 | 385.8348576 | 644.6347454 | 430.2713999 |
| MA2139 | 84.36237817 | 104.1201825 | 125.6884783 | 686.3511662 | 1146.723269 | 765.3981263 |
| MA2138 | 621.612599 | 767.195267 | 926.1182927 | 2494.800309 | 4168.195098 | 2782.126084 |
| MA2137 | 101.9274071 | 125.7989694 | 151.8579842 | 1068.678629 | 1785.498024 | 1191.758185 |
| MA2136 | 134.1435726 | 165.5602126 | 199.8556926 | 421.4067997 | 704.0666743 | 469.9401571 |
| MA2135 | 278.0426522 | 343.1606874 | 414.2457646 | 476.3791275 | 795.9118558 | 531.2436396 |
| MA2134 | 1018.787862 | 1257.389614 | 1517.855457 | 2191.542746 | 3661.526615 | 2443.942393 |
| MA2133 | 1243.713655 | 1534.993388 | 1852.964319 | 3682.423417 | 6152.419966 | 4106.527565 |
| MA2132 | 903.1701814 | 1114.694087 | 1345.600826 | 1715.421609 | 2866.045797 | 1912.986456 |
| MA2131a | 16.08795258 | 19.85577688 | 23.96886292 | 72.41692743 | 120.9907987 | 80.75717402 |
| MA2131 | 183.0335977 | 225.9003599 | 272.6951855 | 325.6555185 | 544.0899341 | 363.1612154 |
| MA2130 | 200.1889407 | 247.0735118 | 298.2543151 | 278.7395005 | 465.7048564 | 310.8418867 |
| MA2129 | 204.9923507 | 253.0018881 | 305.4107432 | 1117.023692 | 1866.270683 | 1245.671141 |
| MA2127 | 11.56243719 | 14.27037853 | 17.22646002 | 71.3651888 | 119.2336032 | 79.58430681 |
| MA2125 | 40.91812829 | 50.50121958 | 60.9624502 | 34.23074223 | 57.19111523 | 38.17309164 |
| MA2124 | 386.1885361 | 476.6345109 | 575.3684342 | 1792.933755 | 2995.549448 | 1999.425665 |
| MA2123 | 29.00424512 | 35.79708586 | 43.21238343 | 90.00616523 | 150.3780705 | 100.3721617 |
| MA2122 | 2018.589755 | 2491.346714 | 3007.4244 | 3806.041931 | 6358.955969 | 4244.383205 |
| MA2121 | 519.8457911 | 641.5945093 | 774.4995794 | 861.1618729 | 1438.788782 | 960.3417557 |
| MA2120 | 379.2910971 | 468.1216806 | 565.0921874 | 1252.42009 | 2092.484621 | 1396.661122 |
| MA2119 | 479.0851475 | 591.2876577 | 713.7717601 | 2252.310004 | 3763.053694 | 2511.708207 |
| MA2118 | 126.1579697 | 155.7043687 | 187.9582295 | 1012.130361 | 1691.019836 | 1128.697262 |
| MA2117 | 59.53682346 | 73.48044304 | 88.70177565 | 388.5165505 | 649.1151919 | 433.2619429 |
| MA2116 | 241.8232618 | 298.4586577 | 360.2837953 | 1656.48675 | 2767.580206 | 1847.264079 |
| MA2115 | 26.35275399 | 32.52461125 | 39.26202199 | 565.5248736 | 944.8523785 | 630.6562878 |
| MA2114 | 346.9368755 | 428.1900484 | 516.8887943 | 713.4047246 | 1191.923083 | 795.5674389 |
| MA2113 | 646.2697976 | 797.627221 | 962.8541675 | 4339.067979 | 7249.510838 | 4838.797783 |
| MA2111 | 1012.447571 | 1249.564416 | 1508.409284 | 3521.83323 | 5884.113431 | 3927.442231 |
| MA2110 | 702.8043711 | 867.4022823 | 1047.082999 | 3264.124584 | 5453.545937 | 3640.053319 |
| MA2109 | 101.2827889 | 125.0033806 | 150.8975907 | 614.8149598 | 1027.203938 | 685.6231058 |
| MA2108 | 77.88412776 | 96.12471543 | 116.0367656 | 469.9873471 | 785.2327695 | 524.1157188 |
| MA2107 | 53.92949112 | 66.55986446 | 80.34761252 | 193.8903915 | 323.9429531 | 216.2207186 |
| MA2106 | 359.8912221 | 444.1783237 | 536.1890102 | 1311.318641 | 2190.889551 | 1462.34301 |
| MA2105 | 28.2373587 | 34.85059341 | 42.06982689 | 339.3062654 | 566.8969604 | 378.3841167 |
| MA2103 | 17.72573989 | 21.87713661 | 26.40894343 | 76.57023522 | 127.9299501 | 85.38881764 |
| MA2102 | 237.0247613 | 292.5363407 | 353.1346816 | 3867.23384 | 6461.192535 | 4312.622577 |
| MA2100 | 34.94986746 | 43.13518249 | 52.07055268 | 175.4983598 | 293.2144109 | 195.7104794 |
| MA2099 | 320.3591093 | 395.3877266 | 477.2915346 | 1374.85243 | 2297.038821 | 1533.193977 |
| MA2098 | 134.5963147 | 166.1189875 | 200.5302166 | 982.4859368 | 1641.491325 | 1095.638694 |
| MA2097 | 1361.674722 | 1680.581126 | 2028.710278 | 7661.759211 | 12800.90718 | 8544.162861 |
| MA2096 | 622.9270363 | 768.8175476 | 928.0766256 | 1729.33717 | 2889.295263 | 1928.504671 |
| MA2093 | 206.4248962 | 254.7699381 | 307.5450413 | 552.4023698 | 922.9279158 | 616.0224672 |
| MA2092 | 84.8235428 | 104.6893526 | 126.3755509 | 172.3523687 | 287.9582368 | 192.2021649 |
| MA2091 | 153.1101434 | 188.9687846 | 228.1133054 | 420.0291099 | 701.7648949 | 468.403799 |
| MA2090 | 17.57719912 | 21.69380736 | 26.18763788 | 78.61243136 | 131.3419554 | 87.6662132 |
| MA2089 | 73.38891524 | 90.57671693 | 109.3395098 | 284.7194668 | 475.695903 | 317.5105648 |
| MA2088 | 313.0524456 | 386.3698307 | 466.4055988 | 784.7744701 | 1311.164299 | 875.1568272 |
| MA2086 | 19.74451238 | 24.36870885 | 29.41664008 | 88.16492034 | 147.301805 | 98.31886089 |
| MA2085 | 194.6868776 | 240.2828567 | 290.0569888 | 959.1687864 | 1602.534126 | 1069.636111 |
| MA2084 | 255.9213072 | 315.8584879 | 381.2879669 | 935.222412 | 1562.525649 | 1042.931836 |
| MA2082 | 982.7095639 | 1212.86172 | 1464.103696 | 3398.315489 | 5677.745794 | 3789.69897 |
| MA2081 | 65.06001089 | 80.29716982 | 96.93057429 | 211.1600147 | 352.7962278 | 235.4792817 |
| MA2078 | 2772.827985 | 3422.228748 | 4131.136859 | 5829.766787 | 9740.100341 | 6501.180147 |
| MA2077 | 308.2418831 | 380.4326267 | 459.2385144 | 500.2748035 | 835.8356281 | 557.891377 |
| MA2076 | 794.6238383 | 980.7260161 | 1183.881526 | 2305.987219 | 3852.735062 | 2571.567419 |
| MA2075 | 331.6308327 | 409.2993057 | 494.0848706 | 1756.96495 | 2935.454459 | 1959.314338 |
| MA2074 | 201.4505185 | 248.6305531 | 300.1338945 | 892.0437245 | 1490.384728 | 994.7802654 |
| MA2073 | 70.95995346 | 87.5788884 | 105.7206869 | 237.2754442 | 396.4286599 | 264.6024212 |
| MA2071n | 134.2019933 | 165.6323154 | 199.9427314 | 606.7583058 | 1013.743259 | 676.6385681 |
| MA2070 | 7.854276855 | 9.693761096 | 11.70180508 | 40.67036865 | 67.95014038 | 45.35436886 |
| MA2066n | 63.59379786 | 78.48756735 | 94.74611614 | 150.6421917 | 251.6858934 | 167.9916301 |
| MA2065 | 34.73846672 | 42.87427135 | 51.7555943 | 61.15556218 | 102.1758389 | 68.19883903 |
| MA2064 | 119.8434461 | 147.9109734 | 178.5504474 | 1308.431789 | 2186.066336 | 1459.123681 |
| MA2063 | 196.9578316 | 243.0856718 | 293.4404017 | 557.1387419 | 930.8412238 | 621.3043266 |
| MA2062 | 24.75275845 | 30.54989419 | 36.87824608 | 78.77431337 | 131.6124203 | 87.84673913 |
| MA2061 | 101.3306964 | 125.0625081 | 150.9689664 | 205.7416581 | 343.7434923 | 229.4368938 |
| MA2059 | 1259.210831 | 1554.120027 | 1876.053005 | 2585.737778 | 4320.129146 | 2883.536807 |
| MA2058 | 371.3403787 | 458.3088911 | 553.2466975 | 1791.895189 | 2993.814261 | 1998.267488 |
| MA2057 | 22.89861054 | 28.26150187 | 34.11581769 | 108.0711272 | 180.5601598 | 120.5176625 |
| MA2056 | 253.0806568 | 312.3525527 | 377.0557839 | 992.8359228 | 1658.783595 | 1107.180686 |
| MA2055 | 690.8207644 | 852.6120957 | 1029.229053 | 2266.940755 | 3787.498065 | 2528.023979 |
| MA2054 | 18.03883958 | 22.26356475 | 26.87541942 | 64.26252858 | 107.3668123 | 71.66363428 |
| MA2053 | 82.70571323 | 102.075524 | 123.2202726 | 356.89822 | 596.2887714 | 398.0021341 |
| MA2052 | 323.4040629 | 399.1458133 | 481.8281017 | 880.7777652 | 1471.562092 | 982.2168073 |
| MA2051 | 72.34400705 | 89.28708957 | 107.7827386 | 726.0098837 | 1212.98319 | 809.6243323 |
| MA2050 | 65.22636537 | 80.50248478 | 97.17841985 | 353.9206474 | 591.313983 | 394.6816349 |
| MA2049 | 32.9413234 | 40.6562342 | 49.07809501 | 215.3127117 | 359.7343587 | 240.1102441 |
| MA2048a | 22.16328884 | 27.35396664 | 33.02028828 | 154.7952272 | 258.6245901 | 172.6229702 |
| MA2048 | 2.662845916 | 3.286488701 | 3.967278522 | 58.35857514 | 97.50276442 | 65.07972342 |
| MA2047 | 62.07453883 | 76.61249541 | 92.48262666 | 640.8919162 | 1070.772091 | 714.7033413 |
| MA2046 | 130.3163176 | 160.8366082 | 194.1536027 | 1597.46148 | 2668.963558 | 1781.440877 |
| MA2045 | 811.6894147 | 1001.788378 | 1209.306916 | 4729.020273 | 7901.024802 | 5273.660823 |
| MA2044 | 63.46451702 | 78.32800873 | 94.55350526 | 1080.749607 | 1805.665647 | 1205.219376 |
| MA2043 | 150.3440259 | 185.554838 | 223.9921662 | 1004.401037 | 1678.106044 | 1120.077753 |
| MA2042 | 43.25984445 | 53.39136942 | 64.45128903 | 426.2431833 | 712.1470767 | 475.3335463 |
| MA2041 | 93.2666521 | 115.1098517 | 138.9546363 | 571.9009348 | 955.5052018 | 637.766679 |
| MA2040 | 34.12428165 | 42.11624316 | 50.84054202 | 195.1476008 | 326.0434393 | 217.6227205 |
| MA2039 | 53.56069662 | 66.1046977 | 79.79815884 | 343.1115252 | 573.2546096 | 382.6276277 |
| MA2038 | 144.0028518 | 177.7285507 | 214.5446785 | 596.0726107 | 995.8901017 | 664.7222032 |
| MA2037 | 189.0744218 | 233.3559547 | 281.6951924 | 981.712862 | 1640.199708 | 1094.776584 |
| MA2036 | 129.8100212 | 160.2117364 | 193.3992898 | 968.6522756 | 1618.378694 | 1080.211812 |
| MA2035 | 88.84570181 | 109.6535077 | 132.3680212 | 143.7020588 | 240.0906457 | 160.2522032 |
| MA2034 | 317.711514 | 392.1200603 | 473.3469775 | 410.1484075 | 685.2566818 | 457.3851376 |
| MA2033 | 399.9194936 | 493.5812807 | 595.8256948 | 1339.79958 | 2238.474165 | 1494.104095 |
| MA2032 | 1267.301301 | 1564.105298 | 1888.106706 | 3853.429762 | 6438.129331 | 4297.228686 |
| MA2031 | 16.0934241 | 19.86252984 | 23.97701474 | 68.60037526 | 114.6142827 | 76.50107011 |
| MA2030 | 50.74396037 | 62.6282773 | 75.60160463 | 205.4550944 | 343.264715 | 229.1173266 |
| MA2029 | 430.8129341 | 531.7100145 | 641.8527225 | 2487.264719 | 4155.604988 | 2773.722621 |
| MA2026 | 63.73535932 | 78.66228273 | 94.95702348 | 286.3147175 | 478.3611729 | 319.2895403 |
| MA2025 | 208.1540337 | 256.9040424 | 310.1212212 | 1836.847824 | 3068.91901 | 2048.39731 |
| MA2024 | 782.8267397 | 966.1660181 | 1166.305453 | 4137.753709 | 6913.164417 | 4614.298178 |
| MA2023 | 529.3774338 | 653.3584781 | 788.7004317 | 1878.266706 | 3138.119732 | 2094.586399 |
| MA2022 | 45.92158151 | 56.67648957 | 68.41691551 | 175.0801046 | 292.5156097 | 195.2440538 |
| MA2021 | 192.6562359 | 237.7766354 | 287.0316087 | 620.1037951 | 1036.040275 | 691.5210554 |
| MA2020 | 772.261953 | 953.1269414 | 1150.565356 | 785.7334876 | 1312.766581 | 876.2262946 |
| MA2019 | 2277.500011 | 2810.894168 | 3393.165494 | 4786.224175 | 7996.598393 | 5337.45289 |
| MA2018 | 26.90071164 | 33.20090147 | 40.07840443 | 87.61947796 | 146.3905055 | 97.71059999 |
| MA2017 | 700.402579 | 864.4379867 | 1043.504655 | 1440.859253 | 2407.319917 | 1606.802795 |
| MA2016 | 51.9030767 | 64.05886054 | 77.32853043 | 172.0719165 | 287.4896704 | 191.8894131 |
| MA2015 | 182.0197996 | 224.6491287 | 271.1847642 | 1115.214479 | 1863.247935 | 1243.653562 |
| MA2014 | 260.0294161 | 320.9287225 | 387.4084908 | 1935.005038 | 3232.915471 | 2157.859276 |
| MA2013 | 2464.877889 | 3042.156245 | 3672.333067 | 1690.555364 | 2824.500445 | 1885.25637 |
| MA2012 | 101.0807424 | 124.7540145 | 150.5965689 | 153.0971114 | 255.7874579 | 170.7292826 |
| MA2011 | 217.7744454 | 268.7775699 | 324.4543272 | 310.1844791 | 518.2416486 | 345.9083787 |
| MA2010 | 143.4781871 | 177.0810087 | 213.7629994 | 254.7850735 | 425.6829255 | 284.1286319 |
| MA2009 | 279.7969936 | 345.3258984 | 416.8594948 | 900.1876832 | 1503.991271 | 1003.862163 |
| MA2008 | 188.2392137 | 232.3251395 | 280.4508458 | 1099.966585 | 1837.77247 | 1226.64957 |
| MA2006 | 172.5517522 | 212.9636494 | 257.0786603 | 651.2293081 | 1088.043319 | 726.2312891 |
| MA2005 | 101.0101018 | 124.6668297 | 150.491324 | 1054.323079 | 1761.513446 | 1175.749309 |
| MA2004 | 317.7952423 | 392.2233979 | 473.4717212 | 2869.49475 | 4794.216959 | 3199.974027 |
| MA2003 | 2145.459945 | 2647.930106 | 3196.443741 | 1017.182129 | 1699.460091 | 1134.330841 |
| MA2002 | 375.6243799 | 463.5962123 | 559.6292771 | 831.8961373 | 1389.892966 | 927.7054897 |
| MA2001 | 1137.483002 | 1403.883346 | 1694.695085 | 900.0326319 | 1503.732219 | 1003.689254 |
| MA2000 | 530.0983935 | 654.2482877 | 789.7745636 | 1444.937475 | 2414.133619 | 1611.350705 |
| MA1999 | 221.2059329 | 273.0127172 | 329.5667771 | 460.9736135 | 770.173047 | 514.0638748 |
| MA1998 | 584.3157701 | 721.1634609 | 870.5510864 | 483.4979682 | 807.8056802 | 539.1823561 |
| MA1997 | 295.0662711 | 364.1712652 | 439.6086432 | 844.2067108 | 1410.460894 | 941.4338701 |
| MA1996 | 90.45268946 | 111.6368545 | 134.7622144 | 294.6973123 | 492.3664183 | 328.6375572 |
| MA1995 | 213.9362725 | 264.0404908 | 318.7359712 | 1665.008277 | 2781.817572 | 1856.767029 |
| MA1994 | 25.74040082 | 31.76884399 | 38.34969899 | 104.6299003 | 174.8107196 | 116.6801101 |
| MA1993 | 90.31678921 | 111.4691261 | 134.5597415 | 434.1287787 | 725.3219589 | 484.1273246 |
| MA1991 | 5687.680993 | 7019.745007 | 8473.8717 | 17888.50551 | 29887.27422 | 19948.72199 |
| MA1990 | 201.2310619 | 248.3596994 | 299.8069341 | 274.8901318 | 459.2735121 | 306.5491868 |
| MA1989 | 7539.615025 | 9305.40496 | 11233.00172 | 8308.207918 | 13880.96329 | 9265.062968 |
| MA1988 | 106.0669841 | 130.908042 | 158.0253914 | 428.660131 | 716.1851992 | 478.0288535 |
| MA1987 | 24.77530491 | 30.57772107 | 36.91183725 | 88.92387227 | 148.5698263 | 99.1652212 |
| MA1986 | 180.8746092 | 223.2357329 | 269.4785861 | 1077.526608 | 1800.280812 | 1201.625185 |
| MA1985 | 86.51492397 | 106.776858 | 128.895479 | 171.656109 | 286.7949589 | 191.4257172 |
| MA1984 | 52.14605494 | 64.35874467 | 77.69053499 | 202.0071993 | 337.5041341 | 225.2723379 |
| MA1983 | 31.29754153 | 38.62747597 | 46.62908341 | 142.0114771 | 237.2661012 | 158.3669174 |
| MA1982a | 112.1810232 | 138.4539988 | 167.1344786 | 491.4671528 | 821.1202193 | 548.0693505 |
| MA1982 | 765.974297 | 945.3667062 | 1141.197603 | 7084.464326 | 11836.39002 | 7900.381012 |
| MA1981 | 1144.338193 | 1412.344034 | 1704.908387 | 7474.008223 | 12487.22165 | 8334.78862 |
| MA1980 | 81.32676298 | 100.3736214 | 121.1658242 | 459.6779508 | 768.0083147 | 512.6189909 |
| MA1979 | 107.4500054 | 132.6149691 | 160.0859051 | 373.7857447 | 624.5036539 | 416.8345924 |
| MA1978 | 959.6649698 | 1184.420045 | 1429.770382 | 2349.563783 | 3925.540736 | 2620.162689 |
| MA1977 | 352.72397 | 435.3324897 | 525.5107786 | 1086.547027 | 1815.351703 | 1211.684484 |
| MA1976 | 162.6521239 | 200.7455123 | 242.3295595 | 1233.271061 | 2060.491324 | 1375.306703 |
| MA1975 | 75.22014418 | 92.83682262 | 112.0677921 | 797.4111465 | 1332.277064 | 889.2488677 |
| MA1974 | 152.0766395 | 187.6932325 | 226.573525 | 1993.668237 | 3330.927185 | 2223.278706 |
| MA1973 | 193.6898592 | 239.052335 | 288.5715669 | 653.249141 | 1091.417961 | 728.4837458 |
| MA1972 | 530.1435786 | 654.3040552 | 789.8418833 | 684.6018964 | 1143.800671 | 763.4473934 |
| MA1971 | 763.1023525 | 941.8221478 | 1136.918796 | 1705.786794 | 2849.948402 | 1902.242001 |
| MA1970 | 324.8767482 | 400.9634039 | 484.0222027 | 758.1470085 | 1266.676388 | 845.4626849 |
| MA1969 | 178.6660651 | 220.5099442 | 266.1881556 | 503.6013253 | 841.3934245 | 561.6010139 |
| MA1968 | 351.3373192 | 433.621083 | 523.4448573 | 818.9088203 | 1368.194367 | 913.2224253 |
| MA1967 | 2858.074065 | 3527.439597 | 4258.141933 | 4146.765762 | 6928.221332 | 4624.348148 |
| MA1966 | 689.5024634 | 850.9850465 | 1027.264964 | 2919.007604 | 4876.940708 | 3255.189269 |
| MA1965 | 5256.627112 | 6487.737615 | 7831.659998 | 1743.301886 | 2912.626853 | 1944.0777 |
| MA1964 | 345.9408608 | 426.9607654 | 515.4048677 | 2557.419201 | 4272.815798 | 2851.956783 |
| MA1963 | 505.4729858 | 623.8555701 | 753.0860529 | 4068.45754 | 6797.387635 | 4537.02118 |
| MA1962 | 98.67814899 | 121.7887298 | 147.0170312 | 289.4830747 | 483.6547151 | 322.8227966 |
| MA1961 | 348.6860414 | 430.3488717 | 519.4948138 | 1201.771067 | 2007.862614 | 1340.178859 |
| MA1959 | 301.9904714 | 372.7171244 | 449.9247606 | 1503.619522 | 2512.176825 | 1676.791155 |
| MA1958 | 1397.677007 | 1725.015204 | 2082.34879 | 2078.208822 | 3472.173621 | 2317.555818 |
| MA1957 | 27.66908555 | 34.14922978 | 41.22317713 | 119.4751672 | 199.6134938 | 133.2351041 |
| MA1956 | 703.6883916 | 868.4933419 | 1048.400069 | 7463.986171 | 12470.47728 | 8323.61233 |
| MA1955 | 191.5951628 | 236.4670574 | 285.450754 | 908.2649474 | 1517.486384 | 1012.869684 |
| MA1954 | 377.86321 | 466.35938 | 562.9648297 | 1139.503474 | 1903.828847 | 1270.739917 |
| MA1953 | 187.4526407 | 231.3543499 | 279.2789591 | 1148.245882 | 1918.435251 | 1280.489187 |
| MA1952 | 39.68171899 | 48.97524124 | 59.12036838 | 106.9561284 | 178.6972723 | 119.2742496 |
| MA1951 | 606.2496325 | 748.234269 | 903.2295604 | 1809.467771 | 3023.173705 | 2017.863901 |
| MA1950 | 442.5809752 | 546.2341496 | 659.3855043 | 710.0606654 | 1186.335986 | 791.8382449 |
| MA1949 | 901.3960938 | 1112.504505 | 1342.957676 | 1493.638915 | 2495.501696 | 1665.661083 |
| MA1948 | 1907.13226 | 2353.785695 | 2841.36788 | 6754.232499 | 11284.6542 | 7532.116435 |
| MA1947 | 117.7203391 | 145.2906314 | 175.3873066 | 542.6695625 | 906.6667988 | 605.168734 |
| MA1946 | 363.6583057 | 448.8276644 | 541.8014528 | 1088.093703 | 1817.935817 | 1213.40929 |
| MA1945 | 162.4075897 | 200.4437078 | 241.9652368 | 289.4733188 | 483.6384155 | 322.8119172 |
| MA1944 | 156.1408568 | 192.709296 | 232.6286564 | 400.6271622 | 669.3490326 | 446.7673319 |
| MA1943 | 383.6101565 | 473.4522706 | 571.5269989 | 2134.333822 | 3565.944633 | 2380.144726 |
| MA1942 | 39.00601021 | 48.14128038 | 58.11365413 | 656.8371142 | 1097.412578 | 732.4849453 |
| MA1941 | 877.5108868 | 1083.025344 | 1307.371964 | 3055.115405 | 5104.343224 | 3406.972584 |
| MA1940 | 338.1039736 | 417.2884666 | 503.7289708 | 692.0132927 | 1156.183284 | 771.7123591 |
| MA1939a | 68.81768293 | 84.93489468 | 102.5289949 | 194.6995163 | 325.2948008 | 217.1230302 |
| MA1939 | 142.013545 | 175.273345 | 211.5808818 | 331.1519926 | 553.2731847 | 369.2907176 |
| MA1938 | 17.12713508 | 21.1383376 | 25.51710362 | 69.38801515 | 115.9302344 | 77.37942236 |
| MA1937 | 462.8952989 | 571.3061206 | 689.651086 | 3002.231323 | 5015.986986 | 3347.997851 |
| MA1936 | 746.355862 | 921.1536023 | 1111.968801 | 4004.93439 | 6691.256141 | 4466.182078 |
| MA1935 | 87.26152749 | 107.6983172 | 130.0078167 | 474.801383 | 793.2758344 | 529.4841865 |
| MA1934 | 201.6963868 | 248.9340041 | 300.5002049 | 803.0377049 | 1341.677654 | 895.523436 |
| MA1933 | 147.9245667 | 182.5687375 | 220.3875008 | 703.4832469 | 1175.346744 | 784.5033061 |
| MA1932 | 23.49649006 | 28.99940573 | 35.00657691 | 142.3035155 | 237.7540253 | 158.6925899 |
| MA1931 | 43.77860462 | 54.03162406 | 65.22417119 | 216.6136214 | 361.9078576 | 241.5609795 |
| MA1929 | 38.659763 | 47.71394152 | 57.59779285 | 164.7229077 | 275.2112921 | 183.6940201 |
| MA1928 | 58.45489676 | 72.14512736 | 87.0898519 | 194.1531585 | 324.3819717 | 216.5137484 |
| MA1927 | 2.617105063 | 3.230035268 | 3.899130869 | 49.86518829 | 83.31241287 | 55.60815449 |
| MA1926 | 59.64008653 | 73.60789048 | 88.85562359 | 1331.521617 | 2224.643734 | 1484.87276 |
| MA1925 | 890.4770362 | 1099.028187 | 1326.689764 | 2401.571207 | 4012.432296 | 2678.1598 |
| MA1924 | 23.55146473 | 29.06725556 | 35.08848171 | 157.0537549 | 262.398032 | 175.1416122 |
| MA1923 | 408.4351893 | 504.0913659 | 608.512924 | 3025.622529 | 5055.067914 | 3374.083019 |
| MA1922 | 748.0404611 | 923.2327372 | 1114.478625 | 5794.334636 | 9680.901968 | 6461.66728 |
| MA1921 | 868.0469067 | 1071.344885 | 1293.27192 | 3102.38074 | 5183.311925 | 3459.681461 |
| MA1920 | 151.7318856 | 187.2677367 | 226.0598886 | 1601.938837 | 2676.444116 | 1786.433891 |
| MA1919 | 376.1940186 | 464.2992613 | 560.4779613 | 2182.112552 | 3645.771089 | 2433.426124 |
| MA1917n | 116.6110054 | 143.92149 | 173.7345502 | 383.0797804 | 640.0316919 | 427.1990208 |
| MA1916 | 76.98363896 | 95.01333071 | 114.6951596 | 354.2330081 | 591.8358605 | 395.0299701 |
| MA1915 | 399.477209 | 493.0354122 | 595.1667507 | 1758.832136 | 2938.574066 | 1961.396568 |
| MA1914 | 217.9379675 | 268.9793892 | 324.6979529 | 1161.122377 | 1939.948694 | 1294.848666 |
| MA1913 | 51.27527366 | 63.28402502 | 76.39318923 | 396.1195044 | 661.8178499 | 441.7405279 |
| MA1912 | 17.4481732 | 21.53456335 | 25.99540678 | 176.5832694 | 295.027027 | 196.9203379 |
| MA1911 | 32.8908822 | 40.59397958 | 49.00294447 | 338.3524498 | 565.3033701 | 377.3204503 |
| MA1910 | 61.37464456 | 75.74868478 | 91.43987931 | 373.2473116 | 623.6040651 | 416.234148 |
| MA1909 | 87.4176164 | 107.8909624 | 130.240368 | 737.8033702 | 1232.687193 | 822.7760728 |
| MA1908 | 18.08877145 | 22.32519074 | 26.94981112 | 66.22564695 | 110.6466982 | 73.8528447 |
| MA1907 | 76.81886639 | 94.80996814 | 114.4496709 | 863.5540128 | 1442.785456 | 963.0093979 |
| MA1906 | 2087.505515 | 2576.402656 | 3110.099516 | 15639.91924 | 26130.44196 | 17441.16637 |
| MA1905 | 157.0171255 | 193.7907882 | 233.9341776 | 259.3589912 | 433.3248122 | 289.229327 |
| MA1904 | 1784.414295 | 2202.326986 | 2658.53479 | 4765.727552 | 7962.353599 | 5314.595674 |
| MA1903 | 203.2508736 | 250.8525542 | 302.816179 | 810.996877 | 1354.975464 | 904.3992648 |
| MA1902 | 418.0387323 | 515.9440741 | 622.8208978 | 676.1976731 | 1129.75929 | 754.0752569 |
| MA1901 | 611.2848203 | 754.448706 | 910.731306 | 1030.644082 | 1721.951691 | 1149.343205 |
| MA1899 | 57.09984567 | 70.47272113 | 85.07100993 | 312.7192031 | 522.4765464 | 348.7350265 |
| MA1898 | 40.18742991 | 49.59939047 | 59.87380892 | 113.1468957 | 189.0405152 | 126.1780065 |
| MA1897 | 51.65999415 | 63.75884766 | 76.96637046 | 148.0506965 | 247.3561451 | 165.1016728 |
| MA1895 | 1088.328032 | 1343.216203 | 1621.460859 | 2052.131961 | 3428.605628 | 2288.475689 |
| MA1894 | 82.85287799 | 102.2571549 | 123.4395281 | 112.1202835 | 187.3252999 | 125.0331596 |
| MA1893 | 513.6813353 | 633.9863282 | 765.3153781 | 2319.014324 | 3874.500135 | 2586.094853 |
| MA1892 | 1166.397152 | 1439.569236 | 1737.773238 | 6259.015105 | 10457.26826 | 6979.864928 |
| MA1891 | 241.7810488 | 298.4065583 | 360.2209036 | 728.6191357 | 1217.342606 | 812.5340915 |
| MA1889 | 35.14141828 | 43.37159482 | 52.35593736 | 229.6590672 | 383.7035752 | 256.1088673 |
| MA1888 | 11.18284257 | 13.80188224 | 16.66091563 | 46.35516337 | 77.44802821 | 51.69388053 |
| MA1887 | 271.6956711 | 335.3272332 | 404.7896253 | 1958.077986 | 3271.464667 | 2183.589532 |
| MA1886 | 122.4957916 | 151.1845025 | 182.5020817 | 1035.501058 | 1730.066497 | 1154.759558 |
| MA1885 | 58.77631433 | 72.54182144 | 87.56872039 | 139.6817104 | 233.3736366 | 155.7688319 |
| MA1884 | 76.00489212 | 93.80535979 | 113.2369599 | 172.6268368 | 288.4168053 | 192.5082435 |
| MA1883 | 627.7939579 | 774.82431 | 935.3276773 | 1744.440402 | 2914.529033 | 1945.347339 |
| MA1882 | 387.4065129 | 478.1377397 | 577.183054 | 678.528455 | 1133.653451 | 756.6744746 |
| MA1881 | 10.05831526 | 12.41398883 | 14.98552277 | 81.87637934 | 136.7952063 | 91.30606958 |
| MA1879n | 172.8036408 | 213.2745307 | 257.4539401 | 1368.906459 | 2287.104572 | 1526.563209 |
| MA1878 | 3282.922687 | 4051.788447 | 4891.10864 | 4698.297667 | 7849.694916 | 5239.399899 |
| MA1877 | 136.6968484 | 168.7114696 | 203.6597264 | 249.3399024 | 416.5853896 | 278.0563413 |
| MA1876 | 58.17415255 | 71.79863241 | 86.67158119 | 159.7477355 | 266.8990082 | 178.1458581 |
| MA1875 | 49.21649758 | 60.74308027 | 73.32589266 | 516.541021 | 863.0124597 | 576.0309723 |
| MA1874 | 635.4884626 | 784.3208801 | 946.7914437 | 2563.720246 | 4283.343288 | 2858.983519 |
| MA1873 | 968.2221921 | 1194.981383 | 1442.519481 | 3669.485718 | 6130.804267 | 4092.099834 |
| MA1872 | 51.17312066 | 63.15794762 | 76.24099513 | 81.48914251 | 136.1482292 | 90.87423474 |
| MA1871 | 17.45998407 | 21.54914035 | 26.01300338 | 105.5105479 | 176.2820643 | 117.6621817 |
| MA1870 | 631.4159548 | 779.2945844 | 940.7239604 | 1765.51346 | 2949.736907 | 1968.847378 |
| MA1869 | 102.8936092 | 126.9914575 | 153.297494 | 361.3928371 | 603.7981663 | 403.0143955 |
| MA1868 | 664.4772619 | 820.0989027 | 989.9808149 | 1426.379288 | 2383.127473 | 1590.655175 |
| MA1867 | 79.68981965 | 98.35330326 | 118.7270011 | 330.8852555 | 552.8275329 | 368.9932604 |
| MA1866 | 155.7428021 | 192.2180162 | 232.0356089 | 1559.469468 | 2605.48829 | 1739.073331 |
| MA1864n | 512.359432 | 632.354833 | 763.3459218 | 2561.795174 | 4280.126969 | 2856.836737 |
| MA1863 | 10577.87195 | 13055.22653 | 15759.59162 | 85219.3183 | 142380.4316 | 95034.01431 |
| MA1862 | 21255.24462 | 26233.25701 | 31667.4258 | 161403.7254 | 269665.7582 | 179992.568 |
| MA1861 | 2224.925369 | 2746.00646 | 3314.836423 | 18051.13456 | 30158.9872 | 20130.081 |
| MA1860 | 1265.717744 | 1562.15087 | 1885.747422 | 1854.488858 | 3098.39282 | 2068.070059 |
| MA1859 | 96.93570588 | 119.6382037 | 144.4210278 | 317.2413318 | 530.0319065 | 353.7779681 |
| MA1858 | 223.656765 | 276.0375382 | 333.218184 | 625.6449315 | 1045.298145 | 697.7003637 |
| MA1857 | 175.7699507 | 216.9355552 | 261.8733387 | 426.9056809 | 713.2539466 | 476.0723437 |
| MA1856 | 717.5318107 | 885.578912 | 1069.024882 | 3059.180378 | 5111.13479 | 3411.50572 |
| MA1855 | 174.3601538 | 215.1955815 | 259.7729329 | 800.9468659 | 1338.184378 | 893.1917954 |
| MA1854 | 317.2936849 | 391.6043749 | 472.7244689 | 1702.702705 | 2844.79565 | 1898.802718 |
| MA1853 | 544.1753695 | 671.6221141 | 810.7473448 | 1566.788611 | 2617.716771 | 1747.235419 |
| MA1852 | 161.9401142 | 199.8667488 | 241.2687618 | 630.6478011 | 1053.656705 | 703.2794131 |
| MA1851 | 106.7564462 | 131.7589772 | 159.0525962 | 306.4963804 | 512.0797467 | 341.7955222 |
| MA1850 | 144.5722032 | 178.4312451 | 215.3929347 | 450.4417225 | 752.5768587 | 502.3190276 |
| MA1849 | 56.27068497 | 69.44936966 | 83.83567317 | 219.8919536 | 367.3851409 | 245.2168767 |
| MA1848 | 43.96291186 | 54.25909635 | 65.49876392 | 233.3069371 | 389.7982648 | 260.1768618 |
| MA1847 | 29.00466434 | 35.79760326 | 43.21300801 | 88.49183661 | 147.8480014 | 98.68342805 |
| MA1844 | 1730.892992 | 2136.270908 | 2578.795323 | 7933.751732 | 13255.3395 | 8847.48072 |
| MA4669 | 440.7025353 | 543.9157761 | 656.5868842 | 852.4780342 | 1424.280232 | 950.6578006 |
| MA1842 | 316.4270924 | 390.5348251 | 471.4333639 | 431.3025009 | 720.5999468 | 480.9755448 |
| MA1841 | 571.5216919 | 705.372989 | 851.4896486 | 1692.868319 | 2828.36482 | 1887.835707 |
| MA1840 | 202.1805725 | 249.531587 | 301.2215758 | 468.3939555 | 782.5706057 | 522.3388165 |
| MA1839 | 1346.169925 | 1661.445081 | 2005.610238 | 5309.25841 | 8870.459409 | 5920.724898 |
| MA1838 | 637.5784222 | 786.9003116 | 949.9051995 | 1249.282623 | 2087.242689 | 1393.162314 |
| MA1837 | 109.9820713 | 135.7400488 | 163.8583392 | 194.7430174 | 325.3674804 | 217.1715413 |
| MA1836 | 131.4099223 | 162.186337 | 195.7829251 | 419.6520616 | 701.1349403 | 467.9833261 |
| MA1835 | 145.7949764 | 179.9403938 | 217.2147007 | 854.4727958 | 1427.612986 | 952.8822985 |
| MA1834 | 409.8901387 | 505.8870667 | 610.6806008 | 2462.223001 | 4113.766462 | 2745.796851 |
| MA1833 | 117.2278848 | 144.6828435 | 174.6536165 | 734.9156619 | 1227.862546 | 819.5557876 |
| MA1832 | 37.52172709 | 46.30937577 | 55.902274 | 217.483398 | 363.3610394 | 242.5309281 |
| MA1831 | 94.98432188 | 117.2298024 | 141.5137308 | 483.4005729 | 807.6429567 | 539.0737438 |
| MA1828 | 108.1915752 | 133.5302157 | 161.1907433 | 979.1080091 | 1635.847642 | 1091.87173 |
| MA1827 | 1805.99766 | 2228.96521 | 2690.691071 | 4305.100145 | 7192.758977 | 4800.917878 |
| MA1826 | 340.2964355 | 419.9944066 | 506.9954411 | 1002.604821 | 1675.105012 | 1118.074667 |
| MA1825 | 562.778282 | 694.5818585 | 838.4631561 | 638.1607538 | 1066.208993 | 711.6576314 |
| MA1824 | 8594.359205 | 10607.17192 | 12804.42721 | 715.0898981 | 1194.738592 | 797.4466935 |
| MA1823 | 2.219196101 | 2.738935388 | 3.306300592 | 40.7135943 | 68.02235976 | 45.4025728 |
| MA1822 | 398.3538012 | 491.6489006 | 593.4930259 | 7072.644751 | 11816.64243 | 7887.200178 |
| MA1821 | 4107.012486 | 5068.8814 | 6118.890444 | 74549.30877 | 124553.4812 | 83135.1414 |
| MA1820 | 143.9844707 | 177.7058647 | 214.5172932 | 2282.160878 | 3812.927131 | 2544.997001 |
| MA1819 | 1727.315932 | 2131.856095 | 2573.465989 | 16238.77603 | 27130.98375 | 18108.99341 |
| MA1818 | 609.2350631 | 751.9188923 | 907.6774464 | 5800.372603 | 9690.98992 | 6468.400639 |
| MA1817 | 809.2341878 | 998.7581329 | 1205.648961 | 7136.471107 | 11923.2805 | 7958.377405 |
| MA1816 | 2987.878092 | 3687.643934 | 4451.532291 | 27565.43252 | 46055.02905 | 30740.13922 |
| MA1815 | 339.5198519 | 419.035946 | 505.8384372 | 2742.890395 | 4582.692352 | 3058.788667 |
| MA1813 | 912.6714895 | 1126.420617 | 1359.756483 | 2070.913829 | 3459.985489 | 2309.420661 |
| MA1812 | 121.1918601 | 149.5751881 | 180.559401 | 219.8895544 | 367.3811324 | 245.2142011 |
| MA1811 | 1535.921599 | 1895.636902 | 2288.314444 | 3344.369504 | 5587.615378 | 3729.540034 |
| MA1810 | 2739.002382 | 3380.481134 | 4080.741307 | 8949.961134 | 14953.1744 | 9980.726805 |
| MA1809a | 35.11066993 | 43.33364515 | 52.3101265 | 115.4933089 | 192.9607922 | 128.7946559 |
| MA1808 | 79.67985358 | 98.34100312 | 118.712153 | 230.7342821 | 385.4999937 | 257.3079145 |
| MA1807 | 30.95856928 | 38.20911588 | 46.12406083 | 113.0431244 | 188.867139 | 126.0622839 |
| MA1806 | 627.9322702 | 774.9950153 | 935.5337439 | 4689.965641 | 7835.774158 | 5230.108275 |
| MA1805 | 229.8369782 | 283.6651673 | 342.4258617 | 1843.090453 | 3079.348901 | 2055.358902 |
| MA1804 | 266.2393826 | 328.5930731 | 396.6604969 | 3585.826317 | 5991.030072 | 3998.805392 |
| MA1803a | 1078.952417 | 1331.644805 | 1607.492468 | 1580.300588 | 2640.291946 | 1762.303568 |
| MA1802 | 1985.468378 | 2450.468257 | 2958.078049 | 12739.97519 | 21285.35176 | 14207.23621 |
| MA1801 | 163.5679479 | 201.8758237 | 243.6940127 | 541.624196 | 904.9202496 | 604.0029728 |
| MA1800 | 1464.145626 | 1807.050881 | 2181.377999 | 2293.590622 | 3832.023409 | 2557.743107 |
| MA1799 | 867.0049231 | 1070.058867 | 1291.719506 | 1442.689706 | 2410.378153 | 1608.844062 |
| MA1798 | 65.90678252 | 81.34225673 | 98.19214894 | 133.7619726 | 223.4832168 | 149.1673188 |
| MA1797 | 160.4057924 | 197.9730864 | 238.9828309 | 408.0551895 | 681.7594315 | 455.050844 |
| MA1796 | 849.4578437 | 1048.402233 | 1265.576742 | 2982.220317 | 4982.553538 | 3325.682181 |
| MA1795 | 63.74100383 | 78.66924919 | 94.96543303 | 488.0343212 | 815.3848057 | 544.2411603 |
| MA1794 | 1503.116964 | 1855.149368 | 2239.439996 | 1618.480522 | 2704.081186 | 1804.88068 |
| MA1793 | 76.71703113 | 94.6842829 | 114.2979502 | 225.5511587 | 376.8402748 | 251.5278516 |
| MA1792 | 129.9257918 | 160.3546207 | 193.5717723 | 530.2486458 | 885.914515 | 591.3173022 |
| MA1791 | 363.6357292 | 448.7998003 | 541.7678168 | 1687.167711 | 2818.840512 | 1881.478561 |
| MA1790 | 1463.657756 | 1806.448751 | 2180.651139 | 7976.683693 | 13327.0682 | 8895.357148 |
| MA1789 | 3987.930039 | 4921.909652 | 5941.473781 | 9513.41285 | 15894.56305 | 10609.07117 |
| MA1788 | 78.02811625 | 96.30242626 | 116.2512889 | 375.6560362 | 627.6284489 | 418.9202851 |
| MA1787b | 141.5313967 | 174.6782769 | 210.8625464 | 343.8726783 | 574.5263084 | 383.4764427 |
| MA1787 | 6.538124017 | 8.069363152 | 9.740916227 | 35.32358084 | 59.01697862 | 39.39179231 |
| MA1786 | 1338.866942 | 1652.431727 | 1994.729785 | 1261.108125 | 2107.000181 | 1406.349757 |
| MA1785 | 87.70094293 | 108.2406445 | 130.662486 | 193.422575 | 323.1613472 | 215.6990238 |
| MA1784 | 16317.35447 | 20138.90506 | 24310.64056 | 56995.10051 | 95224.73509 | 63559.21762 |
| MA1783 | 243.0353276 | 299.9545913 | 362.0896086 | 4542.234105 | 7588.951253 | 5065.362521 |
| MA1782 | 209.8949582 | 259.0526942 | 312.7149621 | 4165.378904 | 6959.319295 | 4645.104963 |
| MA1781 | 118.3906282 | 146.1179033 | 176.3859462 | 2694.811032 | 4502.363613 | 3005.172011 |
| MA1780 | 606.027085 | 747.9596006 | 902.897995 | 1309.824549 | 2188.393293 | 1460.676844 |
| MA1779 | 4710.776527 | 5814.047947 | 7018.41681 | 16979.26589 | 28368.15941 | 18934.76538 |
| MA1778 | 5037.300979 | 6217.044949 | 7504.893867 | 24957.95985 | 41698.58626 | 27832.36431 |
| MA1777 | 2786.875285 | 3439.565948 | 4152.065427 | 20645.93221 | 34494.25315 | 23023.7211 |
| MA1776a | 6018.995874 | 7428.654364 | 8967.485845 | 51585.26778 | 86186.24083 | 57526.33527 |
| MA1776 | 1978.218935 | 2441.520983 | 2947.277364 | 15175.39081 | 25354.3297 | 16923.13828 |
| MA1775 | 1169.373296 | 1443.242398 | 1742.207288 | 12228.84941 | 20431.38682 | 13637.24414 |
| MA4672 | 219.4018692 | 270.786139 | 326.8789675 | 2655.973167 | 4437.475133 | 2961.861195 |
| MA1774 | 531.4313675 | 655.8934464 | 791.7605138 | 817.9507588 | 1366.593683 | 912.1540241 |
| MA1773 | 4623.2023 | 5705.963695 | 6887.943113 | 1279.17206 | 2137.180555 | 1426.494113 |
| MA1772 | 1833.305837 | 2262.669006 | 2731.376543 | 1614.382913 | 2697.235092 | 1800.311149 |
| MA1771 | 1086.232235 | 1340.629567 | 1618.338406 | 1633.36209 | 2728.944609 | 1821.476155 |
| MA1770 | 419.2757669 | 517.4708242 | 624.6639114 | 873.5149967 | 1459.4278 | 974.1175868 |
| MA1769 | 110.8175939 | 136.7712521 | 165.1031543 | 259.3692346 | 433.3419265 | 289.2407502 |
| MA1768 | 941.761505 | 1162.32356 | 1403.096653 | 4833.023835 | 8074.789065 | 5389.642459 |
| MA1767 | 3238.962115 | 3997.532239 | 4825.613363 | 11297.33216 | 18875.05158 | 12598.44419 |
| MA1766 | 2801.795948 | 3457.981054 | 4174.295186 | 9140.243886 | 15271.08988 | 10192.92439 |
| MA1765 | 178.0320962 | 219.7274988 | 265.2436281 | 2035.092351 | 3400.136648 | 2269.473629 |
| MA1764 | 1102.761198 | 1361.02964 | 1642.964315 | 4163.097208 | 6955.507144 | 4642.560484 |
| MA1763 | 27.18259095 | 33.54879736 | 40.49836628 | 235.1084898 | 392.8082143 | 262.185899 |
| MA1762 | 2660.195918 | 3283.21807 | 3963.330385 | 6433.972624 | 10749.57906 | 7174.972279 |
| MA1761 | 546.623039 | 674.6430317 | 814.3940396 | 1726.66924 | 2884.83781 | 1925.529475 |
| MA1760 | 475.4448587 | 586.7948074 | 708.3482245 | 3167.313248 | 5291.798107 | 3532.092236 |
| MA1759 | 163.2682192 | 201.5058981 | 243.2474577 | 942.374865 | 1574.475632 | 1050.908036 |
| MA1758 | 1302.109385 | 1607.065492 | 1939.966021 | 3832.535399 | 6403.220011 | 4273.927922 |
| MA1757 | 7.684056354 | 9.48367468 | 11.44819967 | 50.14469551 | 83.7794004 | 55.91985251 |
| MA1756 | 1026.432352 | 1266.824455 | 1529.244707 | 5493.12284 | 9177.651457 | 6125.764967 |
| MA1755a | 109.0380788 | 134.5749718 | 162.4519187 | 324.8320834 | 542.7141775 | 362.2429454 |
| MA1754 | 593.9129456 | 733.0083103 | 884.8495736 | 1884.97973 | 3149.335536 | 2102.07256 |
| MA1753 | 2279.972882 | 2813.946189 | 3396.849736 | 3217.436439 | 5375.541579 | 3587.988108 |
| MA1752 | 183.2446185 | 226.1608022 | 273.0095778 | 671.6866512 | 1122.222487 | 749.0447014 |
| MA1751 | 62.42313913 | 77.04273846 | 93.00199373 | 1147.713936 | 1917.5465 | 1279.895976 |
| MA1750 | 529.574666 | 653.6019025 | 788.9942809 | 3279.690553 | 5479.552826 | 3657.412018 |
| MA1749 | 489.8971694 | 604.6318724 | 729.8802033 | 1984.705475 | 3315.952624 | 2213.283705 |
| MA1748 | 150.5223649 | 185.7749442 | 224.2578671 | 486.3263565 | 812.5312185 | 542.3364895 |
| MA1747 | 157.5746395 | 194.4788729 | 234.7647978 | 751.5971774 | 1255.733238 | 838.1585107 |
| MA1746 | 111.6508982 | 137.7997176 | 166.3446644 | 688.4299062 | 1150.19633 | 767.7162745 |
| MA1745 | 37.52981529 | 46.31935825 | 55.91432432 | 585.1150827 | 977.582779 | 652.5026983 |
| MA1744 | 80.47523678 | 99.32266635 | 119.8971659 | 1713.766315 | 2863.28021 | 1911.140522 |
| MA1743 | 80.58257441 | 99.45514261 | 120.0570843 | 796.4445206 | 1330.662071 | 888.1709157 |
| MA1742 | 220.4126454 | 272.0336406 | 328.3848866 | 3433.657249 | 5736.793145 | 3829.111035 |
| MA1741 | 865.8298596 | 1068.608602 | 1289.968821 | 3163.290784 | 5285.077563 | 3527.606505 |
| MA1740 | 16.41254487 | 20.25638921 | 24.45246132 | 159.2643126 | 266.0913279 | 177.6067595 |
| MA1739 | 261.1025632 | 322.2532024 | 389.0073342 | 1124.993826 | 1879.586809 | 1254.559195 |
| MA1738 | 705.9068593 | 871.2313782 | 1051.705285 | 2119.563717 | 3541.267436 | 2363.67355 |
| MA1737 | 984.6628158 | 1215.272427 | 1467.013776 | 1096.266555 | 1831.590634 | 1222.523408 |
| MA1736 | 207.2866161 | 255.8334742 | 308.8288869 | 396.9032729 | 663.1273333 | 442.6145628 |
| MA1735 | 4970.09814 | 6134.103098 | 7404.770771 | 2103.964889 | 3515.205648 | 2346.278208 |
| MA1734 | 101.964296 | 125.8444978 | 151.9129437 | 498.3215197 | 832.5721734 | 555.7131338 |
| MA1733 | 89.04074665 | 109.8942324 | 132.6586115 | 397.4029372 | 663.9621491 | 443.1717734 |
| MA1732 | 287.3556409 | 354.654793 | 428.1208521 | 690.2933762 | 1153.309728 | 769.79436 |
| MA1731 | 178.7522492 | 220.6163127 | 266.3165581 | 625.8012553 | 1045.559324 | 697.8746913 |
| MA1730 | 170.0202186 | 209.8392265 | 253.3070192 | 372.3921026 | 622.1752222 | 415.2804448 |
| MA1729 | 51.6711813 | 63.77265486 | 76.98303781 | 219.3391842 | 366.4615998 | 244.6004449 |
| MA1728 | 44.70562841 | 55.17575831 | 66.60531065 | 341.8431145 | 571.1354087 | 381.2131344 |
| MA1727 | 680.5760095 | 839.9680029 | 1013.965761 | 2711.554878 | 4530.338443 | 3023.844243 |
| MA1726 | 194.6447944 | 240.2309175 | 289.9942906 | 938.1048405 | 1567.341475 | 1046.146233 |
| MA1725 | 762.327328 | 940.8656113 | 1135.764115 | 3718.322082 | 6212.397769 | 4146.560675 |
| MA1724 | 1275.52094 | 1574.24999 | 1900.352852 | 5996.44924 | 10018.58555 | 6687.059393 |
| MA1723 | 397.8267306 | 490.9983892 | 592.7077623 | 2961.879677 | 4948.569352 | 3302.998912 |
| MA1722 | 481.4506748 | 594.2071952 | 717.2960742 | 636.1725824 | 1062.88725 | 709.4404826 |
| MA1721 | 331.4973569 | 409.1345697 | 493.8860098 | 882.757979 | 1474.870541 | 984.425082 |
| MA1719 | 98.42835442 | 121.4804329 | 146.6448712 | 577.2333082 | 964.4142807 | 643.7131811 |
| MA1718 | 85.03362855 | 104.9486408 | 126.6885501 | 438.5280461 | 732.6720481 | 489.0332549 |
| MA1717 | 282.5485984 | 348.7219334 | 420.959012 | 734.7864958 | 1227.646741 | 819.4117454 |
| MA1716 | 186.0129947 | 229.5775365 | 277.1340822 | 405.2998628 | 677.1559611 | 451.9781867 |
| MA1715 | 88.36911022 | 109.0652976 | 131.6579645 | 298.1569431 | 498.1466067 | 332.4956331 |
| MA1714 | 34056.79843 | 42032.95527 | 50740.0012 | 8895.554749 | 14862.27477 | 9920.054444 |
| MA1713 | 235.405923 | 290.5383678 | 350.7228325 | 112.6864676 | 188.2712537 | 125.6645511 |
| MA1712 | 404.1220473 | 498.7680791 | 602.0869285 | 6716.308369 | 11221.29235 | 7489.824589 |
| MA1711 | 725.2008496 | 895.0440521 | 1080.450708 | 1022.136762 | 1707.73806 | 1139.856098 |
| MA1710 | 2146.041729 | 2648.648145 | 3197.310519 | 15442.48467 | 25800.57756 | 17220.99329 |
| MA1709 | 123.8003045 | 152.7945345 | 184.4456288 | 1383.374846 | 2311.277672 | 1542.697918 |
| MA1708 | 184.3145582 | 227.4813235 | 274.6036426 | 1653.333458 | 2762.311833 | 1843.747623 |
| MA1707 | 11.35882982 | 14.01908599 | 16.92311272 | 78.21906391 | 130.6847356 | 87.22754168 |
| MA1706 | 204.2316604 | 252.0630429 | 304.277418 | 1246.109145 | 2081.94059 | 1389.623346 |
| MA1705 | 695.3556275 | 858.209031 | 1035.985383 | 3464.951665 | 5789.078384 | 3864.009624 |
| MA1704 | 413.2030224 | 509.9758331 | 615.6163472 | 759.7877938 | 1269.417735 | 847.2924392 |
| MA1703a | 11.55630644 | 14.26281195 | 17.21732604 | 117.100399 | 195.6458428 | 130.5868343 |
| MA1703 | 90.71912362 | 111.9656879 | 135.1591651 | 728.5934424 | 1217.299679 | 812.5054391 |
| MA1702 | 54.2627958 | 66.97122963 | 80.84419119 | 487.5172223 | 814.520861 | 543.6645071 |
| MA1701 | 119.3079711 | 147.2500893 | 177.7526624 | 798.3624519 | 1333.866461 | 890.3097348 |
| MA1700 | 21.75074564 | 26.84480517 | 32.40565499 | 187.957214 | 314.030079 | 209.6042179 |
| MA1698n | 134.6164017 | 166.1437789 | 200.5601435 | 547.19517 | 914.2279711 | 610.215555 |
| MA1697 | 81.25047734 | 100.2794695 | 121.0521689 | 349.3986883 | 583.7589063 | 389.6388825 |
| MA1696 | 58.92586698 | 72.72639956 | 87.79153352 | 505.3818291 | 844.3682065 | 563.586578 |
| MA1695 | 34.84078469 | 43.00055237 | 51.90803418 | 319.1774707 | 533.2667163 | 355.9370918 |
| MA1694 | 59.42267513 | 73.33956098 | 88.53171014 | 299.2619625 | 499.9928213 | 333.7279175 |
| MA1693 | 144.2864017 | 178.0785085 | 214.9671294 | 901.8912404 | 1506.837494 | 1005.761918 |
| MA1692 | 715.7392647 | 883.3665488 | 1066.354232 | 2755.242341 | 4603.329403 | 3072.563185 |
| MA1691 | 143.3023607 | 176.8640035 | 213.501042 | 1556.922188 | 2601.232415 | 1736.232681 |
| MA1690 | 147.0536419 | 181.4938407 | 219.0899412 | 594.0733543 | 992.5498382 | 662.4926928 |
| MA1689 | 1117.827539 | 1379.624541 | 1665.411115 | 2851.560375 | 4764.253047 | 3179.974152 |
| MA1688 | 198.5078257 | 244.9986769 | 295.7496822 | 366.2643607 | 611.937279 | 408.4469718 |
| MA1687 | 349.2055767 | 430.990083 | 520.2688508 | 824.4106353 | 1377.386541 | 919.3578835 |
| MA1686 | 90.7700836 | 112.0285828 | 135.2350885 | 1330.955315 | 2223.697584 | 1484.241237 |
| MA1685a | 1085.02005 | 1339.133487 | 1616.532416 | 1071.593704 | 1790.368395 | 1195.008989 |
| MA1685 | 456.2089147 | 563.0537745 | 679.6892822 | 1329.272398 | 2220.885845 | 1482.364499 |
| MA1684 | 808.9682914 | 998.4299632 | 1205.252812 | 18624.74797 | 31117.35353 | 20769.75738 |
| MA1683 | 127.6190262 | 157.5076069 | 190.1350051 | 4361.610019 | 7287.173019 | 4863.935986 |
| MA1682 | 1985.655911 | 2450.699711 | 2958.357448 | 52301.96303 | 87383.66157 | 58325.5722 |
| MA1681 | 5.878695192 | 7.255495038 | 8.758456897 | 180.8360805 | 302.1324238 | 201.6629446 |
| MA1680 | 194.6765618 | 240.2701249 | 290.0416197 | 318.5121769 | 532.1551747 | 355.1951762 |
| MA1679 | 58.13753755 | 71.75344212 | 86.61702981 | 160.2247433 | 267.6959704 | 178.6778028 |
| MA1678 | 867.1563008 | 1070.245698 | 1291.945038 | 1143.60598 | 1910.683121 | 1275.314908 |
| MA1677 | 290.325947 | 358.3207496 | 432.5462045 | 993.9595107 | 1660.660833 | 1108.433677 |
| MA1676 | 1070.019102 | 1320.619293 | 1594.183042 | 2322.586813 | 3880.468882 | 2590.078785 |
| MA1675 | 143.3912546 | 176.9737165 | 213.6334818 | 1496.82518 | 2500.825158 | 1669.21431 |
| MA1674 | 2260.81177 | 2790.297514 | 3368.302284 | 2459.053774 | 4108.471466 | 2742.262625 |
| MA1673 | 34.31961995 | 42.35733001 | 51.13156955 | 131.4789394 | 219.6688323 | 146.621349 |
| MA1672 | 6541.444533 | 8073.461338 | 9745.863344 | 22907.94934 | 38273.52506 | 25546.2544 |
| MA1671 | 1231.023507 | 1519.331187 | 1834.057724 | 4397.345804 | 7346.878689 | 4903.787456 |
| MA1670 | 302.4354402 | 373.2663056 | 450.5877037 | 1905.300001 | 3183.28569 | 2124.733114 |
| MA1669 | 879.5279114 | 1085.514759 | 1310.377056 | 2983.362404 | 4984.461682 | 3326.955801 |
| MA1668 | 1342.009372 | 1656.31012 | 1999.41158 | 3366.546059 | 5624.666924 | 3754.27066 |
| MA1667 | 82.57985399 | 101.9201883 | 123.0327595 | 506.1303825 | 845.6188544 | 564.4213422 |
| MA1666 | 1620.519465 | 2000.047724 | 2414.353768 | 885.9287821 | 1480.168169 | 987.9610661 |
| MA1665 | 345.1310742 | 425.9613255 | 514.1983957 | 282.5604606 | 472.0887369 | 315.1029061 |
| MA1664 | 27.6771373 | 34.15916726 | 41.23517313 | 78.18347949 | 130.6252828 | 87.187859 |
| MA1663 | 92.47226238 | 114.1294146 | 137.7711036 | 433.7942829 | 724.763099 | 483.7543049 |
| MA1662 | 77.07226637 | 95.1227148 | 114.8272024 | 748.5014208 | 1250.560993 | 834.7062163 |
| MA1661 | 676.7734271 | 835.2748496 | 1008.30043 | 6525.641018 | 10902.7343 | 7277.198108 |
| MA1660 | 497.7143009 | 614.2797887 | 741.5266668 | 1554.310323 | 2596.868632 | 1733.320008 |
| MA1659 | 748.1332886 | 923.3473051 | 1114.616926 | 1185.907263 | 1981.358116 | 1322.488024 |
| MA1658 | 1174.163508 | 1449.154485 | 1749.344053 | 2081.759314 | 3478.105617 | 2321.51522 |
| MA1657 | 532.9464252 | 657.763333 | 794.0177438 | 1790.09237 | 2990.802195 | 1996.257038 |
| MA1656 | 465.4593551 | 574.4706828 | 693.4711811 | 2094.620471 | 3499.593433 | 2335.857594 |
| MA1655 | 651.2042884 | 803.7173775 | 970.2058882 | 1003.195449 | 1676.091805 | 1118.733317 |
| MA1654 | 754.7214031 | 931.4783665 | 1124.432321 | 520.6544023 | 869.8849039 | 580.6180911 |
| MA1653 | 377.8045454 | 466.2869761 | 562.8774274 | 2594.813 | 4335.291601 | 2893.657221 |
| MA1652 | 826.310242 | 1019.833426 | 1231.089961 | 1995.156621 | 3333.413907 | 2224.938507 |
| MA1651 | 135.0085032 | 166.6277112 | 201.1443215 | 581.7290418 | 971.925541 | 648.7266876 |
| MA1649n | 175.7590723 | 216.922129 | 261.8571313 | 1461.852163 | 2442.393886 | 1630.213456 |
| MA1648 | 80.68438945 | 99.58080291 | 120.2087749 | 213.9508518 | 357.4590272 | 238.591539 |
| MA1647 | 135.6422532 | 167.4098866 | 202.088523 | 397.7049029 | 664.4666591 | 443.5085164 |
| MA1646 | 2196.51786 | 2710.945867 | 3272.513095 | 9426.579733 | 15749.48636 | 10512.2375 |
| MA1645 | 2069.121044 | 2553.712513 | 3082.709154 | 6990.851729 | 11679.98649 | 7795.987066 |
| MA1644 | 7.266116777 | 8.967852984 | 10.8255265 | 31.47605403 | 52.58871165 | 35.10114641 |
| MA1643 | 379.9228574 | 468.9014001 | 566.0334243 | 943.6885273 | 1576.670437 | 1052.372992 |
| MA1642 | 165.0988923 | 203.7653178 | 245.9749119 | 1003.401879 | 1676.436698 | 1118.963522 |
| MA1641 | 1674.468136 | 2066.63126 | 2494.729956 | 8104.958359 | 13541.38348 | 9038.405189 |
| MA1640 | 558.2956612 | 689.0494007 | 831.7846604 | 3196.861777 | 5341.1664 | 3565.043864 |
| MA1639 | 47.30784389 | 58.38741683 | 70.48225806 | 320.598859 | 535.6415051 | 357.5221812 |
| MA1638 | 116.927919 | 144.3126253 | 174.2067084 | 867.1491882 | 1448.792107 | 967.0186291 |
| MA1636 | 955.4483386 | 1179.215872 | 1423.488175 | 6070.280019 | 10141.93855 | 6769.393251 |
| MA1635 | 60.78784851 | 75.02446016 | 90.56563295 | 150.7253504 | 251.8248312 | 168.0843662 |
| MA1634 | 159.4607937 | 196.8067674 | 237.5749112 | 518.2744532 | 865.9085968 | 577.9640436 |
| MA1633 | 377.6026918 | 466.037848 | 562.5766929 | 755.5978566 | 1262.417385 | 842.6199476 |
| MA1632 | 444.8549906 | 549.0407431 | 662.7734782 | 925.6559926 | 1546.542525 | 1032.263653 |
| MA1631 | 670.7249724 | 827.809837 | 999.2890545 | 1558.16366 | 2603.306607 | 1737.617133 |
| MA1630 | 825.1742106 | 1018.431335 | 1229.397429 | 2623.246453 | 4382.796878 | 2925.365351 |
| MA1628 | 545.6823486 | 673.4820301 | 812.9925388 | 3027.480836 | 5058.172685 | 3376.155346 |
| MA1627 | 727.2454755 | 897.5675326 | 1083.496923 | 3654.231266 | 6105.317846 | 4075.088529 |
| MA1626 | 76.44070497 | 94.34324071 | 113.8862618 | 245.1209433 | 409.5365511 | 273.3514853 |
| MA1625 | 18.92602578 | 23.35853138 | 28.19720628 | 56.53828694 | 94.46151245 | 63.0497929 |
| MA1624 | 213.3244958 | 263.2854349 | 317.824507 | 688.6058167 | 1150.490233 | 767.9124445 |
| MA1623 | 77.37802465 | 95.50008217 | 115.2827407 | 242.5134309 | 405.1800419 | 270.4436661 |
| MA1621 | 12382833.88 | 15282913.44 | 18448739.6 | 10744057.58 | 17950666.42 | 11981449.07 |
| MA1619 | 338.5086552 | 417.7879253 | 504.3318914 | 891.3731274 | 1489.264326 | 994.0324357 |
| MA1617 | 11424.6357 | 14100.30371 | 17021.15454 | 5462.517824 | 9126.518035 | 6091.635176 |
| MA1616 | 12184.91275 | 15038.63887 | 18153.86402 | 5116.182792 | 8547.877742 | 5705.412791 |
| MA1615 | 1605.879145 | 1981.978617 | 2392.54168 | 1209.434564 | 2020.666425 | 1348.72496 |
| MA1614 | 49.23806262 | 60.76969588 | 73.35802164 | 211.325388 | 353.0725257 | 235.663701 |
| MA1613 | 8021.436611 | 9900.069935 | 11950.8504 | 20701.83307 | 34587.64968 | 23086.06005 |
| MA1612 | 10.40881411 | 12.84657507 | 15.50771842 | 81.69665561 | 136.4949323 | 91.10564709 |
| MA1611 | 1602.780353 | 1978.154083 | 2387.924901 | 14105.86476 | 23567.41584 | 15730.43507 |
| MA1610 | 818.341836 | 1009.998807 | 1219.218119 | 8458.664004 | 14132.33825 | 9432.847059 |
| MA1609 | 15.89647905 | 19.61945995 | 23.68359339 | 206.7281067 | 345.3916042 | 230.5369515 |
| MA1608 | 54.91673824 | 67.77832644 | 81.81847656 | 237.6875101 | 397.1171203 | 265.0619448 |
| MA1607 | 9.589180871 | 11.83498242 | 14.28657629 | 64.08571876 | 107.0714068 | 71.46646131 |
| MA1606 | 15.56773972 | 19.21372934 | 23.19381647 | 124.8547263 | 208.6014084 | 139.2342263 |
| MA1605 | 1020.008585 | 1258.896232 | 1519.674168 | 6464.502818 | 10800.58747 | 7209.01863 |
| MA1603n | 1587.325067 | 1959.079145 | 2364.898626 | 5631.686522 | 9409.157144 | 6280.287007 |
| MA1602 | 94.50227317 | 116.6348572 | 140.7955438 | 419.5666948 | 700.9923135 | 467.8881276 |
| MA1601 | 245.3478196 | 302.8086726 | 365.5349074 | 393.6929758 | 657.7637199 | 439.0345363 |
| MA1600 | 236.3185066 | 291.66468 | 352.0824582 | 1079.761504 | 1804.01477 | 1204.117473 |
| MA1599 | 63.39010538 | 78.23616976 | 94.44264203 | 212.7703451 | 355.4866921 | 237.2750736 |
| MA1598 | 423.7466893 | 522.9888438 | 631.3249781 | 727.1638839 | 1214.91124 | 810.9112385 |
| MA1597 | 634.9720924 | 783.6835752 | 946.0221222 | 875.8748581 | 1463.370546 | 976.7492331 |
| MA1596 | 503.7785004 | 621.7642334 | 750.5614999 | 1180.299425 | 1971.988804 | 1316.234332 |
| MA1595 | 14.73755839 | 18.18911821 | 21.95695911 | 34.31302232 | 57.32858494 | 38.26484791 |
| MA1594 | 63.99343656 | 78.98080207 | 95.34152349 | 174.9560073 | 292.3082738 | 195.1056642 |
| MA1593 | 1482.592509 | 1829.818054 | 2208.861348 | 2985.79451 | 4988.525131 | 3329.668012 |
| MA1591 | 519.4392926 | 641.0928083 | 773.8939519 | 4103.990744 | 6856.754842 | 4576.646738 |
| MA1590 | 462.4514949 | 570.7583769 | 688.9898782 | 4420.396863 | 7385.391314 | 4929.493301 |
| MA1589 | 92.73153942 | 114.4494147 | 138.1573911 | 1487.65378 | 2485.502015 | 1658.986642 |
| MA1588 | 207.8794843 | 256.5651931 | 309.7121799 | 582.7518347 | 973.6343754 | 649.8672755 |
| MA1587 | 1173.779318 | 1448.680318 | 1748.771662 | 7948.651554 | 13280.23342 | 8864.096552 |
| MA1586 | 567.2814393 | 700.1396625 | 845.1722486 | 4439.575065 | 7417.433353 | 4950.880254 |
| MA1585 | 46.0529219 | 56.83859009 | 68.61259484 | 664.193995 | 1109.704109 | 740.689117 |
| MA1584 | 1770.33995 | 2184.956407 | 2637.565929 | 11091.70542 | 18531.50008 | 12369.13545 |
| MA1583 | 1600.404387 | 1975.221662 | 2384.385034 | 13754.067 | 22979.64869 | 15338.12082 |
| MA1582 | 849.6199218 | 1048.602271 | 1265.818217 | 2469.772187 | 4126.379288 | 2754.215477 |
| MA1581 | 656.9025456 | 810.7501757 | 978.695517 | 5009.982095 | 8370.442608 | 5586.980975 |
| MA1580 | 92.74974557 | 114.4718848 | 138.1845158 | 490.8973121 | 820.1681562 | 547.4338813 |
| MA1579 | 1017.055036 | 1255.250957 | 1515.273781 | 1673.588645 | 2796.153248 | 1866.335597 |
| MA1577 | 183.9202087 | 226.9946167 | 274.0161154 | 872.3664741 | 1457.508903 | 972.8367891 |
| MA1576 | 233.0935187 | 287.6843947 | 347.2776644 | 501.1612101 | 837.3165946 | 558.8798709 |
| MA1575 | 49.11639251 | 60.61953043 | 73.1767497 | 302.339991 | 505.1354467 | 337.1604421 |
| MA1574 | 1103.446563 | 1361.875518 | 1643.985415 | 712.9208887 | 1191.114713 | 795.0278797 |
| MA1573 | 252.5040142 | 311.6408595 | 376.1966648 | 471.9021457 | 788.4319251 | 526.2510445 |
| MA1572 | 110.7244736 | 136.656323 | 164.9644178 | 333.4369609 | 557.0908024 | 371.8388453 |
| MA1571 | 274.0199431 | 338.1958534 | 408.2524747 | 655.8127409 | 1095.701103 | 731.3425951 |
| MA1570 | 222.7590737 | 274.9296061 | 331.8807458 | 164.0610705 | 274.1055256 | 182.9559592 |
| MA1569 | 104.4886803 | 128.9600969 | 155.6739332 | 218.6005958 | 365.227601 | 243.7767934 |
| MA1568 | 6125.397148 | 7559.974987 | 9126.009282 | 7927.844272 | 13245.46959 | 8840.892898 |
| MA1567 | 1169.037507 | 1442.827967 | 1741.707009 | 8950.062376 | 14953.34355 | 9980.839707 |
| MA1566 | 579.4562223 | 715.1657992 | 863.3110206 | 5417.652373 | 9051.558948 | 6041.602578 |
| MA1565 | 414.0885884 | 511.0688002 | 616.9357204 | 1268.777891 | 2119.814466 | 1414.902849 |
| MA1564 | 65.66361335 | 81.04213695 | 97.82985993 | 321.338786 | 536.8777403 | 358.3473254 |
| MA1563 | 559.3037688 | 690.2936088 | 833.2866038 | 1058.65557 | 1768.751967 | 1180.580771 |
| MA1562a | 31.46497345 | 38.83412071 | 46.87853421 | 231.3267371 | 386.4898398 | 257.9686025 |
| MA1562 | 124.171315 | 153.2524361 | 184.9983839 | 1536.012532 | 2566.297544 | 1712.914863 |
| MA1561 | 20.6668437 | 25.50705165 | 30.7907884 | 114.1771683 | 190.7618463 | 127.3269355 |
| MA1560 | 3.168382344 | 3.910422572 | 4.720459096 | 47.04634804 | 78.60282707 | 52.46466884 |
| MA1559 | 52.327823 | 64.5830831 | 77.9613447 | 276.8536388 | 462.5540472 | 308.7388305 |
| MA1558 | 11.54085842 | 14.24374598 | 17.19431059 | 128.127567 | 214.0695168 | 142.8839996 |
| MA1557 | 4.629053009 | 5.713184651 | 6.896659876 | 80.8567315 | 135.0916266 | 90.16898905 |
| MA1556 | 242.8090887 | 299.6753669 | 361.7525434 | 1138.274891 | 1901.776188 | 1269.369839 |
| MA1555 | 300.4341819 | 370.7963493 | 447.6061007 | 1696.910095 | 2835.117629 | 1892.342974 |
| MA1554 | 1710.105467 | 2110.614911 | 2547.824736 | 2885.604196 | 4821.131864 | 3217.938796 |
| MA1553 | 275.2057734 | 339.659407 | 410.0192007 | 1448.000127 | 2419.250554 | 1614.766082 |
| MA1552 | 589.436809 | 727.4838553 | 878.1807384 | 2432.833186 | 4064.663341 | 2713.022216 |
| MA1551 | 209.6351111 | 258.7319905 | 312.3278252 | 998.0874121 | 1667.557537 | 1113.036989 |
| MA1550 | 56.05845878 | 69.18743977 | 83.519485 | 427.7595045 | 714.6804749 | 477.024502 |
| MA1549 | 37.68781221 | 46.5143583 | 56.14971826 | 305.0653507 | 509.6888495 | 340.199681 |
| MA1548 | 331.6895427 | 409.3717657 | 494.1723405 | 1513.07881 | 2527.980959 | 1687.339868 |
| MA1547 | 300.8552168 | 371.3159913 | 448.2333855 | 893.1786375 | 1492.280887 | 996.0458862 |
| MA1546 | 71.47711472 | 88.21716966 | 106.4911869 | 563.4284458 | 941.3497655 | 628.3184148 |
| MA1545 | 31.38845588 | 38.7396826 | 46.76453343 | 110.8019574 | 185.1227026 | 123.5630021 |
| MA1544 | 40.70534172 | 50.23859806 | 60.64542714 | 223.7547851 | 373.8389782 | 249.5245899 |
| MA1543 | 66.92085921 | 82.59383181 | 99.7029854 | 411.0081385 | 686.6930798 | 458.3438836 |
| MA1542 | 45.27430352 | 55.87761805 | 67.45255928 | 105.3924079 | 176.0846815 | 117.5304356 |
| MA1540 | 240.442701 | 296.7547673 | 358.2269473 | 1569.195586 | 2621.738231 | 1749.919605 |
| MA1539 | 59.2953689 | 73.18243943 | 88.34204117 | 260.728251 | 435.6125072 | 290.7562842 |
| MA1538 | 58.77292581 | 72.53763933 | 87.56367196 | 347.377199 | 580.3814969 | 387.3845785 |
| MA1537 | 101.1322439 | 124.8175776 | 150.673299 | 460.4543238 | 769.3054423 | 513.4847787 |
| MA1536 | 74.0188103 | 91.35413443 | 110.2779679 | 451.6717586 | 754.6319451 | 503.6907268 |
| MA1535 | 151.8418951 | 187.4035106 | 226.2237878 | 619.6467041 | 1035.276589 | 691.0113213 |
| MA1534 | 66.83452925 | 82.48728323 | 99.57436548 | 64.85207745 | 108.3518029 | 72.32108141 |
| MA1532 | 514.6944734 | 635.2367448 | 766.8248161 | 1014.690826 | 1695.29774 | 1131.552615 |
| MA1531 | 390.9825742 | 482.5513204 | 582.5108994 | 883.9829238 | 1476.917121 | 985.7911036 |
| MA1530 | 471.7567193 | 582.2428998 | 702.853398 | 1611.257846 | 2692.013877 | 1796.826168 |
| MA1529 | 160.7498377 | 198.3977076 | 239.4954116 | 685.6245691 | 1145.509305 | 764.5878471 |
| MA1528 | 331.0970707 | 408.6405358 | 493.2896378 | 1204.051924 | 2011.673363 | 1342.722403 |
| MA1527 | 105.2648452 | 129.9180409 | 156.8303135 | 553.7054542 | 925.1050481 | 617.4756277 |
| MA1526 | 445.9392036 | 550.3788806 | 664.3888084 | 13645.20554 | 22797.76807 | 15216.7218 |
| MA1525 | 2176.777988 | 2686.582885 | 3243.103368 | 54612.96033 | 91244.76724 | 60902.72671 |
| MA1524 | 754.4067938 | 931.0900753 | 1123.963596 | 22312.74031 | 37279.07778 | 24882.49524 |
| MA1523 | 157.1316036 | 193.9320773 | 234.1047345 | 3296.908346 | 5508.319506 | 3676.612782 |
| MA1522 | 502.3017306 | 619.9416018 | 748.3613136 | 13325.26787 | 22263.23126 | 14859.93696 |
| MA1521 | 1160.595023 | 1432.40824 | 1729.128855 | 38240.6995 | 63890.76336 | 42644.87508 |
| MA1520 | 277.1505803 | 342.0596908 | 412.9166986 | 813.156317 | 1358.583355 | 906.8074071 |
| MA1519 | 189.2192329 | 233.5346809 | 281.9109414 | 658.346264 | 1099.933995 | 734.1679038 |
| MA1518 | 92.63380952 | 114.3287963 | 138.0117869 | 935.4994934 | 1562.988584 | 1043.240829 |
| MA1517 | 2202.880563 | 2718.798725 | 3281.992658 | 3220.194376 | 5380.14941 | 3591.063675 |
| MA1516 | 104.2627085 | 128.6812021 | 155.3372659 | 700.7373419 | 1170.759015 | 781.4411557 |
| MA1515 | 66.45584595 | 82.01991169 | 99.01017883 | 470.8881609 | 786.7378069 | 525.1202792 |
| MA1514 | 42.64828768 | 52.63658507 | 63.54015255 | 530.9837658 | 887.142719 | 592.1370859 |
| MA1513 | 123.8712795 | 152.8821319 | 184.5513719 | 615.343804 | 1028.087506 | 686.2128569 |
| MA1512 | 62.19072375 | 76.75589103 | 92.65572641 | 240.3929293 | 401.6372075 | 268.0789466 |
| MA1511 | 25.67323712 | 31.68595044 | 38.24963421 | 269.7802025 | 450.7360822 | 300.8507476 |
| MA1510 | 916.7453419 | 1131.448572 | 1365.82597 | 5577.116921 | 9317.98482 | 6219.432634 |
| MA1509 | 8.04296834 | 9.926644429 | 11.98292975 | 73.12276326 | 122.170076 | 81.54430085 |
| MA1508 | 344.1368629 | 424.7342683 | 512.717156 | 1614.717414 | 2697.793961 | 1800.684174 |
| MA1507 | 323.1853975 | 398.8759361 | 481.5023199 | 3622.860151 | 6052.904461 | 4040.104407 |
| MA1506 | 753.592213 | 930.0847184 | 1122.749981 | 8467.717483 | 14147.46438 | 9442.943226 |
| MA1505 | 1104.651253 | 1363.362349 | 1645.78024 | 13380.08072 | 22354.80998 | 14921.06259 |
| MA1504 | 1368.428749 | 1688.916958 | 2038.772861 | 20103.79961 | 33588.48348 | 22419.15117 |
| MA1503 | 189.6785609 | 234.1015842 | 282.5952776 | 2852.976062 | 4766.618311 | 3181.552884 |
| MA1502 | 136.4050589 | 168.3513425 | 203.2249997 | 2323.380356 | 3881.794697 | 2590.96372 |
| MA1501 | 69.44878674 | 85.71380401 | 103.4692538 | 1247.519755 | 2084.297372 | 1391.196417 |
| MA1500 | 203.0452444 | 250.5987664 | 302.5098194 | 3523.209781 | 5886.413306 | 3928.977319 |
| MA1499 | 791.1130675 | 976.3930172 | 1178.650955 | 8393.41676 | 14023.32622 | 9360.0853 |
| MA1498 | 2289.089414 | 2825.197827 | 3410.43213 | 30169.92897 | 50406.49931 | 33644.59513 |
| MA1497 | 804.8000897 | 993.2855619 | 1199.042758 | 11636.53881 | 19441.78212 | 12976.71723 |
| MA1496 | 705.2746676 | 870.4511263 | 1050.763405 | 9336.878073 | 15599.61704 | 10412.20491 |
| MA1495 | 572.408376 | 706.467336 | 852.8106875 | 8732.7873 | 14590.33057 | 9738.541094 |
| MA1494 | 202.9360867 | 250.4640438 | 302.3471893 | 2470.298375 | 4127.258418 | 2754.802266 |
| MA1493 | 170.3616664 | 210.260642 | 253.8157301 | 1573.134298 | 2628.318845 | 1754.311937 |
| MA1492 | 1067.180332 | 1317.115678 | 1589.95366 | 5653.599981 | 9445.769122 | 6304.724236 |
| MA1491 | 820.0187495 | 1012.068456 | 1221.716493 | 6728.537648 | 11241.72445 | 7503.462312 |
| MA1490 | 1270.852492 | 1568.488184 | 1893.397498 | 10515.17615 | 17568.26209 | 11726.2074 |
| MA1489 | 892.2406967 | 1101.204899 | 1329.317379 | 11000.95147 | 18379.87265 | 12267.92939 |
| MA1488 | 117.7972335 | 145.3855345 | 175.5018687 | 1600.89236 | 2674.695712 | 1785.266892 |
| MA1487 | 139.0824485 | 171.6557811 | 207.2139464 | 1748.72819 | 2921.692867 | 1950.128951 |
| MA1486 | 278.8154994 | 344.1145367 | 415.3972019 | 3121.415611 | 5215.114492 | 3480.908575 |
| MA1485 | 290.7554514 | 358.8508446 | 433.1861076 | 2372.522212 | 3963.898601 | 2645.765237 |
| MA1484 | 2999.728384 | 3702.269584 | 4469.187616 | 12739.33759 | 21284.28648 | 14206.52517 |
| MA1483 | 457.5455702 | 564.7034768 | 681.6807172 | 1889.945041 | 3157.631345 | 2107.609726 |
| MA1482 | 1306.634719 | 1612.650666 | 1946.708153 | 4219.764428 | 7050.184073 | 4705.75406 |
| MA1481 | 749.7178123 | 925.3029268 | 1116.977651 | 1373.75211 | 2295.200458 | 1531.966933 |
| MA1480 | 224.7848129 | 277.4297767 | 334.8988219 | 2402.640991 | 4014.21964 | 2679.352791 |
| MA1479 | 2951.858038 | 3643.187927 | 4397.867307 | 50572.17626 | 84493.61513 | 56396.56615 |
| MA1478 | 13595.43233 | 16779.50439 | 20255.34649 | 321707.5316 | 537493.8232 | 358758.5394 |
| MA1477 | 2414.147334 | 2979.544512 | 3596.751434 | 63114.33329 | 105448.4616 | 70383.20152 |
| MA1476 | 543.9742998 | 671.3739535 | 810.4477782 | 3465.611233 | 5790.180361 | 3864.745155 |
| MA1475 | 213.7171607 | 263.7700626 | 318.4095244 | 3357.629785 | 5609.770032 | 3744.3275 |
| MA1474 | 183.4895159 | 226.4630549 | 273.3744416 | 3441.124301 | 5749.268745 | 3837.438067 |
| MA1473 | 36.44013194 | 44.97446931 | 54.29084423 | 993.0481208 | 1659.138126 | 1107.417323 |
| MA1472 | 319.3212464 | 394.1067947 | 475.7452601 | 8312.816115 | 13888.66245 | 9270.201891 |
| MA1471 | 192.1309647 | 237.1283448 | 286.2490259 | 4671.176657 | 7804.382407 | 5209.155366 |
| MA1470 | 3502.830562 | 4323.1991 | 5218.741488 | 4612.957715 | 7707.112935 | 5144.231357 |
| MA1469 | 1217.473525 | 1502.607778 | 1813.870093 | 2883.654083 | 4817.873707 | 3215.764088 |
| MA1468 | 129.8913457 | 160.3121072 | 193.5204522 | 727.9197861 | 1216.174165 | 811.7541978 |
| MA1467 | 96.35422742 | 118.9205421 | 143.5547039 | 134.633795 | 224.9398167 | 150.1395488 |
| MA1466 | 715.9879307 | 883.6734528 | 1066.724711 | 2953.39179 | 4934.38819 | 3293.533476 |
| MA1465 | 62746.89097 | 77442.3135 | 93484.33997 | 39535.69204 | 66054.37603 | 44089.01172 |
| MA1464 | 363.8128295 | 449.0183779 | 542.0316723 | 2540.720395 | 4244.916219 | 2833.334779 |
| MA1463 | 177.1370862 | 218.622876 | 263.9101848 | 1302.538577 | 2176.220234 | 1452.551748 |
| MA1462 | 2471.987868 | 3050.931393 | 3682.92597 | 3221.803068 | 5382.837137 | 3592.85764 |
| MA1461 | 334.1261846 | 412.3790731 | 497.8026059 | 1044.662994 | 1745.37383 | 1164.976673 |
| MA1460 | 3237.073227 | 3995.200971 | 4822.799177 | 8012.684708 | 13387.217 | 8935.504395 |
| MA1459 | 257.7567256 | 318.1237642 | 384.0224909 | 1086.082868 | 1814.576209 | 1211.166868 |
| MA1458 | 553.5414194 | 683.1817079 | 824.7014862 | 4721.916339 | 7889.155883 | 5265.73873 |
| MA1457 | 1535.349194 | 1894.930439 | 2287.461639 | 8999.687291 | 15036.25453 | 10036.17992 |
| MA1456 | 2467.651711 | 3045.5797 | 3676.465684 | 13302.42787 | 22225.07126 | 14834.46648 |
| MA1455 | 1447.89191 | 1786.990518 | 2157.162171 | 4663.697267 | 7791.886194 | 5200.814577 |
| MA1454 | 1154.541406 | 1424.936855 | 1720.109787 | 1151.233303 | 1923.426494 | 1283.820669 |
| MA1453 | 290.2506771 | 358.2278514 | 432.4340626 | 691.5835287 | 1155.465254 | 771.2330991 |
| MA1453a | 3565.111097 | 4400.065835 | 5311.53102 | 11984.16731 | 20022.58347 | 13364.38204 |
| MA1452 | 12.12104375 | 14.95981164 | 18.05870788 | 57.47024625 | 96.01858627 | 64.08908583 |
| MA1451 | 32.99714532 | 40.72512969 | 49.16126207 | 141.5853661 | 236.554175 | 157.8917313 |
| MA1450 | 449.5876999 | 554.8818605 | 669.824572 | 1864.808649 | 3115.634642 | 2079.578379 |
| MA1449 | 385.141782 | 475.3426052 | 573.8089127 | 2186.981016 | 3653.90509 | 2438.855289 |
| MA1448 | 125.511293 | 154.9062391 | 186.994769 | 993.4092686 | 1659.741514 | 1107.820064 |
| MA1447 | 1203.588399 | 1485.470733 | 1793.183142 | 8434.492424 | 14091.95351 | 9405.891642 |
| MA1446 | 364.9712665 | 450.4481226 | 543.7575858 | 4922.257689 | 8223.876795 | 5489.153363 |
| MA1445 | 236.2549809 | 291.5862764 | 351.9878134 | 3619.571133 | 6047.409325 | 4036.436594 |
| MA1444 | 3016.631614 | 3723.131577 | 4494.371131 | 8720.278964 | 14569.43223 | 9724.592174 |
| MA1443 | 3504.734835 | 4325.549356 | 5221.578596 | 7720.27306 | 12898.66937 | 8609.415741 |
| MA1442 | 3396.40094 | 4191.843489 | 5060.175815 | 7184.348648 | 12003.27205 | 8011.768996 |
| MA1441 | 255.9839889 | 315.9358497 | 381.3813541 | 621.0996487 | 1037.704101 | 692.6316013 |
| MA1440 | 123.4322083 | 152.3402295 | 183.8972154 | 434.6560677 | 726.2029284 | 484.7153413 |
| MA1439 | 2121.972537 | 2618.94191 | 3161.450696 | 2716.193872 | 4538.089057 | 3029.01751 |
| MA1438b | 240.3808821 | 296.6784703 | 358.1348455 | 779.0409025 | 1301.584924 | 868.7629254 |
| MA1438a | 29.72751483 | 36.6897465 | 44.289957 | 125.224301 | 209.2188764 | 139.6463648 |
| MA1437 | 291.17631 | 359.370269 | 433.8131298 | 1162.895279 | 1942.910775 | 1296.825753 |
| MA1436 | 336.0957978 | 414.8099729 | 500.7370619 | 1562.8269 | 2611.097729 | 1742.817438 |
| MA1435 | 867.1694961 | 1070.261983 | 1291.964697 | 4919.863807 | 8219.877209 | 5486.483778 |
| MA1433 | 893.8034156 | 1103.13361 | 1331.645618 | 598.8737646 | 1000.570138 | 667.8459656 |
| MA1432a | 17435.99692 | 21519.53538 | 25977.26579 | 3410.325663 | 5697.811829 | 3803.092354 |
| MA1432 | 5495.187593 | 6782.169343 | 8187.082693 | 2648.941612 | 4425.727142 | 2954.019817 |
| MA1431 | 1932.979315 | 2385.686172 | 2879.87648 | 1532.93129 | 2561.149549 | 1709.478755 |
| MA1430 | 663.5137734 | 818.9097636 | 988.5453479 | 1578.589697 | 2637.433471 | 1760.395634 |
| MA1429 | 188.6522197 | 232.8348722 | 281.0661687 | 572.3391015 | 956.2372702 | 638.2553094 |
| MA1428 | 305.071988 | 376.520337 | 454.5158015 | 1022.721087 | 1708.714322 | 1140.50772 |
| MA1427 | 43.59927608 | 53.81029648 | 64.95699602 | 252.297437 | 421.5266995 | 281.3544947 |
| MA1426 | 1847.052985 | 2279.635758 | 2751.857925 | 1045.592699 | 1746.927138 | 1166.013452 |
| MA1425 | 709.9030253 | 876.1634528 | 1057.659029 | 3306.599006 | 5524.510204 | 3687.419513 |
| MA1424 | 1224.949566 | 1511.83472 | 1825.008377 | 8355.873973 | 13960.6015 | 9318.218715 |
| MA1423 | 791.5305468 | 976.9082708 | 1179.272942 | 8627.459905 | 14414.35451 | 9621.083158 |
| MA1422 | 2019.372803 | 2492.313153 | 3008.591035 | 20291.52452 | 33902.12544 | 22628.49633 |
| MA1421 | 284.5421272 | 351.1823499 | 423.9290991 | 363.0764171 | 606.6110126 | 404.8918732 |
| MA1420 | 995.4959169 | 1228.642658 | 1483.153624 | 1249.358224 | 2087.368998 | 1393.246621 |
| MA1419 | 256.8048011 | 316.9488975 | 382.6042528 | 416.980394 | 696.6712437 | 465.0039629 |
| MA1418 | 1746.816148 | 2155.923292 | 2602.518661 | 5289.621352 | 8837.650736 | 5898.826244 |
| MA1417 | 723.9389617 | 893.486628 | 1078.570666 | 1981.329926 | 3310.312916 | 2209.519395 |
| MA1416 | 773.9770415 | 955.2437064 | 1153.120605 | 977.001058 | 1632.327447 | 1089.522122 |
| MA1415 | 115.712403 | 142.8124334 | 172.3957547 | 271.8206641 | 454.1451897 | 303.1262089 |
| MA1414 | 2274.334488 | 2806.987274 | 3388.449295 | 15658.16435 | 26160.92504 | 17461.51277 |
| MA1413 | 827.3250497 | 1021.085904 | 1232.601887 | 6387.729326 | 10672.31793 | 7123.403147 |
| MA1412 | 540.0176666 | 666.4906704 | 804.5529324 | 1921.230191 | 3209.901103 | 2142.497981 |
| MA1411 | 2392.106246 | 2952.341367 | 3563.913209 | 10257.76341 | 17138.18898 | 11439.14847 |
| MA1410 | 83.38582062 | 102.9149136 | 124.2335402 | 390.779868 | 652.8966363 | 435.7859263 |
| MA1409 | 590.9687134 | 729.3745342 | 880.4630677 | 1383.865973 | 2312.098224 | 1543.245608 |
| MA1408 | 3611.547025 | 4457.377132 | 5380.714242 | 8107.678989 | 13545.92898 | 9041.439152 |
| MA1407 | 1530.181501 | 1888.552464 | 2279.762479 | 2118.950543 | 3540.242974 | 2362.989757 |
| MA1406 | 35.89797536 | 44.30533878 | 53.4831046 | 269.6020181 | 450.4383803 | 300.6520418 |
| MA1405 | 38.02332517 | 46.92844892 | 56.64958696 | 93.92758506 | 156.9297944 | 104.7452108 |
| MA1404 | 264.859076 | 326.8894965 | 394.6040278 | 487.7748168 | 814.9512378 | 543.9517687 |
| MA1403 | 156.1073251 | 192.6679111 | 232.5786988 | 233.1030825 | 389.4576741 | 259.9495293 |
| MA1402 | 13.92457027 | 17.18572696 | 20.74571729 | 58.2103283 | 97.25508057 | 64.91440302 |
| MA1401 | 19.44686686 | 24.00135428 | 28.97318871 | 50.1225738 | 83.74244047 | 55.89518304 |
| MA1400 | 722.2902369 | 891.4517692 | 1076.114291 | 1720.991376 | 2875.351503 | 1919.197693 |
| MA1399 | 41.79745351 | 51.58648418 | 62.27252528 | 82.95781344 | 138.6020156 | 92.51205228 |
| MA1398 | 86.79473203 | 107.1221975 | 129.312355 | 213.938994 | 357.4392157 | 238.5783156 |
| MA1397 | 115.6017547 | 142.6758711 | 172.2309037 | 897.9920995 | 1500.322993 | 1001.413714 |
| MA1396 | 552.6777967 | 682.1158234 | 823.4148058 | 2533.910375 | 4233.538358 | 2825.74045 |
| MA1395 | 231.3000814 | 285.4709315 | 344.6056866 | 570.083657 | 952.4689795 | 635.7401056 |
| MA1394 | 3033.876996 | 3744.415856 | 4520.064407 | 828.8083959 | 1384.734113 | 924.2621336 |
| MA1393 | 3155.931569 | 3895.055806 | 4701.909133 | 9170.277922 | 15321.26934 | 10226.41744 |
| MA1392 | 192.5067147 | 237.5920961 | 286.8088424 | 1549.262654 | 2588.435224 | 1727.690999 |
| MA1391 | 9238.027636 | 11401.58853 | 13763.40569 | 12048.68891 | 20130.38313 | 13436.33459 |
| MA1390 | 698.1386238 | 861.6438095 | 1040.131669 | 10951.80786 | 18297.76583 | 12213.12592 |
| MA1389 | 217.4180638 | 268.3377232 | 323.9233671 | 819.8473441 | 1369.762409 | 914.2690388 |
| MA1388 | 105.6695293 | 130.4175026 | 157.4332378 | 268.9240288 | 449.3056276 | 299.8959686 |
| MA1386n | 131.7000237 | 162.5443805 | 196.2151367 | 504.8223246 | 843.4334127 | 562.9626354 |
| MA1385 | 1942.541108 | 2397.487352 | 2894.122252 | 6998.675298 | 11693.05774 | 7804.711674 |
| MA1384 | 1525.266728 | 1882.486643 | 2272.440135 | 9452.661573 | 15793.06267 | 10541.32318 |
| MA1383 | 640.737478 | 790.7992233 | 954.6117632 | 4747.341173 | 7931.6345 | 5294.091738 |
| MA1382 | 1315.276316 | 1623.316139 | 1959.582959 | 7681.736696 | 12834.28462 | 8566.44115 |
| MA1381 | 2015.777346 | 2487.875634 | 3003.234292 | 9266.159302 | 15481.46344 | 10333.34147 |
| MA1380 | 1052.937149 | 1299.536719 | 1568.733254 | 5586.637795 | 9333.891849 | 6230.050026 |
| MA1378 | 2046.466485 | 2525.752219 | 3048.956939 | 5138.635785 | 8585.391147 | 5730.451692 |
| MA1377 | 1325.730703 | 1636.21896 | 1975.158575 | 2940.208848 | 4912.36275 | 3278.832257 |
| MA1376 | 812.1447259 | 1002.350324 | 1209.985268 | 2226.70342 | 3720.271416 | 2483.152516 |
| MA1375 | 23.15629365 | 28.57953477 | 34.4997305 | 168.3057393 | 281.1973186 | 187.6894858 |
| MA1374 | 286.9056727 | 354.0994415 | 427.4504606 | 2872.364422 | 4799.011471 | 3203.174198 |
| MA1373 | 311.9804357 | 385.0467542 | 464.8084497 | 3292.42983 | 5500.837012 | 3671.618476 |
| MA1372 | 492.0613623 | 607.3029228 | 733.1045566 | 2157.43816 | 3604.546275 | 2405.909987 |
| MA1371 | 90.98752227 | 112.296946 | 135.5590426 | 871.7671774 | 1456.507626 | 972.1684715 |
| MA1370 | 442.423987 | 546.0403946 | 659.1516134 | 2727.817506 | 4557.509278 | 3041.979836 |
| MA4683 | 242.3233419 | 299.0758573 | 361.0288466 | 582.223413 | 972.7515132 | 649.2779955 |
| MA1368 | 2365.879563 | 2919.972352 | 3524.839015 | 7072.263749 | 11816.00587 | 7886.775296 |
| MA1367 | 1930.823331 | 2383.025253 | 2876.664356 | 1993.927477 | 3331.360311 | 2223.567803 |
| MA1366 | 503.7179398 | 621.6894894 | 750.4712729 | 782.1149407 | 1306.720882 | 872.1910002 |
| MA1365 | 34.11424726 | 42.1038587 | 50.82559215 | 129.0444257 | 215.6013612 | 143.9064527 |
| MA1364 | 28086.17043 | 34663.9966 | 41844.5769 | 2579.443243 | 4309.612533 | 2876.517331 |
| MA1363 | 347.877016 | 429.3503713 | 518.2894759 | 2011.006612 | 3359.895328 | 2242.61394 |
| MA1362 | 859.7610648 | 1061.118486 | 1280.927141 | 8013.667001 | 13388.85817 | 8936.599819 |
| MA1361 | 228.7081588 | 282.2719766 | 340.7440742 | 1209.143823 | 2020.180668 | 1348.400734 |
| MA1360 | 207.6782076 | 256.3167771 | 309.412305 | 1094.350717 | 1828.389743 | 1220.386924 |
| MA1358 | 132.6907406 | 163.7671249 | 197.6911702 | 176.6491496 | 295.1370964 | 196.9938054 |
| MA1357 | 39.07504031 | 48.22647744 | 58.2164996 | 250.5717156 | 418.6434453 | 279.4300222 |
| MA1356 | 767.8435627 | 947.673757 | 1143.982555 | 5554.031312 | 9279.41447 | 6193.688259 |
| MA1355 | 432.2542709 | 533.4889146 | 644.0001184 | 905.651524 | 1513.119999 | 1009.955273 |
| MA1354 | 1486.980243 | 1835.233401 | 2215.398475 | 2971.940924 | 4965.37921 | 3314.21891 |
| MA1353 | 452.6525803 | 558.6645408 | 674.3908272 | 863.7709792 | 1443.147954 | 963.2513524 |
| MA1352 | 1455.909792 | 1796.886201 | 2169.107726 | 6399.673379 | 10692.27349 | 7136.722795 |
| MA1351 | 196.7169897 | 242.7884243 | 293.08158 | 4003.737847 | 6689.257014 | 4464.84773 |
| MA1350 | 1601.745521 | 1976.876891 | 2386.383142 | 8170.541175 | 13650.95617 | 9111.541167 |
| MA1349a | 84.33013913 | 104.080393 | 125.6404465 | 467.6468865 | 781.322438 | 521.5057078 |
| MA1349 | 337.5148298 | 416.5613445 | 502.8512267 | 1681.944846 | 2810.114396 | 1875.65418 |
| MA1348 | 39.70729242 | 49.00680401 | 59.15846932 | 82.31491782 | 137.5278958 | 91.79511446 |
| MA1347 | 38.81584617 | 47.90657962 | 57.83033555 | 222.8923664 | 372.3980896 | 248.5628466 |
| MA1346 | 180.9169253 | 223.2879595 | 269.5416314 | 289.4590169 | 483.6145204 | 322.7959681 |
| MA1345 | 217.4028775 | 268.3189802 | 323.9007415 | 186.8894692 | 312.246141 | 208.4135011 |
| MA1344 | 102.5151113 | 126.5243148 | 152.7335836 | 516.8681801 | 863.5590619 | 576.3958103 |
| MA1343 | 357.2607187 | 440.9317522 | 532.2699176 | 852.4482517 | 1424.230473 | 950.624588 |
| MA1342 | 7499.004969 | 9255.283963 | 11172.49826 | 12919.77153 | 21585.74702 | 14407.73966 |
| MA1341 | 111.5424087 | 137.6658198 | 166.1830299 | 2109.33455 | 3524.177025 | 2352.266292 |
| MA1340 | 179.4731863 | 221.5060945 | 267.3906564 | 4679.184037 | 7817.760762 | 5218.084955 |
| MA1339 | 258.9315451 | 319.5737283 | 385.7728122 | 1996.207888 | 3335.170315 | 2226.110849 |
| MA1338 | 518.0107026 | 639.3296402 | 771.765547 | 440.6090347 | 736.148866 | 491.3539106 |
| MA1337 | 259.2135023 | 319.9217204 | 386.1928902 | 310.6674094 | 519.0485059 | 346.446928 |
| MA1336 | 18.26191949 | 22.53889033 | 27.20777817 | 33.5148953 | 55.99511183 | 37.37480073 |
| MA1334 | 295.3680032 | 364.5436634 | 440.058183 | 535.6484611 | 894.9362727 | 597.3390134 |
| MA1333a | 40.79052452 | 50.3437308 | 60.77233792 | 64.68392503 | 108.0708617 | 72.1335629 |
| MA1332 | 65.37953594 | 80.69152815 | 97.40662319 | 205.4160919 | 343.1995516 | 229.0738322 |
| MA1331 | 64.16251417 | 79.18947793 | 95.59342616 | 116.3698773 | 194.4253214 | 129.7721784 |
| MA1330 | 17.50299236 | 21.60222125 | 26.07707989 | 41.85096868 | 69.92263142 | 46.67093842 |
| MA1329 | 21.51016807 | 26.54788394 | 32.0472271 | 68.17266537 | 113.8996851 | 76.02410094 |
| MA1328a | 26.62554321 | 32.86128814 | 39.66844085 | 53.5973751 | 89.54797517 | 59.77017669 |
| MA1328 | 271.112891 | 334.6079651 | 403.9213622 | 991.5339513 | 1656.608322 | 1105.728767 |
| MA1327 | 101.0077267 | 124.6638984 | 150.4877854 | 425.6843669 | 711.2134325 | 474.7103711 |
| MA1326 | 747.1888279 | 922.1816502 | 1113.209807 | 1732.239569 | 2894.144455 | 1931.741339 |
| MA1325 | 1481.455865 | 1828.415205 | 2207.167902 | 4135.669867 | 6909.682832 | 4611.97434 |
| MA1324 | 87.30021125 | 107.7460608 | 130.0654503 | 404.8341007 | 676.3777878 | 451.4587829 |
| MA1323a | 26.69785403 | 32.95053427 | 39.77617415 | 167.2467736 | 279.4280485 | 186.5085592 |
| MA1323 | 7.918355602 | 9.772847188 | 11.79727371 | 40.39281465 | 67.48641621 | 45.04484901 |
| MA1322 | 2809.049622 | 3466.93355 | 4185.102175 | 12330.03977 | 20600.45091 | 13750.08858 |
| MA1321 | 480.5703433 | 593.1206887 | 715.9844999 | 1764.304549 | 2947.717116 | 1967.499237 |
| MA1320 | 125.210365 | 154.5348333 | 186.5464272 | 364.2290542 | 608.5367845 | 406.1772593 |
| MA1319 | 392.676384 | 484.6418231 | 585.0344457 | 899.9055426 | 1503.519884 | 1003.547528 |
| MA1318 | 1054.312114 | 1301.233703 | 1570.781765 | 6398.045708 | 10689.55405 | 7134.907665 |
| MA1317 | 1007.218863 | 1243.111137 | 1500.61922 | 6515.757386 | 10886.22119 | 7266.176179 |
| MA1316a | 1432.337557 | 1767.793311 | 2133.988299 | 2780.758782 | 4645.961075 | 3101.018351 |
| MA1316 | 2274.284599 | 2806.925701 | 3388.374968 | 5300.117024 | 8855.186411 | 5910.5307 |
| MA1314n | 260.9467496 | 322.060897 | 388.7751931 | 1399.027685 | 2337.429701 | 1560.153492 |
| MA1313 | 896.8841064 | 1106.935803 | 1336.235428 | 3713.015616 | 6203.531976 | 4140.643064 |
| MA1312 | 521.3934541 | 643.5046375 | 776.8053869 | 2955.467585 | 4937.856331 | 3295.84834 |
| MA1310 | 792.3154786 | 977.8770349 | 1180.442384 | 11348.10362 | 18959.87815 | 12655.063 |
| MA1309 | 2236.889009 | 2760.772004 | 3332.66062 | 1359.362427 | 2271.158851 | 1515.919992 |
| MA1308 | 163.5595775 | 201.865493 | 243.6815421 | 1135.465564 | 1897.082497 | 1266.236962 |
| MA1307 | 660.1565085 | 814.7662219 | 983.5434796 | 4317.474144 | 7213.432873 | 4814.716988 |
| MA1306 | 140.3535876 | 173.224623 | 209.1077709 | 941.6170054 | 1573.209435 | 1050.062894 |
| MA1305 | 65.57022336 | 80.92687487 | 97.69072153 | 212.0857074 | 354.3428317 | 236.5115863 |
| MA1304 | 18.65128575 | 23.01944679 | 27.78788098 | 141.3343394 | 236.1347714 | 157.6117939 |
| MA1303 | 4817.062531 | 5945.226304 | 7176.768512 | 6136.196072 | 10252.06799 | 6842.900846 |
| MA1302 | 1709.327422 | 2109.654646 | 2546.665554 | 3492.843117 | 5835.678112 | 3895.113331 |
| MA1301 | 73.77448006 | 91.05258165 | 109.913949 | 232.233979 | 388.0056168 | 258.9803312 |
| MA1300 | 74.57490038 | 92.04046169 | 111.1064665 | 185.0334067 | 309.1451191 | 206.3436761 |
| MA1299 | 269.7844239 | 332.9683688 | 401.9421267 | 802.1135664 | 1340.133646 | 894.4928646 |
| MA1298 | 117.9926765 | 145.6267507 | 175.7930523 | 242.9376127 | 405.8887449 | 270.9167008 |
| MA1297 | 50.8513649 | 62.76083614 | 75.76162278 | 98.36742854 | 164.3476762 | 109.6963904 |
| MA1296 | 409.8331517 | 505.8167333 | 610.5956979 | 766.4376223 | 1280.527956 | 854.7081274 |
| MA1295 | 1518.546389 | 1874.192389 | 2262.427742 | 3900.15617 | 6516.197617 | 4349.336567 |
| MA1294a | 44.04300229 | 54.35794409 | 65.61808778 | 66.96859911 | 111.8879878 | 74.68136255 |
| MA1293 | 196.6636807 | 242.7226303 | 293.0021569 | 433.7513437 | 724.6913582 | 483.7064204 |
| MA1292 | 1528.719702 | 1886.748308 | 2277.584596 | 6149.921019 | 10274.99898 | 6858.206493 |
| MA1291 | 200.7540705 | 247.7709959 | 299.0962817 | 520.4764309 | 869.5875577 | 580.4196228 |
| MA1290 | 561.6837336 | 693.2309652 | 836.8324279 | 2494.891802 | 4168.347959 | 2782.228113 |
| MA1289 | 684.4993299 | 844.8101711 | 1019.810975 | 2308.252063 | 3856.519056 | 2574.093104 |
| MA1288 | 416.7691499 | 514.3771534 | 620.9293926 | 1879.121827 | 3139.548427 | 2095.540004 |
| MA1287 | 29.82980037 | 36.81598747 | 44.44234855 | 89.43596004 | 149.4253984 | 99.73628605 |
| MA1286 | 80.34261934 | 99.15898969 | 119.6995839 | 745.2953439 | 1245.204431 | 831.1308961 |
| MA1285 | 111.5537323 | 137.6797954 | 166.1999005 | 1342.690117 | 2243.30354 | 1497.327535 |
| MA1284 | 207.800277 | 256.4674353 | 309.5941718 | 2997.971202 | 5008.869376 | 3343.247092 |
| MA1283 | 88.88189161 | 109.6981732 | 132.4219391 | 356.8491366 | 596.2067651 | 397.9473977 |
| MA1282 | 62.25936811 | 76.84061201 | 92.75799717 | 864.85883 | 1444.965484 | 964.4644908 |
| MA1281 | 268.2024998 | 331.0159556 | 399.5852749 | 1000.38465 | 1671.395654 | 1115.598799 |
| MA1280 | 740.034658 | 913.3519622 | 1102.551067 | 4902.085853 | 8190.174639 | 5466.658339 |
| MA1279 | 303.6295121 | 374.740031 | 452.3667085 | 3159.445808 | 5278.653558 | 3523.318704 |
| MA1278 | 486.4656205 | 600.3966493 | 724.7676618 | 5756.549516 | 9617.772367 | 6419.530454 |
| MA1277 | 123.4465915 | 152.3579813 | 183.9186444 | 1492.39387 | 2493.421534 | 1664.272647 |
| MA1276 | 1744.465794 | 2153.022481 | 2599.016952 | 8792.744817 | 14690.50477 | 9805.403909 |
| MA1275 | 2373.892463 | 2929.861886 | 3536.777147 | 13824.55181 | 23097.41139 | 15416.72335 |
| MA1274 | 2988.406534 | 3688.296139 | 4452.319599 | 5853.717187 | 9780.115543 | 6527.88891 |
| MA1273 | 103.8542074 | 128.1770294 | 154.7286548 | 623.0514017 | 1040.964998 | 694.8081374 |
| MA1272 | 153.0315834 | 188.8718257 | 227.9962616 | 1021.172957 | 1706.12778 | 1138.781292 |
| MA1271 | 343.3652237 | 423.78191 | 511.5675185 | 3089.878232 | 5162.423322 | 3445.739041 |
| MA1270 | 6469.25013 | 7984.358889 | 9638.303495 | 12331.0414 | 20602.1244 | 13751.20558 |
| MA1269 | 152.754327 | 188.5296354 | 227.5831872 | 984.4016342 | 1644.691982 | 1097.775022 |
| MA1268 | 108.1092271 | 133.4285816 | 161.0680559 | 1128.302086 | 1885.114094 | 1258.248467 |
| MA1267 | 377.6438851 | 466.0886888 | 562.6380653 | 4247.238868 | 7096.087074 | 4736.392726 |
| MA1266 | 1032.114995 | 1273.837983 | 1537.711074 | 4938.756292 | 8251.441884 | 5507.552107 |
| MA1265 | 4206.114681 | 5191.193488 | 6266.539246 | 41468.45013 | 69283.5374 | 46244.36526 |
| MA1264 | 3475.876566 | 4289.932434 | 5178.583697 | 31794.03736 | 53119.98326 | 35455.75184 |
| MA1263 | 3813.00472 | 4706.016543 | 5680.858829 | 31485.8638 | 52605.10136 | 35112.08597 |
| MA1262 | 2494.840372 | 3079.13599 | 3716.973095 | 26688.49149 | 44589.87719 | 29762.20102 |
| MA1261 | 644.5991347 | 795.5652862 | 960.3651069 | 11954.32349 | 19972.72182 | 13331.10112 |
| MA1260 | 788.6181277 | 973.3137584 | 1174.933834 | 12724.80432 | 21260.00497 | 14190.31811 |
| MA1259 | 1784.857491 | 2202.873978 | 2659.195091 | 33253.40866 | 55558.23224 | 37083.19871 |
| MA1258 | 2325.79138 | 2870.495452 | 3465.113071 | 39377.83722 | 65790.63961 | 43912.97678 |
| MA1257 | 6006.047682 | 7412.673685 | 8948.194798 | 129679.0643 | 216661.6856 | 144614.1825 |
| MA1256 | 9743.996517 | 12026.05613 | 14517.23056 | 194759.0069 | 325394.1948 | 217189.3723 |
| MA1255 | 1083.817491 | 1337.649286 | 1614.740766 | 21505.83228 | 35930.93376 | 23982.65573 |
| MA1254 | 50.14691636 | 61.89140463 | 74.7120902 | 222.7286535 | 372.1245658 | 248.3802789 |
| MA1253a | 85.00810762 | 104.9171428 | 126.6505274 | 286.9865662 | 479.483666 | 320.0387656 |
| MA1252 | 80.81043679 | 99.73637073 | 120.3965683 | 283.8667171 | 474.2711689 | 316.559604 |
| MA1251 | 62.71940234 | 77.408387 | 93.44338566 | 1403.231309 | 2344.452918 | 1564.841247 |
| MA1250 | 596.68787 | 736.4331264 | 888.9838338 | 6115.896525 | 10218.15246 | 6820.263404 |
| MA1249 | 187.1354433 | 230.9628644 | 278.806378 | 2265.153946 | 3784.512748 | 2526.031384 |
| MA1248 | 34.58213408 | 42.68132537 | 51.52267992 | 566.8446724 | 947.0574363 | 632.1280877 |
| MA1247 | 35.55259494 | 43.87906971 | 52.96853471 | 497.3725185 | 830.9866268 | 554.6548363 |
| MA1246 | 44.74368348 | 55.22272594 | 66.66200753 | 271.375486 | 453.401407 | 302.6297597 |
| MA1245 | 10.18132247 | 12.56580453 | 15.16878679 | 190.453174 | 318.2002116 | 212.3876373 |
| MA1244 | 49.96750495 | 61.66997478 | 74.44479158 | 379.8048977 | 634.5601718 | 423.5469704 |
| MA1243 | 541.5765215 | 668.4146118 | 806.8754145 | 2753.583129 | 4600.55727 | 3070.712882 |
| MA1241 | 105.1186158 | 129.7375642 | 156.6124514 | 344.2340106 | 575.1300056 | 383.8793896 |
| MA1239 | 93.55576997 | 115.4666814 | 139.3853826 | 685.2850463 | 1144.942046 | 764.2092215 |
| MA1238 | 2193.127537 | 2706.761525 | 3267.461975 | 2871.547256 | 4797.646189 | 3202.262919 |
| MA1237 | 1349.47015 | 1665.518224 | 2010.527125 | 3348.563277 | 5594.622136 | 3734.216803 |
| MA1236 | 715.6911261 | 883.3071361 | 1066.282512 | 1693.34054 | 2829.153785 | 1888.362314 |
| MA1235 | 422.2548894 | 521.147662 | 629.102399 | 1613.218822 | 2695.290185 | 1799.01299 |
| MA1234 | 21.62004278 | 26.68349149 | 32.21092549 | 158.6461334 | 265.0585031 | 176.9173846 |
| MA1233 | 28.16939445 | 34.76671182 | 41.96856939 | 210.1471234 | 351.1039367 | 234.3497359 |
| MA1232 | 96.13025609 | 118.6441163 | 143.2210171 | 444.7826661 | 743.1219732 | 496.0082186 |
| MA1231 | 123.6975302 | 152.6676902 | 184.292509 | 775.3680255 | 1295.448454 | 864.6670438 |
| MA1230 | 222.2764453 | 274.3339454 | 331.1616952 | 1446.787785 | 2417.225031 | 1613.414116 |
| MA1229 | 140.5934353 | 173.5206434 | 209.4651113 | 831.5850498 | 1389.373216 | 927.3585743 |
| MA1228 | 111.8759854 | 138.0775207 | 166.6800139 | 856.5270025 | 1431.045058 | 955.1730879 |
| MA1227 | 143.2548524 | 176.8053686 | 213.4302611 | 1317.651335 | 2201.46992 | 1469.405041 |
| MA1226 | 106.1430565 | 131.0019306 | 158.1387288 | 995.4540635 | 1663.157862 | 1110.100358 |
| MA1225 | 175.6145998 | 216.7438208 | 261.6418869 | 784.49577 | 1310.69866 | 874.8460293 |
| MA1224 | 199.9344266 | 246.7593901 | 297.8751237 | 1254.637956 | 2096.190127 | 1399.134419 |
| MA1223 | 37.14087453 | 45.83932694 | 55.33485545 | 262.4922547 | 438.5597217 | 292.723448 |
| MA1222 | 152.5630167 | 188.29352 | 227.2981609 | 703.6734114 | 1175.664462 | 784.7153718 |
| MA1221 | 111.4122695 | 137.5052018 | 165.9891401 | 685.0374409 | 1144.528359 | 763.9330995 |
| MA1220 | 222.6815345 | 274.8339072 | 331.7652231 | 975.9457527 | 1630.564293 | 1088.345278 |
| MA1219 | 361.3443336 | 445.9717563 | 538.3539489 | 1524.182855 | 2546.533076 | 1699.722764 |
| MA1218 | 137.5240398 | 169.7323906 | 204.8921293 | 624.9375886 | 1044.11635 | 696.9115562 |
| MA1217 | 39.71940941 | 49.02175882 | 59.17652198 | 178.6153809 | 298.4221833 | 199.1864874 |
| MA1216 | 320.6129001 | 395.7009557 | 477.6696485 | 1338.103893 | 2235.641092 | 1492.213116 |
| MA1215 | 46.73793692 | 57.68403673 | 69.63317413 | 314.0079321 | 524.6296942 | 350.1721782 |
| MA1214 | 16.02873123 | 19.7826858 | 23.88063116 | 130.6165781 | 218.2280395 | 145.6596696 |
| MA1213 | 22.69824942 | 28.01421585 | 33.81730684 | 147.7354857 | 246.8295058 | 164.7501593 |
| MA1212 | 16.44375296 | 20.29490628 | 24.49895713 | 237.4563542 | 396.7309159 | 264.8041667 |
| MA1211 | 50.08082717 | 61.80983724 | 74.61362628 | 737.378372 | 1231.977126 | 822.3021277 |
| MA1210 | 290.7519227 | 358.8464894 | 433.1808503 | 3468.249559 | 5794.588352 | 3867.687337 |
| MA1209 | 69.83924175 | 86.19570422 | 104.0509788 | 1112.222419 | 1858.248943 | 1240.316907 |
| MA1208 | 419.4358215 | 517.6683638 | 624.9023709 | 4206.215616 | 7027.547354 | 4690.644833 |
| MA1207 | 224.7390768 | 277.3733291 | 334.8306814 | 1307.957723 | 2185.274289 | 1458.595017 |
| MA1206 | 38.53512254 | 47.56011006 | 57.41209549 | 174.2751968 | 291.1708076 | 194.3464448 |
| MA1205 | 16.98857836 | 20.96733067 | 25.31067294 | 552.5619993 | 923.1946174 | 616.2004813 |
| MA1204 | 98.55388032 | 121.6353572 | 146.8318878 | 811.6108173 | 1356.001207 | 905.0839125 |
| MA1203 | 33.43314608 | 41.26324253 | 49.81084395 | 214.4047107 | 358.2173134 | 239.0976688 |
| MA1202 | 36.85657444 | 45.48844331 | 54.91128695 | 92.8665082 | 155.1569971 | 103.5619299 |
| MA1200 | 45.17285409 | 55.75240899 | 67.30141341 | 291.5281323 | 487.0715013 | 325.1033832 |
| MA1199 | 23.14299838 | 28.56312573 | 34.47992236 | 124.022156 | 207.2103891 | 138.305769 |
| MA1198 | 18.08507055 | 22.32062308 | 26.94429727 | 108.479263 | 181.242054 | 120.9728033 |
| MA1197 | 140.9221888 | 173.9263915 | 209.9549093 | 807.3114628 | 1348.818047 | 900.289402 |
| MA1196 | 644.3905313 | 795.3078276 | 960.0543162 | 1021.430703 | 1706.55841 | 1139.068723 |
| MA1195 | 136.0369484 | 167.89702 | 202.6765651 | 744.3873837 | 1243.687454 | 830.1183662 |
| MA1194 | 306.7803862 | 378.6288448 | 457.0610826 | 1132.94454 | 1892.870487 | 1263.425591 |
| MA1193 | 682.6582437 | 842.5378996 | 1017.068007 | 1809.317619 | 3022.922837 | 2017.696455 |
| MA1192 | 1918.4699 | 2367.778629 | 2858.259423 | 2713.494725 | 4533.579449 | 3026.007503 |
| MA1191 | 146.8840803 | 181.2845675 | 218.8373174 | 505.5051417 | 844.5742314 | 563.7240925 |
| MA1190 | 1128.751712 | 1393.107174 | 1681.686649 | 7390.343507 | 12347.43858 | 8241.488252 |
| MA1189a | 348.8445802 | 430.5445405 | 519.731015 | 682.8156189 | 1140.816243 | 761.4553905 |
| MA1189 | 754.9522823 | 931.763318 | 1124.776299 | 4658.704537 | 7783.544575 | 5195.246834 |
| MA1188 | 11.49977706 | 14.1930433 | 17.13310496 | 53.41418405 | 89.24190818 | 59.56588756 |
| MA1187 | 869.0175451 | 1072.542848 | 1294.718039 | 4741.610472 | 7922.059915 | 5287.701033 |
| MA1186 | 172.4743323 | 212.8680976 | 256.9633151 | 1409.473556 | 2354.88217 | 1571.802412 |
| MA1185 | 265.0550215 | 327.1313328 | 394.8959599 | 5306.957445 | 8866.615066 | 5918.158932 |
| MA1184 | 162.4113842 | 200.4483909 | 241.97089 | 4003.791037 | 6689.345881 | 4464.907045 |
| MA1183 | 183.7426863 | 226.7755182 | 273.7516312 | 2591.770236 | 4330.207895 | 2890.264023 |
| MA1182 | 187.7943351 | 231.7760697 | 279.7880373 | 1712.312899 | 2860.851912 | 1909.519717 |
| MA1181 | 1134.793468 | 1400.563919 | 1690.688044 | 5964.387728 | 9965.018683 | 6651.305362 |
| MA1180 | 215.8016462 | 266.3427379 | 321.5151244 | 2390.779654 | 3994.402276 | 2666.125385 |
| MA1179 | 338.429378 | 417.6900813 | 504.2137792 | 4192.628236 | 7004.846197 | 4675.492596 |
| MA1178 | 561.6754166 | 693.2207003 | 836.8200366 | 5140.6907 | 8588.824403 | 5732.743271 |
| MA1177 | 245.0951258 | 302.4967975 | 365.158428 | 2357.370002 | 3938.583001 | 2628.867949 |
| MA1176 | 181.3148965 | 223.7791362 | 270.1345543 | 1821.49095 | 3043.261468 | 2031.271788 |
| MA1175 | 435.0279402 | 536.9121817 | 648.1325087 | 4691.29074 | 7837.988073 | 5231.585986 |
| MA1174 | 1897.480519 | 2341.873501 | 2826.988098 | 22126.92473 | 36968.62585 | 24675.27931 |
| MA1173 | 951.0322695 | 1173.765553 | 1416.908832 | 13639.73609 | 22788.62997 | 15210.62243 |
| MA1172 | 2025.188566 | 2499.490978 | 3017.255732 | 14165.19653 | 23666.54458 | 15796.60007 |
| MA1171 | 4686.937356 | 5784.625605 | 6982.899686 | 12697.44793 | 21214.29921 | 14159.81109 |
| MA1170 | 3027.357418 | 3736.369382 | 4510.351122 | 1783.857365 | 2980.385042 | 1989.303948 |
| MA1169 | 935.5911146 | 1154.708055 | 1393.90361 | 1370.96082 | 2290.536902 | 1528.854171 |
| MA1168 | 936.4282646 | 1155.741266 | 1395.15085 | 1604.508478 | 2680.737351 | 1789.299477 |
| MA1167 | 898.8822022 | 1109.401856 | 1339.212319 | 5389.106447 | 9003.865756 | 6009.769022 |
| MA1166 | 2059.931087 | 2542.370253 | 3069.017366 | 8593.996304 | 14358.4451 | 9583.765559 |
| MA1165 | 389.287788 | 480.4596125 | 579.9858982 | 2920.16699 | 4878.877755 | 3256.482181 |
| MA1164 | 3976.484865 | 4907.784 | 5924.422028 | 16569.20477 | 27683.04855 | 18477.47758 |
| MA1163 | 1829.836027 | 2258.38656 | 2726.206997 | 8679.31493 | 14500.99145 | 9678.910319 |
| MA1162 | 89.21477658 | 110.1090204 | 132.9178924 | 458.3941638 | 765.8634238 | 511.1873504 |
| MA1161 | 78.63961837 | 97.05714315 | 117.1623439 | 249.4405208 | 416.7534981 | 278.1685479 |
| MA1160 | 9.223529715 | 11.38369517 | 13.74180576 | 85.48776012 | 142.8289316 | 95.33337253 |
| MA1158 | 66.94267566 | 82.6207577 | 99.73548894 | 417.7344973 | 697.931164 | 465.8449162 |
| MA1157 | 796.6566511 | 983.2349169 | 1186.910141 | 1732.100897 | 2893.912768 | 1931.586696 |
| MA1156 | 69.44696011 | 85.71154958 | 103.4665324 | 181.7944711 | 303.7336577 | 202.7317129 |
| MA1155 | 161.1038602 | 198.8346427 | 240.022857 | 611.1188195 | 1021.0286 | 681.5012816 |
| MA1154 | 2.136941528 | 2.637416663 | 3.183752457 | 42.59008994 | 71.15752048 | 47.49518416 |
| MA1153 | 92.80518577 | 114.5403092 | 138.2671142 | 1220.323569 | 2038.859263 | 1360.86805 |
| MA1152 | 1588.107775 | 1960.045163 | 2366.064754 | 11899.61062 | 19881.31012 | 13270.08697 |
| MA1151 | 235.3366797 | 290.4529076 | 350.6196695 | 603.8542245 | 1008.891256 | 673.4000243 |
| MA1150 | 98.56881348 | 121.6537877 | 146.8541362 | 803.6041984 | 1342.624124 | 896.1551725 |
| MA1149 | 241.5015269 | 298.0615719 | 359.8044539 | 1537.776749 | 2569.245115 | 1714.882265 |
| MA1148 | 161.0829675 | 198.8088569 | 239.9917297 | 609.5451513 | 1018.399389 | 679.7463743 |
| MA1147 | 602.5035844 | 743.6108905 | 897.648458 | 1506.417597 | 2516.851717 | 1679.911484 |
| MA1146 | 843.5341118 | 1041.091154 | 1256.751187 | 2671.423767 | 4463.28927 | 2979.091238 |
| MA1145 | 1253.701195 | 1547.320027 | 1867.844398 | 3982.370779 | 6653.55792 | 4441.019819 |
| MA1144 | 151.9173391 | 187.4966236 | 226.336189 | 1660.874198 | 2774.910547 | 1852.156828 |
| MA1143 | 307.0556403 | 378.9685638 | 457.4711738 | 3453.272551 | 5769.56547 | 3850.985429 |
| MA1142 | 1052.219415 | 1298.650891 | 1567.663928 | 12490.72701 | 20868.91961 | 13929.28215 |
| MA1141 | 1193.368471 | 1472.857281 | 1777.956837 | 15685.36677 | 26206.37357 | 17491.84809 |
| MA1140 | 1062.705016 | 1311.592236 | 1583.286048 | 3046.230569 | 5089.498859 | 3397.064484 |
| MA1139 | 548.294607 | 676.7060836 | 816.8844488 | 1865.299402 | 3116.454569 | 2080.125652 |
| MA1138 | 249.3730708 | 307.7766441 | 371.5319847 | 1120.398012 | 1871.908338 | 1249.434082 |
| MA1137 | 442.7344165 | 546.4235272 | 659.6141111 | 4335.624052 | 7243.756886 | 4834.957218 |
| MA1136 | 356.6736909 | 440.2072415 | 531.3953259 | 2461.201353 | 4112.059541 | 2744.657541 |
| MA1134 | 834.6201256 | 1030.089498 | 1243.470559 | 1949.734328 | 3257.524475 | 2174.284936 |
| MA1133 | 880.4159614 | 1086.610792 | 1311.70013 | 1906.064242 | 3184.562548 | 2125.585373 |
| MA1132 | 65.46771383 | 80.80035744 | 97.53799626 | 474.7564935 | 793.2008353 | 529.4341272 |
| MA1131 | 230.1696123 | 284.0757047 | 342.9214412 | 1232.403028 | 2059.041055 | 1374.338699 |
| MA1130 | 91.71320533 | 113.1925852 | 136.6402117 | 1796.458583 | 3001.438564 | 2003.356446 |
| MA1129 | 83.98001704 | 103.6482718 | 125.1188121 | 298.1930246 | 498.20689 | 332.5358702 |
| MA1128 | 66.68928165 | 82.30801841 | 99.3579663 | 1366.580345 | 2283.218209 | 1523.969197 |
| MA1127 | 122.2414957 | 150.8706501 | 182.1232154 | 1921.463165 | 3210.290345 | 2142.757787 |
| MA1126 | 910.3094822 | 1123.505423 | 1356.237413 | 1467.843801 | 2452.404432 | 1636.895149 |
| MA1125 | 100.6055613 | 124.1675452 | 149.8886136 | 210.2452809 | 351.2679335 | 234.4591981 |
| MA1124 | 141.6040835 | 174.767987 | 210.9708398 | 376.5630726 | 629.1438827 | 419.9317847 |
| MA1123 | 36.0293193 | 44.46744368 | 53.67878921 | 205.4218311 | 343.2091403 | 229.0802324 |
| MA1122 | 31.73651737 | 39.16926065 | 47.28309775 | 166.4850923 | 278.1554673 | 185.6591553 |
| MA1121 | 43.05080433 | 53.13337177 | 64.13984766 | 287.3633187 | 480.1131264 | 320.4589086 |
| MA1120 | 98.25512947 | 121.2666384 | 146.3867896 | 976.5863423 | 1631.63456 | 1089.059644 |
| MA1119 | 164.3242085 | 202.8092017 | 244.8207383 | 1844.754418 | 3082.128975 | 2057.214505 |
| MA1118 | 163.7510805 | 202.1018463 | 243.9668555 | 1208.258075 | 2018.700802 | 1347.412975 |
| MA1117 | 256.1822932 | 316.1805972 | 381.6768005 | 572.1088154 | 955.8525189 | 637.9985012 |
| MA1115 | 296.7012133 | 366.1891135 | 442.0444849 | 440.3748133 | 735.7575399 | 491.092714 |
| MA1114 | 693.9190748 | 856.4360354 | 1033.845115 | 832.4392955 | 1390.800449 | 928.3112034 |
| MA1113 | 93.34499492 | 115.2065425 | 139.0713564 | 120.4447377 | 201.2334066 | 134.3163399 |
| MA1111 | 1070.075842 | 1320.68932 | 1594.267576 | 15513.73671 | 25919.62214 | 17300.45143 |
| MA1110 | 1025.455095 | 1265.618324 | 1527.788728 | 18791.55964 | 31396.05462 | 20955.78072 |
| MA1109 | 2173.801834 | 2682.909711 | 3238.669304 | 44575.77499 | 74475.10974 | 49709.56026 |
| MA1108 | 4739.3999 | 5849.374961 | 7061.061746 | 67083.71112 | 112080.3115 | 74809.73199 |
| MA1107 | 350.7304445 | 432.8720773 | 522.5406966 | 1567.974529 | 2619.698146 | 1748.557919 |
| MA1106 | 363.9169886 | 449.1469312 | 542.1868551 | 1613.886394 | 2696.405533 | 1799.757447 |
| MA1105 | 181.0310799 | 223.4288492 | 269.7117061 | 1262.693666 | 2109.649228 | 1408.117904 |
| MA1104 | 339.1721316 | 418.6067889 | 505.3203811 | 4137.608189 | 6912.921289 | 4614.135898 |
| MA1103 | 543.8481595 | 671.218271 | 810.2598463 | 6521.524747 | 10895.85703 | 7272.607766 |
| MA1102 | 280.8225292 | 346.591616 | 418.3874035 | 1042.352802 | 1741.51407 | 1162.400417 |
| MA1101a | 31.4803541 | 38.85310354 | 46.90144929 | 165.803178 | 277.0161568 | 184.8987049 |
| MA1101 | 44.56607502 | 55.00352129 | 66.39739506 | 263.5118085 | 440.2631443 | 293.8604237 |
| MA1100 | 500.0290005 | 617.1365947 | 744.9752547 | 4252.294648 | 7104.534034 | 4742.03078 |
| MA1098 | 35.53091836 | 43.85231645 | 52.93623956 | 215.6573454 | 360.3101565 | 240.4945693 |
| MA1097 | 860.1132307 | 1061.553129 | 1281.451821 | 794.7526437 | 1327.835363 | 886.2841856 |
| MA1096 | 894.9520601 | 1104.551268 | 1333.356942 | 8950.955126 | 14954.83512 | 9981.835275 |
| MA1095 | 3011.465168 | 3716.755141 | 4486.673829 | 27745.86109 | 46356.48061 | 30941.34771 |
| MA1094 | 862.7176725 | 1064.767536 | 1285.332085 | 31168.71485 | 52075.2238 | 34758.41102 |
| MA1093 | 954.0974359 | 1177.548586 | 1421.475513 | 28623.66465 | 47823.07355 | 31920.24778 |
| MA1092 | 878.2541847 | 1083.942724 | 1308.479377 | 23400.68247 | 39096.76041 | 26095.73553 |
| MA1091 | 1611.369796 | 1988.755187 | 2400.722004 | 55867.92375 | 93341.50114 | 62302.22409 |
| MA1090 | 1934.387096 | 2387.423657 | 2881.973881 | 67159.43582 | 112206.8288 | 74894.1779 |
| MA1089 | 918.4654394 | 1133.571519 | 1368.388681 | 29306.92258 | 48964.62878 | 32682.19642 |
| MA1088 | 1310.55184 | 1617.485183 | 1952.544132 | 43344.59003 | 72418.10377 | 48336.57991 |
| MA1087 | 585.7460053 | 722.9286595 | 872.6819425 | 18859.28205 | 31509.20204 | 21031.30271 |
| MA1086 | 253.0004736 | 312.2535904 | 376.9363218 | 8311.715289 | 13886.82324 | 9268.974283 |
| MA1085 | 818.9596195 | 1010.761276 | 1220.138533 | 20329.29297 | 33965.2272 | 22670.61457 |
| MA1084 | 1270.328284 | 1567.841207 | 1892.616501 | 34114.51972 | 56996.93612 | 38043.4838 |
| MA1083 | 1175.621952 | 1450.954499 | 1751.516936 | 41464.24576 | 69276.51295 | 46239.67668 |
| MA1082 | 419.6120741 | 517.8858951 | 625.1649634 | 11415.93686 | 19073.21074 | 12730.70858 |
| MA1081 | 655.7169415 | 809.2869012 | 976.9291281 | 24782.15943 | 41404.86718 | 27636.31698 |
| MA1080 | 802.2081499 | 990.0865856 | 1195.18112 | 27527.61822 | 45991.85069 | 30697.96985 |
| MA1079 | 239.2729679 | 295.3110809 | 356.484204 | 6891.08921 | 11513.30796 | 7684.734913 |
| MA1078 | 1790.660655 | 2210.036253 | 2667.841017 | 45202.93378 | 75522.93717 | 50408.94883 |
| MA1077 | 1377.475708 | 1700.082729 | 2052.251601 | 43671.35441 | 72964.04635 | 48700.9777 |
| MA1076 | 960.8866439 | 1185.927837 | 1431.590511 | 31229.89738 | 52177.4447 | 34826.63994 |
| MA1075 | 1031.090229 | 1272.573215 | 1536.184312 | 27666.02497 | 46223.09418 | 30852.31689 |
| MA1074 | 612.3626697 | 755.7789893 | 912.3371551 | 21670.66384 | 36206.32659 | 24166.47092 |
| MA1073 | 1444.499746 | 1782.803904 | 2152.108308 | 45602.45655 | 76190.44104 | 50854.48458 |
| MA1072 | 1611.337545 | 1988.715383 | 2400.673954 | 50116.73939 | 83732.69263 | 55888.6767 |
| MA1071 | 55.44185592 | 68.42642753 | 82.60083054 | 57.47203806 | 96.02157993 | 64.09108399 |
| MA1071a | 4.070889744 | 5.024298653 | 6.065072467 | 48.09199996 | 80.34985315 | 53.63074833 |
| MA1070 | 29.43128657 | 36.3241411 | 43.84861714 | 36.21657445 | 60.50895037 | 40.38763186 |
| MA1069 | 521.113211 | 643.1587611 | 776.3878629 | 2003.747849 | 3347.767728 | 2234.519186 |
| MA1068 | 1207.807844 | 1490.678378 | 1799.469541 | 9416.086601 | 15731.95493 | 10500.53587 |
| MA1067 | 471.1712494 | 581.5203119 | 701.9811274 | 5038.684868 | 8418.397853 | 5618.989443 |
| MA1066 | 196.1345476 | 242.0695734 | 292.2138204 | 2185.61077 | 3651.615748 | 2437.327232 |
| MA1065 | 89.92248726 | 110.982478 | 133.9722852 | 960.9089914 | 1605.441579 | 1071.576735 |
| MA1064 | 153.2463997 | 189.1369524 | 228.3163087 | 1443.552293 | 2411.819322 | 1609.805992 |
| MA1063 | 338.4987511 | 417.7757017 | 504.3171357 | 1465.201312 | 2447.989486 | 1633.948326 |
| MA1062 | 674.4190258 | 832.3690437 | 1004.792692 | 3417.448274 | 5709.711952 | 3811.035275 |
| MA1061 | 289.1236308 | 356.836849 | 430.7549168 | 1542.059744 | 2576.400941 | 1719.658532 |
| MA1060 | 825.7233418 | 1019.109073 | 1230.21556 | 2650.787773 | 4428.811621 | 2956.0786 |
| MA1059 | 2248.247618 | 2774.790817 | 3349.583404 | 6082.1465 | 10161.7645 | 6782.626393 |
| MA1058b | 0.066872407 | 0.082534032 | 0.099630799 | 670.4271053 | 1120.118097 | 747.6400937 |
| MA1058 | 75.69669974 | 93.42498826 | 112.7777951 | 397.9067204 | 664.8038464 | 443.7335773 |
| MA1057 | 162.3074403 | 200.3201032 | 241.8160277 | 277.9291632 | 464.3509828 | 309.9382229 |
| MA1056 | 22.7321765 | 28.05608871 | 33.86785358 | 65.04785355 | 108.6788964 | 72.53940501 |
| MA1054a | 170.5130046 | 210.4474238 | 254.0412034 | 385.7063095 | 644.4199732 | 430.1280469 |
| MA1054 | 206.3328883 | 254.6563818 | 307.407962 | 1853.5243 | 3096.781283 | 2066.994414 |
| MA1053 | 484.1620875 | 597.5536251 | 721.3357106 | 3433.510315 | 5736.547655 | 3828.947179 |
| MA1052a | 17.14348772 | 21.15852004 | 25.54146682 | 139.438592 | 232.9674458 | 155.4977136 |
| MA1052 | 11.2435778 | 13.87684176 | 16.75140287 | 79.22762265 | 132.3697881 | 88.35225598 |
| MA1050n | 49.38573609 | 60.95195472 | 73.57803504 | 385.2108023 | 643.592103 | 429.5754721 |
| MA1049 | 132.8547896 | 163.9695944 | 197.9355809 | 645.2633926 | 1078.075748 | 719.57828 |
| MA1048 | 269.2428626 | 332.2999729 | 401.1352739 | 1227.610313 | 2051.033613 | 1368.994008 |
| MA1047 | 28.8459474 | 35.60171457 | 42.97654134 | 94.01683083 | 157.078902 | 104.844735 |
| MA1046 | 40.93062846 | 50.51664732 | 60.98107376 | 626.6637924 | 1047.00041 | 698.8365667 |
| MA1045 | 104.5726467 | 129.0637284 | 155.7990318 | 1397.116373 | 2334.23637 | 1558.022055 |
| MA1044 | 96.04505785 | 118.5389645 | 143.0940833 | 230.4615391 | 385.0443076 | 257.0037598 |
| MA1043 | 272.7811993 | 336.6669938 | 406.4069148 | 517.0054384 | 863.7883865 | 576.5488766 |
| MA1042a | 14.24238694 | 17.57797681 | 21.21922094 | 171.999051 | 287.3679302 | 191.8081557 |
| MA1042 | 4107.041341 | 5068.917012 | 6118.933433 | 9835.168515 | 16432.13729 | 10967.88339 |
| MA1041 | 51.91124755 | 64.06894501 | 77.34070389 | 120.1406075 | 200.7252802 | 133.9771831 |
| MA1040 | 71.51213056 | 88.26038627 | 106.5433557 | 565.3877857 | 944.6233384 | 630.5034115 |
| MA1039a | 78.67899432 | 97.10574101 | 117.2210087 | 246.439125 | 411.7389071 | 274.8214818 |
| MA1036 | 74.01186181 | 91.34555858 | 110.2676156 | 430.7390665 | 719.658587 | 480.3472197 |
| MA1035 | 29.72751483 | 36.6897465 | 44.289957 | 125.224301 | 209.2188764 | 139.6463648 |
| MA1034 | 555.3055839 | 685.3590425 | 827.3298516 | 2533.268315 | 4232.465634 | 2825.024444 |
| MA1033 | 1242.235238 | 1533.168724 | 1850.76168 | 1613.750268 | 2696.178099 | 1799.605642 |
| MA1032 | 190.6593592 | 235.3120871 | 284.0565338 | 1237.467919 | 2067.503236 | 1379.986912 |
| MA1031 | 800.0273207 | 987.3950028 | 1191.931981 | 2948.246737 | 4925.792077 | 3287.795868 |
| MA1030 | 377.1692781 | 465.5029281 | 561.9309654 | 1533.370744 | 2561.883769 | 1709.968821 |
| MA1029 | 140.7349796 | 173.6953377 | 209.6759932 | 615.2180065 | 1027.877329 | 686.0725714 |
| MA1028 | 450.8441255 | 556.432543 | 671.6964754 | 1231.271116 | 2057.149909 | 1373.076424 |
| MA1027 | 89.2073854 | 110.0998982 | 132.9068806 | 300.9331164 | 502.7849067 | 335.5915379 |
| MA1024n | 75.3890974 | 93.04534496 | 112.3195094 | 992.1368142 | 1657.615557 | 1106.401061 |
| MA1023 | 308.5262203 | 380.7835561 | 459.6621382 | 3240.177967 | 5413.537055 | 3613.348774 |
| MA1022 | 1008.475831 | 1244.662488 | 1502.491931 | 9686.246651 | 16183.32564 | 10801.81021 |
| MA1021 | 611.8205858 | 755.1099486 | 911.5295238 | 1704.591417 | 2847.951222 | 1900.908953 |
| MA1020 | 637.7675636 | 787.1337502 | 950.1869943 | 787.4509358 | 1315.636012 | 878.1415411 |
| MA1019 | 171.1873147 | 211.2796585 | 255.0458339 | 596.0707034 | 995.8869151 | 664.7200762 |
| MA1018 | 431.5476052 | 532.6167467 | 642.9472826 | 1682.430936 | 2810.926532 | 1876.196253 |
| MA1017 | 680.167503 | 839.4638235 | 1013.357142 | 1973.363519 | 3297.003018 | 2200.635498 |
| MA1016 | 3789.868281 | 4677.461513 | 5646.388679 | 15325.44874 | 25605.03944 | 17090.47836 |
| MA1015 | 907.2793278 | 1119.765602 | 1351.722895 | 3877.3353 | 6478.06958 | 4323.88742 |
| MA1014 | 3263.020225 | 4027.224797 | 4861.456677 | 16173.56598 | 27022.03386 | 18036.27313 |
| MA1013 | 1796.380515 | 2217.095713 | 2676.362831 | 9335.207179 | 15596.82539 | 10410.34158 |
| MA1012 | 2819.289879 | 3479.572091 | 4200.358766 | 10905.10833 | 18219.74244 | 12161.04801 |
| MA1011 | 2972.945913 | 3669.214616 | 4429.285375 | 12090.43128 | 20200.12431 | 13482.88442 |
| MA1009 | 2467.498768 | 3045.390939 | 3676.237821 | 4064.587986 | 6790.922566 | 4532.70597 |
| MA1008 | 461.1118716 | 569.1050118 | 686.9940217 | 765.5054817 | 1278.970579 | 853.6686324 |
| MA1007 | 285.6591542 | 352.5609864 | 425.5933174 | 1594.693573 | 2664.33907 | 1778.354191 |
| MA1006 | 494.4584579 | 610.261422 | 736.6759032 | 2219.059245 | 3707.499887 | 2474.627962 |
| MA1005 | 473.0082212 | 583.7875055 | 704.7179658 | 2238.296072 | 3739.639874 | 2496.080292 |
| MA1004 | 108.9228726 | 134.4327842 | 162.2802772 | 504.6811157 | 843.1974875 | 562.8051635 |
| MA1003 | 421.9551731 | 520.7777516 | 628.6558623 | 1030.115056 | 1721.067818 | 1148.753251 |
| MA1002 | 110.622765 | 136.530794 | 164.8128857 | 294.7058348 | 492.3806572 | 328.6470612 |
| MA1001 | 306.2880989 | 378.0212631 | 456.3276415 | 633.0118377 | 1057.606426 | 705.9157154 |
| MA1000 | 136.7714648 | 168.8035613 | 203.7708947 | 788.1289774 | 1316.768852 | 878.8976726 |
| MA0999 | 471.9376361 | 582.4661876 | 703.1229394 | 2883.302636 | 4817.286526 | 3215.372165 |
| MA0997n | 208.0106517 | 256.7270801 | 309.9076015 | 950.970996 | 1588.837643 | 1060.494182 |
| MA0996 | 138.9174272 | 171.4521116 | 206.9680871 | 1061.553777 | 1773.594156 | 1183.812765 |
| MA0995 | 221.0369197 | 272.8041209 | 329.3149705 | 1169.420265 | 1953.812414 | 1304.10222 |
| MA0994 | 32.23375184 | 39.78294824 | 48.02390954 | 146.4347581 | 244.6563112 | 163.299627 |
| MA0993 | 64.71988636 | 79.87738758 | 96.42383496 | 263.5403607 | 440.310848 | 293.8922642 |
| MA0992 | 73.05292752 | 90.16204034 | 108.8389338 | 475.7720251 | 794.8975377 | 530.5666174 |
| MA0991 | 49.303292 | 60.85020209 | 73.45520455 | 202.7578816 | 338.7583387 | 226.1094762 |
| MA0990 | 80.5821697 | 99.45464313 | 120.0564814 | 375.6446921 | 627.6094958 | 418.9076345 |
| MA0989 | 130.2017802 | 160.6952461 | 193.9829576 | 612.1906438 | 1022.819354 | 682.6965477 |
| MA0988 | 78.81132381 | 97.26906228 | 117.4181617 | 363.0276871 | 606.5295968 | 404.837531 |
| MA0987 | 267.3688124 | 329.9870172 | 398.3431939 | 524.6215781 | 876.5130749 | 585.042166 |
| MA0986 | 260.1054635 | 321.0225804 | 387.5217912 | 252.2276658 | 421.410129 | 281.276688 |
| MA0985 | 455.2454339 | 561.8646449 | 678.2538267 | 562.207361 | 939.3096344 | 626.9566978 |
| MA0984 | 23.03031071 | 28.42404642 | 34.31203304 | 282.1055046 | 471.3286178 | 314.595553 |
| MA0983 | 25.15392871 | 31.04501917 | 37.47593526 | 62.87355271 | 105.0461767 | 70.11469028 |
| MA0982 | 354.7690853 | 437.8565743 | 528.5577225 | 405.9052728 | 678.1674517 | 452.6533217 |
| MA0981 | 252.9285362 | 312.1648052 | 376.8291449 | 785.7341939 | 1312.767761 | 876.2270823 |
| MA0980 | 536.0041326 | 661.5371604 | 798.5733123 | 1014.884217 | 1695.620849 | 1131.768279 |
| MA0979 | 399.9189849 | 493.5806528 | 595.8249368 | 1589.02988 | 2654.876437 | 1772.038211 |
| MA0978 | 284.4237519 | 351.0362509 | 423.7527359 | 564.8012863 | 943.6434427 | 629.849365 |
| MA0977 | 346.2600825 | 427.3547494 | 515.8804647 | 597.9408778 | 999.0115147 | 666.8056384 |
| MA0976 | 220.3743616 | 271.9863906 | 328.3278489 | 334.3079902 | 558.5460771 | 372.8101909 |
| MA0975 | 683.9403141 | 844.1202329 | 1018.978118 | 1003.084391 | 1675.906254 | 1118.609469 |
| MA0974 | 16.72397614 | 20.64075818 | 24.91645158 | 39.33659077 | 65.72172699 | 43.86697998 |
| MA0972 | 2470.724102 | 3049.37165 | 3681.04313 | 4045.343901 | 6758.770454 | 4511.245547 |
| MA0971 | 278.0388481 | 343.1559924 | 414.240097 | 771.8172097 | 1289.515918 | 860.7072811 |
| MA0970 | 1742.042757 | 2150.031966 | 2595.406957 | 2980.583218 | 4979.818349 | 3323.856536 |
| MA0969 | 235.4270684 | 290.5644655 | 350.7543364 | 1350.791508 | 2256.838963 | 1506.361962 |
| MA0968 | 639.8675021 | 789.7254977 | 953.3156174 | 3632.538501 | 6069.074593 | 4050.897411 |
| MA0967 | 74.09090123 | 91.44310916 | 110.3853736 | 366.9725681 | 613.1205186 | 409.2367433 |
| MA0966 | 55.1358577 | 68.04876403 | 82.14493477 | 204.5629999 | 341.7742455 | 228.1224897 |
| MA0965 | 242.3089772 | 299.0581285 | 361.0074453 | 314.7521276 | 525.8730611 | 351.0020826 |
| MA0964 | 6120.650617 | 7554.116811 | 9118.937597 | 1712.286441 | 2860.807706 | 1909.490211 |
| MA0963 | 3640.572286 | 4493.200156 | 5423.957936 | 5494.896566 | 9180.614915 | 6127.742973 |
| MA0962 | 2519.636138 | 3109.738964 | 3753.915416 | 4477.534005 | 7480.853366 | 4993.21092 |
| MA0961 | 342.4112568 | 422.6045225 | 510.1462375 | 1407.651744 | 2351.838372 | 1569.770782 |
| MA0960 | 1443.577787 | 1781.66602 | 2150.734714 | 2532.050876 | 4230.431594 | 2823.666793 |
| MA0959 | 374.4784228 | 462.1818703 | 557.9219568 | 5022.521231 | 8391.392407 | 5600.964242 |
| MA0958 | 412.5252444 | 509.1393184 | 614.6065502 | 5940.764138 | 9925.549498 | 6624.961046 |
| MA0957 | 3127.967861 | 3860.542952 | 4660.247008 | 6035.189474 | 10083.31091 | 6730.261334 |
| MA0956 | 40.33205166 | 49.77788287 | 60.08927567 | 96.33176129 | 160.9465791 | 107.4262757 |
| MA0955 | 564.4530439 | 696.6488523 | 840.9583238 | 2116.486039 | 3536.125397 | 2360.241417 |
| MA0954a | 64.45160947 | 79.54627981 | 96.02413886 | 377.8487 | 631.2918487 | 421.3654777 |
| MA0954 | 1044.254155 | 1288.820154 | 1555.796773 | 1680.987785 | 2808.515383 | 1874.586894 |
| MA0953 | 612.7395005 | 756.2440745 | 912.8985817 | 1183.925018 | 1978.046274 | 1320.277483 |
| MA0952 | 1169.647798 | 1443.581189 | 1742.61626 | 17828.32401 | 29786.7258 | 19881.60939 |
| MA0951 | 683.0070248 | 842.9683658 | 1017.587643 | 3782.942231 | 6320.362077 | 4218.623116 |
| MA0950 | 312.1490787 | 385.2548937 | 465.0597048 | 2331.010944 | 3894.543525 | 2599.473122 |
| MA0949 | 2915.872923 | 3598.77504 | 4344.254376 | 24774.79188 | 41392.55783 | 27628.10092 |
| MA0948 | 32.88968716 | 40.59250466 | 49.00116402 | 81.16977354 | 135.6146425 | 90.51808409 |
| MA0947 | 5.131701838 | 6.333554641 | 7.645538318 | 70.84086226 | 118.3575831 | 78.99959366 |
| MA0946 | 27.72317358 | 34.21598531 | 41.30376094 | 110.3404607 | 184.3516556 | 123.0483549 |
| MA0945 | 45.45741364 | 56.10361285 | 67.72536847 | 330.333412 | 551.9055387 | 368.3778611 |
| MA0944 | 22.60693272 | 27.90151264 | 33.68125738 | 0.213480223 | 0.356672723 | 0.238066708 |
| MA0943 | 448.8532536 | 553.9754057 | 668.7303468 | 4488.689976 | 7499.492239 | 5005.651723 |
| MA0942 | 3464.082173 | 4275.375776 | 5161.011654 | 20794.36455 | 34742.24692 | 23189.24837 |
| MA0941 | 731.935644 | 903.3561461 | 1090.484636 | 4296.526052 | 7178.433785 | 4791.356308 |
| MA0940 | 642.8443747 | 793.3995586 | 957.750753 | 6009.016234 | 10039.58189 | 6701.073726 |
| MA0939 | 807.2019823 | 996.2499815 | 1202.621251 | 8708.197948 | 14549.24784 | 9711.11979 |
| MA0938 | 350.6934204 | 432.8263821 | 522.4855357 | 3663.538191 | 6120.867419 | 4085.467331 |
| MA0937 | 1178.072319 | 1453.978746 | 1755.16765 | 9581.797375 | 16008.8167 | 10685.33153 |
| MA0936 | 876.164212 | 1081.363276 | 1305.365601 | 6549.079923 | 10941.89492 | 7303.336467 |
| MA0935 | 219.3086075 | 270.6710352 | 326.7400202 | 2026.963961 | 3386.556116 | 2260.409094 |
| MA0934 | 437.1058805 | 539.476779 | 651.2283574 | 814.2131855 | 1360.349121 | 907.985995 |
| MA0933 | 377.9654202 | 466.485528 | 563.117109 | 780.8513457 | 1304.609727 | 870.7818771 |
| MA0932 | 247.4939172 | 305.4573896 | 368.7323011 | 850.7328742 | 1421.364501 | 948.7116507 |
| MA0931 | 118.1173293 | 145.7805973 | 175.9787679 | 403.3168869 | 673.8428982 | 449.7668318 |
| MA0929n | 119.2733792 | 147.207396 | 177.7011252 | 295.830421 | 494.2595629 | 329.9011658 |
| MA0928 | 13.71319793 | 16.92485089 | 20.43080124 | 41.22894719 | 68.88338715 | 45.97727881 |
| MA0927 | 268.5106097 | 331.3962253 | 400.0443167 | 1358.762516 | 2270.156548 | 1515.250989 |
| MA0926 | 1118.488526 | 1380.440334 | 1666.395897 | 4013.313175 | 6705.255022 | 4475.525847 |
| MA0925 | 905.7234577 | 1117.845345 | 1349.40486 | 12971.06578 | 21671.44705 | 14464.94147 |
| MA0924 | 1781.00554 | 2198.119895 | 2653.456208 | 15758.76468 | 26329.00335 | 17573.69923 |
| MA0923 | 3455.672583 | 4264.996646 | 5148.482507 | 34874.65119 | 58266.92805 | 38891.15949 |
| MA0922 | 856.9837927 | 1057.690772 | 1276.789383 | 4131.140987 | 6902.116192 | 4606.92387 |
| MA0921 | 4891.217191 | 6036.748105 | 7287.248877 | 4796.147725 | 8013.178193 | 5348.519333 |
| MA0920 | 596.7351023 | 736.4914206 | 889.0542035 | 2656.523221 | 4438.394138 | 2962.474599 |
| MA0919 | 2632.262821 | 3248.742996 | 3921.713867 | 6537.394912 | 10922.37215 | 7290.305696 |
| MA0918 | 207.5978745 | 256.2176298 | 309.2926196 | 576.2721741 | 962.8084629 | 642.6413533 |
| MA0917 | 2140.123291 | 2641.343599 | 3188.492851 | 9841.220788 | 16442.24914 | 10974.6327 |
| MA0916 | 4982.440726 | 6149.336338 | 7423.159546 | 12541.99685 | 20954.57885 | 13986.45673 |
| MA0915 | 3234.181987 | 3991.632598 | 4818.491622 | 10708.29234 | 17890.91154 | 11941.56475 |
| MA0914 | 728.8619911 | 899.5626388 | 1085.905311 | 7877.693101 | 13161.67937 | 8784.965825 |
| MA0913 | 236.2576383 | 291.5895563 | 351.9917727 | 2181.537792 | 3644.810808 | 2432.78517 |
| MA0912a | 4158.004526 | 5131.815856 | 6194.861652 | 3547.470318 | 5926.946671 | 3956.031938 |
| MA0912 | 37.11858055 | 45.81181167 | 55.30164045 | 109.2249717 | 182.4879491 | 121.8043952 |
| MA0911 | 30.27784875 | 37.36896951 | 45.10987975 | 102.8690807 | 171.868825 | 114.7164971 |
| MA0910 | 72.05946966 | 88.93591306 | 107.3588166 | 158.2543056 | 264.4038557 | 176.4804301 |
| MA0909 | 1302.857837 | 1607.989232 | 1941.081113 | 6942.485086 | 11599.17778 | 7742.050044 |
| MA0908 | 90.14046658 | 111.2515085 | 134.2970447 | 559.2016369 | 934.2878118 | 623.604805 |
| MA0907 | 85.65495926 | 105.7154881 | 127.6142484 | 129.7747346 | 216.821527 | 144.7208712 |
| MA0906 | 70.30417267 | 86.7695227 | 104.7436626 | 89.29919636 | 149.1969001 | 99.58377131 |
| MA0905 | 4414.093672 | 5447.881492 | 6576.399678 | 8010.328661 | 13383.28063 | 8932.877002 |
| MA0904 | 4466.03154 | 5511.983292 | 6653.780042 | 18805.03953 | 31418.57619 | 20970.81309 |
| MA0903 | 9101.657926 | 11233.28081 | 13560.23335 | 27998.09997 | 46777.90948 | 31222.63691 |
| MA0902 | 2299.002134 | 2837.432122 | 3425.200735 | 5078.763086 | 8485.358656 | 5663.683463 |
| MA0901 | 826.6725066 | 1020.280534 | 1231.629687 | 1217.98037 | 2034.944355 | 1358.254985 |
| MA0895 | 2050.321296 | 2530.509833 | 3054.700083 | 6283.019074 | 10497.37296 | 7006.633431 |
| MA0894 | 2305.554545 | 2845.519118 | 3434.962937 | 10178.85198 | 17006.34747 | 11351.14882 |
| MA0893 | 948.3189868 | 1170.416815 | 1412.86641 | 4869.888449 | 8136.380729 | 5430.752765 |
| MA0892 | 507.0532749 | 625.8059654 | 755.4404689 | 2867.747867 | 4791.298348 | 3198.025955 |
| MA0891 | 150.3146581 | 185.5185922 | 223.9484122 | 361.9196745 | 604.6783815 | 403.6019088 |
| MA0890 | 70.20066578 | 86.64177433 | 104.5894514 | 256.1023884 | 427.8838335 | 285.5976618 |
| MA0889 | 33.3299996 | 41.13593898 | 49.65716972 | 177.7373792 | 296.9552592 | 198.2073663 |
| MA0888 | 34.04847607 | 42.0226838 | 50.72760202 | 171.820124 | 287.0689874 | 191.6086217 |
| MA0887 | 250.1825568 | 308.775713 | 372.7380088 | 2846.574203 | 4755.922386 | 3174.413724 |
| MA0886 | 24.36465983 | 30.07090225 | 36.30003188 | 44.52721741 | 74.39398202 | 49.65541031 |
| MA0885 | 2860.280018 | 3530.162187 | 4261.428502 | 15512.98226 | 25918.36163 | 17299.61008 |
| MA0884 | 416.3112803 | 513.8120501 | 620.2472292 | 1530.378145 | 2556.883874 | 1706.631564 |
| MA0883 | 447.5639157 | 552.3841028 | 666.8094085 | 3237.808068 | 5409.577539 | 3610.705934 |
| MA0882 | 1192.109883 | 1471.303931 | 1776.081713 | 4533.734682 | 7574.750815 | 5055.88422 |
| MA0881a | 10.08541319 | 12.44743313 | 15.025895 | 56.84564045 | 94.97502426 | 63.39254427 |
| MA0881 | 88.26254203 | 108.933771 | 131.4991924 | 438.4566833 | 732.5528185 | 488.9536733 |
| MA0880 | 93.14194836 | 114.9559421 | 138.7688447 | 337.3053268 | 563.5538862 | 376.1527303 |
| MA0879 | 92.25540312 | 113.8617665 | 137.4480127 | 376.5638294 | 629.1451471 | 419.9326287 |
| MA0878 | 74.7764391 | 92.2892011 | 111.4067318 | 555.7515835 | 928.5236247 | 619.7574094 |
| MA0877 | 97.29638954 | 120.0833601 | 144.9583974 | 3149.831704 | 5262.590765 | 3512.597345 |
| MA0876 | 43.52173731 | 53.71459802 | 64.8414738 | 2537.614776 | 4239.727497 | 2829.871486 |
| MA0875 | 88.68291442 | 109.4525953 | 132.1254901 | 1405.220605 | 2347.776539 | 1567.059649 |
| MA0874 | 222.6928453 | 274.847867 | 331.7820746 | 4041.197108 | 6751.842187 | 4506.621168 |
| MA0873 | 269.8978325 | 333.1083379 | 402.1110902 | 5292.230472 | 8842.009932 | 5901.735856 |
| MA0872 | 511.6822927 | 631.5191066 | 762.3370764 | 9673.399903 | 16161.8619 | 10787.48391 |
| MA0871 | 97.06348027 | 119.7959031 | 144.6113943 | 1692.659359 | 2828.0157 | 1887.602681 |
| MA0870 | 175.0614038 | 216.0610654 | 260.8177001 | 1959.667675 | 3274.120645 | 2185.362305 |
| MA0869 | 65.78709907 | 81.19454322 | 98.01383686 | 343.1666951 | 573.346785 | 382.6891515 |
| MA0868 | 248.1894992 | 306.3158781 | 369.7686237 | 875.632091 | 1462.964942 | 976.4785066 |
| MA0867 | 65.44240228 | 80.76911788 | 97.50028549 | 263.3474149 | 439.9884831 | 293.6770969 |
| MA0866 | 1196.383887 | 1476.578913 | 1782.449398 | 15039.34859 | 25127.03676 | 16771.4281 |
| MA0865 | 20.42527558 | 25.20890789 | 30.43088473 | 89.34127648 | 149.2672056 | 99.63069778 |
| MA0864 | 1238.975216 | 1529.145199 | 1845.904689 | 4073.515028 | 6805.837449 | 4542.661139 |
| MA0863 | 2548.849189 | 3145.793758 | 3797.438892 | 8802.865829 | 14707.41448 | 9816.690556 |
| MA0862 | 487.9976179 | 602.2874429 | 727.0501297 | 2432.150735 | 4063.523133 | 2712.261167 |
| MA0861 | 76.42275759 | 94.32109002 | 113.8595226 | 367.6892805 | 614.3179679 | 410.0359993 |
| MA0860 | 129.5785059 | 159.9259999 | 193.0543636 | 354.4936955 | 592.2714048 | 395.3206808 |
| MA0859 | 3030.161479 | 3739.830158 | 4514.528791 | 794.9584755 | 1328.179257 | 886.513723 |
| MA0858 | 2000.951824 | 2469.577952 | 2981.14628 | 563.1163594 | 940.8283462 | 627.9703854 |
| MA0857 | 1719.70257 | 2122.459671 | 2562.123115 | 1240.843311 | 2073.142683 | 1383.751048 |
| MA0856 | 418.8912244 | 516.9962211 | 624.0909952 | 597.8593697 | 998.8753347 | 666.7147429 |
| MA0855 | 715.9984566 | 883.6864439 | 1066.740393 | 2510.65491 | 4194.68422 | 2799.806657 |
| MA0854 | 229.0257231 | 282.6639149 | 341.2172019 | 433.2085957 | 723.7845604 | 483.1011642 |
| MA0853 | 199.8034862 | 246.5977832 | 297.6800403 | 566.450661 | 946.3991406 | 631.688698 |
| MA0852 | 78.95981625 | 97.45233189 | 117.6393952 | 530.3386974 | 886.064969 | 591.417725 |
| MA0851 | 1130.696495 | 1395.507428 | 1684.584111 | 3469.810784 | 5797.196773 | 3869.428368 |
| MA0850 | 2439.341159 | 3010.638772 | 3634.28681 | 6429.780911 | 10742.57574 | 7170.297807 |
| MA0849 | 1470.069863 | 1814.362583 | 2190.204307 | 388.6906179 | 649.4060155 | 433.4560577 |
| MA0848 | 183.8708588 | 226.9337089 | 273.9425907 | 77.89051606 | 130.1358133 | 86.86115503 |
| MA0847 | 1621.045361 | 2000.696785 | 2415.137281 | 3125.810459 | 5222.457198 | 3485.809577 |
| MA0846 | 139.7201268 | 172.4428047 | 208.1640004 | 724.0069499 | 1209.636783 | 807.3907209 |
| MA0845 | 176.8330467 | 218.2476299 | 263.4572072 | 1274.684547 | 2129.683029 | 1421.489774 |
| MA0844 | 218.4454367 | 269.6057085 | 325.4540131 | 3002.041632 | 5015.670059 | 3347.786313 |
| MA0843 | 171.8207634 | 212.0614619 | 255.9895863 | 2621.702336 | 4380.217039 | 2923.643397 |
| MA0842 | 302.8248876 | 373.7469621 | 451.1679273 | 6096.888886 | 10186.39539 | 6799.066657 |
| MA0841 | 446.9690438 | 551.6499109 | 665.9231302 | 11303.62139 | 18885.55933 | 12605.45775 |
| MA0840 | 1180.861181 | 1457.420764 | 1759.322675 | 31115.52187 | 51986.35146 | 34699.09182 |
| MA0839 | 42.19033873 | 52.07138375 | 62.85787086 | 175.3511562 | 292.9684701 | 195.5463224 |
| MA0837 | 104.1605521 | 128.5551205 | 155.1850668 | 379.8917421 | 634.7052674 | 423.6438167 |
| MA0836 | 270.5055352 | 333.8583655 | 403.0164845 | 1695.954388 | 2833.52088 | 1891.277199 |
| MA0835; | 1396.147316 | 1723.127257 | 2080.069759 | 3335.400348 | 5572.630133 | 3719.537901 |
| MA0834 | 4064.168859 | 5016.003726 | 6055.059262 | 10037.49281 | 16770.17122 | 11193.50934 |
| MA0833 | 6568.318227 | 8106.628893 | 9785.901495 | 14214.69533 | 23749.24485 | 15851.79965 |
| MA0832 | 6367.675049 | 7858.994762 | 9486.970429 | 15425.53481 | 25772.25853 | 17202.09132 |
| MA0831 | 248.1629477 | 306.2831081 | 369.7290656 | 583.3462134 | 974.6274353 | 650.5301086 |
| MA0830 | 1190.037943 | 1468.746739 | 1772.994804 | 8892.129046 | 14856.55127 | 9916.234204 |
| MA0829 | 163701.647 | 202040.8354 | 243893.2063 | 725742.1725 | 1212535.911 | 809325.7888 |
| MA0828 | 43.63022026 | 53.84848784 | 65.00309865 | 287.5304886 | 480.392426 | 320.6453315 |
| MA0827 | 1681.79642 | 2075.67584 | 2505.648102 | 16264.58957 | 27174.1118 | 18137.77989 |
| MA0826 | 805.9872261 | 994.7507275 | 1200.81143 | 6698.342606 | 11191.276 | 7469.789712 |
| MA0825 | 1171.157759 | 1445.444785 | 1744.865895 | 7185.99927 | 12006.02983 | 8013.60972 |
| MA0824 | 554.4193644 | 684.2652691 | 826.0095051 | 4491.58088 | 7504.322224 | 5008.875571 |
| MA0823 | 1131.319685 | 1396.27657 | 1685.512579 | 5950.038012 | 9941.043851 | 6635.30299 |
| MA0822 | 1319.604362 | 1628.657821 | 1966.03116 | 6292.85686 | 10513.80947 | 7017.604233 |
| MA0821 | 926.3989229 | 1143.363037 | 1380.208494 | 3965.773689 | 6625.828283 | 4422.511245 |
| MA0820 | 408.2028372 | 503.8045965 | 608.1667508 | 1563.097789 | 2611.550319 | 1743.119526 |
| MA0819 | 554.1191178 | 683.8947042 | 825.5621783 | 5073.729191 | 8476.948261 | 5658.069815 |
| MA0818 | 927.3981501 | 1144.596285 | 1381.697206 | 4643.155672 | 7757.566262 | 5177.907209 |
| MA0816n | 770.3459208 | 950.7621713 | 1147.710729 | 5908.737319 | 9872.040594 | 6589.245703 |
| MA0815 | 1328.393608 | 1639.505523 | 1979.125942 | 16762.34342 | 28005.73554 | 18692.85998 |
| MA0814 | 7830.074452 | 9663.890451 | 11665.74679 | 21243.0252 | 35491.84805 | 23689.5812 |
| MA0813 | 12093.69265 | 14926.05487 | 18017.95846 | 24487.53516 | 40912.6228 | 27307.76088 |
| MA0812 | 375.5324351 | 463.4827339 | 559.4922918 | 897.0265063 | 1498.709725 | 1000.336914 |
| MA0811 | 561.3432649 | 692.8107581 | 836.3251757 | 1807.007204 | 3019.062705 | 2015.119951 |
| MA0810 | 1372.235265 | 1693.614965 | 2044.444052 | 1936.027553 | 3234.62384 | 2158.999554 |
| MA0809 | 2411.757397 | 2976.594848 | 3593.190753 | 2667.249358 | 4456.314863 | 2974.436063 |
| MA0808 | 2233.936905 | 2757.128513 | 3328.262388 | 2224.012535 | 3715.775612 | 2480.151722 |
| MA0807 | 929.1993771 | 1146.819362 | 1384.38079 | 1251.809457 | 2091.464405 | 1395.980163 |
| MA0806 | 787.4012217 | 971.8118511 | 1173.120809 | 1009.229508 | 1686.173227 | 1125.462318 |
| MA0805 | 1275.156324 | 1573.799981 | 1899.809623 | 1461.304864 | 2441.479483 | 1629.603124 |
| MA0804 | 1012.654089 | 1249.819301 | 1508.716968 | 1845.952481 | 3084.130644 | 2058.550549 |
| MA0803 | 243.4703318 | 300.4914741 | 362.7377057 | 738.2601693 | 1233.450392 | 823.2854814 |
| MA0802 | 577.2665766 | 712.4633349 | 860.0487461 | 2128.785906 | 3556.675436 | 2373.957857 |
| MA0800 | 376.5686047 | 464.7615761 | 561.0360437 | 837.5310292 | 1399.307479 | 933.9893513 |
| MA0799 | 92.52436517 | 114.1937199 | 137.8487296 | 211.8017968 | 353.8684871 | 236.1949777 |
| MA0798 | 65.49344543 | 80.83211543 | 97.57633285 | 163.4400964 | 273.0680311 | 182.2634676 |
| MA0797 | 44.62327079 | 55.0741124 | 66.48260898 | 114.7402952 | 191.7026923 | 127.9549177 |
| MA0796 | 148.0687151 | 182.7466457 | 220.6022623 | 221.7719381 | 370.5261306 | 247.3133787 |
| MA0795 | 281.3901358 | 347.2921571 | 419.2330602 | 278.7912669 | 465.7913452 | 310.899615 |
| MA0794 | 892.8288674 | 1101.930821 | 1330.193673 | 904.9535997 | 1511.95394 | 1009.176969 |
| MA0793 | 374.0538125 | 461.6578155 | 557.2893451 | 283.2635422 | 473.2634125 | 315.8869615 |
| MA0792 | 235.5813874 | 290.7549262 | 350.9842506 | 324.1646579 | 541.5990742 | 361.4986525 |
| MA0791 | 196.427603 | 242.4312628 | 292.6504332 | 609.0041135 | 1017.495449 | 679.1430252 |
| MA0790 | 180.3676497 | 222.6100427 | 268.7232854 | 1394.134552 | 2329.254484 | 1554.696818 |
| MA0788n | 17.48554956 | 21.58069332 | 26.05109248 | 33.84678375 | 56.54961545 | 37.74491272 |
| MA0787 | 11.37533228 | 14.03945335 | 16.94769914 | 78.03417562 | 130.3758329 | 87.02135983 |
| MA0786 | 5.866923792 | 7.240966756 | 8.74091911 | 42.7435188 | 71.41386221 | 47.6662834 |
| MA0785 | 11904.58892 | 14692.66275 | 17736.21961 | 27666.92458 | 46224.5972 | 30853.3201 |
| MA0784 | 124.0538043 | 153.1074043 | 184.823309 | 924.6263348 | 1544.822222 | 1031.11541 |
| MA0783 | 6829.648463 | 8429.16309 | 10175.24803 | 11085.46342 | 18521.07125 | 12362.17456 |
| MA0782 | 219.3493031 | 270.7212618 | 326.8006511 | 605.5462635 | 1011.718236 | 675.2869351 |
| MA0781 | 202.8811042 | 250.3961843 | 302.2652729 | 963.7964232 | 1610.265764 | 1074.796712 |
| MA0780 | 115.8404286 | 142.9704428 | 172.5864953 | 897.2133484 | 1499.021892 | 1000.545274 |
| MA0779 | 695.6267092 | 858.5436006 | 1036.389258 | 933.9201201 | 1560.349841 | 1041.479559 |
| MA0778 | 40.17315982 | 49.5817783 | 59.85254843 | 89.59485346 | 149.6908701 | 99.9134792 |
| MA0777 | 1049.720337 | 1295.566524 | 1563.940641 | 2355.719458 | 3935.825348 | 2627.027312 |
| MA0776 | 1870.116633 | 2308.100949 | 2786.219627 | 8597.160956 | 14363.73245 | 9587.294684 |
| MA0775 | 20.20981045 | 24.94298048 | 30.10987096 | 93.43333236 | 156.1040202 | 104.1940351 |
| MA0774 | 462.6730188 | 571.0317821 | 689.3199188 | 450.8834491 | 753.3148747 | 502.8116278 |
| MA0773 | 841.4867346 | 1038.564278 | 1253.700873 | 704.3683409 | 1176.825518 | 785.4903363 |
| MA0772 | 79.85383623 | 98.55573279 | 118.9713635 | 199.9873673 | 334.1294937 | 223.0198822 |
| MA0771a | 84.45591753 | 104.2356289 | 125.8278392 | 96.5032747 | 161.2331356 | 107.6175423 |
| MA0771 | 109.0342005 | 134.5701852 | 162.4461405 | 368.6309655 | 615.891291 | 411.086138 |
| MA0770 | 39.17108708 | 48.34501852 | 58.35959623 | 108.4214841 | 181.1455196 | 120.90837 |
| MA0769 | 138.0778324 | 170.4158823 | 205.7172049 | 264.1609321 | 441.3476694 | 294.5843067 |
| MA0768 | 145.0756204 | 179.0525635 | 216.1429581 | 394.1809407 | 658.5789888 | 439.5787001 |
| MA0767 | 162.9756215 | 201.1447735 | 242.8115269 | 331.3274886 | 553.5663951 | 369.4864253 |
| MA0766 | 86.61466733 | 106.8999614 | 129.044083 | 139.3727411 | 232.8574252 | 155.4242787 |
| MA0765 | 237.6920005 | 293.3598483 | 354.1287773 | 255.4871656 | 426.8559481 | 284.9115839 |
| MA0764 | 133.3665905 | 164.6012599 | 198.6980947 | 143.8957287 | 240.4142202 | 160.468178 |
| MA0763 | 1082.34335 | 1335.829899 | 1612.544496 | 7666.318012 | 12808.52381 | 8549.246699 |
| MA0762 | 612.1370242 | 755.5004974 | 912.000974 | 2903.740939 | 4851.433882 | 3238.164344 |
| MA0761 | 451.8448166 | 557.6675976 | 673.1873691 | 524.9685661 | 877.0928061 | 585.4291165 |
| MA0760 | 94.82580523 | 117.034161 | 141.2775626 | 3133.274828 | 5234.928314 | 3494.133616 |
| MA0759 | 2279.110399 | 2812.881711 | 3395.564754 | 3377.690313 | 5643.28622 | 3766.698396 |
| MA0758 | 1825.521561 | 2253.06164 | 2719.77903 | 8742.97585 | 14607.35312 | 9749.903057 |
| MA0757 | 536.0602146 | 661.6063768 | 798.6568668 | 927.2112003 | 1549.140893 | 1033.997974 |
| MA0756 | 28.2255036 | 34.83596183 | 42.0521644 | 66.25887966 | 110.7022219 | 73.88990482 |
| MA0755 | 45.45679991 | 56.10285537 | 67.72445408 | 88.2554523 | 147.4530616 | 98.41981941 |
| MA0754 | 27.11094012 | 33.46036579 | 40.39161628 | 23.83744568 | 39.82648385 | 26.58280069 |
| MA0753 | 147.7052696 | 182.2980808 | 220.060778 | 845.3915467 | 1412.440462 | 942.7551634 |
| MA0752 | 885.9077681 | 1093.388789 | 1319.882176 | 2589.92738 | 4327.128937 | 2888.208925 |
| MA0751 | 148.4662288 | 183.2372578 | 221.1945037 | 242.7628185 | 405.5967069 | 270.7217756 |
| MA0750 | 830.8411167 | 1025.425439 | 1237.840349 | 1518.577901 | 2537.168583 | 1693.472289 |
| MA0749 | 2243.026055 | 2768.346356 | 3341.803985 | 3507.142974 | 5859.56964 | 3911.0601 |
| MA0748 | 153.41155 | 189.3407811 | 228.5623601 | 341.2391791 | 570.1263817 | 380.5396437 |
| MA0747 | 624.0481925 | 770.2012804 | 929.7469961 | 1698.145742 | 2837.182091 | 1893.720931 |
| MA0746 | 83.7383871 | 103.3500517 | 124.7588164 | 237.0460663 | 396.0454262 | 264.346626 |
| MA0745 | 25.2284628 | 31.13700926 | 37.58698091 | 61.37918224 | 102.5494528 | 68.44821338 |
| MA0744 | 226.2010361 | 279.1776816 | 337.008802 | 420.6397289 | 702.7850884 | 469.0847429 |
| MA0743 | 89.41532985 | 110.3565436 | 133.2166895 | 391.4091581 | 653.9480246 | 436.4876916 |
| MA0742a | 43.7844935 | 54.03889213 | 65.23294482 | 439.9412583 | 735.0331766 | 490.6092266 |
| MA0742 | 92.01769686 | 113.5683891 | 137.0938627 | 216.1867721 | 361.1946978 | 241.08497 |
| MA0741 | 42.15582626 | 52.0287884 | 62.80645196 | 212.8902708 | 355.6870584 | 237.4088111 |
| MA0740 | 43.68802094 | 53.91982554 | 65.08921381 | 108.524489 | 181.3176154 | 121.023238 |
| MA0739 | 269.2682219 | 332.3312715 | 401.1730559 | 1534.397638 | 2563.599455 | 1711.113982 |
| MA0738 | 26.57672613 | 32.80103802 | 39.59571004 | 69.91154066 | 116.8049162 | 77.96324223 |
| MA0737 | 503.0209707 | 620.829289 | 749.4328836 | 2243.844779 | 3748.910392 | 2502.268043 |
| MA0736 | 294.5265061 | 363.5050864 | 438.8044667 | 1351.245481 | 2257.59744 | 1506.868219 |
| MA0735 | 186.8852022 | 230.6540164 | 278.4335528 | 762.1292827 | 1273.329785 | 849.903597 |
| MA0734 | 84.20597249 | 103.9271463 | 125.4554551 | 451.3363227 | 754.0715146 | 503.3166588 |
| MA0732n | 463.1887962 | 571.6683554 | 690.088357 | 848.4788263 | 1417.598543 | 946.1980045 |
| MA0731 | 53.62562222 | 66.18482898 | 79.89488916 | 329.4707465 | 550.4642378 | 367.4158426 |
| MA0730 | 9.486942969 | 11.70880023 | 14.13425571 | 57.38033481 | 95.86836645 | 63.9888193 |
| MA0729 | 184.4426394 | 227.6394015 | 274.7944662 | 815.1504446 | 1361.91505 | 909.0311981 |
| MA0728 | 1732.668422 | 2138.462146 | 2581.440472 | 1917.790453 | 3204.15415 | 2138.66209 |
| MA0727 | 867.4740287 | 1070.637838 | 1292.418409 | 2454.104154 | 4100.201875 | 2736.742958 |
| MA0726 | 763.115921 | 941.838894 | 1136.939011 | 5604.529436 | 9363.784362 | 6250.002244 |
| MA0725 | 752.6113066 | 928.8740821 | 1121.288564 | 2284.755909 | 3817.262787 | 2547.890902 |
| MA0724 | 1570.885757 | 1938.789721 | 2340.406288 | 2175.425817 | 3634.599207 | 2425.96928 |
| MA0723 | 532.6082171 | 657.3459161 | 793.5138598 | 1084.131746 | 1811.316366 | 1208.991036 |
| MA0722 | 986.1498937 | 1217.107781 | 1469.22932 | 7812.492236 | 13052.74483 | 8712.255786 |
| MA0721 | 215.4256634 | 265.8786994 | 320.9549612 | 1444.901041 | 2414.072747 | 1611.310076 |
| MA0720 | 2968.351236 | 3663.543857 | 4422.43993 | 15331.04567 | 25614.39052 | 17096.71988 |
| MA0719 | 376.8764305 | 465.1414952 | 561.4946624 | 1304.281449 | 2179.132142 | 1454.495346 |
| MA0718 | 432.5395082 | 533.840955 | 644.4250833 | 1500.87309 | 2507.588216 | 1673.728417 |
| MA0716 | 4.90213143 | 6.050218475 | 7.303509609 | 47.67459181 | 79.65246723 | 53.16526734 |
| MA0715 | 111.0237873 | 137.0257364 | 165.4103544 | 1564.046904 | 2613.136055 | 1744.177949 |
| MA0714 | 2965.758249 | 3660.343589 | 4418.576732 | 4878.236979 | 8150.329061 | 5440.062794 |
| MA0713 | 2309.384022 | 2850.245465 | 3440.668337 | 15410.55798 | 25747.23596 | 17185.38961 |
| MA0712 | 433.1197288 | 534.5570642 | 645.2895331 | 1878.069663 | 3137.790522 | 2094.366663 |
| MA0711 | 324.3599142 | 400.3255265 | 483.2521903 | 1364.718492 | 2280.107513 | 1521.892915 |
| MA0710 | 3228.00504 | 3984.009 | 4809.28881 | 4378.077341 | 7314.685846 | 4882.299847 |
| MA0709 | 1735.867288 | 2142.410191 | 2586.206347 | 8816.660315 | 14730.46166 | 9832.073751 |
| MA0708 | 1123.783204 | 1386.975033 | 1674.284247 | 7194.390456 | 12020.04943 | 8022.967318 |
| MA0707 | 96.5476725 | 119.1592923 | 143.8429108 | 474.0217622 | 791.9732807 | 528.614777 |
| MA0706 | 58.81459899 | 72.58907244 | 87.62575934 | 277.013231 | 462.8206864 | 308.9168029 |
| MA0705 | 151.5772238 | 187.0768528 | 225.8294634 | 474.5932741 | 792.9281359 | 529.2521097 |
| MA0704 | 19.16068283 | 23.64814549 | 28.5468134 | 1212.410189 | 2025.63796 | 1352.043288 |
| MA0703 | 451.3246204 | 557.0255705 | 672.4123474 | 404.3724966 | 675.6065612 | 450.9440159 |
| MA0702 | 5.967968928 | 7.365676825 | 8.89146263 | 86.89703649 | 145.1834843 | 96.90495505 |
| MA0701 | 246.6928852 | 304.4687547 | 367.5388723 | 4579.658105 | 7651.477512 | 5107.096637 |
| MA0700 | 104.257314 | 128.6745442 | 155.3292288 | 1278.938161 | 2136.789768 | 1426.233276 |
| MA0699 | 82.8350421 | 102.2351419 | 123.4129551 | 359.4647893 | 600.5768747 | 400.8642948 |
| MA0698 | 42.91723366 | 52.96851864 | 63.94084552 | 160.9805467 | 268.9587311 | 179.5206519 |
| MA0697 | 103.6257259 | 127.8950372 | 154.3882484 | 408.7027283 | 682.8413088 | 455.7729597 |
| MA0696 | 249.5048739 | 307.9393156 | 371.7283533 | 941.3507319 | 1572.764558 | 1049.765953 |
| MA0695 | 169.628666 | 209.3559717 | 252.7236591 | 3214.038686 | 5369.864772 | 3584.199035 |
| MA0694 | 687.7529327 | 848.825773 | 1024.658401 | 7035.446509 | 11754.49335 | 7845.71782 |
| MA0693 | 4332.313324 | 5346.948055 | 6454.558073 | 16248.15714 | 27146.65726 | 18119.45494 |
| MA0692 | 338.075311 | 417.2530912 | 503.6862675 | 618.4650626 | 1033.302358 | 689.6935904 |
| MA0691 | 73.94203387 | 91.25937683 | 110.1635814 | 153.1785671 | 255.9235504 | 170.8201195 |
| MA0690 | 166.6572251 | 205.6886146 | 248.2966159 | 924.2979541 | 1544.273579 | 1030.74921 |
| MA0689 | 817.507107 | 1008.968583 | 1217.974487 | 3316.060241 | 5540.317591 | 3697.970398 |
| MA0688 | 3994.973424 | 4930.602609 | 5951.967468 | 19112.91996 | 31932.96834 | 21314.15206 |
| MA0687 | 4708.061029 | 5810.696476 | 7014.371088 | 28042.51941 | 46852.1234 | 31272.17213 |
| MA0685 | 2692.893399 | 3323.573353 | 4012.045189 | 3966.414213 | 6626.89844 | 4423.225538 |
| MA0684 | 423.7254308 | 522.9626065 | 631.2933057 | 12950.23925 | 21636.65106 | 14441.71635 |
| MA0683 | 136.5546123 | 168.5359216 | 203.447814 | 1670.377194 | 2790.787706 | 1862.754283 |
| MA0682 | 266.8241685 | 329.3148168 | 397.5317485 | 2517.971024 | 4206.907638 | 2807.965366 |
| MA0681 | 1431.479999 | 1766.734911 | 2132.710654 | 9561.819147 | 15975.43801 | 10663.05241 |
| MA0680 | 300.5196659 | 370.9018538 | 447.7334603 | 737.8497883 | 1232.764746 | 822.8278369 |
| MA0679 | 3780.409977 | 4665.788059 | 5632.297092 | 11088.81921 | 18526.67794 | 12365.91684 |
| MA0678 | 3499.850054 | 4319.520551 | 5214.300935 | 11027.02832 | 18423.44064 | 12297.00951 |
| MA0677 | 594.3851726 | 733.5911337 | 885.5531277 | 1891.906693 | 3160.90878 | 2109.797301 |
| MA0676 | 2905.534916 | 3586.015855 | 4328.85215 | 5555.632722 | 9282.09003 | 6195.474103 |
| MA0675 | 3318.410235 | 4095.587235 | 4943.980263 | 17252.95518 | 28825.4266 | 19239.97542 |
| MA0674 | 3721.301754 | 4592.836595 | 5544.233872 | 20493.01198 | 34238.76122 | 22853.18908 |
| MA0673 | 342.2032533 | 422.3478042 | 509.8363405 | 2988.409895 | 4992.894794 | 3332.584611 |
| MA0672 | 1617.821896 | 1996.71838 | 2410.334756 | 3448.141856 | 5760.993346 | 3845.263833 |
| MA0671 | 801.2507716 | 988.9049878 | 1193.754756 | 3352.030469 | 5600.414959 | 3738.083312 |
| MA0670 | 719.8936641 | 888.4939152 | 1072.543723 | 4092.434504 | 6837.44722 | 4563.759568 |
| MA0669 | 1256.330487 | 1550.565103 | 1871.761685 | 5397.718251 | 9018.253955 | 6019.372647 |
| MA0668a | 1500.355871 | 1851.741623 | 2235.326343 | 3201.942717 | 5349.655397 | 3570.709976 |
| MA0667 | 26628.94199 | 32865.48293 | 39673.50458 | 686.3625991 | 1146.742371 | 765.4108758 |
| MA0666 | 3235.23218 | 3992.928747 | 4820.056267 | 590.8508995 | 987.1659122 | 658.8991083 |
| MA0665 | 1709.603904 | 2109.995881 | 2547.077474 | 2708.251908 | 4524.819996 | 3020.16087 |
| MA0664 | 2892.548888 | 3569.988479 | 4309.504734 | 7464.381411 | 12471.13763 | 8324.05309 |
| MA0663 | 778.7128675 | 961.0886704 | 1160.176342 | 1905.874482 | 3184.245506 | 2125.373758 |
| MA0662 | 1300.633175 | 1605.243551 | 1937.766669 | 3637.607906 | 6077.544317 | 4056.550659 |
| MA0661 | 1009.052643 | 1245.374391 | 1503.351304 | 2472.275567 | 4130.561817 | 2757.00717 |
| MA0660 | 2786.126284 | 3438.64153 | 4150.949517 | 9167.40354 | 15316.46695 | 10223.21202 |
| MA0659 | 5260.119231 | 6492.047594 | 7836.862781 | 14202.27373 | 23728.49142 | 15837.94744 |
| MA0658 | 21609.90251 | 24328.96031 | 37297.17691 | 20771.88645 | 34704.69158 | 23164.18148 |
| MA0657 | 1610.966133 | 1988.256985 | 2400.1206 | 5638.129397 | 9419.921597 | 6287.471908 |
| MA0656 | 357.8756162 | 441.6906597 | 533.1860313 | 457.6308359 | 764.5880915 | 510.3361102 |
| MA0655 | 2390.260531 | 2950.063382 | 3561.163344 | 5850.492329 | 9774.727603 | 6524.292646 |
| MA0654 | 495.2853494 | 611.2819728 | 737.9078592 | 791.7368527 | 1322.796721 | 882.9210663 |
| MA0653 | 466.474285 | 575.723311 | 694.9832889 | 11958.11565 | 19979.05757 | 13335.33002 |
| MA0652 | 468.855917 | 578.662725 | 698.5315968 | 2866.875247 | 4789.840416 | 3197.052836 |
| MA0651 | 1568.825044 | 1936.246387 | 2337.336106 | 9993.831477 | 16697.22391 | 11144.81954 |
| MA0650 | 131.1401316 | 161.8533608 | 195.3809736 | 344.3848386 | 575.3820017 | 384.0475884 |
| MA0649 | 141.1473611 | 174.2042996 | 210.2903856 | 490.0382679 | 818.7329056 | 546.4759011 |
| MA0648 | 1131.538558 | 1396.546703 | 1685.83867 | 4408.002088 | 7364.682706 | 4915.671022 |
| MA0647 | 898.2025426 | 1108.563019 | 1338.199719 | 3056.888399 | 5107.305459 | 3408.949774 |
| MA0646 | 2503.680179 | 3090.046094 | 3730.143206 | 52674.50091 | 88006.08036 | 58741.01522 |
| MA0645 | 536.7923614 | 662.5099936 | 799.747666 | 8213.122815 | 13722.09957 | 9159.026929 |
| MA0644 | 4566.240927 | 5635.661878 | 6803.07841 | 59444.26206 | 99316.67909 | 66290.44874 |
| MA0643 | 185.8861852 | 229.421028 | 276.9451532 | 2110.766764 | 3526.5699 | 2353.863453 |
| MA0642 | 1850.266873 | 2283.602343 | 2756.646181 | 20831.09588 | 34803.61591 | 23230.21004 |
| MA0641 | 1238.432243 | 1528.475061 | 1845.095733 | 8559.994033 | 14301.6357 | 9545.847252 |
| MA0640 | 92.88896153 | 114.6437053 | 138.3919287 | 658.9995858 | 1101.025534 | 734.8964685 |
| MA0639 | 2587.395714 | 3193.367941 | 3854.867976 | 6955.371646 | 11620.70803 | 7756.420747 |
| MA0638 | 291.1279622 | 359.310598 | 433.7410982 | 1137.66946 | 1900.764662 | 1268.69468 |
| MA0637 | 769.589987 | 949.8291967 | 1146.58449 | 4638.426806 | 7749.665495 | 5172.63372 |
| MA0636 | 954.882455 | 1178.517458 | 1422.645085 | 3537.968735 | 5911.071874 | 3945.43606 |
| MA0635 | 219.9498832 | 271.4624987 | 327.6954338 | 631.2737493 | 1054.70251 | 703.9774516 |
| MA0634 | 47.67241345 | 58.83736916 | 71.02541715 | 406.7166078 | 679.5229921 | 453.558098 |
| MA0633 | 137.1584519 | 169.2811813 | 204.3474529 | 999.1872948 | 1669.395169 | 1114.263544 |
| MA0631 | 18071.77481 | 22304.21347 | 26924.48844 | 219668.4062 | 367011.6484 | 244967.5833 |
| MA0630 | 4499.797407 | 5553.657179 | 6704.086594 | 63259.42599 | 105690.8757 | 70545.00452 |
| MA0629 | 1056.25005 | 1303.625507 | 1573.669026 | 4558.629589 | 7616.344056 | 5083.646269 |
| MA0628 | 1045.516654 | 1290.378332 | 1557.677724 | 5106.72344 | 8532.073501 | 5694.864006 |
| MA0627 | 2096.840666 | 2587.924114 | 3124.007623 | 7664.697616 | 12805.81653 | 8547.439682 |
| MA0626 | 838.1736046 | 1034.475208 | 1248.764759 | 2216.002475 | 3702.392781 | 2471.219145 |
| MA0624 | 140.0999529 | 172.9115867 | 208.7298897 | 60.51321425 | 101.1026342 | 67.48251199 |
| MA0623 | 183.1350037 | 226.0255154 | 272.8462667 | 533.2498999 | 890.9288694 | 594.6642103 |
| MA0622 | 57.05587829 | 70.41845652 | 85.00550451 | 505.614554 | 844.7570324 | 563.8461058 |
| MA0621 | 1370.210477 | 1691.115969 | 2041.427394 | 1945.694509 | 3250.774936 | 2169.779852 |
| MA0620 | 185.6397703 | 229.1169023 | 276.5780284 | 644.7426896 | 1077.205782 | 718.9976077 |
| MA0619 | 428.5712689 | 528.9433476 | 638.512946 | 634.9682112 | 1060.875043 | 708.0974041 |
| MA0618 | 169.0703928 | 208.66695 | 251.8919079 | 670.0248945 | 1119.446102 | 747.1915604 |
| MA0617 | 962.7656135 | 1188.246865 | 1434.38992 | 5389.406193 | 9004.366559 | 6010.10329 |
| MA0616 | 664.6631248 | 820.328295 | 990.2577254 | 650.9707452 | 1087.611325 | 725.9429475 |
| MA0615 | 334.4364576 | 412.7620125 | 498.2648704 | 437.2962397 | 730.6140039 | 487.6595816 |
| MA0614 | 331.7519331 | 409.448768 | 494.2652938 | 1095.666344 | 1830.58783 | 1221.854072 |
| MA0613 | 157.1342362 | 193.9353265 | 234.1086568 | 1438.844885 | 2403.954406 | 1604.556433 |
| MA0612 | 359.5865299 | 443.8022721 | 535.7350601 | 823.5752744 | 1375.990859 | 918.4263143 |
| MA0611 | 469.5212974 | 579.4839386 | 699.5229232 | 920.991125 | 1538.748684 | 1027.061534 |
| MA0610 | 4652.25902 | 5741.825546 | 6931.233678 | 12495.96845 | 20877.67676 | 13935.12725 |
| MA0609 | 4686.18946 | 5783.70255 | 6981.785422 | 9522.316929 | 15909.43956 | 10619.00073 |
| MA0608 | 9601.925043 | 11850.71129 | 14305.56337 | 75278.8524 | 125772.3684 | 83948.70645 |
| MA0606 | 1030.138836 | 1271.399004 | 1534.766865 | 8137.684855 | 13596.06137 | 9074.90079 |
| MA0605 | 1793.720133 | 2213.812266 | 2672.399224 | 18307.26895 | 30586.92449 | 20415.71434 |
| MA0604 | 1058.11663 | 1305.929243 | 1576.449977 | 4324.937011 | 7225.90148 | 4823.039353 |
| MA0603 | 686.4079275 | 847.1657653 | 1022.654526 | 3745.441101 | 6257.706951 | 4176.802986 |
| MA0602 | 1094.502044 | 1350.836179 | 1630.659298 | 5493.83519 | 9178.841618 | 6126.559358 |
| MA0601 | 1248.913261 | 1541.41075 | 1860.711026 | 8844.102948 | 14776.31152 | 9862.676948 |
| MA0600 | 2170.197533 | 2678.461276 | 3233.299384 | 39243.28635 | 65565.83836 | 43762.92971 |
| MA0599 | 600.769008 | 741.4700736 | 895.0641749 | 10974.54307 | 18335.75075 | 12238.47954 |
| MA0598 | 187.0423703 | 230.8479935 | 278.6677119 | 3135.889694 | 5239.297109 | 3497.049636 |
| MA0597 | 1421.45854 | 1754.366411 | 2117.780042 | 15668.08264 | 26177.49606 | 17472.57335 |
| MA0596 | 2515.347681 | 3104.446144 | 3747.526198 | 33573.59374 | 56093.18242 | 37440.25946 |
| MA0595 | 1730.976703 | 2136.374224 | 2578.92004 | 19493.06131 | 32568.09063 | 21738.07423 |
| MA0594 | 886.3924348 | 1093.986965 | 1320.604263 | 1680.630715 | 2807.918807 | 1874.188701 |
| MA0593 | 141.027327 | 174.0561532 | 210.111551 | 1207.997087 | 2018.264756 | 1347.121929 |
| MA0592 | 3140.102799 | 3875.519913 | 4678.326418 | 28935.10781 | 48343.41814 | 32267.5598 |
| MA0591 | 591.4476409 | 729.9656274 | 881.1766047 | 3181.084097 | 5314.805794 | 3547.449072 |
| MA0590 | 1732.806887 | 2138.633041 | 2581.646766 | 5282.343848 | 8825.491824 | 5890.71059 |
| MA0589 | 6.898958672 | 8.514705862 | 10.27851082 | 41.09477691 | 68.65922175 | 45.82765615 |
| MA0588 | 1113.241737 | 1373.964738 | 1658.578894 | 2733.240269 | 4566.569376 | 3048.027138 |
| MA0587 | 693.8697899 | 856.3752079 | 1033.771687 | 8405.540058 | 14043.58126 | 9373.604836 |
| MA0586 | 77.39893237 | 95.5258865 | 115.3138904 | 283.0502006 | 472.9069715 | 315.6490495 |
| MA0585 | 988.7697763 | 1220.341244 | 1473.132589 | 3906.090721 | 6526.112786 | 4355.954599 |
| MA0584 | 840.4634572 | 1037.301347 | 1252.176329 | 4054.167611 | 6773.512693 | 4521.085482 |
| MA0583 | 758.7981111 | 936.5098462 | 1130.506062 | 2973.710354 | 4968.335491 | 3316.192125 |
| MA0582 | 1577.204035 | 1946.58775 | 2349.819663 | 5626.253509 | 9400.079923 | 6274.228276 |
| MA0581 | 2220.128579 | 2740.086254 | 3307.689858 | 6425.196812 | 10734.91684 | 7165.185757 |
| MA0580 | 234.9802829 | 290.0130422 | 350.0886867 | 371.4987733 | 620.6826895 | 414.2842309 |
| MA0578 | 725.7574654 | 895.7310281 | 1081.27999 | 2969.617865 | 4961.497954 | 3311.628306 |
| MA0577 | 1173.941569 | 1448.880568 | 1749.013394 | 4822.934082 | 8057.931581 | 5378.390672 |
| MA0576 | 201.9184544 | 249.2080803 | 300.8310554 | 800.5251929 | 1337.479867 | 892.7215583 |
| MA0575 | 165.5652427 | 204.3408882 | 246.6697106 | 1269.484272 | 2120.994655 | 1415.690584 |
| MA0574 | 262.8808322 | 324.4479448 | 391.6567133 | 1713.926105 | 2863.547179 | 1911.318715 |
| MA0573 | 902.3587357 | 1113.692599 | 1344.391882 | 4764.53011 | 7960.352969 | 5313.260323 |
| MA0572 | 45.03350254 | 55.58042109 | 67.09379854 | 188.9275773 | 315.6513163 | 210.6863378 |
| MA0571 | 31.93731447 | 39.41708475 | 47.58225813 | 253.5358328 | 423.595753 | 282.7355164 |
| MA0569 | 31.22732611 | 38.54081598 | 46.52447197 | 74.39525453 | 124.2960946 | 82.96334474 |
| MA0568 | 314.6478361 | 388.3388642 | 468.7825139 | 484.1545861 | 808.9027266 | 539.9145966 |
| MA0567 | 47.62042093 | 58.77319991 | 70.94795537 | 387.804833 | 647.9260877 | 432.468257 |
| MA0566 | 307.1677467 | 379.1069258 | 457.6381972 | 1760.137374 | 2940.754797 | 1962.85213 |
| MA0565a | 9.432881125 | 11.64207702 | 14.05371091 | 46.26402656 | 77.29576111 | 51.59224751 |
| MA0565 | 73.34443305 | 90.52181694 | 109.2732374 | 766.4003261 | 1280.465643 | 854.6665358 |
| MA0564 | 356.5711591 | 440.0806966 | 531.2425674 | 1272.840588 | 2126.60223 | 1419.433446 |
| MA0563 | 163.8824724 | 202.2640104 | 244.1626116 | 611.2396593 | 1021.230494 | 681.6360385 |
| MA0562 | 60.78610564 | 75.02230911 | 90.56303632 | 357.2154674 | 596.8188134 | 398.3559188 |
| MA0561 | 140.1966431 | 173.0309218 | 208.8739448 | 1094.074657 | 1827.928513 | 1220.079069 |
| MA0560 | 341.9044145 | 421.9789769 | 509.3911114 | 1730.952726 | 2891.994458 | 1930.30629 |
| MA0559 | 50.91465768 | 62.83895219 | 75.85592042 | 284.2837621 | 474.9679481 | 317.0246801 |
| MA0558 | 133.2667984 | 164.4780963 | 198.549418 | 585.5077426 | 978.2388167 | 652.9405808 |
| MA0557 | 68.09135626 | 84.03846114 | 101.4468669 | 380.3976668 | 635.5505425 | 424.2080086 |
| MA0556 | 140.748974 | 173.7126095 | 209.6968428 | 1079.471768 | 1803.530694 | 1203.794368 |
| MA0555 | 138.5475616 | 170.9956228 | 206.4170376 | 1844.036545 | 3080.929588 | 2056.413955 |
| MA0554 | 273.2332844 | 337.224958 | 407.0804602 | 1556.668743 | 2600.808971 | 1735.950047 |
| MA0552 | 512.3641895 | 632.3607047 | 763.3530099 | 1587.866746 | 2652.933128 | 1770.741119 |
| MA0551 | 2984.508939 | 3683.48572 | 4446.512711 | 14210.04084 | 23741.46835 | 15846.6091 |
| MA0550 | 260.7659265 | 321.8377249 | 388.5057913 | 1632.444392 | 2727.411363 | 1820.452766 |
| MA0549 | 116.5227046 | 143.8125089 | 173.6029939 | 271.2514022 | 453.1940936 | 302.4913852 |
| MA0548 | 709.9094916 | 876.1714335 | 1057.668663 | 2775.134996 | 4636.565116 | 3094.746874 |
| MA0547 | 1249.538953 | 1542.182981 | 1861.643223 | 5259.739714 | 8787.725899 | 5865.503142 |
| MA0546 | 53.40965944 | 65.91828737 | 79.57313396 | 150.1871419 | 250.9256177 | 167.4841723 |
| MA0545 | 2393.772597 | 2954.39798 | 3566.395845 | 4534.332929 | 7575.750339 | 5056.551367 |
| MA0544 | 825.1632836 | 1018.417848 | 1229.381149 | 2124.023222 | 3548.718168 | 2368.646656 |
| MA0543 | 505.6923219 | 624.126275 | 753.4128338 | 1560.717785 | 2607.57392 | 1740.465417 |
| MA0542 | 422.017331 | 520.8544669 | 628.7484692 | 1126.490481 | 1882.087349 | 1256.228219 |
| MA0541 | 1265.800795 | 1562.253372 | 1885.871156 | 3723.757743 | 6221.479416 | 4152.622359 |
| MA0540 | 543.1727701 | 670.3847043 | 809.253608 | 2410.503832 | 4027.356507 | 2688.121195 |
| MA0539 | 7928.28528 | 9785.102412 | 11812.06758 | 6355.170317 | 10617.91986 | 7087.094322 |
| MA0538 | 865.7492996 | 1068.509175 | 1289.848797 | 5864.684384 | 9798.439021 | 6540.119198 |
| MA0537 | 90.16800779 | 111.2854999 | 134.3380774 | 765.4180206 | 1278.824453 | 853.5710983 |
| MA0536 | 2132.87641 | 2632.399487 | 3177.695983 | 3811.231583 | 6367.626596 | 4250.170548 |
| MA0535 | 691.7424484 | 853.7496396 | 1030.602237 | 2238.955258 | 3740.741211 | 2496.815396 |
| MA0534 | 984.7999683 | 1215.441701 | 1467.218114 | 5599.61222 | 9355.568908 | 6244.518713 |
| MA0533 | 135.7236926 | 167.5103991 | 202.2098565 | 863.1219069 | 1442.063514 | 962.5275264 |
| MA0532 | 1059.831967 | 1308.046314 | 1579.005595 | 850.744253 | 1421.383512 | 948.7243401 |
| MA0531 | 191.8317039 | 236.7589968 | 285.8031681 | 182.6478464 | 305.1594371 | 203.6833713 |
| MA0530 | 765.5748643 | 944.8737258 | 1140.602503 | 512.8273152 | 856.8077747 | 571.8895596 |
| MA0529 | 1483.469262 | 1830.900143 | 2210.16759 | 727.2130912 | 1214.993453 | 810.966113 |
| MA0528 | 2561.010765 | 3160.803595 | 3815.557988 | 1151.697628 | 1924.202266 | 1284.33847 |
| MA0527 | 1718.264868 | 2120.685257 | 2559.981134 | 521.5058265 | 871.3074234 | 581.5675737 |
| MA0526 | 424.3502284 | 523.7337327 | 632.2241692 | 1255.43718 | 2097.525434 | 1400.025691 |
| MA0525 | 155.6415621 | 192.0930656 | 231.8847752 | 346.0748451 | 578.2055852 | 385.9322327 |
| MA0524 | 339.7493406 | 419.3191812 | 506.1803441 | 2323.031607 | 3881.212024 | 2590.574806 |
| MA0523 | 4673.278441 | 5767.76775 | 6962.549759 | 21595.53035 | 36080.7971 | 24082.68432 |
| MA0522 | 1738.91169 | 2146.167597 | 2590.742092 | 10967.91192 | 18324.67173 | 12231.08468 |
| MA0521 | 1480.54905 | 1827.296013 | 2205.816871 | 6807.79281 | 11374.14025 | 7591.845279 |
| MA0520 | 404.2562681 | 498.9337346 | 602.2868993 | 2326.836804 | 3887.569568 | 2594.818247 |
| MA0519 | 76.33824538 | 94.21678492 | 113.7336109 | 429.9850181 | 718.3987584 | 479.5063276 |
| MA0518 | 47.30965824 | 58.3896561 | 70.48496119 | 79.60440261 | 132.9992944 | 88.77242962 |
| MA0517 | 177.8143286 | 219.4587297 | 264.9191839 | 302.4719186 | 505.3558651 | 337.3075638 |
| MA0516 | 140.3516024 | 173.2221729 | 209.1048132 | 242.9467973 | 405.9040902 | 270.9269433 |
| MA0515 | 134.6393125 | 166.1720555 | 200.5942776 | 525.1666582 | 877.4237691 | 585.6500229 |
| MA0514 | 124.6035571 | 153.78591 | 185.6423659 | 338.3218804 | 565.2522963 | 377.2863603 |
| MA0513 | 191.604728 | 236.4788627 | 285.4650048 | 309.5055157 | 517.1072684 | 345.1512191 |
| MA0512 | 1229.671276 | 1517.662262 | 1832.043084 | 2083.295244 | 3480.671776 | 2323.228042 |
| MA0511 | 188.5423221 | 232.6992365 | 280.9024363 | 1265.415663 | 2114.197014 | 1411.153394 |
| MA0510 | 395.3715484 | 487.9681994 | 589.0498745 | 2393.676594 | 3999.242347 | 2669.355965 |
| MA0509 | 1373.134447 | 1694.724737 | 2045.78371 | 6254.366043 | 10449.50082 | 6974.680435 |
| MA0508 | 214.1418302 | 264.2941904 | 319.0422242 | 885.1769489 | 1478.912042 | 987.1226443 |
| MA0507 | 94.31808233 | 116.4075285 | 140.5211244 | 295.3873553 | 493.5193096 | 329.4070723 |
| MA0506 | 121.4864503 | 149.9387719 | 180.9983004 | 527.7667476 | 881.7678761 | 588.5495641 |
| MA0505 | 59.55951943 | 73.50845444 | 88.73558956 | 230.9132156 | 385.7989474 | 257.5074558 |
| MA0504 | 232.3501219 | 286.7668932 | 346.1701042 | 277.7210693 | 464.0033093 | 309.7061629 |
| MA0503 | 54.38105637 | 67.11718702 | 81.02038337 | 52.00671867 | 86.89038118 | 57.9963246 |
| MA0502 | 160.7208291 | 198.3619051 | 239.4521927 | 778.6813621 | 1300.984221 | 868.3619769 |
| MA0501 | 3.607431665 | 4.452297948 | 5.374582915 | 43.06488545 | 71.9507865 | 48.02466179 |
| MA0500 | 63.27153713 | 78.08983263 | 94.26599144 | 215.7316308 | 360.4342691 | 240.5774102 |
| MA0499 | 128.1279865 | 158.1357665 | 190.8932868 | 705.6140819 | 1178.906843 | 786.8795491 |
| MA0497 | 284.8930134 | 351.6154142 | 424.4518718 | 1213.453421 | 2027.380942 | 1353.206668 |
| MA0496 | 131.0563732 | 161.7499861 | 195.256185 | 335.462883 | 560.4756177 | 374.0980925 |
| MA0495 | 34.2728994 | 42.29966743 | 51.06196228 | 185.2654122 | 309.5327431 | 206.6024018 |
| MA0494 | 40.89112092 | 50.46788705 | 60.9222129 | 202.8582539 | 338.9260361 | 226.2214084 |
| MA0493 | 408.256711 | 503.8710877 | 608.2470156 | 2519.596672 | 4209.623696 | 2809.77824 |
| MA0492 | 170.2136113 | 210.0779121 | 253.595148 | 306.725462 | 512.4624854 | 342.0509871 |
| MA0491 | 171.4643233 | 211.6215429 | 255.458539 | 520.5471043 | 869.7056354 | 580.4984356 |
| MA0490 | 329.3701299 | 406.5091428 | 490.7167308 | 6842.305854 | 11431.80302 | 7630.333185 |
| MA0488 | 334.5432755 | 412.8938473 | 498.4240146 | 3084.37581 | 5153.230135 | 3439.602906 |
| MA0487 | 1124.804605 | 1388.235648 | 1675.805996 | 2422.842808 | 4047.971887 | 2701.881248 |
| MA0486 | 1185.218014 | 1462.797975 | 1765.813765 | 1266.417255 | 2115.870426 | 1412.270339 |
| MA0485 | 96.90456838 | 119.5997738 | 144.3746371 | 174.7600329 | 291.9808491 | 194.8871195 |
| MA0484 | 1143.637809 | 1411.479619 | 1703.864911 | 4315.519803 | 7210.167651 | 4812.537566 |
| MA0482 | 23.3272181 | 28.79049 | 34.75438469 | 107.0705245 | 178.8883998 | 119.4018206 |
| MA0481 | 115.7628571 | 142.874704 | 172.4709245 | 260.390705 | 435.0485513 | 290.3798632 |
| MA0480 | 204.7185723 | 252.6639904 | 305.0028506 | 605.593577 | 1011.797285 | 675.3396978 |
| MA0479 | 320.6704653 | 395.7720027 | 477.7554128 | 1277.291138 | 2134.037999 | 1424.396565 |
| MA0478 | 41333.1477 | 51013.4372 | 61580.77272 | 16335.3791 | 27292.38362 | 18216.72224 |
| MA0477 | 1099.002313 | 1356.390418 | 1637.364088 | 1762.803173 | 2945.208688 | 1965.824948 |
| MA0476a | 26.95148116 | 33.26356129 | 40.15404411 | 137.4714098 | 229.6807702 | 153.3039713 |
| MA0476 | 126.1204689 | 155.658085 | 187.9023583 | 404.9798033 | 676.6212208 | 451.6212661 |
| MA0475 | 90.40648816 | 111.5798327 | 134.6933807 | 841.952888 | 1406.695312 | 938.9204749 |
| MA0474 | 127.8850444 | 157.835927 | 190.5313361 | 787.7853955 | 1316.194812 | 878.5145204 |
| MA0472 | 71.99949098 | 88.86188728 | 107.2694565 | 804.4553922 | 1344.046259 | 897.1043981 |
| MA0471 | 116.9436589 | 144.3320514 | 174.2301586 | 536.685954 | 896.669667 | 598.4959942 |
| MA0470 | 43.13402985 | 53.23608884 | 64.26384237 | 253.1619661 | 422.971114 | 282.3185915 |
| MA0469 | 74.86757687 | 92.40168349 | 111.5425147 | 670.4379126 | 1120.136153 | 747.6521456 |
| MA0468 | 241.2435316 | 297.7431537 | 359.420076 | 496.3132531 | 829.216856 | 553.4735755 |
| MA0467 | 295.2061154 | 364.3438613 | 439.8169922 | 478.9380086 | 800.1871141 | 534.0972267 |
| MA0466 | 76.17170616 | 94.01124195 | 113.4854901 | 158.9525483 | 265.5704468 | 177.2590893 |
| MA0465 | 184.6274384 | 227.8674807 | 275.0697915 | 449.668376 | 751.2847876 | 501.4566149 |
| MA0464 | 7955.098283 | 9818.195064 | 11852.01531 | 6957.550517 | 11624.34839 | 7758.850559 |
| MA0463 | 3427.049427 | 4229.6699 | 5105.837895 | 8795.360667 | 14694.87521 | 9808.321025 |
| MA0462 | 160.9167987 | 198.6037711 | 239.7441608 | 288.0561399 | 481.2706595 | 321.231522 |
| MA0461 | 1612.607208 | 1990.282403 | 2402.565579 | 2227.006665 | 3720.778064 | 2483.490686 |
| MA0460 | 496.0619213 | 612.2404192 | 739.0648458 | 1663.68172 | 2779.601222 | 1855.287692 |
| MA0459 | 3045.871862 | 3759.219939 | 4537.935128 | 10071.93358 | 16827.71323 | 11231.91665 |
| MA0457 | 418.1141788 | 516.0371903 | 622.9333029 | 19895.28883 | 33240.11347 | 22186.62623 |
| MA0456 | 3118.523306 | 3848.886468 | 4646.175906 | 100560.297 | 168011.4178 | 112141.8112 |
| MA0455 | 5418.020532 | 6686.929633 | 8072.11426 | 150290.6686 | 251098.5852 | 167599.6222 |
| MA0454 | 57.90350262 | 71.46459583 | 86.26834957 | 357.5171939 | 597.3229239 | 398.6923951 |
| MA0453 | 126.4082381 | 156.0132503 | 188.3310954 | 628.3573693 | 1049.829959 | 700.7251925 |
| MA0452 | 313.0360365 | 386.3495785 | 466.3811515 | 1432.479472 | 2393.319373 | 1597.457916 |
| MA0451 | 137.2499367 | 169.3940921 | 204.4837529 | 1686.128282 | 2817.103883 | 1880.319421 |
| MA0450 | 74.97141794 | 92.52984431 | 111.6972238 | 120.2870905 | 200.9700171 | 134.1405365 |
| MA0449 | 1019.454904 | 1258.212878 | 1518.849259 | 1194.028565 | 1994.926806 | 1331.544655 |
| MA0448 | 34.03078153 | 42.00084516 | 50.70123955 | 145.7661413 | 243.5392178 | 162.5540058 |
| MA0447 | 540.5736972 | 667.1769243 | 805.3813425 | 2363.606498 | 3949.002644 | 2635.822701 |
| MA0446 | 20.1092036 | 24.81881135 | 29.95998044 | 33.8047217 | 56.47934014 | 37.69800638 |
| MA0445 | 426.7638193 | 526.7125905 | 635.8200918 | 1040.488994 | 1738.400107 | 1160.321954 |
| MA0444 | 255.2903131 | 315.0797141 | 380.3478715 | 315.6340454 | 527.3465278 | 351.9855707 |
| MA0443 | 169.7480684 | 209.5033384 | 252.9015525 | 361.4273316 | 603.8557981 | 403.0528627 |
| MA0442 | 56.62660567 | 69.88864757 | 84.36594665 | 663.7889301 | 1109.027345 | 740.2374008 |
| MA0441 | 1448.681401 | 1787.964909 | 2158.338406 | 5139.719687 | 8587.202079 | 5731.660426 |
| MA0440 | 272.6087111 | 336.4541086 | 406.1499308 | 2901.378604 | 4847.487003 | 3235.529939 |
| MA0439 | 1893.844317 | 2337.385695 | 2821.570651 | 11066.49437 | 18489.37865 | 12341.02085 |
| MA0438 | 58.29375433 | 71.94624512 | 86.84977159 | 662.8581543 | 1107.472249 | 739.1994277 |
| MA0437 | 1118.15662 | 1380.030694 | 1665.901401 | 7743.639913 | 12937.70961 | 8635.473752 |
| MA0436 | 820.4787751 | 1012.636221 | 1222.401868 | 5040.832865 | 8421.986626 | 5621.384825 |
| MA0435 | 5832.581104 | 7198.580956 | 8689.753172 | 16953.09059 | 28324.42694 | 18905.57547 |
| MA0434 | 188.5438527 | 232.7011254 | 280.9047165 | 222.4026109 | 371.5798291 | 248.016686 |
| MA0433 | 1312.100879 | 1619.39701 | 1954.85199 | 6575.141567 | 10985.43749 | 7332.399627 |
| MA0432 | 182.3721193 | 225.0839623 | 271.7096727 | 1020.012414 | 1704.188799 | 1137.487089 |
| MA0431 | 9597.604926 | 11845.3794 | 14299.12698 | 24660.47584 | 41201.56396 | 27500.61912 |
| MA0430 | 1223.844669 | 1510.471054 | 1823.362231 | 5723.734339 | 9562.946311 | 6382.935958 |
| MA0429 | 626.330589 | 773.0182178 | 933.1474565 | 2503.42141 | 4182.598828 | 2791.740076 |
| MA0428 | 850.2492419 | 1049.378978 | 1266.755818 | 3956.60205 | 6610.504741 | 4412.283311 |
| MA0427 | 181.0057973 | 223.3976454 | 269.6740385 | 967.8712093 | 1617.073725 | 1079.34079 |
| MA0426 | 13.51740883 | 16.68320767 | 20.13910209 | 61.85835807 | 103.3500372 | 68.98257582 |
| MA0425 | 205.3681058 | 253.4656457 | 305.9705673 | 477.3872283 | 797.5961433 | 532.3678432 |
| MA0424 | 568.6309616 | 701.8052451 | 847.1828534 | 2693.861244 | 4500.776751 | 3004.112836 |
| MA0422 | 80.83324445 | 99.76451999 | 120.4305486 | 415.6529676 | 694.453442 | 463.5236571 |
| MA0421 | 247.6209432 | 305.6141653 | 368.9215526 | 1004.619607 | 1678.471222 | 1120.321496 |
| MA0420 | 12.75167242 | 15.73813456 | 18.9982589 | 75.29389016 | 125.7974928 | 83.96547611 |
| MA0419 | 27.56843543 | 34.02500724 | 41.07322213 | 166.7849672 | 278.6564841 | 185.9935667 |
| MA0418 | 43.28805527 | 53.42618726 | 64.49331931 | 179.904371 | 300.5757674 | 200.6239302 |
| MA0417 | 199.5817688 | 246.3241393 | 297.3497116 | 1392.517758 | 2326.55322 | 1552.893818 |
| MA0416 | 34.21850441 | 42.23253305 | 50.98092113 | 328.8128847 | 549.3651132 | 366.682215 |
| MA0415 | 71.50234513 | 88.24830908 | 106.5287768 | 524.2711027 | 875.9275169 | 584.6513263 |
| MA0414 | 27.00904316 | 33.3346044 | 40.23980366 | 91.11997232 | 152.238967 | 101.6142458 |
| MA0413 | 324.5533566 | 400.5642734 | 483.5403931 | 2142.654509 | 3579.846446 | 2389.423705 |
| MA0411n | 188.0136454 | 232.0467428 | 280.1147797 | 1229.347767 | 2053.936468 | 1370.931563 |
| MA0410 | 105.9266633 | 130.7348578 | 157.8163324 | 381.9931304 | 638.2161681 | 425.9872215 |
| MA0409 | 164.4856026 | 203.0083946 | 245.0611936 | 734.5920794 | 1227.32192 | 819.1949382 |
| MA0408 | 105.4215032 | 130.1113884 | 157.0637125 | 690.006541 | 1152.830498 | 769.47449 |
| MA0407 | 24.95793179 | 30.80311946 | 37.18392648 | 239.6805023 | 400.4469179 | 267.2844695 |
| MA0406 | 11.5508493 | 14.25607674 | 17.20919565 | 149.5465696 | 249.8553796 | 166.7698254 |
| MA0405 | 127.9133887 | 157.8709095 | 190.5735652 | 1198.733933 | 2002.788313 | 1336.791939 |
| MA0404 | 119.01078 | 146.8832957 | 177.3098882 | 598.1782557 | 999.4081145 | 667.070355 |
| MA0403 | 207.965003 | 256.6707404 | 309.8395912 | 730.0305213 | 1219.700683 | 814.1080261 |
| MA0402 | 32.66993677 | 40.32128837 | 48.67376579 | 129.6538608 | 216.6195768 | 144.5860764 |
| MA0401 | 12.05647213 | 14.88011724 | 17.96250494 | 58.16072301 | 97.17220238 | 64.85908469 |
| MA0400 | 82.8403371 | 102.241677 | 123.4208439 | 425.4690011 | 710.8536094 | 474.4702016 |
| MA0399 | 1290.645549 | 1592.916808 | 1922.886464 | 2516.687242 | 4204.762756 | 2806.533731 |
| MA0398 | 16.35363208 | 20.18367895 | 24.36468926 | 56.27611261 | 94.0234839 | 62.75742399 |
| MA0397 | 55.04313549 | 67.93432613 | 82.00679128 | 118.394955 | 197.8087261 | 132.030484 |
| MA0396 | 67.09306724 | 82.80637123 | 99.95955195 | 168.9354971 | 282.2494884 | 188.3917728 |
| MA0395 | 91.83142816 | 113.338496 | 136.8163477 | 249.4769738 | 416.8144022 | 278.2091992 |
| MA0394 | 375.232076 | 463.1120302 | 559.0447976 | 1201.022261 | 2006.611544 | 1339.343813 |
| MA0393 | 44.13243729 | 54.46832491 | 65.75133378 | 108.0261743 | 180.4850546 | 120.4675325 |
| MA0392 | 52.52233387 | 64.82314872 | 78.25113945 | 385.1914511 | 643.559772 | 429.5538923 |
| MA0391 | 70.84559389 | 87.43774564 | 105.5503067 | 191.6444033 | 320.1904614 | 213.7160602 |
| MA0390 | 21.26527801 | 26.24564024 | 31.68237419 | 112.8388832 | 188.5259026 | 125.8345204 |
| MA0389 | 22.89366318 | 28.25539582 | 34.10844679 | 93.1325618 | 155.6015069 | 103.8586249 |
| MA0388 | 139.869514 | 172.6271786 | 208.386567 | 443.1352689 | 740.3695794 | 494.1710909 |
| MA0387 | 29.32900918 | 36.19791018 | 43.69623774 | 155.8364984 | 260.364297 | 173.7841644 |
| MA0386 | 815.8468118 | 1006.919444 | 1215.500872 | 2729.782977 | 4560.793095 | 3044.17167 |
| MA0385 | 730.8068311 | 901.9629634 | 1088.802859 | 2761.370117 | 4613.567403 | 3079.396696 |
| MA0384 | 524.2574693 | 647.0394097 | 781.0723805 | 1789.824079 | 2990.353948 | 1995.957848 |
| MA0383 | 249.8464885 | 308.3609369 | 372.2373126 | 932.3385394 | 1557.707411 | 1039.715828 |
| MA0382 | 586.9627684 | 724.4303905 | 874.4947542 | 7499.117619 | 12529.17325 | 8362.789862 |
| MA0381 | 687.2992217 | 848.2658021 | 1023.982433 | 8087.103696 | 13511.55275 | 9018.494207 |
| MA0380 | 276.4596588 | 341.2069544 | 411.8873195 | 1848.638395 | 3088.618141 | 2061.545799 |
| MA0379 | 1612.856298 | 1990.58983 | 2402.936689 | 2694.68566 | 4502.154148 | 3005.032201 |
| MA0378 | 918.069353 | 1133.082668 | 1367.798566 | 3011.403883 | 5031.312069 | 3358.226814 |
| MA0377 | 395.1950536 | 487.7503691 | 588.7869211 | 1026.916132 | 1715.723208 | 1145.185908 |
| MA0376 | 52.24107122 | 64.47601392 | 77.83209634 | 158.5127236 | 264.8356084 | 176.7686101 |
| MA0375 | 400.4200371 | 494.1990522 | 596.5714365 | 1084.184189 | 1811.403985 | 1209.049518 |
| MA0374 | 480.6457052 | 593.2137005 | 716.0967789 | 520.9030794 | 870.300382 | 580.8954084 |
| MA0373 | 1556.143502 | 1920.594807 | 2318.442332 | 1763.385744 | 2946.18202 | 1966.474614 |
| MA0372 | 59.81218866 | 73.8202992 | 89.11203237 | 1319.741592 | 2204.962223 | 1471.736032 |
| MA0370 | 2188.614413 | 2701.19142 | 3260.738033 | 1933.944286 | 3231.143216 | 2156.676358 |
| MA0369 | 141.8009516 | 175.010962 | 211.2641466 | 232.9766636 | 389.2464593 | 259.8085507 |
| MA0368 | 3246.027521 | 4006.252375 | 4836.139858 | 6429.158503 | 10741.53585 | 7169.603716 |
| MA0367 | 207.5709652 | 256.1844182 | 309.2525283 | 637.2924437 | 1064.758261 | 710.6893181 |
| MA0366 | 444.207754 | 548.2419227 | 661.8091837 | 872.6544207 | 1457.98999 | 973.1578984 |
| MA0365 | 266.9761468 | 329.5023887 | 397.7581756 | 783.3242099 | 1308.741273 | 873.5395407 |
| MA0364 | 555.336645 | 685.3973783 | 827.3761285 | 3226.887674 | 5391.332257 | 3598.527839 |
| MA0363 | 222.0441945 | 274.0473011 | 330.815673 | 1574.615574 | 2630.793692 | 1755.963812 |
| MA0362 | 167.5446289 | 206.7838497 | 249.618727 | 541.9772787 | 905.5101636 | 604.39672 |
| MA0361 | 121.6323729 | 150.1188697 | 181.2157052 | 651.1719615 | 1087.947507 | 726.1673379 |
| MA0360 | 137.5038639 | 169.7074894 | 204.8620698 | 620.9792411 | 1037.502929 | 692.4973265 |
| MA0359b | 84.05833817 | 103.7449358 | 125.2355 | 194.9340841 | 325.6867055 | 217.3846131 |
| MA0359 | 56.76314967 | 70.05717039 | 84.56937865 | 138.9024973 | 232.0717639 | 154.8998769 |
| MA0358 | 101.5155759 | 125.2906867 | 151.2444117 | 396.4203104 | 662.3204224 | 442.0759777 |
| MA0357 | 13.09418208 | 16.16086053 | 19.50855174 | 99.18730264 | 165.7174833 | 110.6106893 |
| MA0356 | 29.95148858 | 36.96617525 | 44.62364744 | 125.8158457 | 210.2072014 | 140.3060376 |
| MA0355 | 6.912667622 | 8.53162547 | 10.2989353 | 101.0420313 | 168.8162768 | 112.6790268 |
| MA0353n | 31.71558296 | 39.14342337 | 47.25190833 | 334.8508687 | 559.4530931 | 373.4155927 |
| MA0352 | 256.6379826 | 316.7430099 | 382.3557159 | 746.2129987 | 1246.737605 | 832.1542369 |
| MA0350 | 27.05609568 | 33.3926767 | 40.30990552 | 39.9186798 | 66.69425397 | 44.51610811 |
| MA0349 | 47.74113863 | 58.92218989 | 71.12780833 | 145.9197768 | 243.7959047 | 162.7253355 |
| MA0348 | 1858.401625 | 2293.642267 | 2768.765856 | 5411.542958 | 9041.351625 | 6034.789543 |
| MA0347 | 17.56554552 | 21.67942447 | 26.1702756 | 66.17721616 | 110.5657824 | 73.79883614 |
| MA0346 | 998.6256656 | 1232.505399 | 1487.816524 | 2308.546668 | 3857.011268 | 2574.421639 |
| MA0345 | 55.42217994 | 68.4021434 | 82.57151599 | 326.627995 | 545.7147022 | 364.2456918 |
| MA0344 | 170.1779634 | 210.0339155 | 253.5420376 | 927.376951 | 1549.417822 | 1034.182814 |
| MA0343 | 152.3185873 | 187.9918448 | 226.9339943 | 647.9585643 | 1082.578714 | 722.5838542 |
| MA0342 | 536.461873 | 662.1021044 | 799.2552833 | 3429.864472 | 5730.456352 | 3824.881445 |
| MA0341 | 284.3533861 | 350.9494053 | 423.6479005 | 1743.047409 | 2912.201686 | 1943.793916 |
| MA0340 | 819.7531409 | 1011.740642 | 1221.320772 | 5111.382592 | 8539.857793 | 5700.059752 |
| MA0339 | 740.9429045 | 914.4729214 | 1103.90423 | 3712.513545 | 6202.693139 | 4140.083169 |
| MA0338 | 418.331799 | 516.3057775 | 623.2575275 | 3632.983022 | 6069.817278 | 4051.393127 |
| MA0337 | 1670.274326 | 2061.455253 | 2488.481748 | 7599.904573 | 12697.56336 | 8475.184434 |
| MA0336 | 2852.361758 | 3520.389457 | 4249.631371 | 42744.42783 | 71415.38097 | 47667.29712 |
| MA0335 | 1044.497595 | 1289.120608 | 1556.159465 | 7641.316525 | 12766.7525 | 8521.365795 |
| MA0334 | 661.0861042 | 815.9135304 | 984.9284508 | 7179.194848 | 11994.66132 | 8006.021633 |
| MA0333 | 31.01891152 | 38.28359036 | 46.21396256 | 383.3290026 | 640.4480805 | 427.4769458 |
| MA0332 | 564.3420042 | 696.511807 | 840.7928898 | 1390.002068 | 2322.350123 | 1550.088396 |
| MA0331 | 501.3779498 | 618.80147 | 746.9850058 | 1087.850261 | 1817.529086 | 1213.137811 |
| MA0330 | 225.2725387 | 278.0317287 | 335.6254671 | 397.1924818 | 663.6105301 | 442.9370799 |
| MA0329 | 186.7346047 | 230.4681488 | 278.209183 | 743.53445 | 1242.262412 | 829.1672002 |
| MA0328 | 622.6091041 | 768.4251552 | 927.6029499 | 1404.24166 | 2346.140965 | 1565.96796 |
| MA0327 | 195.2453167 | 240.9720831 | 290.8889872 | 542.614099 | 906.5741331 | 605.1068828 |
| MA0326 | 673.0813093 | 830.7180318 | 1002.799676 | 1515.096861 | 2531.352625 | 1689.590338 |
| MA0325 | 314.830548 | 388.5643675 | 469.0547297 | 7885.882306 | 13175.36151 | 8794.098179 |
| MA0324 | 258.9663771 | 319.6167181 | 385.8247072 | 2793.720744 | 4667.617311 | 3115.47314 |
| MA0323 | 179.3741109 | 221.3838155 | 267.2430476 | 3126.368663 | 5223.389819 | 3486.43207 |
| MA0322 | 489.0518278 | 603.5885505 | 728.6207592 | 4236.184938 | 7077.618689 | 4724.065717 |
| MA0321 | 277.4524613 | 342.4322729 | 413.3664604 | 1432.236434 | 2392.913317 | 1597.186888 |
| MA0320 | 215.6561948 | 266.1632216 | 321.2984216 | 784.6329301 | 1310.927821 | 874.9989861 |
| MA0319 | 20.83676022 | 25.71676289 | 31.04394092 | 75.18605527 | 125.6173273 | 83.8452219 |
| MA0317n | 206.5655292 | 254.9435075 | 307.7545653 | 1621.230265 | 2708.675327 | 1807.94711 |
| MA0316 | 148.4376696 | 183.20201 | 221.1519544 | 925.1275657 | 1545.659655 | 1031.674368 |
| MA0315 | 146.8512485 | 181.2440465 | 218.7884025 | 801.1183494 | 1338.470885 | 893.3830286 |
| MA0314 | 866.726612 | 1069.715375 | 1291.30486 | 1360.728626 | 2273.441432 | 1517.443536 |
| MA0313 | 288.9772467 | 356.6561815 | 430.5368243 | 743.6323381 | 1242.425959 | 829.2763621 |
| MA0312 | 59.97146577 | 74.01687927 | 89.34933361 | 159.6885317 | 266.8000933 | 178.0798358 |
| MA0311 | 1959.582892 | 2418.520349 | 2919.512193 | 1731.464301 | 2892.849173 | 1930.876783 |
| MA0310 | 37.8609903 | 46.72809497 | 56.40773007 | 132.4453408 | 221.2834503 | 147.6990506 |
| MA0309 | 4550.605445 | 5616.364541 | 6779.783666 | 20648.78978 | 34499.02744 | 23026.90778 |
| MA0308 | 1030.683789 | 1272.071586 | 1535.578771 | 6124.541117 | 10232.59544 | 6829.903592 |
| MA0307 | 3906.704224 | 4821.660622 | 5820.458356 | 23377.30813 | 39057.70765 | 26069.66917 |
| MA0306 | 8748.926341 | 10797.93893 | 13034.71123 | 48127.55661 | 80409.25953 | 53670.40003 |
| MA0305 | 6177.672917 | 7624.493826 | 9203.893075 | 26390.73802 | 44092.40469 | 29430.15533 |
| MA0304 | 3612.557405 | 4458.624145 | 5382.219571 | 17837.69523 | 29802.3828 | 19892.0599 |
| MA0303 | 406.9124001 | 502.211937 | 606.244175 | 6267.594835 | 10471.60287 | 6989.432785 |
| MA0302 | 838.976373 | 1035.465986 | 1249.960775 | 16784.20929 | 28042.26801 | 18717.24414 |
| MA0301 | 128.316122 | 158.3679636 | 191.173583 | 2103.11274 | 3513.781917 | 2345.327916 |
| MA0300 | 97.28288602 | 120.066694 | 144.938279 | 1029.359224 | 1719.80501 | 1147.91037 |
| MA0299 | 67.95594888 | 83.87134113 | 101.2451283 | 892.4588324 | 1491.07827 | 995.2431812 |
| MA0298 | 71.89594699 | 88.73409313 | 107.1151901 | 1225.284091 | 2047.147069 | 1366.399874 |
| MA0297 | 48.13824511 | 59.41229935 | 71.71944302 | 1389.813341 | 2322.034807 | 1549.877934 |
| MA0296 | 28.7022647 | 35.42438114 | 42.76247364 | 113.0516843 | 188.8814406 | 126.0718297 |
| MA0295 | 58.18279301 | 71.80929648 | 86.6844543 | 207.4004752 | 346.5149659 | 231.2867566 |
| MA0294 | 1355.73268 | 1673.247449 | 2019.857443 | 3826.435481 | 6393.028554 | 4267.125477 |
| MA0293 | 35.62156587 | 43.96419374 | 53.07129202 | 171.5584839 | 286.6318514 | 191.3168485 |
| MA0292 | 494.297438 | 610.0626911 | 736.4360055 | 2534.553036 | 4234.612085 | 2826.457126 |
| MA0291 | 298.3367999 | 368.2077571 | 444.4812866 | 1236.697487 | 2066.216035 | 1379.12775 |
| MA0290 | 110.3752711 | 136.2253367 | 164.4441535 | 562.5319236 | 939.8518982 | 627.3186402 |
| MA0289 | 1144.027242 | 1411.960257 | 1704.445112 | 3260.186957 | 5446.967134 | 3635.662196 |
| MA0287 | 239.8748194 | 296.053887 | 357.3808809 | 2937.955464 | 4908.597901 | 3276.319351 |
| MA0286 | 1405.551103 | 1734.733426 | 2094.080123 | 12270.08105 | 20500.27471 | 13683.22443 |
| MA0285 | 991.0631887 | 1223.171778 | 1476.549461 | 11914.59358 | 19906.34294 | 13286.79552 |
| MA0284 | 879.9161979 | 1085.993983 | 1310.95555 | 8827.713769 | 14748.92925 | 9844.400229 |
| MA0283 | 439.9893213 | 543.0355262 | 655.5242923 | 1960.0565 | 3274.770274 | 2185.79591 |
| MA0282 | 597.9200375 | 737.9538695 | 890.8195959 | 2403.045164 | 4014.894913 | 2679.803512 |
| MA0281 | 174.4754617 | 215.3378947 | 259.9447259 | 786.6729005 | 1314.336108 | 877.2738996 |
| MA0280 | 1863.175666 | 2299.534396 | 2775.878528 | 5548.224114 | 9269.712076 | 6187.212247 |
| MA0279 | 1483.429987 | 1830.85167 | 2210.109077 | 9317.994888 | 15568.0679 | 10391.14695 |
| MA0278 | 78.69143965 | 97.12110107 | 117.2395506 | 523.4982762 | 874.6363147 | 583.7894935 |
| MA0277 | 371.022854 | 457.9170016 | 552.7736288 | 3041.324769 | 5081.302478 | 3391.593683 |
| MA0276 | 5014.089359 | 6188.397131 | 7470.311709 | 60583.89516 | 101220.7245 | 67561.33321 |
[truncated: 26,023 more chars]
